# Introduction to Wilderness Medicine—A Medical School Elective

**DOI:** 10.21980/J8B93X

**Published:** 2020-01-15

**Authors:** Mark A Pittman, Trevor Slone, Matthew Wilson

**Affiliations:** * University of South Carolina School of Medicine Greenville, Prisma Health – Upstate Department of Emergency Medicine, Greenville, SC; ^ Georgetown University School of Medicine, Department of Emergency Medicine, Washington, DC

## Abstract

**Audience:**

This curriculum provides a capstone experience for fourth year medical students, integrating aspects of the basic sciences and clinical skills in the care of wilderness medicine conditions.

**Length of Curriculum:**

The duration of this course is 2 weeks.

**Introduction:**

Since passage of the Wilderness Act of 1964, leading to the protection and expansion of wilderness areas, there has been steady growth in participation in outdoor recreational activities.^1^ Between the years of 2000 to 2009, there was a 7.5% increase in the total number of individuals participating in outdoor recreation. Notably, during this same timeline, there has also been a 7.1% increase in the total number of people participating in “nature-based outdoor recreation.” 2 Acknowledging this rising interest in the outdoors, along with increasing accessibility to remote locations, it has become clear that healthcare providers must now attain the ability to both identify and treat conditions unique to these environments.

In addition to discrete medical conditions unique to environmental medicine, the practice of wilderness medicine also encompasses the management of the familiar, the “bread-and-butter” medical illnesses, occurring in the unfamiliar, nonclinical environment. Management of these conditions requires both a knowledge of core life support principles and an adaptability and awareness of the non-medical factors affecting a patient’s care.

Wilderness Medicine also teaches core principles of austere medicine – healthcare administration in a resource-limited environment. The skills acquired in a wilderness medical course provide not only training in the wilderness setting, but also encompass medical care necessary in instances of disaster relief, terrorist events, and international medical missions.^3^ Additionally, management of discrete wilderness medicine conditions provides a context to review toxicologic biomechanisms and pathophysiology shared by other, more common conditions.

For these myriad reasons, a wilderness medicine elective in medical school provides students with more than a divergent experience; it provides a review and expansion of core medical principles increasingly applicable to all specialties.

**Educational Goals:**

The primary objective of this course is to provide fourth year medical students an introduction to wilderness medicine. Students will be able to: explain fundamental concepts of practicing medicine in austere conditions; identify and initiate treatment for common wilderness medicine conditions; and utilize the non-medical aspects of providing care in austere environments.

**Educational Methods:**

The educational strategies used in this curriculum include a combination of lecture-based and experiential learning activities, structured through the lens of Kolb’s theory of experiential learning. Core knowledge is preferentially imparted during outdoor experiential components, allowing adaptable, true-environment training. Sessions are complemented by assigned pre-reading in *Auerbach’s Wilderness Medicine^4^* textbook to create a flipped outdoor-classroom experience. In addition to a final examination, the course will include a final multi-day expedition designed to allow students an opportunity to demonstrate their wilderness medicine knowledge. The course format opens it to adaptation as a longitudinal curriculum. Finally, this course may be adapted to serve resident education purposes.

**Research Methods:**

This curriculum has been used and vetted at the authors’ institutions with over 50 medical students. All individual comments were reviewed for applicability, trends noted, and the course was further refined. Student final assessment scores were reviewed to refine the content taught and clarity of assessment.

**Results:**

The current iteration of the curriculum received the following on a 5-point Likert scale by students on post-course evaluation forms: 4.91 for overall educational experience, 4.82 for curriculum effectiveness, and 5.00 for effective faculty instruction. As a result of comments, the use of the flipped-classroom model throughout the course has increased. Topics frequently encountered in spontaneous discussion due to regional importance have been included.

**Discussion:**

Overall, this course has proven both popular and successful. Due to the dynamic and divergent nature of this as a medical school course, the authors have noted increased levels of student engagement with the material. Increasing reliance on the flipped-classroom model with student-led scenarios and discussions has increased students’ ability to recall and apply their knowledge to scenarios during the final expedition. The broad range of conditions included in wilderness medicine provides a unique framework to highlight the relevance of the basic medical sciences and review core medical principles.

**Topics:**

Wilderness trauma stabilization, patient transportation, acute mountain sickness, high-altitude cerebral edema, high-altitude pulmonary edema, hypothermia, frostbite, orienteering, survival skills, expedition medical kits, marine envenomation, decompression illness, plant toxidromes, snake envenomation, arthropod envenomation, high-angle rescue, search and rescue, heat illness, lightning strike, tick-related illness, disaster response, international medicine.^4^

## USER GUIDE

[Table t1-jetem-5-1-c1]

**Table t1-jetem-5-1-c1:** 

List of Resources:	
Abstract	1
User Guide	4
Appendix A: Didactics and Hands on Curriculum Chart	8
Appendix B: Syllabus	14
Appendix C: Trailside Template Explanation	16
Appendix D: Session 1 Introduction to Wilderness Medicine and Wilderness Trauma	19
Appendix E: Session 2 Mountain Emergencies	26
Appendix F: Session 3 Navigation and Survival Skills	38
Appendix G: Session 4 Aquatic Medicine	42
Appendix H: Session 5 Hiking Emergencies	43
Appendix I: Session 6 High Angle Rescue and Safety	56
Appendix J: Session 7 Desert Medicine and Tick-Borne Diseases	57
Appendix K: Session 8 Extensions of Austere Medicine	74
Appendix L: Session 9 Course Expedition	75
Appendix M: Learner Assessment	110
Appendix N: Final Examination	111
Appendix O: Program Evaluation	116
Appendix P: Marine Envenomations	117
Appendix Q: Dive Physics	118
Appendix R: Decompression Illness	119
Appendix S: Toxic Plants	120

**Learner Audience:**

Medical students

**Length of Curriculum:**

The duration of this course is 2 weeks

**Topics:**

Wilderness trauma stabilization, patient transportation, acute mountain sickness, high-altitude cerebral edema, high-altitude pulmonary edema, hypothermia, frostbite, orienteering, survival skills, expedition medical kits, marine envenomation, decompression illness, plant toxidromes, snake envenomation, arthropod envenomation, high-angle rescue, search and rescue, heat illness, lightning strike, tick-related illness, disaster response, international medicine

**Objectives:**

The goals of this curriculum are:

1. Apply fundamental concepts of practicing medicine in austere conditions as demonstrated by successful patient care in final course expedition.
2. Identify and initiate treatment for common wilderness medicine conditions as demonstrated by successful patient care in final course expedition and obtaining 75% or greater on the final written examination.
3. Discuss and utilize non-medical aspects of providing care in austere environments as demonstrated by successful coordination and completion of the final course expedition and completion of two essays on the final examination.

### Brief introduction

Since passage of the Wilderness Act of 1964, there has been steady growth in participation in outdoor recreational activities.^1^ Between the years of 2000 to 2009, there was a 7.5% increase in the total number of individuals participating in outdoor recreation. Notably, during this same timeline, there has also been a 7.1% increase in the total number of people participating in “Nature-Based Outdoor Recreation.” 2 Acknowledging this rising interest in the outdoors, along with increasing accessibility to remote locations, it has become clear that healthcare providers must now attain the ability both to identify and treat conditions unique to these environments.

In addition to discrete medical conditions unique to environmental medicine, the practice of wilderness medicine also encompasses management of the familiar, the “bread-and-butter” medical illnesses, occurring in the unfamiliar, nonclinical environment. Management of these conditions requires both a knowledge of core life support principles and an adaptability and awareness of the non-medical factors affecting a patient’s care.

Wilderness Medicine also teaches core principles of austere medicine, that is, healthcare administration in a resource-limited environment. The skills acquired in a wilderness medical course provide not only training in the wilderness setting, but also encompass medical care necessary in instances of disaster relief, terrorist events, and international medical missions.^3^ Additionally, management of discrete wilderness medicine conditions provides a context to review toxicologic biomechanisms and pathophysiology shared by other, more common conditions.

The interest amongst students, residents and faculty in creation and participation of a wilderness medicine elective cannot be emphasized enough. McGraw and Glickman^5^ surveyed past participants of their own institutional wilderness medicine elective, and an impressive 40% of responders list their elective as the best course of medical school.^5^ The course format opens it to adaptation as a longitudinal curriculum. This course may be adapted to serve resident education purposes.

The wilderness medicine elective in medical school provides students with more than a divergent experience; it provides a review and expansion of core medical principles increasingly applicable to all specialties.

### Problem identification, general and targeted needs assessment

While there is increasing interest in providing medical students with an exposure to wilderness medicine, and even publications helping to guide topic selection for such courses, there are few published curricula allowing educators less experienced in curriculum development and education theory to start high quality programs at their institution.^3^ A search of MedEdPortal, a common source of high-quality, shared curricula, for “wilderness medicine” and “wilderness” yielded zero results. Similarly, the wilderness medicine section of the *Journal of Education and Teaching in Emergency Medicine* only has two entries, neither of which are full-course curricula. The provided curriculum seeks to provide an accessible, quality curriculum from which individual institutions may evolve their own rotation.

Many courses offered are four weeks in duration. While this is advantageous from the educational experience of the student, this duration requires logistical and curricular flexibility from one’s medical school. The provided curriculum is two weeks in duration, easing the burden of initiation of a wilderness medicine course.

The core requirements of this curriculum from an institutional standpoint are minimal – an educator, students, access to the core textbook, basic backpacking supplies often found in garages, and local parks suitable for outdoor education. The largest expense is the final expedition, which educators with minimal budget could elect to host at a local park as a single day event. In addition, while this curriculum is structured as a 2-week course, it could be adapted as a longitudinal course for students or residents, decreasing the burden of concentrated protected time.

### Goals of the curriculum

This course will provide a capstone experience for fourth year medical students, integrating aspects of the basic sciences and clinical skills in the care of wilderness medicine conditions.

### Objectives of the curriculum

The goals of this curriculum are:

1. Apply fundamental concepts of practicing medicine in austere conditions as demonstrated by successful patient care in final course expedition.
2. Identify and initiate treatment for common wilderness medicine conditions as demonstrated by successful patient care in final course expedition and obtaining 75% or greater on the final written examination.
3. Discuss and utilize non-medical aspects of providing care in austere environments as demonstrated by successful coordination and completion of the final course expedition and completion of two essays on the final examination.

Specific objectives for individual sessions are listed below:

**Session 1 Objectives:** After participation, the learner should be able to:
  1. Discuss core wilderness medicine principles and applicability
  2. Employ various methods of resource-constrained patient transportation
  3. Perform treatment and stabilization of common orthopedic injuries
**Session 2 Objectives:** After participation, the learner should be able to:
  1. Demonstrate initial diagnosis and management of acute mountain sickness (AMS), high-altitude pulmonary edema (HAPE), and high-altitude cerebral edema (HACE)
  2. Illustrate management of frostbite and hypothermia
**Session 3 Objectives:** After participation, the learner should be able to:
  1. Apply orienteering principles to navigate in wilderness settings
  2. Explain wilderness principles of survival, including obtaining water, shelter, and signaling
  3. List common expedition medical supplies
**Session 4 Objectives:** After participation, the learner should be able to:
  1. Recognize various marine envenomations and their treatment - including cnidarians, mollusks, and stingrays
  2. Discuss the use of hyperbarics in the treatment of decompression illness
**Session 5 Objectives:** After participation, the learner should be able to:
  1. Recognize arthropod envenomations and their treatment
  2. Recognize elapid and crotalid envenomation pathophysiology and manage envenomation
  3. Identify and manage common plant toxidromes
**Session 6 Objectives:** After participation, the learner should be able to:
  1. Apply basic high-angle techniques for personal safety and patient rescue
  2. Employ basic search and rescue strategy and organizational structure
**Session 7 Objectives:** After participation, the learner should be able to:
  1. Evaluate and manage initial phase of heat illness
  2. Manage lightning victims
  3. Assess patients with tick-related illness
**Session 8 Objectives:** After participation, the learner should be able to:
  1. Discuss extensions of austere medicine, including disaster response, international medicine in the developing world, and remote event medicine
**Session 9 Objectives:** After participation, the learner should be able to:
  1. Plan expedition and manage common wilderness medicine scenarios
  2. Teach other learners about a plant of toxicologic significance

### Educational strategies

(See curriculum chart) Please see the separate document of linked objectives and educational strategies

### Results and tips for successful implementation

This curriculum has been used and vetted at the authors’ institutions, with over 50 fourth year medical students having completed the curriculum in the proposed elective format over several years. Based upon a review of final examination responses, several questions have been modified for clarity and distinction between correct and incorrect answers.

This course is adaptable for implementation in many formats. While it may be offered as a longitudinal course, we find it is best received as an immersive experience, with each session corresponding to one day and the whole course lasting two weeks. Students are better able to integrate principles and strengthen technical skills when sessions are successive. Limiting the size of each course has also helped achieve high levels of student engagement. Due to the hands-on nature of the course and heavy emphasis on student-led teaching and discussion, the authors have found groups larger than 15–20 students are more difficult to effectively engage.

### Evaluation and feedback

The current iteration of the curriculum received the following on a 5-point Likert scale by students: 4.91 for overall educational experience, 4.82 for curriculum effectiveness, and 5.00 for effective faculty instruction. Several of the sessions were indoors due to logistical constraints; however, the participants clearly expressed a preference for the applicationbased, experiential sessions, even if it means more pre-class preparation on their part. As a result, we have further increased the use of the flipped-classroom model throughout the course. While not initially in the course curriculum, informal discussions of tick-borne illnesses occurred frequently, likely due to our geographic location. This has been added to the curriculum. We understand that other institutions may modify some elements to better reflect local disease and illness patterns. The length of sessions has been adjusted through experience with the curriculum. While the lectures and/or cases themselves may be short, we found that the value comes from the discussion and questions generated. As a result, we have lengthened many sessions and shortened a few. Allowing time for this discussion has enhanced student engagement in the course. We should note that discussions have varied and will likely continue to vary based upon the dynamics of each group.

## DIDACTICS AND HANDS-ON CURRICULUM

### Appendix A Curriculum Chart

Please see Trailside Template (Appendix C) for explanation of format used for all cases. Common abbreviations and approach to cases are also defined in this document.[Table t2-jetem-5-1-c1]

**Table t2-jetem-5-1-c1:**

Topic	Recommended Educational Strategy	Educational Content	Objectives	Learners	Timing, Resources Needed (Space, Instructors, Equipment, Citations of JETem pubs or other literature)	Recommended Assessment, Milestones Addressed
Session 1: Introduction to Wilderness Medicine and Wilderness Trauma Duration: 230 minutes	Hands-on patient scenario; fallen in woods from platform Scenario debrief with facilitated discussion Further practice through hands-on scenarios See supplement for full description of session	-Basic principles of wilderness assessment and stabilization -Indications for emergent orthopedic reduction -Methods of fracture stabilization -Methods of transportation, preconstructed and improvised	Discuss core wilderness medicine principles and applicability Employ various methods of resource-constrained patient transportation Perform treatment and stabilization of common orthopedic injuries	M4	0–60 minutes – Patient scenario 60–90 minutes – Course introduction (see syllabus) 90–150 minutes – Scenario debrief 150–210 minutes – Further scenarios 210–230 minutes – Final expedition discussion (logistics, patient UTM coordinates, expectations for student preparation for rescue) Instructors: 1–2 (or 1 instructor and 1 lay patient) Instructional Materials Multiple lengths of rope, up to 50 ft. An overnight backpack An environment with debris - sticks for immobilization Hiking poles, a bandana An EMS backboard with C-collar	Hands-on assessment during final expedition scenarios and final examination
Session 2: Mountain Emergencies Duration: 220 minutes	Hands-on scenarios during hike Post-scenario debriefs See supplement for full description of session	-Pathophysiology of acute mountain sickness (AMS), high-altitude pulmonary edema (HAPE), and high-altitude cerebral edema (HACE) -Treatment of AMS, HAPE, and HACE -Diagnosis and treatment of hypothermia -Diagnosis and treatment in local hypothermia conditions: frostbite spectrum	Demonstrate initial diagnosis and management of AMS, HAPE, and HACE Illustrate management of frostbite and hypothermia	M4	0–30 minutes – Introduction 30–75 minutes – HACE scenario 75–95 minutes – Hike 95–140 minutes – HAPE scenario 140–160 minutes – Hike 160–200 minutes – Hypothermia scenario 200–220 minutes – Conclusion Instructors: 1 Instructional Materials Backpack Tarp Emergency blanket	Hands-on assessment during final expedition scenarios and final examination
Session 3: Navigation and Survival Skills Duration: 225 minutes	Brief lecture on navigation and search and rescue principles Navigation exercises utilizing compass and topographic map Facilitated discussion of survival principles Hands-on practice building shelter Facilitated discussion of expedition medical supplies See supplement for full description of session	-Orienteering: use of compass for bearings, topographic map, and map grid system -Survival principles: water, shelter, signaling, fire -Common expedition medical supplies	Apply orienteering principles to navigate in wilderness settings Explain wilderness principles of survival, including obtaining water, shelter, and signaling List common expedition medical supplies and design med kit for example scenarios	M4	0–30 minutes – Introduction to orienteering and search and rescue 30–120 minutes – Orienteering practice 120–180 minutes – Survival skills discussion and shelter building 180–225 minutes – Medical kits Instructors: 1 Instructional Materials Compasses (5+) USGS Topographic Map of park Water purification devices Fire-starting devices Twine/wire for trapping	Hands-on assessment during final expedition scenarios and final examination
Session 4: Aquatic Medicine Duration: 190 minutes	Lecture on marine envenoma-tion Lecture on dive physics Lecture on decompression illness See supplement for full description of session	-Marine envenomation symptoms and management: including cnidarians (box jelly and irukandji), mollusk (conotoxin), stingray, puffer fish -Boyle’s law, Dalton’s law, and Henry’s law -Diagnosis and treatment of decompression illness, air gas embolism: hyperbarics	Recognize various marine envenomations and their treatment - including cnidarian, mollusks, and stingray Discuss the use of dive tables in prevention and hyperbarics in the treatment of decompresssion illness	M4	0–90 minutes – Marine envenomation (see Marine Envenomation PowerPoint) 90–105 minutes – Break 105–145 minutes – Physics of diving and prevention of illness (see Dive Physics PowerPoint) 145–160 – Break 160–190 – Decompression illness (see Decompression Illness PowerPoint) Instructors: 1 Instructional Materials Projected presentation materials	Hands-on assessment during final expedition scenarios and final examination
Session 5: Hiking Emergencies Duration: 220 minutes	Hands-on scenarios during hike Post-scenario debriefs See supplement for full description of session	-Diagnosis and treatment of North American crotalid and elapid envenomation -Diagnosis and treatment of arthropod envenomation: including latrodectus, hymenoptera, loxosceles -Diagnosis and management of amanita phalloides and false morel ingestions -Diagnosis and management of opiate, sympathomimetic, hallucinogenic plant toxidromes	Recognize arthropod envenomations and their treatment Recognize elapid and crotalid envenomation pathophysiolog y and manage envenomation with, CroFab discussion Identify and manage plant toxidromes	M4	0–30 minutes – Introduction 30–75 minutes – Crotalid envenomation scenario 75–95 minutes – Hike 95–140 minutes – Datura stramonium scenario 140–160 minutes – Hike 160–200 minutes – Amanita phalloides scenario 200–220 minutes – Projected Presentation (See Toxic Plants PowerPoint) Instructors: 1 Instructional Materials Projected presentation materials (projector and screen) Photographs of crotalus horridus, amanita phalloides, and datura stramonium	Hands-on assessment during final expedition scenarios and final examination
Session 6: High Angle Rescue and Safety Duration: 240 minutes	Lecture on search and rescue strategy Discussion of high-angle rescue Hands-on practice See supplement for full description of session	-Search and rescue strategy -Knot tying -High-angle rescue principles	Apply basic high-angle techniques for personal safety and patient rescue in trauma scenario Employ basic search and rescue strategy and organizational structure	M4	0–60 minutes – Introduction to search and rescue strategy 60–120 minutes – High-angle rescue 120–240 minutes – High-angle rescue scenario Instructors: 1 + Search and rescue instructors Instructional Materials Provided by local search and rescue team for rope rescue scenario	Hands-on assessment during final expedition scenarios and final examination
Session 7: Desert Medicine and Tick-Borne Diseases Duration: 220 minutes	Hands-on scenarios during hike Post-scenario debriefs See supplement for full description of session	-Pathophysiology of heat illness -Diagnosis and management of hyperthermia, heat exhaustion, and heat stroke -Triage and treatment of lightning strike -Diagnosis and treatment of Lyme disease and Rocky Mountain Spotted Fever	Evaluate and manage initial phase of heat illness, emphasizing importance of rapid cooling Manage lightning victims Assess patients with tick-related illness	M4	0–30 minutes – Introduction 30–75 minutes – Heat stroke scenario 75–95 minutes – Hike 95–140 minutes – Lightning strike scenario 140–160 minutes – Hike 160–200 minutes – Borrelia burgdorferi scenario 200–220 minutes – Conclusion	Hands-on assessment during final expedition scenarios and final examination
Session 8: Extensions of Austere Medicine Duration: 210 minutes If physicians with experience and expertise are not available for this content, the course instructor may substitute other activities for this day.	Lecture on International medicine Lecture on Disaster medicine Lecture on Event medicine Student presentation of final expedition logistics See supplement for full description of session	-Application of resource-constrained medical principles beyond the wilderness	Discuss extensions of austere medicine, including disaster response, international medicine, and remote event medicine	M4	0–45 minutes – International medicine 45–60 minutes – Break 60–105 minutes – Disaster medicine 105–120 minutes – Break 120–165 minutes – Event medicine 165–180 minutes – Break 180–210 minutes – Expedition Operations Instructors: 4 Instructional Materials Projected presentation materials	Final examination
Session 9: Course Expedition Duration: 36 hours	Hands-on scenarios during hike Post-scenario debriefs See supplement for full description of session	-Scenario-based assessment of student performance	Plan expedition and manage wilderness medicine scenarios Teach other learners about a plant of toxicologic significance	M4	24–36 hours – Overnight backpacking punctuated by student-led scenarios Instructors: 2 Instructional Materials To be determined by students Minimum: compass, USGS map, medical kit, sleeping bag, tent, food/water, weather appropriate clothing	Hands-on assessment during final expedition scenarios and final examination

### Appendix B Syllabus

Welcome to the Wilderness Medicine Elective!

Over the next two weeks you will be exposed to the breadth of wilderness medicine. In addition to discrete medical conditions, wilderness medicine involves the practice of medicine in unfamiliar, non-clinical environments, requiring knowledge of core life support principles, adaptability, and an awareness of the non-medical factors affecting the patient’s care. In the course, you will learn how to initiate care for many conditions in a remote setting, and we also hope you gain an appreciation of the non-medical skills that are essential to providing care in austere environments - and the collaboration necessary to make it happen.

Sessions will be interactive and often outdoors. In order to make them as interactive as possible, each day is accompanied by pre-reading in *Auerbach’s Wilderness Medicine*, 7th Edition (list if available through institution’s library). Having an understanding of the principles in the text will allow you to fully engage in scenarios and discussion. This textbook is extensive, and we have selected discrete sections for reading; yet we encourage you to read further as your interest is peaked. Most of our sessions will be held outside, so please dress and plan accordingly. You will be assigned several case scenarios to act and then debrief the group during our daily sessions. You will act as the content expert for debrief and discussion; please plan accordingly.

In addition to the daily sessions and pre-reading, the course also includes a final presentation on a plant of toxicologic significance of your choosing. The presentation will be a 10-minute talk covering medicinal history, mechanism of toxicity, and toxicologic management. This will be delivered during the final expedition so no electronic equipment will be available.

#### Course Objectives

1. Apply fundamental concepts of practicing medicine in austere conditions as demonstrated by successful patient care in final course expedition.
2. Identify and initiate treatment for common wilderness medicine conditions as demonstrated by successful patient care in final course expedition and obtaining 75% or greater on the final written examination.
3. Discuss and utilize non-medical aspects of providing care in austere environments as demonstrated by successful coordination and completion of the final course expedition and completion of two essays on the final examination.

#### Student Evaluation

This course is Pass/Fail, although the evaluation format can be adapted for a grade for a particular institution. Your grade will be comprised of three equal parts: participation, a final presentation, and a final written examination. The presentation is described above and will be discussed the first day of the course. The final examination will be distributed mid-way through the second week of the course and is due by the Sunday following the final expedition. Equally important will be your active engagement in the daily sessions and scenarios.

#### Course Evaluation

Just like providing care in an austere setting, this course is dynamic, continually evolving to better meet the course objectives. Your feedback is essential in making these changes. After the course is complete, we will request feedback. This will not impact your course grade.

### Appendix C Trailside Template Explanation

*The Trailside Talk & Sleeper Cases provide an opportunity for the resident or student to act as both the* ***victim and the educator*** *of their assigned topic. We feel this provides an opportunity for students to be more personally invested in their own education. The case is meant to be interactive with the other students providing care. The “victim” can offer gentle nudges as needed to keep the group on task. At completion of the scenario, the victim will transition to the “educator” and educate the group on the topic. It is the expectation that the students will have pre-read the material; thus these cases should act as a review and summary, helping to re-affirm the information, leading to mastery of the material.*

*As both participants and instructors of wilderness medicine experiences in the past, we understand that many of these courses can be a bit heavy on PowerPoint presentations, requiring its participants to spend a large amount of time indoors, learning about the medicine of the outdoors. Since most of our learning is provided trailside, lakeside, or mountaintop, we found our learner experience was one of increased satisfaction when compared to typical classroom-based education.*

*We have included the “Trailside Talk/Sleeper – Template” below. We found having a general skeleton of the cases to be beneficial in several ways. Initially, it allowed course instructors the opportunity to talk through the objectives and expectations of the “Trailside Talk” without spoiling any potential cases. We were also able to use this template to review key portions of our trailside assessment tools.*

*More importantly, we believe including this template may provide an easy way for programs that utilize these materials to make changes and take creative liberties as they see fit. While we have worked to create an easy to use course, we understand different programs may want to adjust things. For example, we have included a crotalid envenomation case, but if a program in the Southwest wanted to create a Gila monster envenomation case as well, they could do so with ease, utilizing the template provided.*

*You may send the below template to the students at the beginning of the course for their review and clarity of expectations. Consider removing the discussion explanations at the end of each case before sending to the students since synthesis and discussion are parts of their educational experience.*

#### Trailside Talk/Sleeper – Template

##### Objectives

1. You have been assigned the attached case scenario. At a random time along our outdoor experience, you will become the “victim,” requiring the rest of our group to assess and provide treatment. Moulage and props are encouraged. Keep your case a secret.
2. Upon completion of the case, you will assess the group’s management of your injury/illness. What did they do well? What could they improve upon?
3. Lead a group discussion on your topic. You are the expert. All trailside topics will have been covered in the pre-reading, but it’s your job to review and summarize the material including presentation, diagnosis, and treatment. You can use printouts or your smart device if images are necessary. Just remember, the more you bring, the more you will have to carry in your pack.
4. You will have 30 minutes to complete you case and discussion. On average, the case scenario typically lasts about 10 minutes, with debriefing and discussion filling the remainder.

**Background:**

*In this area, we describe the context of the scenario, giving some background information about location and events.*

“*You are hiking at Mt. Rainier when one of your classmates begins to complain of a headache and nausea. She looks clearly uncomfortable, clutching her head and begins vomiting. She thinks she just has a migraine. You wonder, is that all this is?”*

**Case:**

*In this area, we give a “one-liner” describing the case.*

*“Your friend develops headache, nausea and vomiting as you ascend the mountain.”*

##### Patient Assessment

- Scene survey
  ○ *Is the scene safe for rescuer to enter?*
    ▪ *Fire, water, snow, cliffside – priority is to not become the second victim*
  ○ *How many victims?*
  ○ ^****^ *Learner must verbalize consideration of Scene Safety with every scenario.*
- Primary Survey:
  ○ AVPU:
    ▪ *Initial Assessment focusing on level of responsiveness.*
      - Alert
      - Verbal
      - Painful
      - Unresponsive
  ○ MARCH:
    ▪ *The ABCDE of Wilderness Medicine. Given lack of blood products in austere locations, we focus on hemorrhage control as a priority*.
      - M - Massive Hemorrhage
      - A – Airway With C-Spine
      - R – Respiration
      - C – Circulation
      - H – Hypothermia/Hyperthermia
        *and*
        Hike or Helicopter – *How is the patient going to evacuate?*
- Secondary Survey:
  ○ S – Signs & symptoms
  ○ A – Allergies
  ○ M – Medications or medical alert tags
  ○ P – Past medical & surgical history
  ○ L – Last Meal (how long ago?)
  ○ E – Events (what led up to the incident?)
- Physical Exam:
  ○ General:
  ○ HEENT:
    ▪ Head:
    ▪ Eyes:
    ▪ Ears:
    ▪ Nose:
    ▪ Throat (more accurately oropharynx in practice):
  ○ Neck:
  ○ Chest:
  ○ Abdomen/Pelvis:
  ○ Extremities:
  ○ Neuro:
  ○ Skin:
  ^****^ *The cases will have some key discussion points. These are meant to provide some guidance for the leader of this trailside case in their preparation, but are not all-encompassing. We expect the student or resident to be able to discuss the content of their case well beyond the brief discussion points we have listed.*

### Appendix D Session 1: Introduction to Wilderness Medicine and Wilderness Trauma

The primary goal of this session is to introduce students to the core principles of wilderness medicine. The session will be structured around Kolb’s theory of experiential learning in order to improve assimilation of the information presented. Using Kolb’s stages, the session will proceed as follows: 1. a concrete experience, 2. reflective observation, 3. abstract conceptualization, and 4. active experimentation. This session will be held outdoors at a local park. Students will arrive to be confronted with a rescue scenario: a person fallen off a platform in the woods with obvious fractures who requires extrication. They will respond to this scenario as a group with faculty guidance as needed. After this scenario, the course faculty will facilitate a discussion, eliciting the students’ response to the case and any insights into care in the wilderness setting. This discussion will progress and encompass both stage 2 and stage 3. The final part of the session will present the students with various transportation and orthopedic stabilization scenarios with the goal of students experimenting with prior discussed principles to adapt to new situations. In addition to abstract concepts of care in the wilderness environment, concrete skills needed for the case will be taught, including methods of patient transportation and stabilization of orthopedic injuries.

**Session 1 Objectives:** After participation, the learner should be able to:

1. Discuss core wilderness medicine principles and applicability
2. Employ various methods of resource-constrained patient transportation
3. Perform treatment and stabilization of common orthopedic injuries

**Instructional Materials**

1. Multiple lengths of rope, up to 50 ft.
2. An overnight backpack
3. An environment with debris - sticks for immobilization
4. Hiking poles, a bandana
5. An EMS backboard with C-collar

**Pre-Reading**

None[Table t3-jetem-5-1-c1]

**Table t3-jetem-5-1-c1:**

Time (min)	Segment	Description
0–60	Patient scenario	Students will respond to a person found in the woods, fallen off a platform with a tibial and humeral fracture.
60–90	Course introduction	Welcome to the course and review the objectives and course materials.
90–150	Scenario debrief	Facilitated discussion of the experience, first focusing on student reflection and then formulating core concepts of wilderness medicine.
150–210	Improvisation scenarios	Students will be presented with various orthopedic injuries and will have to devise and implement immobilization and transportation methods.
210–230	Expedition introduction	Students will be briefed on the scenario and requirements for the final course expedition.

Below is the information to communicate to the students assigned to each trailside scenario. This same information will be used for all future scenarios in the course as well.

#### Trailside Talks – Objectives

1. You have been assigned the attached Case Scenario. At a random time along our outdoor experience, you will become the “victim,” requiring the rest of our group to assess and provide treatment. Moulage and props are encouraged. Keep your case a secret.
2. Upon completion of the case, you will assess the group’s management of your injury/illness. What did they do well? What could they improve upon?
3. Lead a group discussion on your topic. You are the expert. All Trailside Talks topics will have been covered in the pre-reading, but it’s your job to review and summarize the material including presentation, diagnosis, and treatment. You can use printouts or your smart device if images are necessary. Just remember, the more you bring, the more you will have to carry in your pack.
4. You will have 30 minutes to complete your case and discussion. On average, the Case scenario typically last about 10 minutes, with debriefing and discussion filling the remainder.

#### Trailside Talks – Trauma

Background:

You are hiking in the mountains with some fellow med students to relieve some “Pre-Match Anxiety.” You friend runs ahead to relieve himself in the woods when you hear a crash and a scream.

Case:

Your friend has fallen from the side of the trail, about 6 feet down. He’s holding his right shoulder.

- Primary Survey:
  ○ “AVPU”: Alert and oriented
    ▪ *AVPU: Initial assessment focusing on level of responsiveness*
      - *A - Alert*
      - *V- Verbal*
      - *P - Painful stimulus*
      - *U - Unresponsive*
  ○ “MARCH”:
    ▪ M – No evidence of massive hemorrhage
    ▪ A – Airway intact
    ▪ R – Tachypneic, but no distress
    ▪ C – Pulses intact
    ▪ H – No evidence of hyper/hypothermia
      - Hike vs Helicopter – Evacuation
      - *MARCH - The ABCs of wilderness medicine. Due to paucity of blood products in austere locations, hemorrhage control is a priority.*
        ○ *M - Massive hemorrhage*
        ○ *A – Airway with c-spine*
        ○ *R – Respirations*
        ○ *C – Circulation*
        ○ *H – Hypothermia/Hyperthermia*
          *Hike or Helicopter – How is the patient going to evacuate?*
- Secondary Survey (SAMPLE):
  ○ S – Complains of right shoulder pain and deformity
  ○ A – None
  ○ M – None
  ○ P – Previous shoulder dislocations/no surgical history
  ○ L – One hour ago
  ○ E – “I was just walking down this hill to use the bathroom behind that tree…I slipped…My shoulder is killing me. I think I dislocated it again.”
    ▪ *SAMPLE –A simple mnemonic useful to assess all components of the secondary survey*
      - *S – Signs & Symptoms*
      - *A – Allergies*
      - *M – Medications*
      - *P – Past medical and surgical history*
      - *L – Last meal (when was it)*
      - *E – Events (what led up to the incident?)*
- Physical Exam:
  ○ General: Awake, alert and oriented. Shaken, but cooperative and with it
  ○ Head, ears, eyes, nose and throat (HEENT):
    ▪ Head: contusion to right forehead
    ▪ Eyes: open, midline, ocular movements intact, pupils equal and responsive
    ▪ Ears: external normal
    ▪ Nose: midline, no septal hematoma
    ▪ Oropharynx: moist mucosa, no dental injuries or malocclusion
  ○ Neck:
    ▪ No crepitus, no palpable hematoma, positive diffuse cervical spine tenderness
  ○ Chest:
    ▪ No external deformity, no crepitus
    ▪ Cardiovascular: tachycardic, pulses bounding
      - Except in right arm – unable to palpate a radial pulse
    ▪ Respiratory: breath sounds equal bilateral
  ○ Abdomen/Pelvis:
    ▪ Non-tender in all four quadrants, non-distended
  ○ Extremities:
    ▪ Right shoulder injury – guarding, cannot move without pain
  ○ Neuro:
    ▪ Alert and oriented ×3
      - Diminished sensation in right hand
  ○ Skin:
    ▪ Moist skin, warm to touch

Critical Actions:

1. Scene Safety – must make sure that everything is safe before approaching the patient.
2. Maintain c-spine immobilization.
3. Shoulder reduction due to neurovascular compromise.

Discussion:

1. Discuss importance of scene safety.
2. Review use of AVPU and MARCH.
3. Discuss risk vs benefit consideration in wilderness and how it differs from in-hospital decisions.
4. Review and practice reduction and immobilization techniques, including c-spine.
5. Discuss and practice patient transportation methods.

Discussion:

1. Discuss importance of scene safety.
  - Is the scene safe for me to enter? Am I risking harm to myself and becoming the second victim? Will my attempt to enter the area cause possible risk to the patient?
  - Consider external hazards:
    - Physical hazards – rocks, ice, fire, trees, etc.
    - Environmental – heat, cold, altitude, lightning
    - Bystanders – mountain bikers approaching on the trail, climbers above, etc.
2. Review use of AVPU and MARCH
  - Primary Survey:
    - AVPU:
      1. *Initial Assessment focusing on level of responsiveness*
        - Alert
        - Verbal
        - Painful stimulus
        - Unresponsive
    - MARCH:
      1. *The ABCDE of wilderness medicine. Given lack of blood products in austere locations, we focus on hemorrhage control as a priority*.
        - M - Massive hemorrhage
        - A – Airway with c-spine
        - R – Respiration
        - C – Circulation
        - H – Hypothermia/Hyperthermia
          Hike or Helicopter – *How is the patient going to evacuate?*
  - Secondary Survey (SAMPLE):
    - S – Signs & Symptoms
    - A – Allergies
    - M – Medications/medical alert tags
    - P – Past medical/surgical history
    - L – Last meal
    - E – Events (what led up to the incident?)
3. Discuss risk vs benefit consideration in the wilderness and how it differs from in-hospital decisions.
  - Injuries occurring in the wild are often hours, and perhaps days, from a facility capable of providing a higher level of care. When assessing an injured patient in an austere location, decisions about evacuation must be regarded early, during the primary survey.
  - Distance from care, type of injury, and potential results of inaction all play an important role in your decision to manage a patient trailside. The patient in our case has sustained a shoulder dislocation. If it was this in isolation, we may choose to splint and abstain from reduction until we’ve reached a higher level of care where imaging, analgesia, and sedation are available. Unfortunately, our victim also has a pulseless extremity, forcing the caregivers to act urgently and definitively.
4. Review and practice reduction and immobilization techniques, including c-spine.
  - General Immobilization
    - Joint above and below the fracture should be immobilized
    - “Just right”
      1. Too tight – if the immobilization device is too tight, neurovascular sequelae may result
      2. Too loose – if the immobilization device is too loose, the fracture ends damage tissues/nerve/vessels due to excess movement
    - Materials
      1. Prefabricated splints/sticks/skis/paddles/cardboard/magazines
    - Remember, the patient’s own body can be used
      1. Upper extremity – shoulder adducted, elbow flexed to 90 degrees, forearm across abdomen
      2. Lower extremity – secure injured limb to non-injured limb
  - Reduction Tips:
    - Reduce early
      1. Muscle spasm is your enemy. It gets worse over time and is difficult to overcome. The earlier the reduction the easier it will be from this standpoint.
    - Traction-countertraction
      1. This is the most common method used
    - If can’t reduce –
      1. Splint in best position for patient comfort
      2. When to abandon reduction attempts
        - 3 strikes (if you fail three times in a row, stop trying to reduce), concern for associated fracture (for dislocations), patient refusal
5. Discuss and practice patient transportation methods
  - One-rescuer
    - Pick-a-back
    - Cradle lift
    - Single human crutch
    - Firefighter crawl
  - Multi-rescuer
    - Handed-seat carry
    - Fore-and-aft carry
    - Double human crutch
  - Improvised Stretcher
    - Rope litter
    - Coat/blanket stretcher

*There are multiple methods and variations of lift maneuvers. It would be impossible to describe them all, but there are multiple videos available online that provide excellent instructional information on how to perform these techniques. The instructor of this scenario should demonstrate an example of a one-rescuer, a multi-rescuer, and a stretcher/litter carry.*

### Appendix E Session 2: Mountain Emergencies

This primary goal of this session is to introduce students to common and emergent altitude- and cold-related diagnoses. This session will be held at a second local park at elevation and structured around the flipped classroom method. The afternoon prior to this session students will read targeted sections of *Auerbach’s Wilderness Medicine* textbook. The faculty member will lead a hike punctuated by several scenarios and educational debriefs. Periods of hiking between scenarios allow for internal reflection and non-structured discussion as well as a period of cognitive rest in preparation for the next scenario.

**Session 2 Objectives:** After participation, the learner should be able to:

1. Demonstrate initial diagnosis and management of acute mountain sickness (AMS), high-altitude pulmonary edema (HAPE), and high-altitude cerebral edema (HACE)
2. Illustrate management of frostbite and hypothermia

**Instructional Materials**

1. Backpack
2. Tarp
3. Emergency blanket

**Pre-Reading**

*Chapters 2 (High Altitude Syndromes), 7 (through Predisposing Factors)* [Table t4-jetem-5-1-c1]

**Table t4-jetem-5-1-c1:**

Time (min)	Segment	Description
0–30	Introduction	Meet at park, review hike logistics, start hike.
30–75	HACE scenario	Students confronted with scenario of high-altitude cerebral edema. Educational discussion after to include AMS.
75–95	Hike	Time to allow for mental reset and internal rehearsal of information.
95–140	HAPE scenario	Students confronted with scenario of high-altitude pulmonary edema. Educational discussion after.
140–160	Hike	Time to allow for mental reset and internal rehearsal of information.
160–200	Hypothermia scenario	Students confronted with scenario of hypothermia. Educational discussion afterwards to also include frostbite.
200–220	Finish Hike/Conclusion	Time to allow for mental reset and internal rehearsal of information.

#### Trailside Talks – High-Altitude Cerebral Edema

Background:

You have just finished 4^th^ year and decided to take a long weekend away with your fellow students to celebrate the end of med school. You convinced your spouse that it wasn’t a crazy idea to fly to Seattle and immediately drive to Mt. Rainier for a summit attempt.

You’ve been doing quite well for this arduous task with little sleep, but you noticed that one of your friends “doesn’t look so good.” He tells you it’s nothing, “just a little jet lag” and that he can keep going. You start to wonder if that’s all it is…

Case:

Your group has just reached 12,000 feet and your “jet-lagged” friend begins to slow and is now “walking funny.”

- Primary Survey:
  ○ AVPU: Awake but disoriented
  ○ MARCH:
    ▪ M – No evidence of massive hemorrhage
    ▪ A – Airway intact
    ▪ R – Tachypneic, but no distress
    ▪ C – Pulses intact
    ▪ H – No evidence of hyper/hypothermia
      - Hike vs helicopter
- Secondary Survey:
  ○ S – “I, I, I… just gotta get my banjo…It’s over there by that rhinoceros” (points to an outcrop of ice and begins walking unsteadily toward it)
  ○ A – Unknown
  ○ M – Unknown
  ○ P – Unknown
  ○ L – 4 hours ago (during a break with you)
  ○ E – Unable to elaborate
- Physical Exam:
  ○ General: Awake, disoriented
  ○ HEENT:
    ▪ Head: Atraumatic
    ▪ Eyes: Open, midline, ocular movements intact, pupils equal and reactive
    ▪ Ears: External normal
    ▪ Nose: Midline, no septal hematoma
    ▪ Oropharynx: Dry, no dental injuries or malocclusion
  ○ Neck:
    ▪ No crepitus, no palpable hematoma, no palpable step-off
  ○ Chest:
    ▪ No external deformity, no crepitus
    ▪ Cardiovascular: Tachycardic
    ▪ Respiratory: Breath sounds equal bilateral, hyperventilating
  ○ Abdomen/Pelvis:
    ▪ Non-tender in all four quadrants, non-distended
  ○ Extremities:
    ▪ Moves all extremities. No obvious trauma
  ○ Neuro: Altered, alert and oriented to self only, ataxic
  ○ Skin: Fully clothed for conditions, warm to touch

Critical Actions:

1. Recognition of high-altitude cerebral edema (HACE)
2. Immediate descent
3. Consideration of medication use if available – dexamethasone

Discussion:

1. Review pathophysiology of HACE
  - Spectrum of acute mountain sickness
2. Discuss symptoms/signs of HACE
  - May present with HAPE
3. Review management
  - Descent being primary
  - Medications
4. Prevention

Discussion

1. Review pathophysiology of HACE
  HACE and AMS are components of the same spectrum of disease. Cerebral edema is a factor in severe AMS and HACE, although less certain in mild AMS. The ‘tight fit’ hypothesis is often invoked, suggesting that individuals have different amounts of ‘extra’ space in their cranial vault and are more or less susceptible to the effects of cerebral edema depending on this. Vascular endothelial growth factor (VEGF), nitrous oxide (NO), and cytokines are all thought to play a role in the increased vascular permeability and loss of autoregulation of intracranial pressure. AMS typically occurs over 2,000 meters, while HACE is less common below 3,000 meters. A history of AMS or HAPE (high-altitude pulmonary edema) are more likely to develop HACE.
2. Discuss symptoms/signs of HACE
  AMS – headache, fatigue, nausea, emesis, anorexia, disturbed sleep
  HACE – ataxia, confusion, drowsiness, coma
    - May present with HAPE. If HAPE present, HACE can present more rapidly
3. Review management
  - AMS – Improves after 1 day with no further ascent or faster with descent (500–1000 meters). Severe AMS may require supplemental oxygen and descent. No further ascent until symptoms resolved. Treat symptoms with acetaminophen, ondansetron, and ibuprofen as needed. Acetazolamide is preferred over sleep aids for disturbed sleep. Dexamethasone may improve symptoms, but does not make acclimatization more rapid. Acetazolamide is favored for this reason.
  - HACE - The primary treatment of HACE is immediate descent at first signs/symptoms. Other medications and treatments should not delay descent. Dexamethasone is very helpful, given immediately and every 6 hours until descent of at least 1000 meters. Even if symptoms improve on dexamethasone, patient must descend. Remember, it does not speed up acclimatization. Supplemental oxygen as needed for oxygen saturation over 90% and portable hyperbaric chambers are helpful, but should not delay descent.
4. Prevention
  - Primary prevention for AMS is slow ascent. If moderate to high risk or rapid ascent, acetazolamide is preferred over dexamethasone. Dexamethasone is helpful for rapid ascent on short notice and short duration at altitude (rescue crews). Acetazolamide is often recommended at 125mg twice a day orally starting the day prior to ascent and stopping when maximum altitude is achieved. Ibuprofen and aspirin may have a role in preventing AMS.

#### Trailside Talks – High-Altitude Pulmonary Edema

Background:

You are hiking at Denali National Park when one of your classmates begins to complain of shortness of breath. You know there is a medical aid station about one kilometer ahead. Your classmate says “I can make it, I’ll get checked out up there. I’m just out of shape.” You wonder, is that all this is…

Case:

You friend is becoming increasingly short of breath as you ascend Denali towards a medical aid station.

- Primary Survey:
  ○ AVPU: Alert and oriented
  ○ MARCH:
    ▪ M – No evidence of massive hemorrhage
    ▪ A – Airway intact
    ▪ R – Hyperventilating, cough
    ▪ C – Pulses intact
    ▪ H – No evidence of hyper/hypothermia
      - Hike vs helicopter
- Secondary Survey:
  ○ S – “I’m really having trouble breathing. How far is the medic station?” (coughing profusely throughout)
  ○ A – None
  ○ M – None
  ○ P – No medical / surgical history
  ○ L – 5 hours ago
  ○ E – “I started feeling short of breath a few hours ago. Now I feel like I can’t breathe at all. I thought I could make it…I’m not so sure.”
- Physical Exam:
  ○ General: Breathing heavily, coughing
  ○ HEENT:
    ▪ Head: Atraumatic
    ▪ Eyes: Open, midline, ocular movements intact, pupils reactive and equal
    ▪ Ears: External normal
    ▪ Nose: Midline, no septal hematoma
    ▪ Oropharynx: Moist mucosa, no dental injuries or malocclusion,
      - Pink, frothy sputum
  ○ Neck:
    ▪ No crepitus, no palpable hematoma, no palpable step-off
  ○ Chest:
    ▪ No external deformity, no crepitus
    ▪ Cardiovascular: Tachycardic, pulses bounding
    ▪ Respiratory:
      - Tachypneic (respiratory rate 40s), crackles on auscultation
  ○ Abdomen/Pelvis:
    ▪ Non-tender in all four quadrants, non-distended
  ○ Extremities:
    ▪ Moves all extremities. No obvious trauma
  ○ Neuro: Alert and oriented ×3, sensation intact
  ○ Skin: Moist skin, warm to touch

Critical Actions:

1. Recognition of high-altitude pulmonary edema (HAPE)
2. Immediate descent
3. Consideration of medications – nifedipine.

Discussion:

1. Review pathophysiology of HAPE
2. Discuss signs/symptoms of HAPE
3. Discuss management of HAPE
  - Descent being primary
  - Medications
4. Review prevention strategies

Discussion:

1. Review pathophysiology of HAPE
  High-altitude pulmonary edema results from a maladaptive response to hypobaric hypoxia. There are many factors, such as uneven pulmonary vasoconstriction, increased sympathetic tone, and insufficient nitric oxide. The end result is damage to the blood-gas barrier and pulmonary edema leading to impaired oxygenation. HAPE is rare below 2,500 meters elevation.
2. Discuss signs/symptoms of HAPE
  Mild at onset - dry cough, dyspnea on exertion. Progresses to dyspnea at rest and cough productive of pink, frothy sputum or overt hemoptysis. Symptoms often start 2–4 days after arriving at elevation but can progress rapidly once they start. Progression is often noted to happen at night.
3. Discuss management of HAPE
  - Reducing pulmonary artery pressure is the goal, with several methods to do this. Unless experienced providers are present, descent is the best option. If resources are available and symptoms very mild, supplemental oxygen may be all that is needed. If available, oxygen should be given to every patient with suspected HAPE. Portable hyperbaric chambers are also beneficial.
  - Nifedipine can be used, although it is adjunctive. Tadalafil and sildenafil are effective in prevention of HAPE but not studied for treatment. Both diuretics and nitrates are not effective and should be avoided.
4. Review prevention strategies
  - As with most altitude illness, slow ascent is the best prevention.
  - Medication
    - No history of severe altitude illness or pulmonary hypertension, no prophylaxis needed
    - History of either of the above, nifedipine is the recommended medication. Start one day prior to ascent, take for 5 days at altitude or if descent earlier

#### Trailside Talks – Hypothermia

Background:

It is January and snowing, but you’ve grown tired of being stuck indoors. You’ve convinced a few of your fellow students to come along. You are parking at the park when you come upon an elderly, homeless appearing man lying under the picnic shelter. You wonder, how long has he been here…

Case:

You find an elderly man lying on a picnic table under the picnic shelter, vodka bottles are strewn about. He appears disoriented.

- Primary Survey:
  ○ AVPU: Awakens. Slurred speech.
  ○ MARCH:
    ▪ M – No evidence of massive hemorrhage
    ▪ A – Airway intact
    ▪ R – Respiration normal
    ▪ C – Pulses intact
    ▪ H – Skin cold to touch, not shivering
      - Hike vs Helicopter
- Secondary Survey:
  ○ S – Garbled, unintelligible speech
  ○ A – Unknown
  ○ M – Unknown
  ○ P – Unknown
  ○ L – Unknown
  ○ E – Unknown. You found him cold and confused on a picnic table
- Physical Exam:
  ○ General: Cold to touch, confused, incomprehensible speech
  ○ HEENT:
    ▪ Head: Atraumatic
    ▪ Eyes: Open, pupils dilated
    ▪ Ears: External normal
    ▪ Nose: Midline, no septal hematoma
    ▪ Oropharynx: Dry mucus membranes, poor dentition
  ○ Neck:
    ▪ No crepitus, no palpable hematoma, no palpable step-off
  ○ Chest:
    ▪ No external deformity, no crepitus
    ▪ Cardiovascular: Bradycardic, diminished pulses
    ▪ Respiratory: Lungs clear, bradypnea
  ○ Abdomen/Pelvis:
    ▪ Non-tender in all four quadrants, non-distended
  ○ Extremities:
    ▪ Moves all extremities slowly. No obvious trauma
  ○ Neuro: Disoriented, incomprehensible speech. Moves slowly, does not sit up.
  ○ Skin: Moist skin, cold to touch

Critical Actions:

1. Scene safety
2. Consideration of hypoglycemia and other etiologies
3. Recognition of hypothermia
4. Initiation of rewarming techniques appropriate for environment (review and practice these methods at this time)

Discussion:

1. Review pathophysiology of hypothermia and body regulation
2. Discuss epidemiology of hypothermia and predisposing factors
3. Discuss signs/symptoms of hypothermia
  - Mild
  - Moderate
  - Severe
  - Profound
4. Review treatment and prognosis
  - Must be warm and dead unless clear other cause of death
    - Discuss and practice out of hospital rewarming methods
    - Discuss in-hospital rewarming methods
  - Utility of CPR

Discussion

1. Review pathophysiology of hypothermia and body regulation
  - Body heat is lost through conduction, convection, radiation, and evaporation. Heat is generated through metabolism, so decreased metabolic states (illness, decreased activity, or substance-induced) and increased heat loss (from mechanisms above) can lead to hypothermia.
  - Body temperature is regulated by the hypothalamus. In response to cold, it may induce shivering, increase metabolism (thyroid, adrenal, and catecholamine mediated), and reduce heat loss through vasoconstriction of peripherally.
  - At temperatures less than 32ºC, cardiopulmonary slowing begins. Many cardiac dysrhythmias are possible.
2. Discuss epidemiology of hypothermia and predisposing factors
  - Increased heat loss: cold water immersion, prolonged exposure to cold weather/wind, improper clothing, blunted peripheral vasoconstriction response (alcohol)
  - Decreased heat production: medications that reduce physical activity or metabolism (opioids, benzodiazepines, sleep aids, alcohol), illness or infirmity that reduces ability to respond to cold appropriately (elderly, altered mental status from infection such as sepsis, hypoglycemia)
3. Discuss signs/symptoms of hypothermia (all temperatures core temperatures)
  Note that individual patients may not transition between these stages at the precise temperatures listed
  - Mild (32–35ºC) – shivering, normal to slightly impaired mental status, slow responses.
    - Cardiac: tachycardic
    - Pulmonary: tachypneic
    - Renal: cold diuresis – decreased peripheral blood flow increases flow to core organs, leading to increased diuresis
  - Moderate (28–32ºC) – decreased level of consciousness, may or may not shiver, may have paradoxical undressing, hyporeflexia
    - Cardiac: normal to decreased heart rate. Atrial fibrillation and junctional rhythms may occur.
    - Pulmonary: decreased respiratory rate
  - Severe (24–28ºC) – unconscious, no shivering, no reflexes, may lose pupillary light reflex
    - Cardiac: bradycardia, ventricular fibrillation, asystole
    - Pulmonary: pulmonary edema
  - Profound (<24ºC) – unconscious, may appear dead (but may recover)
4. Review treatment and prognosis
  - Must be warm (greater than 32–35ºC) and dead unless clear other cause of death, frozen chest, or potassium greater than 12 mEq/L
    - Discuss and practice out of hospital rewarming methods
      1. Take care not to move too much since this may induce dysrhythmias.
      2. Move to warm location if possible, blankets, removal of cold/wet clothes.
      3. ‘Hypothermia burrito wrap’ – tarp on ground, sleeping pads for insulation from ground on tarp, sleeping bags (with patient in one) on top of pads, then wrap with tarp. Can place warmed water bottles or another normothermic person in if needed.
    - Discuss in-hospital rewarming methods
      1. A reliable method of continuous core temperature measurement is vital. The closer to the heart, the better since that is the location of rewarming you care about. Esophageal probes are helpful for this. Labs are useful to look for end-organ damage or predisposing factors. Check a glucose.
      2. External rewarming: warm rooms, baby warmers (for pediatrics), heated blankets, warm air ventilated blankets, removal of cold/wet clothes.
      3. Internal rewarming: warmed intravenous fluids, intubation with warm humidified oxygen to start. Consider peritoneal, pleural, bladder, and bowel irrigation with warmed fluids.
      4. Extracorporeal membrane oxygenation (ECMO) for severe or refractory to warming cases.
  - Utility of CPR
    - Check for a central pulse for 1 minute before initiation.
    - No CPR if obvious other cause of death, incompressible frozen chest, or other signs of life.
    - Continuous CPR if possible, but intermittent CPR due to other considerations such as evacuation/terrain have been successful. Try not to pause greater than 5 minutes.
    - If cardiac monitor available, some experts recommend no CPR for severe hypothermia if organized rhythm is seen (even if no pulses felt) as this may cause further cardiac dysrhythmias.
    - Below 30ºC, consider one shock for shockable rhythms, but do not repeat until temperature above 30ºC.

### Appendix F Session 3: Navigation and Survival Skills

The primary goal of this session is to introduce students to non-medical skills necessary for caring for patients in austere settings. The session will be held at a third local park. The afternoon prior to this session students will read targeted sections of Auerbach’s Wilderness Medicine textbook. The first part of the session will be an interactive introduction to orienteering. Students will be taught the fundamentals of various map and compass navigation methods, highlighting particular methods often used in search and rescue. Students will then be split into four groups, each completing coordinate-based and bearing-based navigation courses. Afterwards, principles of survival for scenarios requiring prolonged field care will be discussed and various shelter methods will be practiced. Finally, a hands-on discussion and demonstration of common items in expedition medical kits will be had.

**Session 3 Objectives:** After participation, the learner should be able to:

1. Apply orienteering principles to navigate in wilderness setting
2. Explain wilderness principles of survival, including obtaining water, shelter, and signaling
3. List common expedition medical supplies

**Instructional Materials**

1. Compasses (5+)
2. USGS Topographic Map of park
3. Water purification devices
4. Fire-starting devices
5. Twine/wire for trapping

**Pre-Reading**

Chapter 59[Table t5-jetem-5-1-c1]

**Table t5-jetem-5-1-c1:**

Time (min)	Segment	Description
0–30	Introduction to Orienteering	Review of navigational principles and methods.
30–120	Orienteering Practice	Students split into smaller groups to complete two different orienteering courses, coordinate-and bearing-based.
120–180	Survival Skills	Discussion, demonstration, and practice of methods to obtain water, shelter, and fire as well as signal for help.
180–225	Medical Kits	Discussion and display of various medical kit supplies.

#### 1. Introduction to orienteering

During this segment, the teacher will facilitate a discussion on the navigation principles in the pre-reading, focusing on compass use and map interpretation. Review with the participants the parts of the compass (baseplate, direction of travel arrow, bezel, orienting arrow, and magnetic needle). Show them how to obtain a bearing on the compass. As an example, ask them all to face 120 degrees from north. To do this, they will line up 120 degrees on the bezel with the direction of travel arrow. They will then hold the compass flat in their hand and turn their entire body until the magnetic north needle is in the orienting arrow. You can select any other directions to practice. At any time in this, ask if anyone is familiar with a compass and can help teach. This is a chance to get students engaged with teaching. Be ready to correct any misinformation.

Then discuss the difference between true north (line from you to the north pole), magnetic north (line from you to the magnetic north pole), and declination (the difference, in degrees, between the two prior directions).

Discuss with them how bearings are used for short distances where precision is needed or travel in environments without many other features that help with navigation (such as flat fields or the ocean).

Following this, have examples of topographic maps. Discuss with the students what contour lines are and how they build a picture of the topography around you. Also review common map symbols (water is blue, roads/trails as solid and intermittent black lines, etc). Discuss how the scale of a map and contour interval impacts the interpretation of the map.

Next review with the students Universal Transverse Mercator system (UTM). This will likely involve a review of longitude and latitude. UTM is a system for dividing maps into squares, making identification of precise points possible. Using a map with UTM coordinates of the area you are, give examples of locations and their UTM coordinates. In the United States, the United States Geological Survey (USGS) has good maps for this use. Then give the students coordinates and have them locate them on the map.

#### 2. Orienteering Practice

During this segment, split the students into groups of 2–4. The smaller the group, the better.

- Half of the groups will start on a bearing-based course. To set this course up ahead of time, select a starting point. Then find a point of interest you can see (try to stay within 50–100 yards, although this can vary based upon the terrain you are in); then determine the bearing to that point yourself. Measure the distance you walk from the start to that point. This becomes the directions for the students (for example, 120 degrees for 200 feet). Then repeat the steps for the next point, starting from the first point of interest you selected. These points of interest can be trees, stumps, rocks, anything you desire. The beginning of a course could look something like this:
  1. 120 degrees, 200 feet
  2. 80 degrees, 40 feet
  3. 350 degrees, 85 feet
  4. 200 degrees, 60 feet
  5. And so on
  Try to have 10–12 points. This gives the students plenty of time to practice bearings. Students will need to determine distance. Measure out 50 feet. Have the students walk this, counting their paces. They can then determine how many feet they cover with each pace. This method of distance measurement is called pace counting and is often used for short distances in indistinct areas.
  If desired, you could hide cards at each point with wilderness medicine questions they have to answer.
- The other half of the class will start on a coordinate-based course. For this course, select 5–6 points on the map you want the students to visit. Try to keep these on trails or easily identifiable areas. For one course the authors do, the students have coordinates on several trails around a small lake. This is introductory, so to avoid someone getting lost, do not send the students into the woods without trails. Once you select your locations that create a loop, determine the coordinates. You then provide these coordinates to the students and let them plot them on a map. They will likely need a plotting tool for this (or show them how to divide the boxes into grids themselves). Verify the accuracy of their plotting on the map before they start the course. Have half of the students start the course in one direction and the other half in the other direction. As a check of accuracy, we have the students take a picture of a group member at each location.

#### 3. Survival Skills

For this segment, lead a discussion of the survival principles from the pre-reading. Focus on the importance of water, shelter, fire, and signaling. Ask the students for various ways to obtain and purify water. If you have the supplies, have examples with you. Sterilization methods discussed are solar stills, ultraviolet light, iodine tablets, and water filters. Share the items you’ve brought and review the advantages and disadvantages of each.

For shelters, review debris huts, caves, and snow huts. If your location is suitable, have the students build a debris hut. If you are in a cold climate with snow, dig a snow hut.

Review fire start techniques and equipment. This can include waterproof matches, lighter, and spark-producing products. If you have a suitable and safe location (our course has shelters with fireplaces), let the students practice fire starting. Be very careful that this is done in a safe manner, in a location safe for fires, and with approval from the park.

Finally, review methods of signaling. This can include radios, satellite/GPS communication devices, flare, fires, mirror use, and arrangement of natural objects into unnatural shapes or words.

If time is available and you have the knowledge, review basic trapping techniques for food. Discourage eating plants unless identification is extremely clear.

#### 4. Medical Kits

Spend time on this segment reviewing items that can be carried in medical kits. It is often helpful to have students create a list based upon common injuries and illnesses. Discuss how limitations of space and weight impact decisions on what to include, as well as the need to tailor the kit for each location and environment. If you have the equipment, bring examples.

### Appendix G Session 4: Aquatic Medicine

The primary goal of this session is to introduce students to marine envenomations and decompression illness. The session will be held in a classroom setting. The afternoon prior to this session students will read targeted sections of *Auerbach’s Wilderness Medicine* textbook. The initial discussion will review common and distinct marine creatures of significance. Following this will be a discussion of decompression illness, including decompression sickness and arterial gas embolism. Preventative measures, including use of dive tables, will be highlighted. Hyperbarics as a potential treatment modality will be reviewed.

**Session 4 Objectives:** After participation, the learner should be able to:

1. Recognize various marine envenomations and their treatment - including cnidarian, mollusks, and stingray
2. Discuss the use of hyperbarics in the treatment of decompression illness

**Instructional Materials**

1. Projected presentation materials

**Pre-Reading**

Chapters 71 (Diving Physics through Arterial Gas Embolism, Decompression Sickness), 74 (Phylum Cnidaria)[Table t6-jetem-5-1-c1]

**Table t6-jetem-5-1-c1:**

Time (min)	Segment	Description
0–90	Marine envenomation	Interactive lecture and discussion of marine envenomations (Appendix P).
90–105	Break	
105–145	Physics of diving and prevention of illness	Review physics of pressurized gas and related safe diving practices (Appendix Q).
145–160	Break	
160–190	Decompression illness	Interactive lecture and discussion of decompression sickness and arterial gas embolism (Appendix R).

### Appendix H Session 5: Hiking Emergencies

This primary goal of this session is to introduce students to the toxicologic aspects of wilderness medicine. This session will be held at a fourth local park and structured around the flipped classroom method. The afternoon prior to this session students will read targeted sections of *Auerbach’s Wilderness Medicine* textbook. The faculty member will lead a hike punctuated by several scenarios and educational debriefs. Periods of hiking between scenarios allow for internal reflection and non-structured discussion as well as a period of cognitive rest in preparation for the next scenario. The day will conclude with an outdoor projector presentation in order to show examples of plant and animal species not located in the park.

**Session 5 Objectives:** After participation, the learner should be able to:

1. Recognize arthropod envenomations and their treatment
2. Recognize elapid and crotalid envenomation pathophysiology and manage envenomation
3. Identify and manage common plant toxidromes

**Instructional Materials**

1. Projected presentation materials (projector and screen)
2. Photographs of *C. horridus horridus*, *Amanita phalloides*, and *Datura stramonium*

**Pre-Reading**

*Chapters 35 (Venomous Snakes: Scope through Management)* [Table t7-jetem-5-1-c1]

**Table t7-jetem-5-1-c1:**

Time (min)	Segment	Description
0–30	Introduction	Meet at park, review hike logistics, start hike.
30–75	*Crotalus horridus horridus* envenomation scenario	Students confronted with scenario of timber rattlesnake envenomation. Educational discussion afterwards.
75–95	Hike	Time to allow for mental reset and internal rehearsal of information.
95–140	*Datura stramonium* ingestion scenario	Students confronted with scenario of jimson weed ingestion. Educational discussion afterwards.
140–160	Hike	Time to allow for mental reset and internal rehearsal of information.
160–200	*Amanita phalloides* ingestion scenario	Students confronted with scenario of amanita phalloides ingestion. Educational discussion after.
200–220	Projected presentation	Review of other plants of toxicologic significance, highlighting common toxidromes (Appendix S).

#### Trailside Talks – Crotalid Envenomation

Background:

Hiking with friends, you are bitten by a snake that was on the trail. You will be bitten on the ankle/foot and develop severe pain and swelling that limits your ability to ambulate. (Moulage under sock: if you get a chance before the hike, color your ankle as if it is bruised with two bite marks; keep this under your sock until your case.) Try to kill the snake in your frustration. If you have a cheap fake snake, bring it as a prop.

Case:

“Ow!! A snake just bit me!”

- Primary Survey:
  ○ AVPU: Awake and alert
  ○ MARCH:
    ▪ M – No evidence of massive hemorrhage
    ▪ A – Airway intact
    ▪ R – No respiratory distress
    ▪ C – Pulses intact, tachycardic
    ▪ H – No hypo/hyperthermia
      - Hike vs helicopter
- Secondary Survey:
  ○ S – Pain in the foot at site of bite, sharp in quality
  ○ A – None: if asked, you have not received Crofab in the past
  ○ M – None
  ○ P – None
  ○ L – 3 hours ago
  ○ E – Hiking with friends, you accidentally stepped on a snake. See above background.
- Physical Exam:
  ○ General: Awake, alert, agitated
  ○ HEENT:
    ▪ Head: atraumatic
    ▪ Eyes: open, extraocular movements intact
    ▪ Ears: external normal
    ▪ Nose: nontender, patent
    ▪ Oropharynx: normal
  ○ Neck:
    ▪ Nontender, supple
  ○ Chest:
    ▪ Cardiovascular: tachycardic
    ▪ Respiratory: breathing comfortably
  ○ Abdomen/Pelvis:
    ▪ Non-tender in all four quadrants, non-distended
  ○ Extremities:
    ▪ Ankle/foot snake bite with ecchymosis. Swelling noted at site. Pulses intact.
  ○ Neuro: Distal sensation intact.

Branch Points:

- You must be stopped from trying to kill the snake; otherwise, you get another bite on the other leg and have to be carried out.
- Constriction band (not tourniquet) and splint should be placed. If not, you start to get lightheaded, nauseous, and pass out.

Critical Actions:

1. Removal from vicinity of snake
2. Transport for medical help

Discussion

1. Discuss pathophysiology of venoms and presentation
  - Elapid
  - Crotalid
2. Review Emergency Department (ED) evaluation and discuss treatment

This is found next to patient:[Fig f1-jetem-5-1-c1]

Image source: Ana_M. Untitled. In: Pixabay. https://pixabay.com/en/snake-rattlesnake-reptile-skin-751722/. CC0.

Discussion

1. Discuss pathophysiology of venoms and presentation
  - Elapid (neurotoxic)
  - Crotalid (hemotoxic, myotoxic, neurotoxic: varies by snake/region)
  Elapids
  - Coral snake: this is found in the Southeast of the United States (US) – Florida primarily
  - Envenomations are rare and comprise less than 1% of US snake bites
  - Distinct banding of snakes
    ○ “Red on black, venom lack, red on yellow, kill a fellow” is a common phrase used to remember the pattern, although it is specific for the Southeastern US.
  - Neurotoxic venom
    ○ Mild symptoms initially with rapid progression following
    ○ Impending respiratory failure signs and symptoms include the following:
      - Respiratory distress
      - Hypersalivation
      - Cyanosis
      - Trismus
      Neurologic dysfunction signs and symptoms include the following:
      - Altered mental status
      - Ptosis
      - Generalized weakness
      - Muscle fasciculations
      Cardiovascular collapse signs and symptoms include the following:
      - Hypotension
  - Must evacuate patients due to the potential for rapid progression and severe symptoms.
  - Outcomes:
    ○ Only one death in about 40 years (which coincides with introduction of anti-venom)
    ○ These patients may require prolonged intubation
    ○ Weakness from the envenomation may persist for months
  Crotalids
  - In the US, this group of pit vipers is responsible for majority of envenomations.
    ○ Though 25% are likely “dry” – without injection of venom
  - US crotalids include:
    ○ Multiple species of rattle snakes
    ○ Copperheads
    ○ Water moccasins
  - Territory
    ○ Found in essentially the entire contiguous US, with exception of Maine
  - Recognizable features
    ○ Triangle-shaped head
    ○ Heat sensing pits
    ○ Elliptical, cat-like eyes
  - Hemotoxic Venom
    ○ Uniform among crotalids, with exception of the Mojave Rattler (it is primarily neurotoxic).
  - Clinical Features of envenomation
    ○ Severe pain and edema at the bite location
      ▪ Can lead to compartment syndrome
    ○ Coagulopathy
    ○ Nausea and vomiting
    ○ Fasciculations, perioral numbness, paresthesias
    ○ Respiratory distress secondary to pulmonary edema, hypotension and shock are possible
  - Must evacuate all envenomations in the wild, as progression to limb- or life-threatening injury is possible.
2. Review Emergency Department evaluation and discuss treatment
  - ABCs (Airway, Breathing, Circulation)
    ○ These patients will often present with mild injury, and perhaps require no intervention in the ED.
    ○ That said, they can often present with clear evidence of an envenomation and a spectrum of potential sequelae. Being prepared for limb ischemia, coagulopathy, respiratory distress, systemic neurotoxicity and shock are of the utmost importance.
3. Local Injury
  ○ Pain, swelling, and hemorrhage are common.
  ○ Monitor for compartment syndrome development.
    ▪ Measure limb circumference above and below bite every 30 minutes.
4. Hematologic abnormalities
  ○ Labs – complete blood count, basic metabolic panel, coagulation factors, fibrinogen.
    ▪ Repeat after 4 hours or after each course of antivenom.
5. Antivenom
  - Crofab (Crotalidae Polivalent Immune Fab (FabAV))
    - Indications
      1. Progressive swelling
      2. Lab abnormalities
        - Platelets < 100,000
        - Fibrinogen < 100
      3. Systemic manifestations
        - Unstable vitals
        - Altered mental status
      4. Dosing (this should be in consultation with toxicologist or poison control center as guidelines vary slightly)
        - Initial dose – 6 vials
        - May repeat at 6, 12 and 18 hours
          - 2 vials per dose
        - Same dosing for adults and pediatric patients
    - Reactions
      1. Allergic reactions and anaphylaxis are possible. They occur in less than 10%, but can be severe
      2. Serum sickness is unlikely, but possible
  - Disposition
    - All patients getting antivenom are admitted
  - Other treatments
    - Suggest against fasciotomy for compartment syndrome – give Crofab instead
  - Elapid
    - US stockpile of antivenom expired in 2008. Expiration date has been extended yearly. New possibilities are being researched.

#### Trailside Talks – *Datura Stramonium* (jimson weed)

Background:

You were blowing off some steam after a rough week in college in Virginia, spending time with new friends. One of them suggested you try smoking something “natural.” He seemed really excited as he picked a plant on the side of the trail and then smoked it. You figured, why not, and gave it a try.

Case:

When found, you are sitting on the side of the trail, mumbling quietly to yourself. It seems your friends have hiked on and left you.

- Primary Survey:
  ○ AVPU: Awake but disoriented
  ○ MARCH:
    ▪ M – No evidence of massive hemorrhage
    ▪ A – Airway intact
    ▪ R – No respiratory distress
    ▪ C – Pulses intact, tachycardic
    ▪ H – Feel hot to the touch
      - Hike vs helicopter
- Secondary Survey:
  ○ S – Mumbling, moving slowly, laughing to self, unable to coherently give info
  ○ A – Unknown
  ○ M – Unknown
  ○ P – Unknown
  ○ L – Unknown
  ○ E – Unable to elaborate
- Physical Exam:
  ○ GEN: Awake, Disoriented
  ○ HEENT:
    ▪ Head: Atraumatic
    ▪ Eyes: Open, midline, ocular movements intact, mydriasis
    ▪ Ears: External normal
    ▪ Nose: Midline, no septal hematoma
    ▪ Oropharynx: normal
  ○ Neck:
    ▪ Nontender, supple
  ○ Chest:
    ▪ No external deformity, no crepitus
    ▪ Cardiovascular: Tachycardic
    ▪ Respiratory: Breathing comfortably
  ○ Abdomen/Pelvis:
    ▪ Non-tender in all four quadrants, non-distended
  ○ Extremities:
    ▪ Moves all extremities. No obvious trauma
  ○ Neuro: Altered. Mumbling, does not answer questions appropriately.
  ○ Skin: Erythematous, hot to touch, dry

Critical Actions:

1. Scene safety
2. Consideration of hypoglycemia – oral treatment
3. Recognition of anticholinergic toxidrome
4. Facilitate transport for evaluation

Discussion:

1. Review pathophysiology of jimson weed
2. Review signs and symptom
  - Anticholinergic toxidrome
3. Review treatment of anticholinergic toxidrome

This is found next to patient:[Fig f2-jetem-5-1-c1]

Image source: WikimediaImages. Untitled. In: Pixabay. https://pixabay.com/en/datura-stramonium-jimson-weed-844548/. CC0.

Discussion

1. Review pathophysiology of jimson weed
  - Tropane Alkaloids (toxin) – atropine, scopolamine, and hyoscine
    - Competitive inhibition of the post-synaptic antimuscarinic receptors, leading to classic anticholinergic toxidrome
2. Review signs and symptom
  - Anticholinergic toxidrome includes:
    - Hallucination
    - Hyperthermia
    - Flushed, dry skin and mucous membranes
    - Mydriasis
    - Tachycardia
    - Urinary retention
  - Classic mnemonic
    - Hot as a hare,
      Blind as a bat,
      Dry as a bone,
      Red as a beet,
      Mad as a hatter
  - Useful clinical sign
    - Check the axilla – in anticholinergic toxicity, this area will be dry. In the sympathomimetic toxidrome, this area will not be dry.
3. Review treatment of anticholinergic toxidrome
  - Supportive measures
    - Intravenous fluids, respiratory support, external cooling
    - Foley catherization for urinary retention if present
  - Agitation
    - Liberal use of benzodiazepines, monitoring closely for respiratory depression
    - Avoid haloperidol and phenothiazines since they will possibly enhance toxicity
  - Physostigmine
    - Cholinesterase inhibitor
    - Very narrow therapeutic window, especially with rapid administration
      1. Can lead to asystole, bradycardia and ventricular dysrhythmia
    - Most cases will never require this medication

#### Trailside Talks – Amanita Phalloides

Background:

You woke up this morning with vomiting and diarrhea. Last night you were out foraging for mushrooms, your favorite Fall activity. You are now tired but the vomiting has stopped. You ate some of what you found last night, but it seems you accidentally picked amanita phalloides. Do not tell the learners what you were doing or ate last night unless asked.

Case:

You are found sitting, feeling unwell, tired from all the vomiting.

- Primary Survey:
  ○ AVPU: Awake, alert
  ○ MARCH:
    ▪ M – No evidence of massive hemorrhage
    ▪ A – Airway intact
    ▪ R – No respiratory distress
    ▪ C – Pulses intact, tachycardic
    ▪ H – No evidence of hypo/hyperthermia
      - Hike vs helicopter
- Secondary Survey:
  ○ S – vomiting, diarrhea
  ○ A – None
  ○ M – Multivitamin
  ○ P – None
  ○ L – Night prior
  ○ E – As noted in background above
- Physical Exam:
  ○ General: Awake and oriented, tired
  ○ HEENT:
    ▪ Head: atraumatic
    ▪ Eyes: pupils normal
    ▪ Ears: external normal
    ▪ Nose: normal
    ▪ Oropharynx: dry mucosa
  ○ Neck:
    ▪ Nontender, supple
  ○ Chest:
    ▪ Cardiovascular: Tachycardic, 108
    ▪ Respiratory: No distress, breathing comfortably
  ○ Abdomen/Pelvis:
    ▪ Mild diffuse discomfort with palpation, non-focal, non-distended
  ○ Extremities:
    ▪ Moves all extremities. No obvious trauma
  ○ Neuro: Altered, normal neuro exam
  ○ Skin: No rash[Fig f3-jetem-5-1-c1]

Image source: skeeze. Untitled. In: Pixabay. https://pixabay.com/en/mushroom-fungus-death-cap-deadly-1063290/. CC0.

**Branch Point:**

- Learners should identify that this is delayed toxicity from mushroom ingestion and call for emergency medical services (EMS) to transport to hospital. Once they do this, you will start to seize.
- At this point, they can assume they are in ED. What would they want to check, what is on their differential? They should evaluate liver function tests, glucose, consult the poison control center.

Critical Actions:

1. Identify delayed mushroom toxicity
2. Evaluate glucose
3. Consider treatment in conjunction with poison control center

Discussion:

1. Review pathophysiology of *Amanita phalloides* ingestion
2. Discuss phases of toxicity and signs/symptoms
3. Discuss treatment and use of poison control center

Discussion

1. Review pathophysiology of *Amanita phalloides* ingestion
  - Alpha-amanitin
    - Inhibits RNA polymerase II
    - The liver is often the main organ impacted, although the kidneys are often involved as well. Overt symptoms are initially delayed and include emesis and diarrhea. Subsequent liver failure can lead to jaundice and death.
2. Discuss phases of toxicity and signs/symptoms
  - Latent
    - Patient is asymptomatic initially
  - Gastrointestinal
    - Nausea, vomiting, diarrhea, and abdominal cramping at 6–8 hours.
      Early gastrointestinal symptoms (within 3–4 hours of ingestion) can be a good indicator of non-lethal ingestion.
  - Convalescent
    - Resolution of gastrointestinal symptoms
  - Liver failure
    - Often within 1–3 days
      1. May require transplant
      2. Frequently fatal
3. Discuss treatment and use of poison control center
  - Supportive care
    - Hypoglycemia is a cause of death – monitor for it
  - Activated charcoal
    - May be of benefit within one hour of ingestion. Due to enterohepatic recirculation, repeat dosing may be beneficial.
  - Specific medications
    - N-acetylcysteine
      1. Dosing is similar to acetaminophen toxicity
    - Silibinin
      1. More commonly used in Europe for treatment. High-dose penicillin has similar mechanism
    - High dose penicillin
      1. Possible benefit in displacing amatoxin uptake by hepatocytes

### Appendix I Session 6: High Angle Rescue and Safety

This primary goal of this session is to introduce students to specialized non-medical skills necessary for caring for patients in austere settings. This session will be held at a fifth local park in conjunction with a local Search and Rescue Team (SAR). The afternoon prior to this session students will read targeted sections of *Auerbach’s Wilderness Medicine* textbook. The Search and Rescue team commonly performs prolonged rescue operations, often involving helicopter evacuation from remote locales involving high angle techniques. This team will conduct a review of their equipment, operational structure, and planning for high-angle rescue operations. An interactive patient rescue scenario will be conducted with student participation.

**Session 6 Objectives:** After participation, the learner should be able to:

1. Apply basic high-angle techniques for personal safety and patient rescue (*PLEASE NOTE: high angle rescues are complicated and dangerous and if a SAR team or other appropriately trained person is unavailable to teach this session should be skipped*)
2. Employ basic search and rescue strategy and organizational structure

**Instructional Materials**

1. Provided by local SAR Team

**Pre-Reading**

Chapter 56 (Planning for Wilderness Medicine Technical Rescue) and Chapter 55 (Search and Rescue Operations)[Table t8-jetem-5-1-c1]

**Table t8-jetem-5-1-c1:**

Time (min)	Segment	Description
0–60	Introduction	Introduction of SAR team and discussion of their purpose, frequency of activation, and search and rescue strategy.
60–120	High-angle equipment	Hands-on overview of their gear and its use.
120–240	High-angle rescue scenario	Students will conduct a rescue operation using high-angle rescue techniques and equipment, with facilitation by SAR team.

### Appendix J Session 7: Desert Medicine and Tick-Borne Diseases

This primary goal of this session is to introduce students to heat injury and tick-borne diseases. This session will be held at a sixth local park and structured around the flipped classroom method. The afternoon prior to this session students will read targeted sections of *Auerbach’s Wilderness Medicine* textbook. The faculty member will lead a hike punctuated by several scenarios and educational debriefs. Periods of hiking between scenarios allow for internal reflection and non-structured discussion as well as a period of cognitive rest in preparation for the next scenario.

NOTE: The final examination will be distributed to the students this afternoon via email.

**Session 7 Objectives:** After participation, the learner should be able to:

1. Evaluate and manage initial phase of heat illness
2. Manage lightning victims
3. Assess patient with tick-related illness

**Instructional Materials**

1. Photograph of Lyme disease target rash
2. Photograph of Lichtenberg figure

**Pre-Reading**

Chapters 42 (Tick-Borne *Borrelial* Diseases), 12 (Heat Stress and Thermoregulation)[Table t9-jetem-5-1-c1]

**Table t9-jetem-5-1-c1:**

Time (min)	Segment	Description
0–30	Introduction	Meet at park, review hike logistics, start hike.
30–75	Heat stroke scenario	Students confronted with scenario of heat stroke. Educational discussion afterwards.
75–95	Hike	Time to allow for mental reset and internal rehearsal of information.
95–140	Lightning strike scenario	Students confronted with pulseless lightning strike victim. Educational discussion afterwards.
140–160	Hike	Time to allow for mental reset and internal rehearsal of information.
160–200	*Borrelia burgdorferi* scenario	Students confronted with scenario of lyme disease. Educational discussion afterwards.
200–220	Finish Hike/Conclusion	Time to allow for mental reset and internal rehearsal of information.

#### Trailside Talks – Heat Stroke

Background:

While running up a trail in July, you become overly tired and exhausted. You are training for a marathon and have run the trail twice already that morning. You then progress to being confused. You will occasionally say the wrong words when talking: “I’m just so tired. I’ve been running up this car and where are my keys? I’ll just sit here and look for my…”

Case:

You run by the group, starting to get tired and off balance. Just after passing them, you sit down on the side of the trail with the above findings.

- Primary Survey:
  ○ AVPU: Awake but disoriented
  ○ MARCH:
    ▪ M – No evidence of massive hemorrhage
    ▪ A – Airway intact
    ▪ R – Slightly tachypneic
    ▪ C – Pulses intact, tachycardic
    ▪ H – Feel hot to the touch
      - Hike vs Helicopter
- Secondary Survey:
  ○ S – Talking, confusion, able to give some info
  ○ A – None
  ○ M – ‘over the counter cold medicine’ (when mental status is better, you find out it is loratadine/pseudoephedrine)
  ○ P – None
  ○ L – 2 hours ago
  ○ E – Training for marathon, running up the trail when you had a hard time walking
- Physical Exam:
  ○ General: Awake, oriented to person, and place, not year
  ○ Temperature, if able – 42C
  ○ HEENT:
    ▪ Head: Atraumatic
    ▪ Eyes: Open, midline, ocular movements intact, pupils normal
    ▪ Ears: External normal
    ▪ Nose: Normal
    ▪ Oropharynx: Dry mucosa
  ○ Neck:
    ▪ Nontender, supple
  ○ Chest:
    ▪ No external deformity
    ▪ Cardiovascular: Tachycardic, 120
    ▪ Respiratory: Tachypneic, rate in low 20s
  ○ Abdomen/Pelvis:
    ▪ Non-tender in all four quadrants, non-distended
  ○ Extremities:
    ▪ Moves all extremities. No obvious trauma
  ○ Neuro: Altered, not oriented to year, difficulty with situation
  ○ Skin: Erythematous, hot to touch, dry

Branch Point:

- A: Must be cooled and stopped from further activity. Insulating clothing should be removed (please remain decent!). Cooling methods may be misting, dunking in a cool stream/lake. Should prioritize rapid cooling.
- B: Consideration of hypoglycemia should be undertaken with possible oral correction.

Critical Actions:

1. Active cooling
  - Cold-water immersion or mist-fan
2. Oral hydration / food
  - If not given oral hydration/food with cooling, start becoming altered again
3. Refuse hospital evaluation initially
  - “Why do I need to go to the hospital? I’m feeling much better?”
  - If they let you stay – start to become altered again
4. Thermoregulation question:
  - Do you think that medicine I take may have contributed to this? Why?

Discussion:

1. Discuss mechanisms of heat regulation.
2. Discuss physiologic response to heat stress
3. Pathophysiology of heat-related injury
4. Clinical manifestations of heat injury
5. Management of heat illnesses

Discussion

1. Discuss mechanisms of heat regulation
  - Four Mechanisms:
    - Radiation
      1. Transfer of heat between the body and the environment
        - Electromagnetic waves
    - Convection
      1. Heat transfer between the body and moving medium
        - Gas or liquid (usually air or water)
        - Rate of heat transfer
          - Depends on how fast the air/water is moving and the temperature of the substance
          - Affected by temperature gradient, area exposed, and air movement
            1. Wind chill is a good example people are often familiar with
    - Conduction
      1. Transfer of heat between two objects in direct contact
        - Heating pad and ice packs are good examples
      2. This is surface area dependent. The larger the surface area in contact, the greater the effect.
      3. This usually has little effect in heat-related illnesses
        - Only 2% of heat loss is via conduction
        - An exception is prolonged lying on cold ground or water
      4. In the ED this can be a useful mechanism for treating hyperthermia
        - A cooling blanket is a good example
    - Evaporation
      1. Heat transfer in change of state from liquid to gas
        - Primary method of human heat regulation
          - This is done through sweating
          - 30% of cooling occurs this way at average temperatures
            1. It is dependent on wind velocity and vapor pressure
      2. Works best in dry environments and is less effective with increasing ambient humidity. When humidity approach 100%, the body is unable to dissipate heat via this mechanism
2. Discuss physiologic response to heat stress
  - Voluntary response
    - Seeking shade, drinking water, cooling self, resting
  - Involuntary response
    - Regulated by the hypothalamus
      1. Vasodilation
        - This leads to increased blood flow to the skin
        - Renal and splanchnic vasoconstriction shunts flow (heat) away from the core
        - This then leads to decreased peripheral vascular resistance, resulting in increased cardiac output and heart rate
        - The net effect is increased blood flow to the skin
      2. Increased Catecholamines
        - Activation of increased number of sweat glands
        - Active glands become “more active”
      3. Inhibition of Metabolic Heat Production
        - Decreased amount of heat the body has to manage
3. Pathophysiology of heat-related injury
  - Heat-related injury occurs when the above mechanisms are either saturated or ineffective. For example, evaporation may be ineffective due to increased humidity preventing sweat from evaporating. Or the production of heat by the body may be greater than the ability of these mechanisms to dissipate heat.
  - Once the body retains too much heat, damage occurs. This is due to decreased functionality of organs in heat, often from dehydration initially and ultimately from denaturing of proteins.
4. Clinical manifestations of heat injury
  - Heat Exhaustion
    - Symptoms
      1. Weakness, lightheadedness, fatigue
      2. Nausea, headache, and extreme thirst
    - Signs
      1. Tachycardia, tachypnea, diaphoresis
      2. Hyperthermia, but temperature less than 40 °C
      3. Key is that mental status is normal
  - Classic Heat Stroke
    - Triad of symptoms
      1. Core temperature greater than 40 °C
      2. Hot and dry skin
      3. Altered mental status and coma
    - Epidemiology
      1. Often during heat waves
      2. Extremes of age, sedentary, and poor are more vulnerable
      3. Medications such as diuretics, anticholinergics, neuroleptics, and antihypertensives can increase susceptibility
      4. Chronic illness, alcohol abuse, and mental illness can also increase susceptibility
  - Exertional Heat Stroke
    - Triad of symptoms
      1. Core temperature greater than 40 °C
      2. Altered mental status and coma
5. Management of Heat Illnesses
  - Remove from heat source and direct sunlight
    - ABCs
    - Mortality directly proportional to duration of hyperthermia
      1. Goal is to cool to 39 °C within 1 hour – even if this delays transport
  - Active cooling
    - Cold-water immersion
      1. Faster than evaporative cooling
      2. A downside is ability to monitor patient while immersed
    - Evaporative
      1. Make the victim wet with water
      2. Fan the patient, helping this water evaporate
  - Emergency department management
    - Cooling continues to goal of 39 °C if not reached prehospital
    - Rehydration
    - Evaluate and treat end-organ failure
  - Mortality
    - Exertional heat stroke: with rapid cooling, survival rates approach 90%–100%
    - Classic heat stroke survival is much lower, around 14%–63%

#### Session 7 - Lightning Injury (multi-student scenario)

Background:

You and a group of friends are out for a post-shift hike. You’re about 2 miles into a 5-mile loop when the skies start to darken in a very ominous way, and you realize the thunderstorm you had hoped to miss is coming. Three of your friends are immensely afraid of getting wet so they run ahead. About 3 minutes later you hear a deafening thunderclap and you realize lightning has just struck somewhere along the trail ahead.

Case:

You are one of three victims of the lighting strike.

**Victim #1:** Glasgow coma scale (GCS) 3. Unresponsive, clothes blown apart (but still on).

- Primary Survey:
  ○ AVPU: Unresponsive, apneic, pulseless
  ○ MARCH:
    ▪ M – No evidence of massive hemorrhage
    ▪ A – Apneic
    ▪ R – No spontaneous respiration
    ▪ C – Pulseless
    ▪ H – No evidence of hyper/hypothermia
      - Hike vs Helicopter
- Secondary Survey:
  ○ SAMPLE – ALL UNKNOWN
- Physical Exam:
  ○ General: Glasgow coma scale is 3, apneic, pulseless, clothing blown off
  ○ HEENT:
    ▪ Head: Atraumatic
    ▪ Eyes: Lids closed, pupils dilated, unresponsive
    ▪ Ears: External normal
    ▪ Nose: Midline, no septal hematoma
    ▪ Oropharynx: moist mucus membranes, no dental Injuries
    ○ Neck:
    ▪ No crepitus, no palpable hematoma, no palpable step-off
  ○ Chest:
    ▪ No external deformity, no crepitus
    ▪ Cardiovascular: No palpable pulse
    ▪ Respiratory: No spontaneous breaths
  ○ Abdomen/Pelvis:
    ▪ Non-tender in all four quadrants, non-distended
  ○ Extremities:
    ▪ No gross deformity, no palpable pulses
  ○ Neuro:
    ▪ GCS 3, pupils fixed and dilated
  ○ Skin:
    ▪ Lichtenberg figures (moulage or picture prop)

**Victim #2:** Awake, Sitting next to a tree. Holding her shoulder.

- Primary Survey:
  ○ AVPU: Alert and oriented
  ○ MARCH:
    ▪ M – No evidence of massive hemorrhage
    ▪ A – Airway intact
    ▪ R – Tachypneic, but no distress
    ▪ C – Pulses intact
    ▪ H – No evidence of hyper/hypothermia
      - Hike vs Helicopter
- Secondary Survey:
  ○ S – Complains of right shoulder pain and deformity
  ○ A – None
  ○ M – oral contraceptive pill
  ○ P – No medical/surgical history
  ○ L – One hour ago
  ○ E – “We were running back to car to beat the storm when it felt like an explosion went off. Everything was so loud and white. I landed over against this tree”.
- Physical Exam:
  ○ General: Awake, Alert and Oriented. Shaken, but cooperative and with it
  ○ HEENT:
    ▪ Head: Contusion to right forehead
    ▪ Eyes: Open, midline, ocular movements intact, pupils equal, reactive
    ▪ Ears: External normal
    ▪ Nose: Midline, no septal hematoma
    ▪ Oropharynx: Moist mucus membrane, no dental injuries or malocclusion
  ○ Neck:
    ▪ No crepitus, no palpable hematoma, positive diffuse c-spine tenderness
  ○ Chest:
    ▪ No external deformity, no crepitus
    ▪ Cardiovascular: Tachycardic, pulses bounding
    ▪ Respiratory: Breath sounds equal bilateral
  ○ Abdomen/Pelvis:
    ▪ Non-tender in all four quadrants, non-distended
  ○ Extremities:
    ▪ Right shoulder injury – holding her arm internally rotated and adducted, and exhibiting flattening of the anterior shoulder
  ○ Neuro: Alert and oriented ×3, moves all extremities (right arm limited due to pain), sensation intact
  ○ Skin: Moist skin, warm to touch

**Victim #3:** Losing his/her mind. No apparent injuries.

- Primary Survey:
  ○ AVPU: Alert and oriented
  ○ MARCH:
    ▪ M – No evidence of massive hemorrhage
    ▪ A – Airway intact
    ▪ R – Hyperventilating
    ▪ C – Pulses intact
    ▪ H – No evidence of hyper/hypothermia
      - Hike vs Helicopter
- Secondary Survey:
  ○ S – Rapidly pacing and yelling for help, no specific injury
  ○ A – None
  ○ M – None
  ○ P – No past medical or surgical history
  ○ L – One hour ago
  ○ E – “What just happened. You gotta help me!! Did we just get struck by lightning?!! You gotta help me!! Please don’t let me die…”
    (Continues rambling and distracting from other victims)
- Physical Exam:
  ○ General: Anxious, agitated,
  ○ HEENT:
    ▪ Head: Contusion to right forehead
    ▪ Eyes: Open, midline, ocular movements intact, pupils equal and reactive
    ▪ Ears: External normal
    ▪ Nose: Midline, no septal hematoma
    ▪ Oropharynx: Mucus membranes moist, no dental injuries or malocclusion
  ○ Neck:
    ▪ No crepitus, no palpable hematoma, no palpable step-off
  ○ Chest:
    ▪ No external deformity, no crepitus
    ▪ Cardiovascular: Tachycardic, pulses bounding
    ▪ Respiratory: Breath sounds equal bilateral, hyperventilating
  ○ Abdomen/Pelvis:
    ▪ Non-tender in all four quadrants, non-distended
  ○ Extremities:
    ▪ Moves all extremities. No obvious trauma
  ○ Neuro: Alert and oriented ×3, sensation intact
  ○ Skin: Moist skin, warm to touch

Critical Actions:

1. Assess scene safety
  - Move patients as needed to avoid repeat strike
2. Reverse triage
  - Begin basic life support on victim #1 first
  - Continue rescue breathing after return of spontaneous circulation (ROSC)
3. C-spine immobilization
  - Victims #1 and #2
    - SAM splints (brand of prefabricated splints hikers often carry) in hikers’ bag
4. Reduce versus splint and evacuate
  - If reduce: Explain reasoning and procedure (patient has no neurovascular compromise)
  - If not, explain reasoning and immobilization
5. Victim #3
  - Don’t let him interfere with care of victims 1 and 2

Discussion:

1. Review epidemiology of lightning strikes
2. Review pathophysiology of injuries and signs/symptom of lightning strike
  - Lichtenberg figures
  - Neurologic/vasogenic
  - Pressure related
  - Internal
3. Review treatment
  - Reverse triage

Discussion

1. Review epidemiology of lightning strikes
  - Roughly 40 deaths per year in the US with an overall declining incidence over the last 50 years
  - About 400 lightning injuries occur per year in the US
  - Worldwide
    - Lightning strikes the earth more than 100 times each second
  - Trends in strikes
    - Who is at the most risk?
      1. Males - 80% of strikes involving people involve males
      2. Ages
        - 20–45 years of age
        - Rural farmers, construction works are at increased risk of sustaining a strike
    - Where are strikes most common?
      1. Florida and Texas –
        - ¼ of all US strikes occur here
        - May to September are the most common times
    - When is a lightning strike most likely to occur?
      1. In the afternoon
      2. Right before or after a storm cloud is overhead.
      3. Lightning can occur without any visible storm overhead
  - Lightning physics
    - Ice particle are the key
      As they rise and sink within the cloud, collisions cause a charge separation. Positive charged ice crystals rise to the top and negative particles drop to the bottom. The resultant electrical differential leads to lightning.
2. Review pathophysiology of injuries and signs/symptom of lightning strike
  - Types of lightning strikes
    - Direct strike
      1. Lightning strikes the victim directly. These are the most deadly
    - Side splash
      1. Initial strike hits another object and then jumps to the victim. This is the most common cause of lightning injury
    - Ground current
      1. Lightning strikes the ground or another object first, then the current travels through the ground to the victim
      2. Common in multi-victim injuries
    - Blunt Trauma
      1. Concussive forces of the strike or the victim being thrown
  - Cardiovascular
    - Massive electrical stimulus can lead to
      1. Asystole and respiratory failure from brainstem stunning
      2. Asystole often resolves spontaneously given inherent automaticity of the heart, but the respiratory arrest may persist, leading to hypoxia, dysrhythmia and death
    - Global cardiac dysfunction, atrial and ventricular dysrhythmias, and coronary spasm are possible
  - Neurologic
    - Direct head trauma related injuries
      1. Especially with cranial burns (four times more likely to die)
    - Confusion and amnesia are common symptoms
    - Neuropsychiatric (concussion-like symptoms)
      1. Sleep disturbances
      2. Memory impairment
      3. Personality changes
    - Keraunoparalysis
      1. Typically transient lower extremity paralysis
        - Extremity may be mottled, cold and insensate
        - Usually improves in less than 24 hours
  - Dermatologic
    - Lichtenberg figures
      1. Also called ferning and feathering
      2. Pathognomonic for lighting strikes
      3. This is not a burn and there is no damage to skin
      4. It presents typically within one hour, but usually gone in 24 hours No intervention is necessary
    - Burns
      1. Deep burns are rare
      2. Linear burns
        - Typically partial-thickness
        - Occur on sweat and rain-covered areas of the body
        - Due to vaporization of sweat or rainwater into steam on the patient’s body
        - Treated as any other superficial burn
      3. Thermal
        - Occur from a metal object becoming superheated, such as belt-buckles, earrings and necklaces
      4. Punctate
        - Multiple, small, circular burns
  - Ocular
    - 50% of victims will have an eye injury
    - Cataracts are most common and are often bilateral
  - Ear
    - 30%–50% of lightning strike victims will have a perforated tympanic membrane
3. Review treatment
  - Reverse Triage
    - Treat those that look dead first. The heart is often stunned, not dead. This is the opposite of traditional mass casualty triage
    - CPR immediately if you suspect cardiopulmonary arrest
      1. CPR until intrinsic pacer resumes. Patients may still need respiratory support after cardiac recovery
  - If they don’t look dead initially, then they are likely to survive
  - Remember that lightning *can* strike the same place twice and therefore scene safety is a priority

#### Trailside Talk – *Borrelia burgdorferi*

Background:

You’ve been hiking all summer, really enjoying 4^th^ year of medical school before starting residency. A few days ago, you noticed a rash on your right leg, and it has gotten bigger. You otherwise feel fine. At a break in the hike today with friends, you notice the spot and decide to show them since it looks pretty cool.

Case:

You are sitting on the trail resting and show your friends your rash, “Check this out!”

- Primary Survey:
  ○ AVPU: Alert and oriented
  ○ MARCH:
    ▪ M – No evidence of massive hemorrhage
    ▪ A – Airway intact
    ▪ R – No respiratory distress
    ▪ C – Central pulses intact
    ▪ H – No evidence of Hyper/hypothermia
      - Hike vs helicopter
- Secondary Survey:
  ○ S – Rash on your right leg that has gotten larger. Felt hot last night.
  ○ A – None
  ○ M – None
  ○ P – No past medical/surgical history
  ○ L – 3 hours ago
  ○ E – See background above
- Physical Exam:
  ○ General: Awake, alert and oriented.
  ○ HEENT:
    ▪ Head: Atraumatic
    ▪ Eyes: Open, midline, ocular movements intact, pupils equal and reactive.
    ▪ Ears: External normal
    ▪ Nose: Midline, no septal hematoma
    ▪ Oropharynx: Moist mucosa, no dental injuries or malocclusion
  ○ Neck:
    ▪ No Crepitus, no palpable hematoma, no c-spine tenderness
  ○ Chest:
    ▪ No external deformity, no crepitus
    ▪ Pulses/heart: Regular rate and rhythm
    ▪ Respiratory: Breath sounds equal bilaterally
  ○ Abdomen/Pelvis:
    ▪ Non-tender in all four quadrants, non-distended
  ○ Extremities:
    ▪ Right thigh: Target rash (moulage or have picture of erythema migrans)
    ▪ Skin Intact. Pulses intact.
  ○ Neuro:
    ▪ Alert and oriented ×3, neurovascularly intact
  ○ Skin:
    ▪ Moist skin, warm to touch. No petechiae

Critical Actions:

1. Recognize rash as characteristic of Lyme disease
2. Transport
  - Safe to hike out but needs to be seen soon
3. Disposition
  - See his physician in the next day or the emergency department if unable
  - Bonus if they tell patient what other symptoms to monitor for

Discussion:

1. Review epidemiology of Lyme disease and vector
2. Discuss signs/symptoms
  - Early
  - Disseminated
3. Review evaluation and management[Fig f4-jetem-5-1-c1]

Bauer S. Adult deer tick. In: Wikimedia Commons. https://commons.wikimedia.org/wiki/File:Adult_deer_tick.jpg. Public domain.[Fig f5-jetem-5-1-c1]

Gathany J. Erythema migrans. In: Wikimedia Commons. https://commons.wikimedia.org/wiki/File:Erythema_migrans_-_erythematous_rash_in_Lyme_disease_-_PHIL_9875.jpg. Public domain.

Discussion

1. Review epidemiology of Lyme disease and vector
  - The vector is the *Ixodes scapularis* (deer tick) and *Ixodes pacificus*
    - Spirochete *Borrelia burgdorferi*
  - Centers for Disease Control (CDC): The tick must be attached over 36 hours for transmission to occur
  - Peak transmission occurs in summer months
  - Endemic areas are the Northeastern, Midwest and Pacific coasts
2. Discuss signs/symptoms
  - Early
    - Localized symptoms such as the rash and local irritation
    - Will occur around one week after bite
    - Flu-like illness may also occur with myalgias and fatigue
    - Rash
      1. Erythema migrans
        - Redness spreads with a central clearing often called a target lesion
  - Disseminated
    - Occurs weeks to months after exposure
    - Polyarticular arthralgia and myalgias
    - Carditis
      1. <10% will develop this
      2. Atrioventricular blocks may also occur
    - Neurological
      1. Cranial neuropathies (this can be a Bell’s palsy mimic)
      2. Radiculopathy
      3. Meningitis
      4. Peripheral neuropathy
3. Review evaluation and management
  - Diagnosis is difficult to make; the CDC recommends a two-step approach
    1. ELISA: Detects antibodies to *Borrelia burgdorferi*, although false positives are common
    2. Western blot: used as a confirmatory test
  - Management
    - Early Localized
      1. Doxycycline for 10–21 days
      2. Cefuroxime or Amoxicillin for 14–21 days for:
        - Adults allergic to doxycycline
        - Children less than 8
        - Pregnant or breast-feeding women
    - Disseminated
      1. May required parenteral dosing of extended duration (14–28 days)

### Appendix K Session 8: Extensions of Austere Medicine

The primary goal of this session is to expose students to natural extensions of wilderness (austere) medicine principles. This session will be classroom based. There is no pre-reading since students receive the final examination the day prior. During this session local experts in these extensions will present their experiences, highlighting the austere principles needed. If physicians with experience and expertise are not available for this content, the course instructor may substitute other activities for this day.

**Session 8 Objectives:** After participation, the learner should be able to:

1. Discuss extensions of austere medicine, including disaster response, international medicine in the developing world, and remote event medicine

**Instructional Materials**

1. Projected presentation materials

**Pre-Reading**

None: Students are expected to begin work on the final examination the day prior.[Table t10-jetem-5-1-c1]

**Table t10-jetem-5-1-c1:**

Time (min)	Segment	Description
0–45	International medicine	Presentation of austere medicine principles through international medical work in developing countries.
45–60	Break	Break and informal discussion with speakers
60–105	Disaster medicine	Presentation of austere medicine principles through disaster response.
105–120	Break	Break and informal discussion with speakers.
120–165	Event medicine	Presentation of austere medicine principles through event medicine.
165–180	Break	Break and informal discussion with speakers.
180–210	Expedition Operations	Students will present their final expedition plan.

### Appendix L Session 9: Course Expedition

The primary objective of this final session is to provide an opportunity for integration and practice of course material. This session will be held at a final local or national park. Students will plan the logistics of this operation throughout the rest of the course and present their plan at the end of the prior session. During this session students will conduct a simulated search and rescue operation for a 26-year-old male or female (the instructor may play this patient) with a fractured tibia unable to self-extricate, requiring stabilization of the fracture and use of a litter. Once the patient is stabilized and carried for 30 minutes, this portion will conclude.

Students will be given the UTM coordinates at the beginning of the course and will be in charge of planning logistics, including equipment, transportation, navigation, and medical supplies. Note: the instructor will need to identify an appropriate location and plot coordinates. Throughout this expedition, each student will also have a secret case, of which only they and the faculty are aware. At any point during this expedition the student can act out their case, with the others required to respond. Each case will highlight various parts of the course material. During the evening of the first day, students will present their plants of toxicologic significance around a campfire.

**Session 9 Objectives:** After participation, the learner should be able to:

1. Plan expedition of and manage common wilderness medicine scenarios
2. Teach other learners about plants of toxicologic significance

**Instructional Materials**

To be determined by students

Minimum: compass, USGS map, medical kit, sleeping bag, tent, food/water, weather appropriate clothing

**Pre-Reading**

None

#### Trailside Talks – *Gyromitra esculenta*

Background:

While out foraging for morels and camping, you woke up this morning with vomiting and diarrhea. You are now asthenic and dry heaving. You have travelled to the stream to rinse off but were too tired and are on the ground dry heaving, which is where the other students will find you. You ate some of what you found last night, but instead of *Morchella esculenta* (morel), you apparently accidentally picked *Gyromitra esculenta* (false morel). Do not tell the learners what you were doing or ate last night unless asked.

**Case:**

You are found dry heaving, tired.

- Primary survey:
  ○ AVPU: awake, alert
  ○ MARCH:
    ▪ M – No evidence of massive hemorrhage
    ▪ A – Airway Intact
    ▪ R – Slightly tachypneic
    ▪ C – Pulses intact, tachycardic
    ▪ H – No evidence of hypo/hyperthermia
      - Hike vs helicopter
- Secondary survey:
  ○ S – Vomiting, diarrhea
  ○ A – Penicillin
  ○ M – Ibuprofen
  ○ P – None
  ○ L – Night prior
  ○ E – As noted in background above
- Physical exam:
  ○ General: Awake, oriented, tired.
  ○ HEENT:
    ▪ Head: Atraumatic
    ▪ Eyes: Pupils normal
    ▪ Ears: External normal
    ▪ Nose: Normal
    ▪ Oropharynx: Dry mucosa
  ○ Neck:
    ▪ Nontender, supple
  ○ Chest:
    ▪ Cardiovascular: Tachycardic, rate 125
    ▪ Respiratory: Tachypneic, rate in low 20s
  ○ Abdomen/Pelvis:
    ▪ Non-tender in all four quadrants, non-distended
  ○ Extremities:
    ▪ Moves all extremities. No obvious trauma
  ○ Neuro: Altered, normal neuro exam
  ○ Skin: Dry, no rash

**Branch Point:**

- A: Learners should identify that this is delayed toxicity from mushroom ingestion and call for Emergency Medical Services (EMS) to transport to hospital. Once they do this, you will start to seize.
- B: After a minute or two of seizure, EMS will arrive. Have the students act as EMS. They should have concern for hypoglycemia as a complication and evaluate glucose. It will be normal at 93. Treatment with benzos should be started.
- C: At this point, they can assume they are in ED. You are still seizing. Walk them through status pathway if needed. None of it will work. If they do not identify need for pyridoxine (70mg/kg, 5g max), suggest they should call Poison Control Center (PCC). Once pyridoxine given, seizure stops.

Critical Actions:

1. Identify delayed mushroom toxicity
2. Evaluate glucose
3. Treat seizure
  - Benzodiazepines
  - Pyridoxine

Discussion:

1. Review false vs true morel and dangers of mushroom identification
2. Review signs/symptoms of false morel ingestion
  - Discuss early vs delayed onset of GI symptom as helpful for prognosis of unknown mushroom ingestion
  - Seizure and pathophysiology as related to isoniazid poisoning
3. Review management
  - Use of poison control center
  - Supportive
  - Treatment of seizures

Discussion:

1. Review false vs true morel and dangers of mushroom identification
  - The false morel is NOT the same as the delicious smoky flavor of the true morel (genus Morchella) but has the same “brain-like” appearance and can be *falsely* identified to even experienced scavengers.
2. Review signs/symptoms of false morel ingestion
  - In general, patients with early onset gastrointestinal (GI) symptoms will have a benign course. Delayed onset of GI symptoms is concerning and should be taken seriously. It suggests symptoms are not from direct effects of the toxin in the mushroom, but downstream effects, such as liver failure.
  - Seizures from false morel ingestion result from inactivation of vitamin B6 (pyridoxine) leading to decreased GABA synthesis similar to isoniazid poisoning.
3. Review management
  - Poison Control Centers (in the United States or other countries with these) are a great resource. Call early for cases because they can help direct care.
  - Aside from specific mushrooms such as the false morel and *Amanita phalloides*, supportive care is the mainstay of treatment. Even for the mushrooms noted, supportive care is still a critical component. For mushroom ingestions, this often means monitoring glucose, electrolytes, hydration status, renal function, and liver function closely. More critical patients will need respiratory/ventilatory support.
  - For false morels, treatment of seizures with B6 can be lifesaving. This is similar to the treatment of isoniazid poisoning because pyridoxine may be the only medication to stop the seizures. While pyridoxine is important, benzodiazepines are still the first line treatment for seizures if the cause or specific mushroom is unknown.
    - Hypoglycemia is an important cause of seizures to evaluate for quickly, even in mushroom ingestion.

#### Trailside Talks – Barotrauma (Middle Ear Squeeze)

Background:

You are celebrating the end of 4^th^ year with a scuba trip to Turks and Caicos. Your friend seems a bit under the weather (“just a spring cold”) but decided to give it a chance anyway. When you suggest that diving might not be the best idea, your friend replies, “Don’t worry, it’s no big deal.” You wonder, is that true…?

Case:

Your friend returns to the boat clutching her ear, clearly in terrible pain.

- Primary survey:
  ○ AVPU: alert and oriented
  ○ MARCH:
    ▪ M – No evidence of massive hemorrhage
    ▪ A – Airway Intact
    ▪ R – Respiration normal
    ▪ C – Pulses intact
    ▪ H – No evidence of hyper/hypothermia
      - Hike vs helicopter
- Secondary Survey:
  ○ S – “My ear…it hurts so much…is that blood? Is my ear bleeding!?”
  ○ A – None
  ○ M – None
  ○ P – No past medical history, no past surgical history
  ○ L – 2 hours ago
  ○ E – “We just got here two days ago for an end of med school celebration. I’ve been a bit snotty with this cold, but otherwise I’ve been good.”
- Physical Exam:
  ○ General: tearful, clutching right ear
  ○ HEENT:
    ▪ Head: Atraumatic
    ▪ Eyes: Open, midline, ocular movements intact, pupils equal and reactive
    ▪ Ears: External normal, blood in the external auditory canal, perforated tympanic membrane (if able to look)
    ▪ Nose: Midline, no septal hematoma
    ▪ Oropharynx: Moist mucosa, no dental injuries or malocclusion
  ○ Neck:
    ▪ No crepitus, no palpable hematoma, no palpable step-off
  ○ Chest:
    ▪ No external deformity, no crepitus
    ▪ Cardiovascular: Regular rhythm, pulses bounding
    ▪ Respiratory: Breath sounds bilateral, no respiratory distress
  ○ Abdomen/Pelvis:
    ▪ Non-tender in all four quadrants, non-distended
  ○ Extremities:
    ▪ Moves all extremities. No obvious trauma
  ○ Neuro:
    ▪ Alert and oriented ×3, sensation intact
  ○ Skin:
    ▪ Moist skin, warm to touch

Critical Actions:

1. Assessment of other signs of trauma
2. Do not let patient back in the water

Discussion:

1. Review pathophysiology and signs/symptoms of diving barotrauma
  - Mask squeeze
  - Barotitis media
  - Barosinusitis
  - Labyrinthine window rupture
2. Discuss management

Discussion:

1. Review pathophysiology and signs/symptoms of diving barotrauma: any air compartment is at risk for rupture due to Boyle’s law (pressure and volume are inversely proportional).
  - Mask squeeze – pressure in mask decreases with descent, leading to relative negative pressure area in front of the face. This can rupture blood vessels in the face.
  - Barotitis media – with descent, the volume of middle ear decreases (unless able to equalize through eustachian tube) until it ruptures. Patient can experience pain and dizziness, although prominent dizziness is concerning for inner ear or central nervous system pathology.
  - Barosinusitis – sinus pressure and pain as a result of sinuses that are not able to equalize pressure, often due to congestion present beforehand.
  - Labyrinthine window rupture – occurs from similar mechanisms as above. Vertigo is the main symptom.
2. Discuss management
  - Treatment of barotitis media is often supportive. The patient should not be allowed to dive again because this can introduce further fluid into the middle ear and increase risk of further injury to other structures. Antibiotics are indicated depending on the type of water and concern for contamination.

#### Trailside Talk - Coral Snake Envenomation

Background:

You’re celebrating the end of a long day at the Resident Wilderness Medicine Day at a camp-out with a few of your friends and some favorite brews. You hear one of your more inebriated co-residents say: “Listen, it’s just a king snake. I’ll pick it up. It’s harmless. Remember, we just learned the rhyme…’red on yellow, don’t do jack, red on black, dead on your back.’” You think…is that right…?

Case:

Your friend decides to pick up a “harmless” snake when he is accidentally bitten on the finger.

- Scene Survey
  ○ The snake is slithering away (prop coral snake/image)
- Primary Survey:
  ○ AVPU: Alert and oriented
  ○ MARCH:
    ▪ M – No evidence of massive hemorrhage
    ▪ A – Airway intact
    ▪ R – Tachypneic, but no distress
    ▪ C – Pulses intact
    ▪ H – No evidence of hyper/hypothermia
      - Hike vs helicopter
- Secondary Survey:
  ○ S – Snake bite to the right long finger
  ○ A – Penicillin
  ○ M – Occasional marijuana
  ○ P – No medical or surgical history
  ○ L – 2 hours ago (numerous beers though)
  ○ E – “I was trying to pick up that king snake when the jerk bit me. Where is that snake? I’m going to kill it (victim moves toward snake, then stops to steady self) … I don’t feel so great.”
- Physical Exam:
  ○ General: Alert. Inebriated.
    ▪ Developing symptoms
      - Nauseated → vomiting→abdominal pain
      - Headache
      - Drowsiness
      - Diaphoresis
  ○ HEENT:
    ▪ Head: Atraumatic
    ▪ Eyes: Open, midline, ocular movements intact, pupils equal and reactive
      - Eyelids drooping
    ▪ Ears: External normal
    ▪ Nose: Midline, no septal hematoma
    ▪ Oropharynx: Moist mucosa, normal
      - Does develop peri-oral numbness
  ○ Neck:
    ▪ No crepitus, no palpable hematoma, no palpable step-off
  ○ Chest:
  ▪ No external deformity, no crepitus
    ▪ Cardiovascular: Tachycardic
    ▪ Respiratory: Breath sounds equal bilateral, hyperventilating
  ○ Abdomen/Pelvis:
    ▪ Generalized cramping pain
  ○ Extremities:
    ▪ Moves all extremities. No obvious trauma
  ○ Neuro: Inebriated. Initially intact
    ▪ Developing symptoms
      - Facial numbness
      - Paresthesia
      - Headache
  ○ Skin: Moist skin, small bite marks for finger (moulage)
    - Small, hard to see bite

Critical Actions:

1. Assure scene safety
  - Make sure venomous snake is gone
  - Don’t let victim try to kill it
2. ID snake
  - Snap a pic
3. Constriction band & splint
  - Australian pressure bandage & immobilization
4. Evacuate

Discussion

1. Review snakes:
  - Elapids
  - Crotalids
2. Discuss pathophysiology of venoms and presentation
  - Elapid
  - Crotalid
3. Review ED Evaluation
4. Discuss Treatment
  - Crotalid
  - Elapid

Discussion

1. Review snakes:
  - Elapids
  - Crotalids
2. Discuss pathophysiology of venoms and presentation
  - Elapid – primarily neurotoxic venom
  - Crotalid – venom is hemotoxic, myotoxic, and neurotoxic; which is more prominent varies by snake and region.
3. Review ED evaluation
  - Initial evaluation should assess for systemic stability and any threat to the limb impacted (neurovascular compromise). Swelling should be noted and outlined for monitoring. Circumferential measurements are helpful for monitoring as well. Depending on the type of snake, laboratory evaluation should look for systemic effects such as hemolysis and rhabdomyolysis.
4. Discuss Treatment
  - Crotalid – Treatment for localized symptoms is often supportive. Antivenom (Crofab) is used for systemic symptoms or rapidly progressing swelling.
  - Elapid – supportive care is the mainstay of treatment. This may mean respiratory support with intubation and a ventilator. There is antivenom, although the US stockpile of antivenom expired in 2008. New possibilities are being researched. Call poison control center early for assistance.

#### Trailside Talk - Femur Fracture

Background:

You spent your shift debating with a co-resident in the pediatric emergency department that despite your clumsy demeanor, you are in fact quite agile and even possess the uncanny ability to always land on your feet whenever you fall. To prove this to your friend, you’ve agreed to demonstrate this skill by performing a backflip from a tree at a local park.

Case:

You are lying on the ground beside the trail, screaming. There is a slight angulation to the right mid-thigh.

- Primary Survey:
  ○ AVPU: Alert and oriented
  ○ MARCH:
    ▪ M – No evidence of massive hemorrhage
    ▪ A – Airway intact
    ▪ R – Tachypneic, but no distress
    ▪ C – Central pulses intact
    ▪ H – No evidence of hyper/hypothermia
      - Hike vs helicopter
- Secondary Survey:
  ○ S – “My leg hurts! Holy smokes, did I land it? Aahhh, help me!”.
  ○ A – None
  ○ M – None
  ○ P – None
  ○ L – 6 hour ago
  ○ E – “Dude, I was just doing a back-flip from that tree branch. I’ve done it a hundred times. I think I landed a little funny. You think I broke my leg?”
- Physical Exam:
  ○ General: Awake, alert and oriented. In quite a bit of pain
  ○ HEENT:
    ▪ Head: Atraumatic
    ▪ Eyes: Open, midline, ocular movements intact, pupils equal and reactive
    ▪ Ears: External normal
    ▪ Nose: Midline, no septal hematoma
    ▪ Oropharynx: Moist mucosa, no dental injuries or malocclusion
  ○ Neck:
    ▪ No crepitus, No palpable hematoma. There is diffuse c-spine tenderness
  ○ Chest:
    ▪ No external deformity, no crepitus
    ▪ Cardiovascular: Tachycardic
    ▪ Respiratory: Breath sounds equal bilateral
  ○ Abdomen/Pelvis:
    ▪ Non-tender in all four quadrants, non-distended
  ○ Extremities:
    ▪ Right Leg: Shortened. Bulge to mid-thigh.
    ▪ Skin intact. Pulses intact. Leg warm.
  ○ Neuro:
    ▪ Alert and oriented ×3, neurovascularly intact
  ○ Skin:
    ▪ Moist skin, warm to touch

Critical Actions:

1. Scene safety
2. Immobilize right lower extremity
  - Can make traction splint (if know how) or buddy-tape legs
3. Reassess neurovascular exam after splint
4. Transport
  - Team to make a litter and carry you 50ish feet (whatever convenient distance)
    - Can be rope, tarp, coats
    - Supplies will be at site

Discussion:

6. Discuss importance of scene safety
7. Review and practice reduction and immobilization techniques, including c-spine
8. Discuss and practice patient transportation methods

Discussion:

1. As with all wilderness scenarios, scene safety is important. If the rescuer becomes another victim, the chances of survival for either diminishes.
2. Review and practice reduction and/or immobilization techniques
  - C-spine
  - Shoulder
  - Finger/Toe
  - Knee/Patella
  - Ankle
3. Discuss and practice patient transportation methods
  - Creating litters from backpack, tarps, rope
  - Fireman carry
  - Others

#### Trailside Talks - Frostbite

Background:

You have been backpacking in the snow for about 8 hours when one of your classmates states that he feels his feet are becoming numb, and he is having trouble walking. He forgot his boots and had to hike in his tennis shoes. The campsite is another few miles away. He tells you he’s fine and his socks are just a little wet, that he’ll just warm them by the fire when he gets there. You wonder, is that all this is…

Case:

Your friend has numbness and pain in bilateral feet after walking in the snow for several hours.

- Primary Survey:
  ○ AVPU: Alert and oriented
  ○ MARCH:
    ▪ M – No evidence of massive hemorrhage
    ▪ A – Airway intact
    ▪ R – Respiration normal
    ▪ C – Pulses intact
    ▪ H – No evidence of hyper/hypothermia
      - Hike vs helicopter
- Secondary Survey:
  ○ S – Pain in bilateral feet, more in toes
  ○ A – None
  ○ M – None
  ○ P – Reflux
  ○ L – 8 hours ago
  ○ E – “I forgot my boots. My feet are really starting to hurt and I can’t feel my toes. I’ll be ok though. Only a few more miles to the campsite and I can warm them over the fire.”
- Physical Exam:
  ○ General: Slow, antalgic gait
  ○ HEENT:
    ▪ Head: Atraumatic
    ▪ Eyes: Open, midline, normal
    ▪ Ears: External normal
    ▪ Nose: Midline, no septal hematoma
    ▪ Oropharynx: Moist mucosa, no dental injuries or malocclusion,
  ○ Neck:
    ▪ No crepitus, no palpable hematoma, no palpable step-off
  ○ Chest:
    ▪ No external deformity, no crepitus
    ▪ Cardiovascular: Regular rate and rhythm, diminished dorsalis pedis and posterior tibialis pulses
    ▪ Respiratory: Breath sounds bilateral, no respiratory distress
  ○ Abdomen/Pelvis:
    ▪ Non-tender in all four quadrants, non-distended,
  ○ Extremities:
    ▪ Moves all extremities. No obvious trauma, edema to bilateral feet
  ○ Neuro: Alert and oriented ×3, numbness to bilateral feet
  ○ Skin:
    ▪ Pale, yellow-white on dorsum of feet. Toes with areas of erythema and clear blisters

Critical Actions:

1. Recognize that patient is developing frostbite
2. Immediate evacuation vs rewarming – explain rationale for their choice
3. Patient wants to “warm his feet over the fire” – must discuss appropriate rewarming
4. Pain control

Discussion:

1. Discuss pathophysiology of cold-induced injuries
  - Life vs. limb
2. Describe clinical features of freezing & non-freezing cold injuries
  - Frostbite
  - Frostnip
  - Chilblain
  - Immersion foot
3. Discuss management of cold-induced injuries
  - Evacuation
  - Out-of-hospital vs. in-hospital
4. Discuss rewarming
  - Thaw-refreeze

Discussion:

1. Discuss pathophysiology of cold-induced injuries
  - Cellular level damage from ice crystals is the primary component
  - Microvascular clotting is also increasingly recognized as a factor
2. Describe clinical features of freezing & non-freezing cold injuries
  - Frostbite – freezing of the skin.
  - Frostnip – pale skin due to superficial vasoconstriction. Numbness may be present. Skin is still pliable. No permanent damage.
  - Chilblain – irritation and pain from inflammation due to recurrent exposure to the cold, without freezing. May have redness and blistering.
  - Immersion foot – due to cold, constantly wet feet. Painful, red, and may lead to necrosis and gangrene.
3. Discuss management of cold-induced injuries
  - Evacuation is vital for definitive evaluation and treatment. Immediate movement to a location where warmth of impacted area can be guaranteed and sustained.
  - Out-of-hospital management focuses on keeping the extremity safe from further damage. If there is a chance that the area of frostbite will refreeze, rewarming should not be attempted out-of-hospital. If it can be maintained, then initiation immediately is helpful. In the hospital, rewarming is often accomplished with warm water immersion (40C). This can be a painful process, so analgesia may be needed. Discussion with a vascular surgeon is also warranted because thrombolysis may be beneficial due to microvascular clotting.
4. Discuss rewarming
  - As noted above, thaw with subsequent refreeze is more harmful than initial evacuation and should be prevented.

#### Trailside Talks – Hyperthermia

Background:

You’ve recently become a nocturnist and have found it challenging adjusting to life on the dark side. To help with this, your buddy gave you some “stay-awake vitamins.” To complicate things more, you allowed your significant other to sign you up for a marathon, which you’ve been training for over the last several hours.

Case:

You collapsed while training for a marathon on the path at Conestee Park. You’re awake, but disoriented. You can give some sparse history initially, but you won’t be fully able to cooperate until cooling measures are started.

- Primary Survey:
  ○ AVPU: Awake, confused
  ○ MARCH:
    ▪ M – No evidence of massive hemorrhage
    ▪ A – Airway intact
    ▪ R – Respiration Intact
    ▪ C – Pulses bounding
    ▪ H – Skin hot to touch
      - hike vs helicopter
- Secondary survey:
  ○ S – “I was…just…running…what happened? I feel like I’m going to pass out.”
  ○ A – no known drug allergies (NKDA)
  ○ M – Some “stimulant vitamin”
  ○ P – None
  ○ L – 8 hours ago
  ○ E –
    ▪ Before cooling: limit info you will give.
    ▪ After cooling started:
      - “I’m training for a marathon. I’ve been running a few hours. I took one of my “stay-awake pills” because I worked last night.”
      - “I haven’t eaten since midnight (crackers on shift).
- Physical exam:
  ○ General: Diaphoretic, hot to touch, awake but disoriented
  ○ HEENT:
    ▪ Head: Atraumatic
    ▪ Eyes: Open, midline, pupils equal, round, and reactive to light (PERRL)
    ▪ Ears: External normal
    ▪ Nose: Midline, no septal hematoma
    ▪ Oropharynx: Dry mucus membranes, no dental injuries or malocclusion
  ○ Neck:
    ▪ No crepitus, no palpable hematoma, no palpable step-off
  ○ Chest:
    ▪ No external deformity, no crepitus
    ▪ Cardiovascular: tachycardia, pulses bounding
    ▪ Respiratory: tachypnea
  ○ Abdomen/Pelvis:
    ▪ Non-tender in all four quadrants, non-distended
  ○ Extremities:
    ▪ Moves all extremities. No obvious trauma
  ○ Neuro:
    ▪ Oriented to self, garbled speech
  ○ Skin:
    ▪ Moist skin, hot to touch

Critical Actions:

1. Active cooling
  - Cold-water immersion or mist-fan
2. Oral hydration / food
  - If not given oral hydration/food with cooling, start becoming altered again
3. Refuse hospital evaluation initially
  - “Why do I need to go to the hospital? I’m feeling much better.”
  - If they let you stay – start to become altered again
4. Thermoregulation question:
  - “Do you think that medicine I take may have contributed to this? Why?”

Discussion:

1. Discuss mechanisms of heat regulation.
2. Pathophysiology of heat-related injury
3. Clinical manifestations of heat injury
  - Mild to Severe (Heat Stroke)
4. Management of heat illnesses

Discussion:

1. Discuss mechanisms of heat regulation
  - Typically, the body regulates heat with evaporation, convection, conduction, and radiation. The body increases sweating in response to heat, although evaporation is less helpful as humidity increases. Cutaneous vasodilation is also helpful, increasing the surface exposure of warm blood, trying to take advantage of radiation, convection, and conduction. These are less helpful as the ambient temperature increases.
2. Pathophysiology of heat-related injury
  - Initial shunting of blood flow and increased sweating can lead to dehydration and decreased blood flow to central organs. Eventually the drive for central flow leads the body to peripherally vasoconstrict and prioritize flow to core organs. This reduces the ability to regulate heat and increases the exposure of core organs to heat, resulting in more damage and eventually denaturing of proteins.
3. Clinical manifestations of heat injury
  - Heat exhaustion – temperature over 101°F and symptomatic (fatigue, muscle cramps, headache)
  - Heat Injury – temperature over 101°F with end organ damage (elevated creatinine, acute liver injury, etc)
  - Heat Stroke – temperature above 104°F with associated neurologic changes (altered mental status, ataxia, etc.)
4. Management of heat illnesses
  - Cessation of activity and immediate cooling saves lives
  - Cooling can be through submersion; ice packs to the axillae, neck, and groin, or evaporative cooling. Choice of which depends on what is available and need for other care. It is difficult to monitor with a patient submerged. Immediate ice water submersion is preferred for exertional heat stroke if possible due to rapidity of response. In the ED, evaporative cooling is often preferred due to need for monitors.

#### Trailside Talks – Jellyfish

Background:

You are celebrating the end of 4^th^ year with a trip to Turks and Caicos. You’ve been snorkeling with friends all day long, and as you’re exiting the water for the evening, you hear one in your party scream, “Ouch, something just stung me…I think it was a Box Jellyfish.” Another, slightly inebriated friend actively untying his trunks, says “I got this, everyone knows you pee on jellyfish stings...” You wonder, is that true…?

Case:

Your friend has just been stung by a jellyfish in the Caribbean.

- Primary Survey:
  ○ AVPU: Alert and oriented
  ○ MARCH:
    ▪ M – No evidence of massive hemorrhage
    ▪ A – Airway intact
    ▪ R – Respiration normal
    ▪ C – Pulses intact
    ▪ H – No evidence of hyper/hypothermia
      - Hike vs helicopter
- Secondary Survey:
  ○ S – “Oh man, that hurts so much”
  ○ A – None
  ○ M – None
  ○ P – No past history
  ○ L – 2 hours ago meal, intermittent beer throughout the day
  ○ E – “We’ve been snorkeling all day. It was great until I got stung. It really hurts.”
- Physical Exam:
  ○ GEN: Rubbing legs vigorously, clearly in pain
  ○ HEENT:
    ▪ Head: Atraumatic
    ▪ Eyes: Open, midline, ocular movements intact, pupils equal and reactive
    ▪ Ears: External normal
    ▪ Nose: Midline, No septal hematoma
    ▪ Mouth: Moist mucosa, No dental injuries or malocclusion,
  ○ Neck:
    ▪ No crepitus, No palpable hematoma, No palpable step-off
  ○ Chest:
    ▪ No external deformity, No crepitus
    ▪ CV: Regular rate and rhythm
    ▪ Respiratory: Breath sounds bilateral, No respiratory distress
  ○ Abdomen/Pelvis:
    ▪ Non-tender in all four quadrants, non-distended
  ○ Extremities:
    ▪ Moves all extremities. No obvious trauma.
  ○ Neuro:
    ▪ Alert and oriented ×3, sensation intact
  ○ Skin:
    ▪ Long, serpiginous erythematous streaks

Critical Actions:

1. Correctly identify as an envenomation.
2. Inactivation of nematocyst
  - Acetic acid
3. Removal of nematocyst with shaving/scraping
  - After inactivation – or pain gets worse

Discussion:

1. Discuss clinical features of nematocyst-related injuries
  - Jellyfish & Man-O-War
2. Describe general management of nematocyst-related injuries
  - Out-of-hospital & in-hospital
3. Evacuation guidelines & antivenom

Discussion:

1. Discuss clinical features of nematocyst-related injuries
  - Jellyfish – mainly irritating. Erythema, pain and swelling are usually localized to site of contact.
  - Man-O-War – symptoms can be more severe.
  - Box Jellyfish – can lead to death due to increased cell permeability and hyperkalemia.
  - Irukandji jellyfish in Australia is a box jellyfish.
2. Describe general management of nematocyst-related injuries
  - Removal of nematocysts is important. Use salt water because fresh water may lead to further discharge.
  - Venom is often heat labile, so immersion in warm water may help.
3. Evacuation guidelines & antivenom
  - In areas where box jellyfish are present or whenever symptoms are severe or rapidly progressing, immediate evacuation to a hospital is important.
  - Antidote/antivenom is available for some box jellyfish. Contact Poison Control Center early.

#### Trailside Talks – Decompression Sickness

Background:

You are celebrating the end of 4^th^ year with a scuba trip to St. Lucia. You were incredibly active, and yesterday your group climbed in the morning and went diving that afternoon (you missed diving because of a cold). You are now about to fly back home when you friend begins rubbing her shoulder. She tells you, “I think I pulled a muscle yesterday climbing.” You wonder, is that all this is…?

Case:

Your friend is developing joint pain after a diving trip before getting on a plane at the airport.

- Primary Survey:
  ○ AVPU: Alert and oriented
  ○ MARCH:
    ▪ M – No evidence of massive hemorrhage
    ▪ A – Airway intact
    ▪ R – Respiration normal
    ▪ C – Pulses intact
    ▪ H – No evidence of hyper/hypothermia
      - Hike vs helicopter
- Secondary Survey:
  ○ S – “My arm is killing me…I don’t remember doing anything climbing yesterday. It’s crazy. You know, my other elbow is sore too…”
  ○ A – None
  ○ M – Oral contraceptive pill
  ○ P – No past medical or surgical history
  ○ L – 2 hours ago
  ○ E – “I can’t think of what I could have done. It was easy climbing yesterday. The diving was relaxed too…wait a minute…You think this is the…Oh, what do they call it, that Radiohead song…You know…”
- Physical Exam:
  ○ General: Anxious, rubbing her right shoulder
  ○ HEENT:
    ▪ Head: Atraumatic
    ▪ Eyes: Open, midline, ocular movements intact, pupils equal and reactive
    ▪ Ears: External normal
    ▪ Nose: Midline, no septal hematoma
    ▪ Oropharynx: Moist mucosa, no dental injuries or malocclusion
  ○ Neck:
    ▪ No Crepitus, no palpable hematoma, no palpable step-off
  ○ Chest:
    ▪ No external deformity, no crepitus
    ▪ Cardiovascular: Regular rate and rhythm
    ▪ Respiratory: Breath sounds bilateral, no respiratory distress
  ○ Abdomen/Pelvis:
    ▪ Non-tender in all four quadrants, non-distended
  ○ Extremities:
    ▪ Moves all extremities. No obvious trauma
      - Pain to range of motion of right shoulder
      - Pain to range of motion of left elbow
  ○ Neuro:
    ▪ Alert and oriented ×3, sensation intact
  ○ Skin:
    ▪ Moist skin, warm to touch

Critical Actions:

1. Assess for non-musculoskeletal signs/symptoms
2. Do not get on the plane

Discussion:

1. Discuss decompression sickness pathophysiology
2. Discuss categorization of decompression illness
3. Review treatment with hyperbarics
4. Discuss risk factors and prevention

Discussion:

1. Discuss decompression sickness pathophysiology: Henry and Boyle’s law explain dissolution of gas at pressure with resultant off-gassing once the dive is over, resulting in pain and DCI (decompression illness)
2. Discuss categorization of DCI
  - Type 1
    - Musculoskeletal, cutaneous, lymphatic
  - Type 2
    - Neurologic, pulmonary, vestibular
3. Review treatment with hyperbarics
  - Navy Dive tables have empirically derived time courses to recompress and allow slower off-gassing. Call DAN (Diver’s Alert Network) for recommendations: 1-800-446-2671.
4. Discuss risk factors and prevention:
  - Risk Factors: Repetitive deep dives, not following dive tables, rapid ascent or flight after dives. Dehydration, obesity, and baseline poor health are also thought to contribute.
  - Prevention: Need to follow NAUI dive tables or similar recommendations for appropriate time underwater at certain depth, may need to stop for decompression after longer dives

#### Trailside Talks – Sea Urchin

Background:

You are celebrating the end of 4^th^ year with a trip to Turks and Caicos. You’ve been snorkeling with friends all day long, and as you’re exiting the water for the evening, you hear someone in your party scream, “Ouch, I think I just stepped on something.” He lifts his foot out of the water and you see numerous black thorn-like objects protruding from his foot. He says, “Oh good, I think it’s just a stick.” You wonder, is that all it was…?”

Case:

Your friend has just been “stung” by a sea urchin.

- Primary Survey:
  ○ AVPU: Alert and oriented
  ○ MARCH:
    ▪ M – No evidence of massive hemorrhage
    ▪ A – Airway intact
    ▪ R – Respiration normal
    ▪ C – Pulses intact
    ▪ H – No evidence of hyper/hypothermia
      - Hike vs helicopter
- Secondary Survey:
  ○ S – “Oh man, that hurts so much!”
  ○ A – None
  ○ M – None
  ○ P – No past history
  ○ L – 2 hours ago meal, intermittent beer throughout the day
  ○ E – “We’ve been snorkeling all day. It was great until I stepped on that stick. It really hurts.”
- Physical Exam:
  ○ General: Rubbing right foot vigorously, clearly in pain
  ○ HEENT:
    ▪ Head: Atraumatic
    ▪ Eyes: Open, midline, normal
    ▪ Ears: External normal
    ▪ Nose: Midline, no septal hematoma
    ▪ Oropharynx: Moist mucosa
  ○ Neck:
    ▪ No crepitus, no palpable hematoma, no palpable step-off
  ○ Chest:
    ▪ No external deformity, no crepitus
    ▪ Cardiovascular: Regular rate and rhythm
    ▪ Respiratory: Breath sounds bilateral, no respiratory distress
  ○ Abdomen/Pelvis:
    ▪ Non-tender in all four quadrants, non-distended
  ○ Extremities:
    ▪ Moves all extremities. No obvious trauma.
  ○ Neuro:
    ▪ Alert and oriented ×3, sensation intact
  ○ Skin:
    ▪ Numerous black barbs protruding from foot.
    ▪ If removed – small punctate wounds, minimal bleeding

Critical Actions:

1. Correctly Identify as an envenomation
2. Removal of spines as able
3. Pain control techniques
  b. Warm water immersion
  c. Adjuncts

Discussion:

1. Discuss clinical features of “stinging fish” injuries
2. Describe general management of “stinging fish” injuries
3. Evacuation guidelines

Discussion:

1. Discuss clinical features of “stinging fish” injuries
  - Lionfish, Scorpionfish, Stonefish: envenomation results in hypotension, weakness, neuromuscular paralysis, cardiac arrhythmias, and pulmonary edema.
  - Stingray: barbed stinger that can require removal
  - Sea Urchin: friable spine that can require surgical debridement, often black/blue pigmenting
2. Treatment: hot water immersion, NSAIDS, possibly opioids, tetanus prophylaxis as indicated, antibiotic prophylaxis
3. Evacuation Guidelines: Wounds can become infected and require antibiotics – and sometimes surgical attention. Systemic symptoms should suggest need for immediate evacuation.

#### Trailside Talk – Hypoglycemia

Background:

You just finished a stretch of nights and decided to go for a walk in the woods before going home. You forgot to eat your post-shift snack and are now feeling a little out of sorts.

Case:

You are altered, but alert. The team will find you sitting on the side of the trail. You cannot give them any history. The key to this case is for them to use clues (medications on your person or medic alert bracelet) to realize you’re a diabetic and likely hypoglycemic.

- Primary Survey:
  ○ AVPU: Alert, profoundly confused
  ○ MARCH:
    ▪ M – No evidence of massive hemorrhage
    ▪ A – Airway intact
    ▪ R – Respiration normal
    ▪ C – Pulses intact
    ▪ H – No evidence of hyper/hypothermia
      - Hike vs helicopter
- Secondary Survey:
  ○ S – “Hi, how are you? I’ve lost my wallet inside that opossum (point to something that isn’t an opossum – or any variation of confusion you choose).
  ○ A – None
  ○ M – Insulin
  ○ P – Migraine
  ○ L – 12 hours ago
  ○ E –
    ▪ Unable to give any actual history
    ▪ After oral glucose: “I just finished working overnight. I needed some air so stopped at the park on the way home. I thought my sugar was getting low but thought I could make it back to my car.”
- Physical Exam:
  ○ General: Awake, confused
  ○ HEENT:
    ▪ Head: Atraumatic
    ▪ Eyes: Open, midline, ocular movements intact, pupils equal and reactive
    ▪ Ears: External normal
    ▪ Nose: Midline, no septal hematoma
    ▪ Oropharynx: Moist mucosa, no dental injuries or malocclusion
  ○ Neck:
    ▪ No crepitus, no palpable hematoma or lymph nodes, no palpable step-off
  ○ Chest:
    ▪ No external deformity, no crepitus
    ▪ Cardiovascular: Regular rate and rhythm, pulses bounding
    ▪ Respiratory: Breath sounds bilaterally, no respiratory distress
  ○ Abdomen/Pelvis:
    ▪ Non-tender in all four quadrants, non-distended
  ○ Extremities:
    ▪ Moves all extremities, no obvious trauma
    ▪ Pink diabetes bracelet on wrist
  ○ Neuro: Alert and oriented ×3, sensation intact
  ○ Skin: Moist skin, warm to touch

Critical Actions:

1. Use clues from your bag/wrist medic alert band to learn you’re a diabetic
  - At 5 minutes, if they haven’t figured it out, you can “accidentally” show them your bracelet.
2. Feed you
3. Repeat history and physical to rule-out any additional injuries (there aren’t any)
4. Dispo – explain their plan to keep you safe

Discussion:

1. Describe the approach to the Altered Patient.
2. Discuss clinical features of hypoglycemic patient.
3. Discuss management options for hypoglycemia.
4. Evacuation guidelines.

Discussion:

1. Describe the approach to the Altered Patient
  - This case is a reminder that common medical conditions still occur in the wilderness. While this algorithm is not specifically covered in this course, this is intended to be at the end of medical school. An often-used mnemonic is AEIOUTIPS.
    A: Alcohol/Acidosis
    E: Electrolytes
    I: Infection
    O: Opiates, Overdose
    U: Uremia
    T: Trauma
    I: Insulin
    P: Psychosis
    S: Stroke, Seizure
2. Discuss clinical features of the hypoglycemic patient.
  - Tremulousness, dizziness, diaphoresis, irritable, headache
  - Progressing to altered mental status, weakness, seizures, and coma
3. Discuss management options for hypoglycemia.
  - If possible, oral glucose – tablets or food. In the wilderness, this may be the fastest option.
  - Glucagon can be given IM. This may be carried on large expeditions or EMS may have it upon arrival.
  - IV dextrose – likely only on very large expeditions or once in hospital.
4. Evacuation guidelines
  - Evacuation may not be needed if it is quickly reversible and a clear cause is found, such as the patient forgetting to eat. If there is no clear cause, there are repeat episodes, or no response to field treatment, evacuation is needed.

#### Trailside Talk – Anaphylaxis

Background:

You just finished a 2-week course of an herbal cleanse that your naturopath suggested would cure you of your lifelong “Hymenoptera-histamine imbalance.” At their suggestion, you’ve decided to take a walk at a park to see if you can “have an encounter with our buzzing buddies.” Also, they forbade you to carry your “auto-injector of evil.”

Case:

You’ve just been stung by a bee. You don’t have an epi-pen and you’re feeling itchy, a little lightheaded, and your throat is tightening a bit

- Primary Survey:
  ○ AVPU: Alert and oriented
  ○ MARCH:
    ▪ M – No evidence of massive hemorrhage
    ▪ A – Airway intact
    ▪ R – Tachypneic, labored breathing
    ▪ C – Central pulses intact
    ▪ H – No evidence of hyper/hypothermia
      - Hike vs helicopter
- Secondary Survey:
  ○ S – “I just got stung by a bee. I mean, I was trying to, but I didn’t think I was allergic anymore. I’m feeling a little dizzy, and my throat is a little tight.”
  ○ A – None
  ○ M – None
  ○ P – Peanut and bee-sting allergy
  ○ L – 5 hours ago
  ○ E – “I was just trying to see if it worked…it didn’t. My doc just treated me for bee-sting allergy and I was trying to see if I’d still react. I don’t have my medicine. Oh, it’s getting hard to breath.”
- Physical Exam:
  ○ General: Awake, alert and oriented ×3. Breathing rapidly. Scratching.
  ○ HEENT:
    ▪ Head: Atraumatic
    ▪ Eyes: Open, midline, ocular movements intact, pupils equal and reactive
    ▪ Ears: External normal
    ▪ Nose: Midline, no septal hematoma
    ▪ Oropharynx: Moist mucosa, no dental injuries or malocclusion, tongue not swollen
  ○ Neck:
    ▪ No crepitus, no palpable lymph nodes, positive stridor
  ○ Chest:
    ▪ No external deformity, no crepitus
    ▪ Cardiovascular: Warm, well-perfused. Pulses bounding. Capillary refill <2 sec
    ▪ Respiratory: Tachypneic. Labored. Scant wheezing.
  ○ Abdomen/Pelvis:
    ▪ Non-tender in all four quadrants, non-distended
  ○ Extremities:
    ▪ Moves all extremities, no gross deformities
  ○ Neuro:
    ▪ Alert and oriented ×3, no motor or sensory deficits
  ○ Skin:
    ▪ Moist skin, urticaria

Critical Actions:

1. Epinephrine
  - Must give within first few minutes or get worse
  - If not given at 5 minutes – you get much worse and nearly unconscious
2. Adjuncts
  - Steroids
  - Histamine (H1 & H2) blockers
  - Albuterol
3. Dispo
  - You want to go home
    - They need to convince you to go to Emergency Department for monitoring in case you need repeat treatment
  - If they let you go, symptoms recur

Discussion:

1. Review pathophysiology of anaphylaxis
2. Discuss signs/symptoms
3. Discuss management

Discussion:

This is a case to remind students that common medical conditions still occur in the wilderness.

1. Review pathophysiology of anaphylaxis
  - Anaphylaxis is a distributive shock, with vasodilation and leaky vessels resulting from histamine due to mast cell degranulation
  - There are multiple criteria for diagnosis, but it can be simplified to exposure to suspected allergen and:
    - Involvement of two or more systems (cardiovascular, dermatologic, gastrointestinal, etc.)
    - Severe systemic collapse or airway compromise
2. Discuss signs/symptoms
  - Tachycardia, hypotension, urticaria, pruritis, oropharyngeal swelling, wheezing, emesis
3. Discuss management
  - Epinephrine is lifesaving for anaphylaxis. For true anaphylaxis, there are no contraindications to epinephrine. For adults, it should be given as 0.3mg (1:1000) intramuscularly in the anterolateral thigh. For pediatrics, 0.15mg is used in the autoinjectors. In the hospital, it is weight-based.
  - Adjuncts
    - H1 blockers
    - H2 blockers
    - Albuterol
    - IV fluids

#### Trailside Talks – Submersion Injury

Background:

You are hiking with friends when you notice a tire swing hanging enticingly above a swift moving river. Unfortunately, one of your more adventurous buddies is also one of your least buoyant friends, and his attempt at the tire swing ends in submersion. Your group pulls him to shore and he coughs and vomits for some time, but eventually calms and states that “he’s good.” You wonder, is he?

Case:

Your friend was submerged for about two minutes. He’s breathing, though coughing and vomiting, as you drag him from the water.

- Primary Survey:
  ○ AVPU: Alert
  ○ MARCH:
    ▪ M – No evidence of massive hemorrhage
    ▪ A – Airway intact
    ▪ R – Respiration – breathing and coughing
    ▪ C – Pulses intact
    ▪ H – No evidence of hyper/hypothermia
      - Hike vs helicopter
- Secondary Survey:
  ○ S – “(coughing throughout) I thought I was going to die. How long was I underwater?”
  ○ A – None
  ○ M – None
  ○ P – Appendectomy
  ○ L – 1 hour ago
  ○ E – “I was just trying to cool down. I saw this awesome looking tire swing and didn’t even think about how terrible a swimmer I am (coughing/vomiting).”
- Physical Exam:
  ○ General: On hands and knees, breathing but coughing.
  ○ HEENT:
    ▪ Head: Atraumatic
    ▪ Eyes: Open, midline, ocular movements intact, pupils equal and reactive
    ▪ Ears: External normal
    ▪ Nose: Midline, no septal hematoma
    ▪ Mouth: Moist mucosa, no dental injuries or malocclusion
  ○ Neck:
    ▪ Midline c-spine tenderness
    ▪ No crepitus, no palpable hematoma, no palpable step-off
  ○ Chest:
    ▪ No external deformity, no crepitus
    ▪ Cardiovascular: Tachycardic
  ○ Respiratory:
    ▪ Tachypnea, cough
  ○ Abdomen/Pelvis:
    ▪ Non-tender in all four quadrants, non-distended
  ○ Extremities:
    ▪ Moves all extremities. No obvious trauma
  ○ Neuro:
    ▪ Alert and oriented ×3, sensation intact
  ○ Skin:
    ▪ Moist skin, warm to touch

Critical Actions:

1. Safely remove patient from water – scene safety is a priority
2. C-spine immobilization
3. Evacuation

Discussion:

1. Discuss pathophysiology of drowning
2. Describe management

Discussion:

1. Discuss pathophysiology of drowning
  - Drowning terminology is often confusing. Recommendations now are to use fatal drowning and nonfatal drowning. No reference is made to dry or wet drowning. In addition, the type of water (salt vs fresh) is not clinically important. Perhaps aside from the Dead Sea, the amount of water typically aspirated is not enough to cause clinically significant electrolyte disturbances.
  - Upon submersion, victims typically hold their breath. Eventually the ventilatory drive is dominant and a breath is taken. Laryngospasm may or may not then occur. Typically, water aspiration leads to hypoxia, ventilation-perfusion mismatch, and decreased alveolar compliance due to surfactant washout. It is the hypoxia that ultimately causes the systemic effects.
2. Describe management
  - On-scene – Respirations are primary. As soon as possible and safe (potentially even while still in the water) rescue breathing should occur. If two breaths are not effective, CPR should be immediately initiated. Because it may complicate respiratory support, c-spine management is not required unless a clear concern exists due to mechanism or overt exam findings.
  - Hospital management – Rapid assessment and treatment of the ABCs as indicated, including positive pressure ventilation and potential intubation. If the patient looks well (normal oxygenation, no increased work of breathing, no systemic or other focal complications), no further testing is needed other than observation on pulse-oximetry for 8 hours. A chest x-ray should be obtained near the end of the observation period. If they remain asymptomatic and the x-ray is normal, they may be discharged. Prophylactic steroids and antibiotics are not recommended (unless clearly contaminated water).

#### Trailside Talks – Acute Mountain Sickness

Background:

You are hiking at Mt. Rainier when one of your classmates begins to complain of a headache and nausea. She looks clearly uncomfortable, clutching her head and begins vomiting. She thinks she just has a migraine. You wonder, is that all this is…?

Case:

Your friend develops headache, nausea and vomiting as you ascend the mountain.

- Primary Survey:
  ○ AVPU: Alert and oriented
  ○ MARCH:
    ▪ M – No evidence of massive hemorrhage
    ▪ A – Airway intact
    ▪ R – Respiration normal
    ▪ C – Pulses intact
    ▪ H – No evidence of hyper/hypothermia
      - Hike vs helicopter
- Secondary Survey:
  ○ S – “I’m sorry…My head is killing me. It feels like I’m getting a migraine (begins retching). I haven’t had a headache this bad since the last time I tried to summit Rainier.”
  ○ A – None
  ○ M – None
  ○ P – Migraine, no surgical history
  ○ L – 8 hours ago
  ○ E – “We’re just in town for a few days. We were hoping to summit the mountain this time. Last time I got a migraine and had to go down.”
- Physical Exam:
  ○ General: Tearful, clutching head and retching
  ○ HEENT:
    ▪ Head: Atraumatic
    ▪ Eyes: Open, midline, ocular movements intact, pupils equal and reactive
    ▪ Ears: External normal
    ▪ Nose: Midline, no septal hematoma
    ▪ Mouth: Mildly dry mucosa, no dental injuries or malocclusion
  ○ Neck:
    ▪ No crepitus, no palpable hematoma, no palpable step-off
  ○ Chest:
    ▪ No external deformity, no crepitus
    ▪ Cardiovascular: Regular rate, pulses bounding
    ▪ Respiratory: Breath sounds bilateral, no respiratory distress
  ○ Abdomen/Pelvis:
    ▪ Non-tender in all four quadrants, non-distended
  ○ Extremities:
    ▪ Moves all extremities. No obvious trauma.
  ○ Neuro: Alert and oriented ×3, moves all extremities, sensation intact
  ○ Skin: Moist skin, warm to touch

Critical Actions:

1. Cessation of ascent, consideration of descent
2. Assessment of neurologic examination

Discussion:

1. Review spectrum of altitude illness and sign/symptom
2. Discuss management
3. Discuss prevention strategies

Discussion:

1. Review spectrum of altitude illness and sign/symptom
  - HACE (high-altitude cerebral edema) and AMS (acute mountain sickness) are components of the same spectrum of disease. Cerebral edema is a factor in severe AMS and HACE, although less certain in mild AMS. The “tight fit” hypothesis is often invoked, suggesting that individuals have different amounts of ‘extra’ space in their cranial vault and are more or less susceptible to the effects of cerebral edema depending on this. VEGF (vascular endothelial growth factor), NO (nitric oxide), and cytokines are all thought to play a role in the increased vascular permeability and loss of autoregulation of intracranial pressure. AMS typically occurs over 2,000 meters, while HACE is less common below 3,000 meters. A history of AMS or HAPE (high-altitude pulmonary edema) is more likely to develop HACE.
  - Acute mountain sickness – headache, fatigue, nausea, emesis, anorexia, disturbed sleep.
  - High-altitude cerebral edema – ataxia, confusion, drowsiness, coma
    - May present with HAPE. If high-altitude pulmonary edema (HAPE) present, HACE can present more rapidly.
  - High-altitude pulmonary edema – initial dry cough, dyspnea on exertion. Progresses to dyspnea at rest and cough productive of pink, frothy sputum or overt hemoptysis. Symptoms often start 2–4 days after arriving at elevation but can progress rapidly once they start. Progression is often noted to happen at night.
2. Discuss management
  - AMS – Improves after 1 day with no further ascent or faster with descent (500–1000 meters). Severe AMS may require supplemental oxygen and descent. No further ascent until symptoms resolved. Treat symptoms with acetaminophen, ondansetron, and ibuprofen as needed. Acetazolamide is preferred over sleep aids for disturbed sleep. Dexamethasone may improve symptoms, but does not make acclimatization more rapid. Acetazolamide is favored for this reason.
  - HACE - The primary treatment of HACE is immediate descent at first signs/symptoms. Other medications and treatments should not delay descent. Dexamethasone is very helpful, given immediately and every 6 hours until descent of at least 1000 meters. Even if symptoms improve on dexamethasone, patient must descend. Remember, it does not speed up acclimatization. Supplemental oxygen as needed for oxygen saturation over 90% and portable hyperbaric chambers are helpful, but should not delay descent.
3. Discuss prevention strategies
  - Primary prevention for AMS is slow ascent. If moderate to high risk or rapid ascent, acetazolamide is preferred over dexamethasone. Dexamethasone is helpful for rapid ascent on short notice and short duration at altitude (rescue crews). Acetazolamide is often recommended at 125mg twice a day orally starting the day prior to ascent and stopping when maximum altitude is achieved. Ibuprofen and aspirin may have a role in preventing AMS.

### Appendix M Learner Assessment

This course is Pass/Fail, although the assessment format can be adapted for a grade for a particular institution. Students will be assessed by an equal split of participation, a final capstone presentation, and a final written examination. To pass the course, the student must pass each individual component.

**Participation:** The faculty will determine whether the student arrived to each session prepared, demonstrating knowledge of the key concepts from the assigned reading, and whether they were consistently engaged in the session activities. At the end of the first week, faculty will notify students of their current standing in order to allow the student sufficient time for corrective action.

**Presentation:** Each student will be given 10 minutes on the final trip to present a plant of toxicologic significance. Selection of the plant is left to the student. Minimum topics to be covered are medicinal history, mechanism of toxicity, and toxicologic management. Use of audiovisual equipment will not be available.

#### Presentation Rubric

**Medicinal History:** Discusses medicinal history of this plant and why they feel it is significant[Table t11-jetem-5-1-c1]

**Table t11-jetem-5-1-c1:**

Unsatisfactory	Satisfactory	Exceptional
1 point	2 points	3 points

**Mechanism of Toxicity:** Demonstrates understanding of mechanism and explains clearly[Table t12-jetem-5-1-c1]

**Table t12-jetem-5-1-c1:**

Unsatisfactory	Satisfactory	Exceptional
1 point	2 points	3 points

**Toxicologic Management:** Provides clear distillation of core management principles[Table t13-jetem-5-1-c1]

**Table t13-jetem-5-1-c1:**

Unsatisfactory	Satisfactory	Exceptional
1 point	2 points	3 points

**General:** Presentation Quality, Organization, Style and Clarity[Table t14-jetem-5-1-c1][Table t15-jetem-5-1-c1]

**Table t14-jetem-5-1-c1:**

Unsatisfactory	Satisfactory	Exceptional
1 point	2 points	3 points

**Table t15-jetem-5-1-c1:**

**Total Score:** _____/12	Minimum 8/12 to achieve score of passing

**Final Examination:** Students will be given a final written examination designed to test both basic knowledge and integration of principles through discrete and case-based questions. (Please see exam on next page). Each of the 10 questions is worth 1 point, and each essay is worth 5 points for a total of 20 available points. A passing score is 14 points or higher.

### Appendix N Final Examination

1. The park medic rangers in Denali National Park are concerned that they don’t have a standing protocol for self-administration of medications for the treatment/prevention of AMS given that they routinely perform rescues in excess of 15,000 ft. Which of the following is the most reasonable addition to their protocols?
  - Acetazolamide 250mg PO BID for 3 days in advance to a high-altitude rescue
  - **Dexamethasone 4 mg PO QID at the beginning of and while participating in a high-altitude rescue**
  - Dexamethasone 10 mg IV ×1 while en-route to a high-altitude rescue
  - Gingko-Biloba 80mg PO BID at the beginning of and while participating in a high-altitude rescue
2. A 37-year-old male sees a shark while participating in a scuba-diving class. He is frightened and quickly ascends to the surface. On the surface he immediately develops left arm paralysis. He appears anxious and is not speaking clearly. What is the definitive care for this patient?
  - Assist him to quickly dive back down to his original depth, then slow ascent
  - **Immediate transport for recompression at a hyperbaric facility**
  - Immediate transport to the nearest emergency department for tPA
  - Provide 100% oxygen and allow him to calm down
3. As medical director for an experiential education program you are called for advice on a group leader’s satellite phone. After having gradually ascended to 12,000 feet, the group became snowbound for 7 days and was limited to their tents and igloos for all activities. The team leader notes that one group’s tent seems to have contracted flu-like symptoms (headaches and nausea only), and he is concerned that all three campers have developed acute mountain sickness. He requests permission to cancel the trip and descend immediately to ease the patients’ symptoms. Which of the following pieces of additional information would cause you to consider helicopter evacuation for one or all of the patients?
  - Continued severe snowfall with whiteout conditions preventing a safe hiking descent
  - **One of the patients develops rapid onset of cough, shortness of breath, and rales**
  - One of the patients has a prior history of asthma
  - The tent group realizes they have been melting snow for drinking water from near the camp latrine
4. A hypothermic victim arrives to your ski-slope clinic in cardiac arrest. He was found face down in a tree well on the final run of the day by the ski patrol. The medics suspect that he likely asphyxiated prior to going into arrest. Termination of resuscitation could be considered with which of the following additional pieces of information?
  - Intermittent CPR was performed on the patient given the difficulty of evacuating him down the ski-slope
  - It is an elderly gentleman with a known thyroid disorder, and his heart rhythm alternates between Afib and Vfib despite multiple attempts at defibrillation
  - **The iStat chemistry analyzer reports the patient’s potassium level to be 15 mg/dL**
  - The patient has a suspected c-spine injury and body temperature of 15 degrees Celsius
5. On a camping trip in North Carolina, one of your friends was out scouting for firewood when he was bitten by a snake on his calf. Your friend thinks it was a corn snake. He states it feels better with pressure, so he wants to put a tight bandage on it and keep looking for firewood. Looking at the bite site, you notice a 2 cm area of redness and swelling and quickly draw a circle around it. You are 3 miles from your cars. What are the next best steps in management for this bite from the pictured snake?[Fig f6-jetem-5-1-c1]
  Maggs M. Untitled. In: Pixabay. https://pixabay.com/photos/snake-rattlesnake-rattle-desert-2799910/
  - Hike back to your cars, place ice on the bite, and go directly to the ED and administer 2 vials of CroFab
  - Immobilize his leg, call for a helicopter to get to the ED, administer 6 vials of CroFab
  - **Immobilize his leg, carry him back to the cars, go directly to the ED, administer 4 vials of CroFab only if the swelling is rapidly spreading**
  - Keep setting up camp and go to the ED only if the swelling spreads
  - Put a tourniquet on his leg, carry him back to the cars, go directly to the ED and administer 4 vials of CroFab only if the swelling is rapidly spreading
6. You see a 48-year-old female in the ED who presents with vomiting and diarrhea. She awoke with these symptoms at 8am this morning and despite trying to wait it out at home, she is now in your ED at 12pm. Yesterday she ate a salad and soup of vegetables from her garden for dinner at 7pm. On further questioning she admits to foraging for mushrooms yesterday as well, something she does every year with her grandmother and includes in salads. What are your next steps in management?
  - In addition to symptomatic treatment, she should have her electrolytes, creatinine, and liver function evaluated. If they are normal and she feels better, she can be discharged home.
  - **In addition to symptomatic treatment, she should have her electrolytes, creatinine, and liver function evaluated. She should be admitted to the hospital for monitoring, support, and trending of the above labs.**
  - In addition to symptomatic treatment, she should have her electrolytes, creatinine, and liver function evaluated. She should be placed on the liver transplant list.
  - She should get symptomatic treatment with ondansetron and IV fluids. If she feels better in 2 hours, she can be discharged home.
  - She should get symptomatic treatment with ondansetron and IV fluids. If she feels better in 6 hours, she can be discharged home.
7. A 23 year-old-female white-water kayaking flipped her kayak and fell into the water. She managed to climb onto a rock in the middle of the river. A bystander witnessed the event and threw a rope to her on the rock. When she grabs the rope and is pulled through the water, she gets pushed downstream and underwater. When she is pulled back to shore, she is coughing and is unable to talk due to shortness of breath. She was back in the water holding the rope for less than 1 minute. EMS transports her to the hospital, where you are her physician. She is now comfortable and talking easily. Her vitals are: P 98, RR 21; T 37.4°C; O2 sat 97%; BP 129/68. What is the next step in management?
  - Admit to the hospital as she is likely to decompensate within 24 hours.
  - Immediately obtain an x-ray, then observe for 8 hours in the Emergency Department. If all normal, discharge
  - Observe her for 2 hours in the Emergency Department. If no symptoms develop, discharge.
  - **Observe her for 8 hours in the Emergency Department with x-ray near the end of her course. If all normal, discharge.**
8. At the end of your shift in your rural ED, a 16-year-old male is brought in by ambulance with syncope. You note on exam that he is very bradycardic and order an ECG which reveals that he is in 3^rd^ degree heart block. Temp 37.8°C, Pulse 37, BP 93/48, RR 19, O2 Sat 95% on room air. Which of the following would decrease the likelihood of Lyme carditis?
  - He has a history of marijuana use
  - He reports never having had a rash
  - He spent the entire summer hiking and camping
  - **Ticks were always removed and were never attached for more than 24 hours**
9. The local school board asks for your expert opinion on a high-yield cost-effective strategy to prevent heat exhaustion and heat stroke among high-school athletes. In choosing one option among their expense proposals you recommend:
  - A full-time team physician for school physicals to screen for genetic susceptibility to exertional rhabdomyolysis
  - **Mandatory water/rest/shade breaks every half-hour**
  - Medic standby for all games/practices with ice water baths for rapid cooling of heat related injuries
  - Prophylactic acetaminophen 650 mg PO q4 hours as an antipyretic on heat-warning days
10. On your shift at a community ED in Fort Lauderdale, FL a patient notes to you generalized body aches, itching, irritability, headache and a dizziness sensation. Having taken a wilderness medicine course in med-school you ask about recent recreational activities and realize that the patient’s symptoms are related to which gas coming out of solution?
  - Helium
  - Hydrogen
  - **Nitrogen**
  - Oxygen

**Essays:**

1. Hypothetical: With the increased popularity of bouldering in remote locations, you are approached by an EMS agency to develop a protocol for spinal clearance in prolonged extrication. They routinely have evacuation times over 12 hours and helicopters are not feasible in their local terrain. The goal of the protocol is to increase the ability of the patient to help self-extricate, while still minimizing the chance of moving a patient with a potentially unstable c-spine injury. Please prepare a brief protocol for that keeps these opposing goals in mind.
2. As director of your local West Virginia Search and Rescue team, you are called at 10pm Monday evening to help coordinate a search and rescue for a 19-year-old male who was hiking alone in the Dolly Sods Wilderness when he was reported missing. According to his mother, his plan was to hike the Blackbird Knob and Red Creek Trails, but he often changed plans and enjoyed getting off trail. He was supposed to return Sunday evening, but she has not heard from him since. His car was found at the eastern Blackbird Knob trailhead.
  Create a search and rescue plan for this incident. In addition to including such items as number of people, supplies needed, and communications, please detail how you would split search areas between personnel and how you would systematically search each area in a way that optimizes your chances of finding him early. Explain your rationale.

### Appendix O Program Evaluation

In order to ensure the objectives of the course and individual sessions are met, the course will be refined through multiple sources of information. As with any new product, this course will benefit from iterative changes based upon performance data.

1. Learner feedback. At the end of each week, a survey will be sent to students for anonymous feedback. Students will be asked about the content and delivery methods for each session. The second week, students will also be asked about the sessions of that week as well as for their perceptions of the course as a whole.
2. Faculty perceptions. After educational sessions, there are often potential improvements recognized that are not memorialized, thus forgotten for future sessions. At the end of each session of this course, faculty will fill out a form to capture areas for improvement and areas that went well. In addition to being used as a primary source for program improvement, this will also be compared with student responses on individual sessions to look for and understand any discrepancies.
3. Student performance data. Student performance on the final examination will be reviewed. For the inaugural year, the goal is for students to achieve a minimum of 14 out of 20 points. In addition to evaluating the appropriateness of this cut-off, student performance on individual questions will be reviewed to identify questions needing improvement, and more importantly, areas of the course that may need further development.

### Appendix P Marine Envenomations

Please see associated lecture

### Appendix Q Program Evaluation

Please see associated lecture

### Appendix R Decompression Illness

Please see associated lecture

### Appendix S Program Evaluation

Please see associated lecture
