# Supplementary material for: Introduction to Wilderness Medicine—A Medical School Elective
Source: J Educ Teach Emerg Med. 2020 Jan 15;5(1):C1–C120. doi: 10.21980/J8B93X (PMC10332540; doi:10.21980/J8B93X)
Supplement: Supplementary file 1 — Please see associated lecture [file jetem-5-1-c1-appendixp.pptx]

## Slide 1
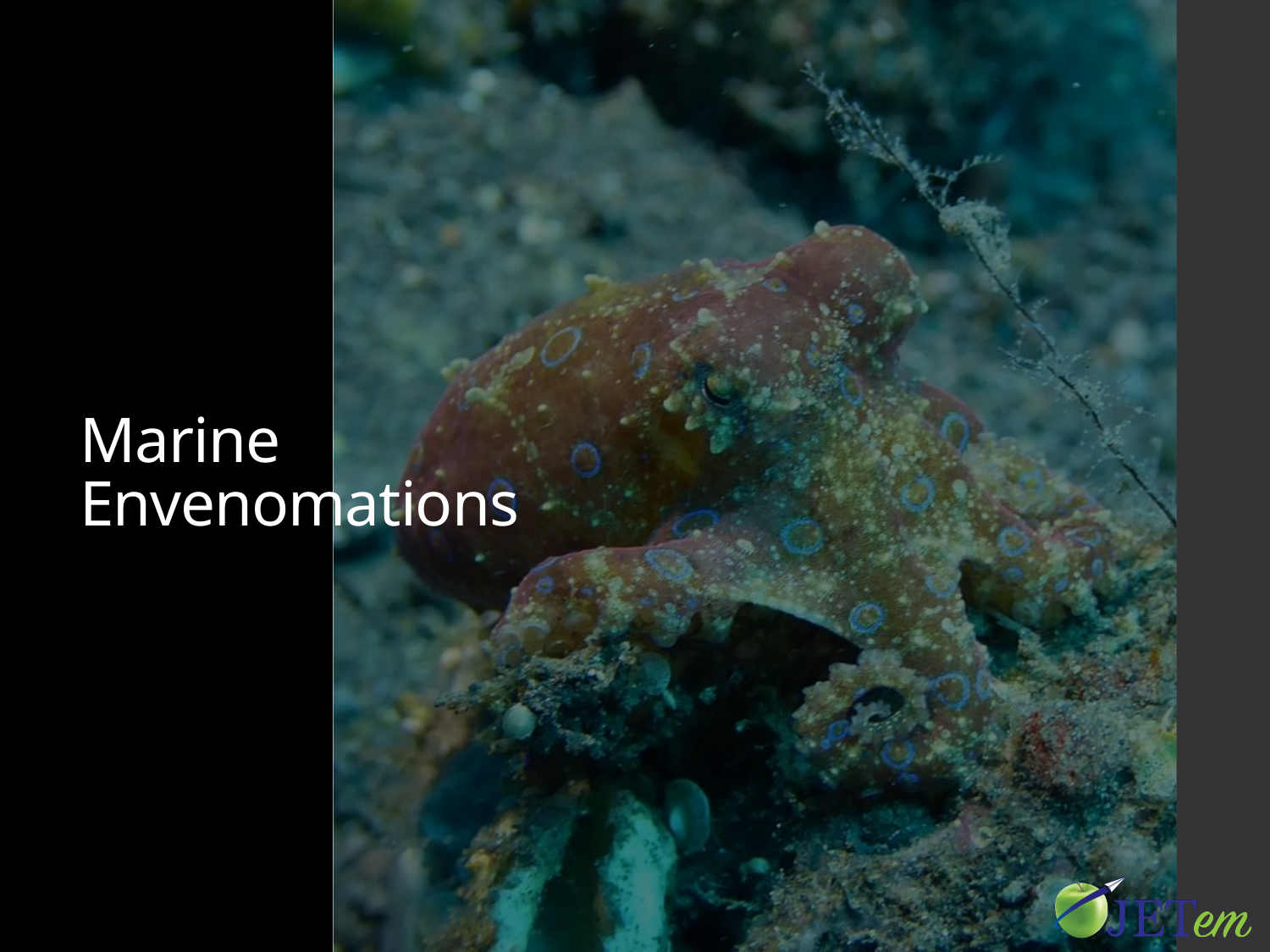

# Marine Envenomations

## Slide 2
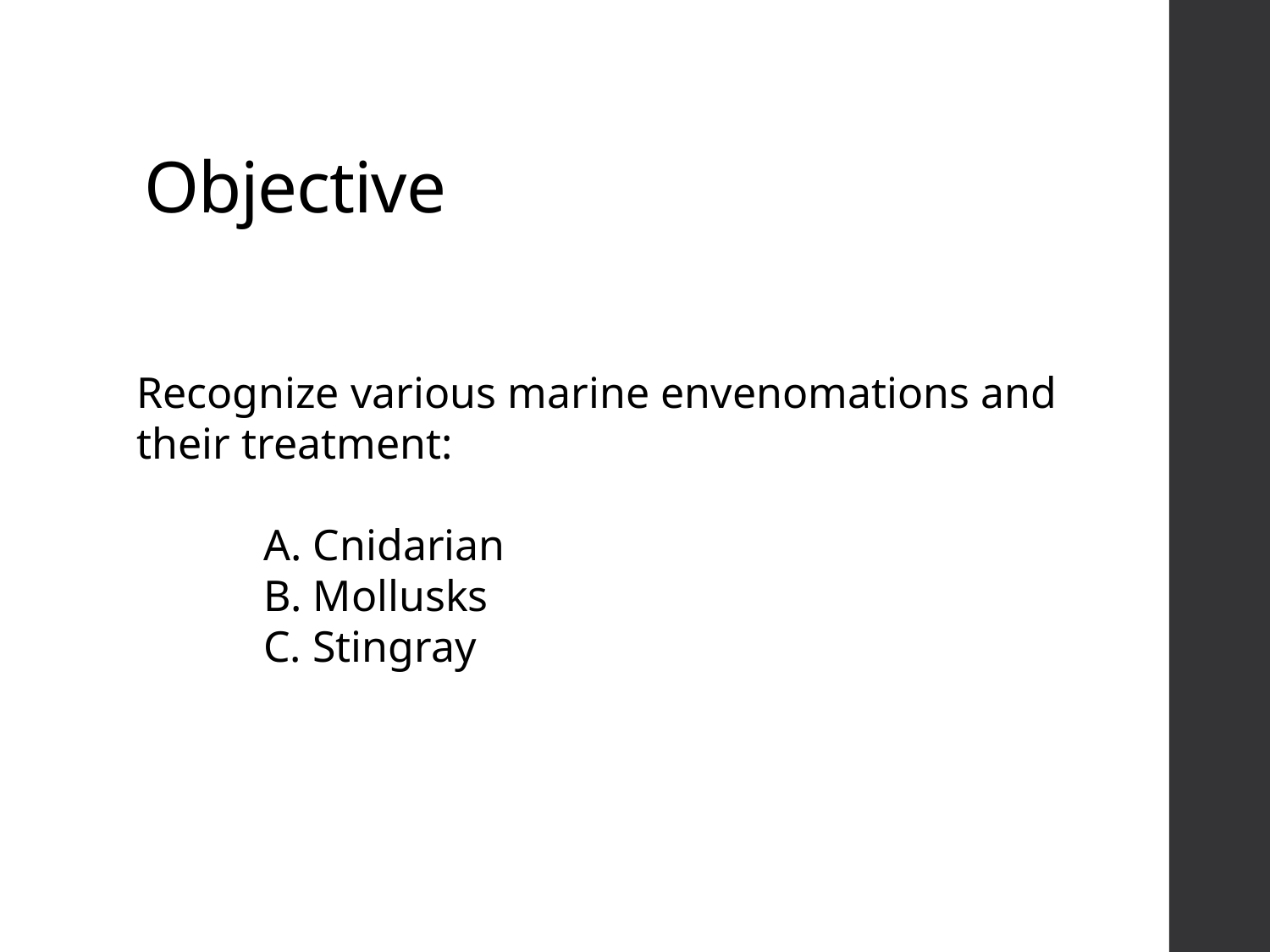

# Objective
Recognize various marine envenomations and
their treatment:
	A. Cnidarian
	B. Mollusks
	C. Stingray

## Slide 3
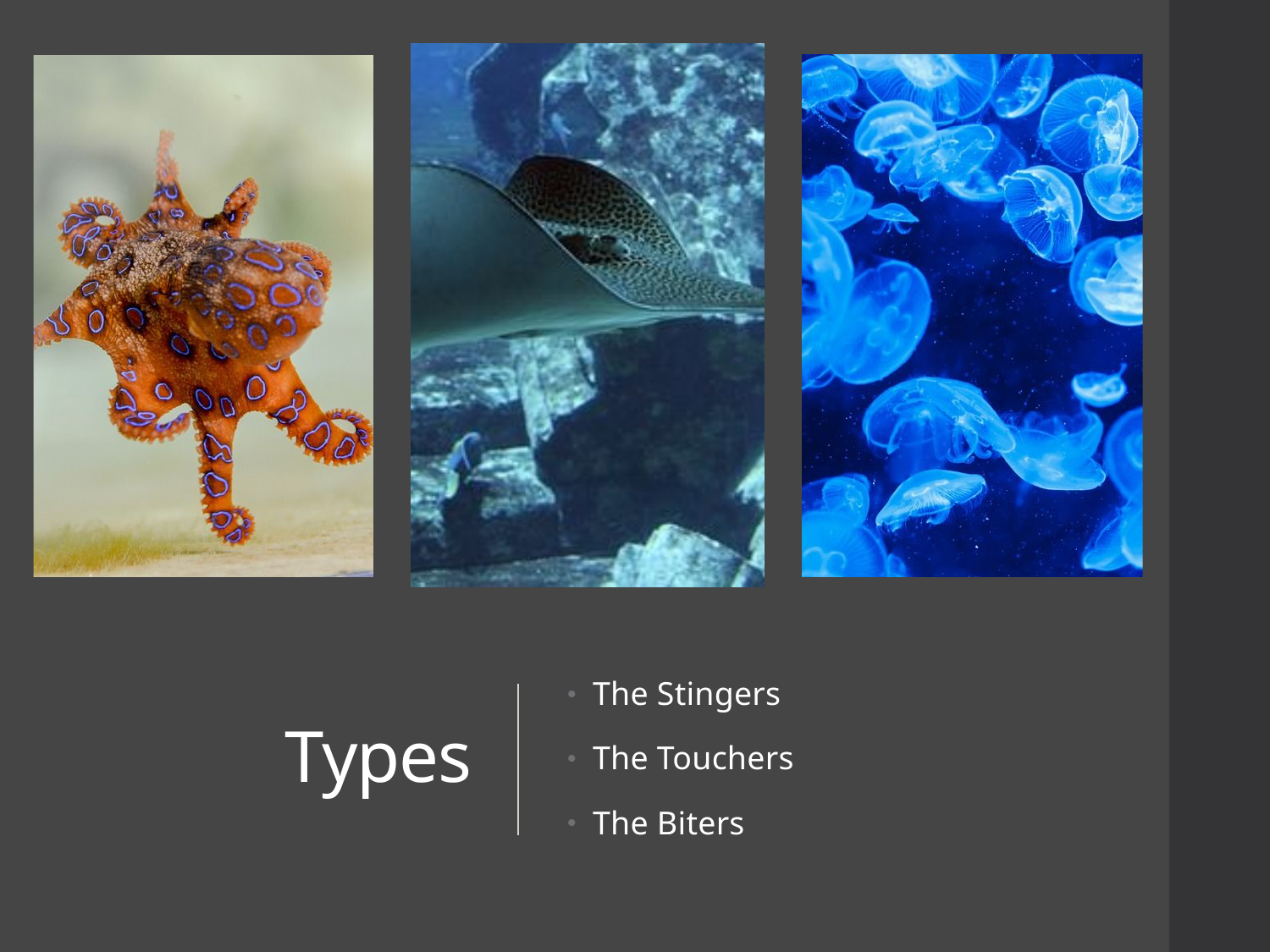

# Types
The Stingers
The Touchers
The Biters

## Slide 4
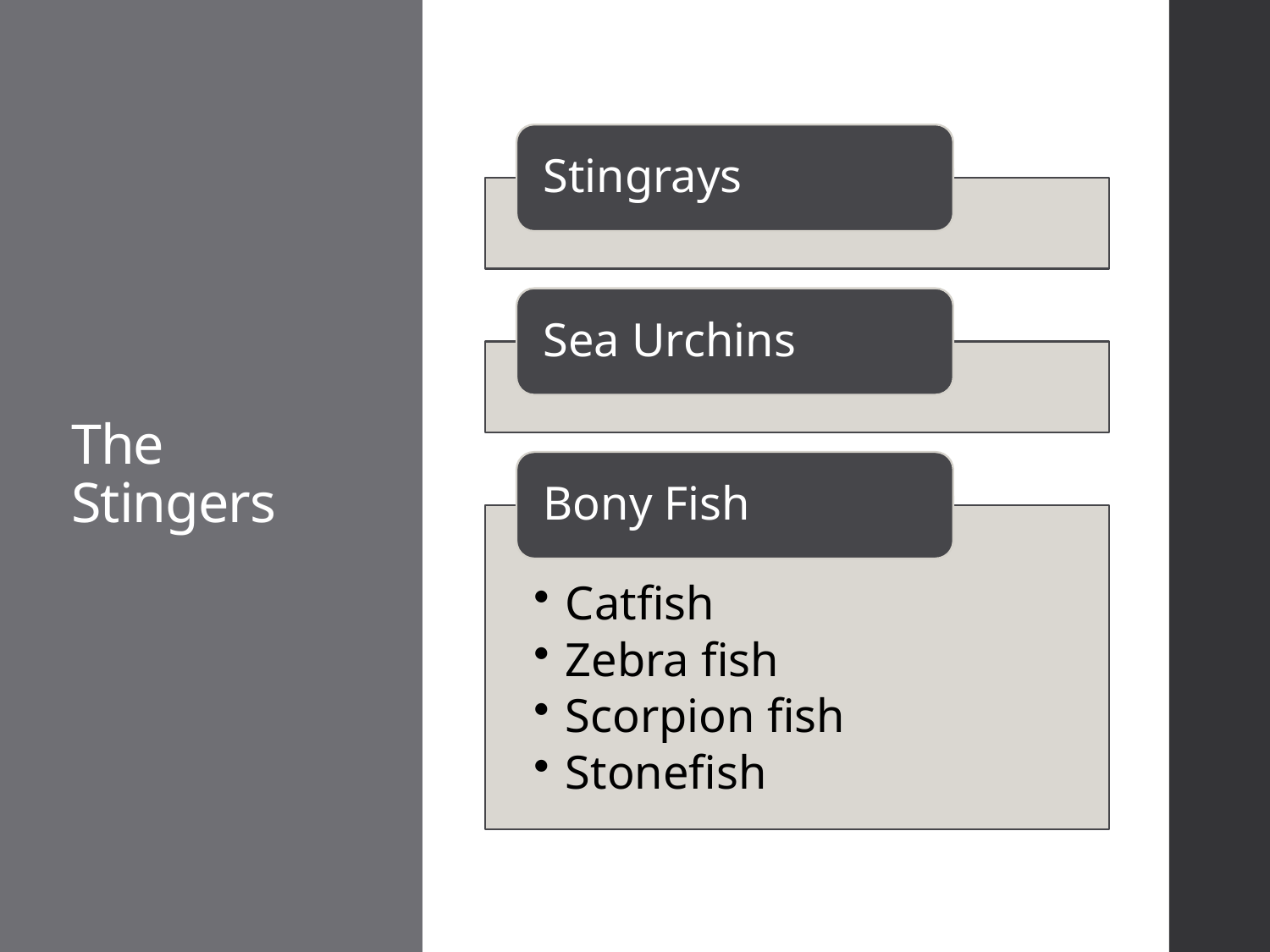

# The Stingers

## Slide 5
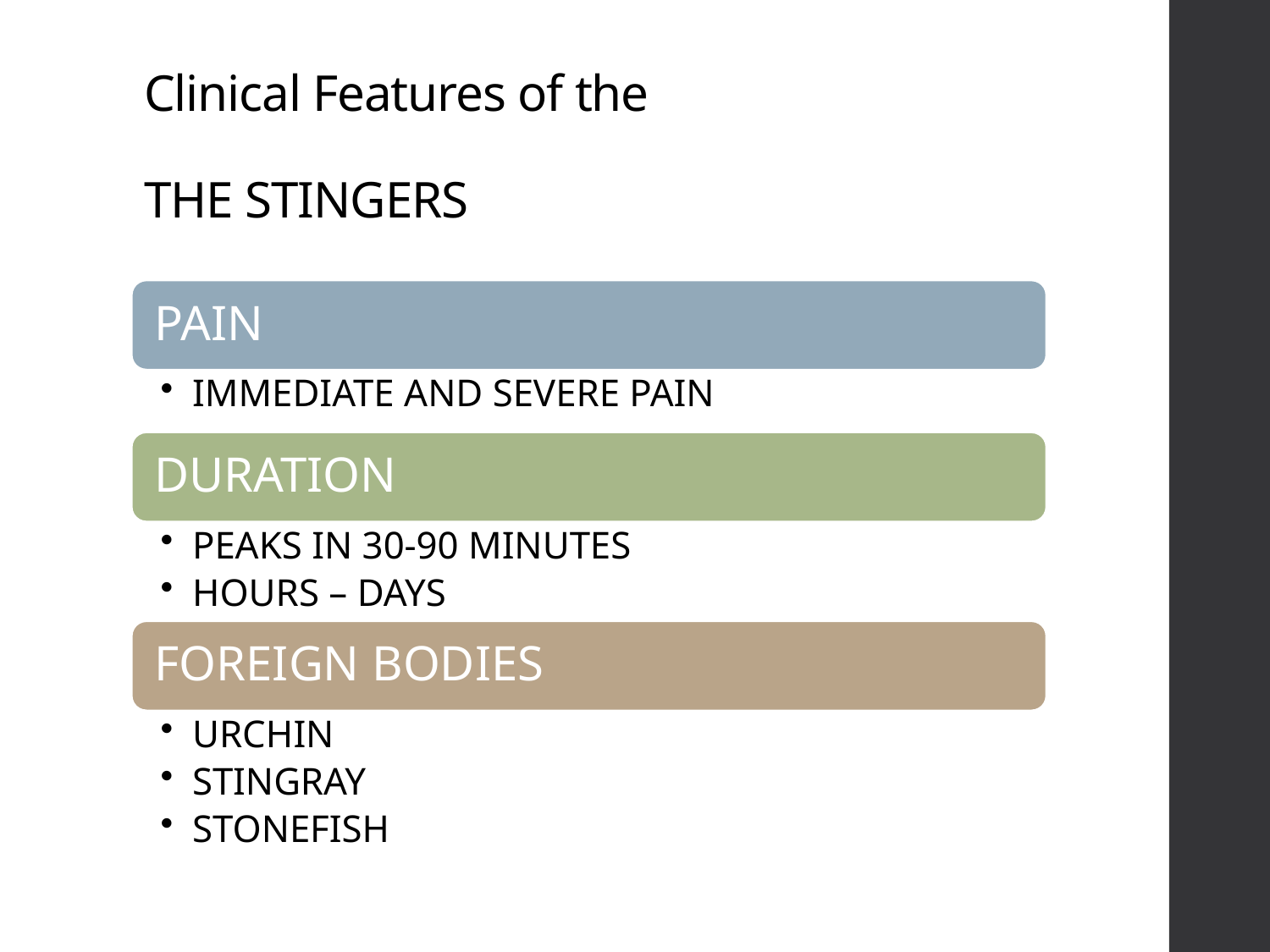

# Clinical Features of the THE STINGERS

## Slide 6
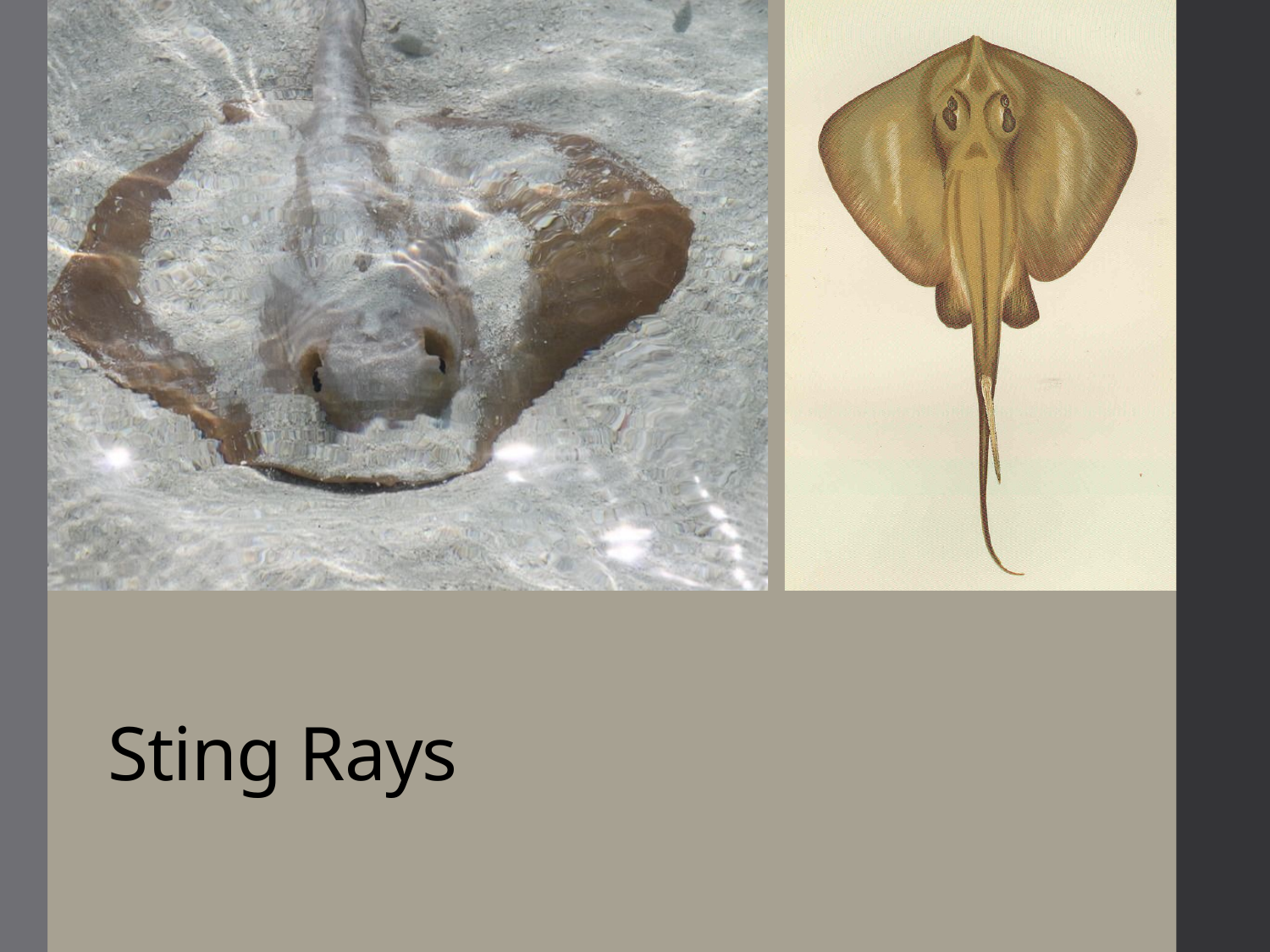

# Sting Rays

## Slide 7
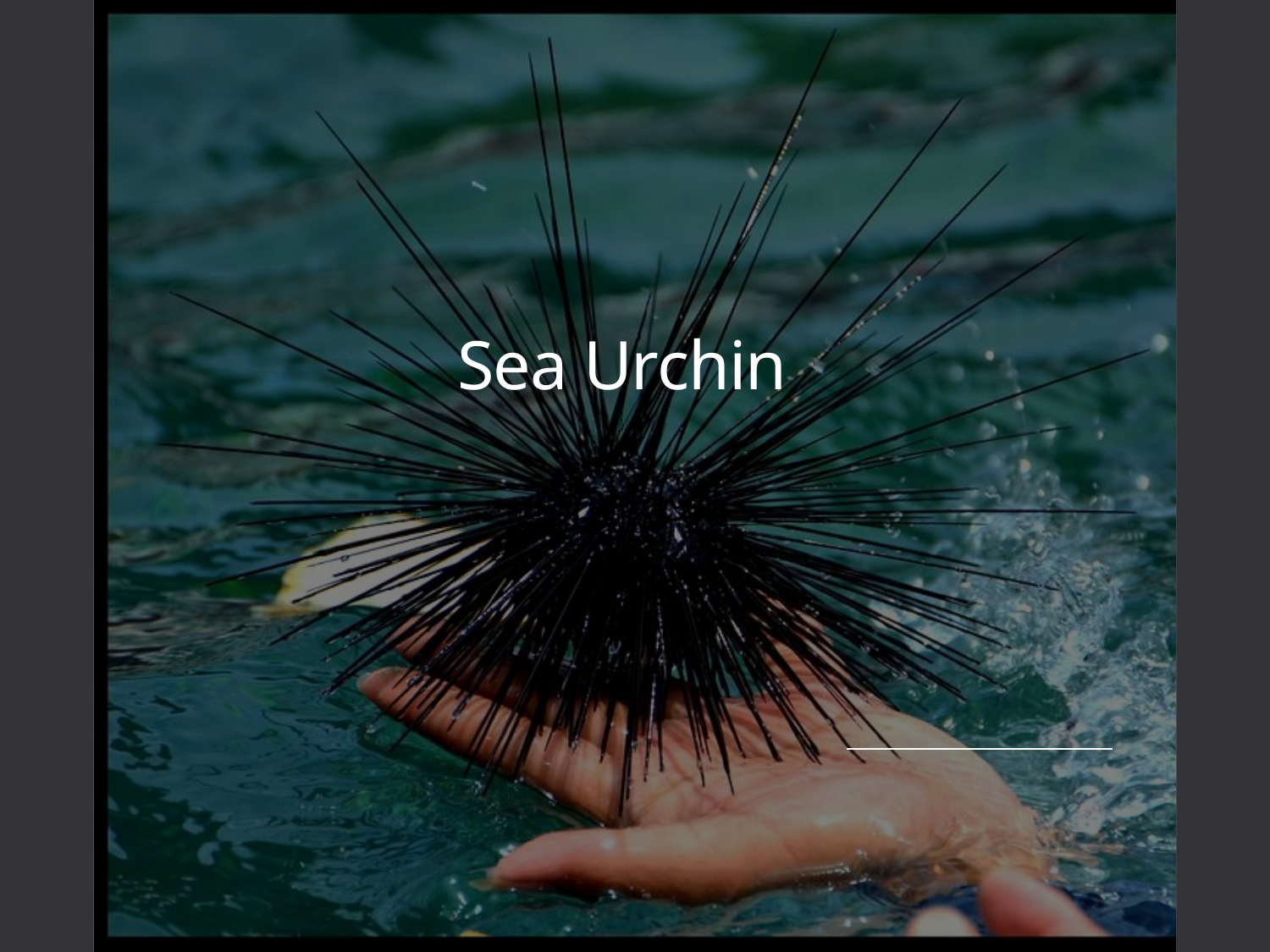

# Sea Urchin

## Slide 8
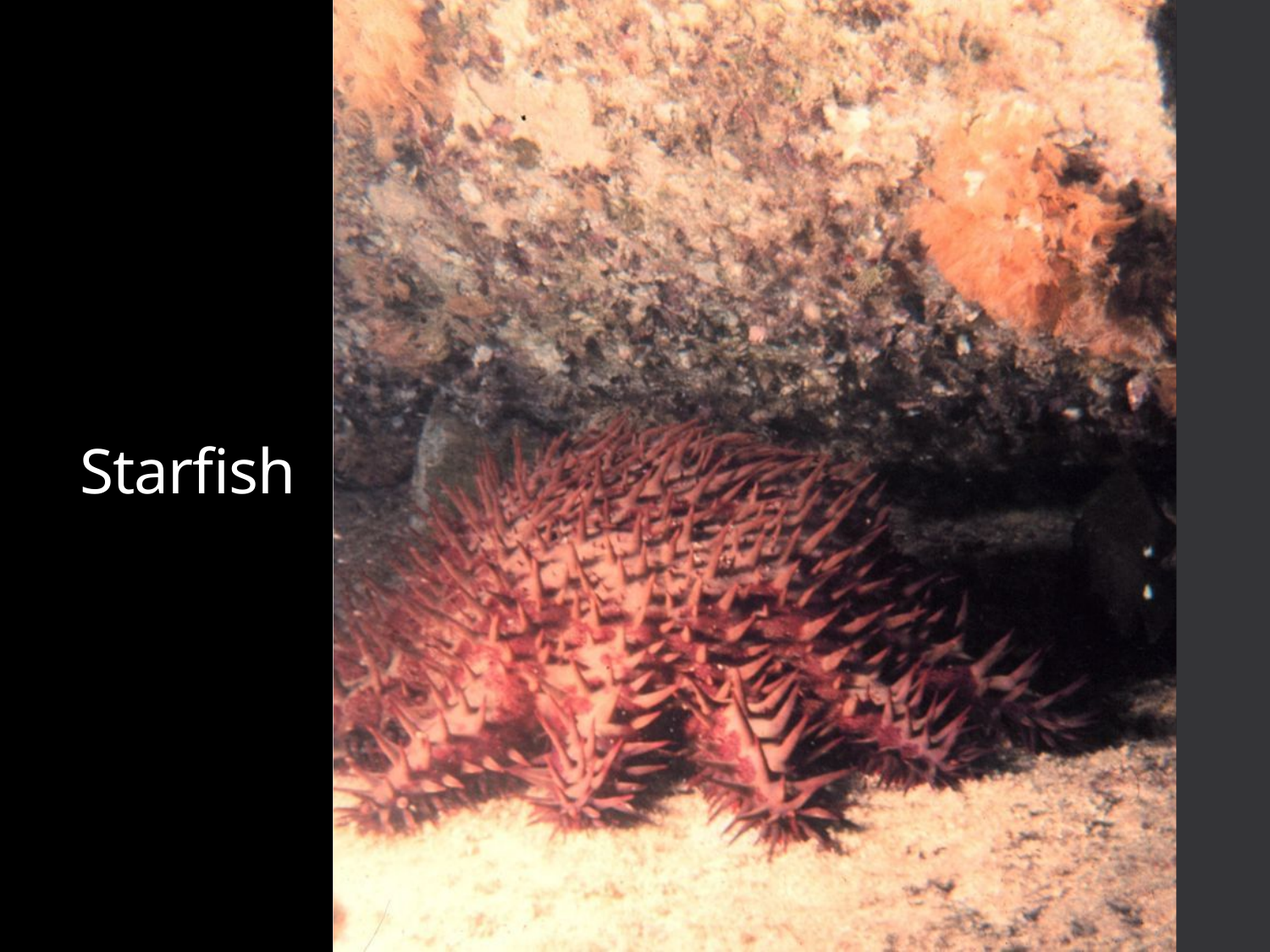

# Starfish

## Slide 9
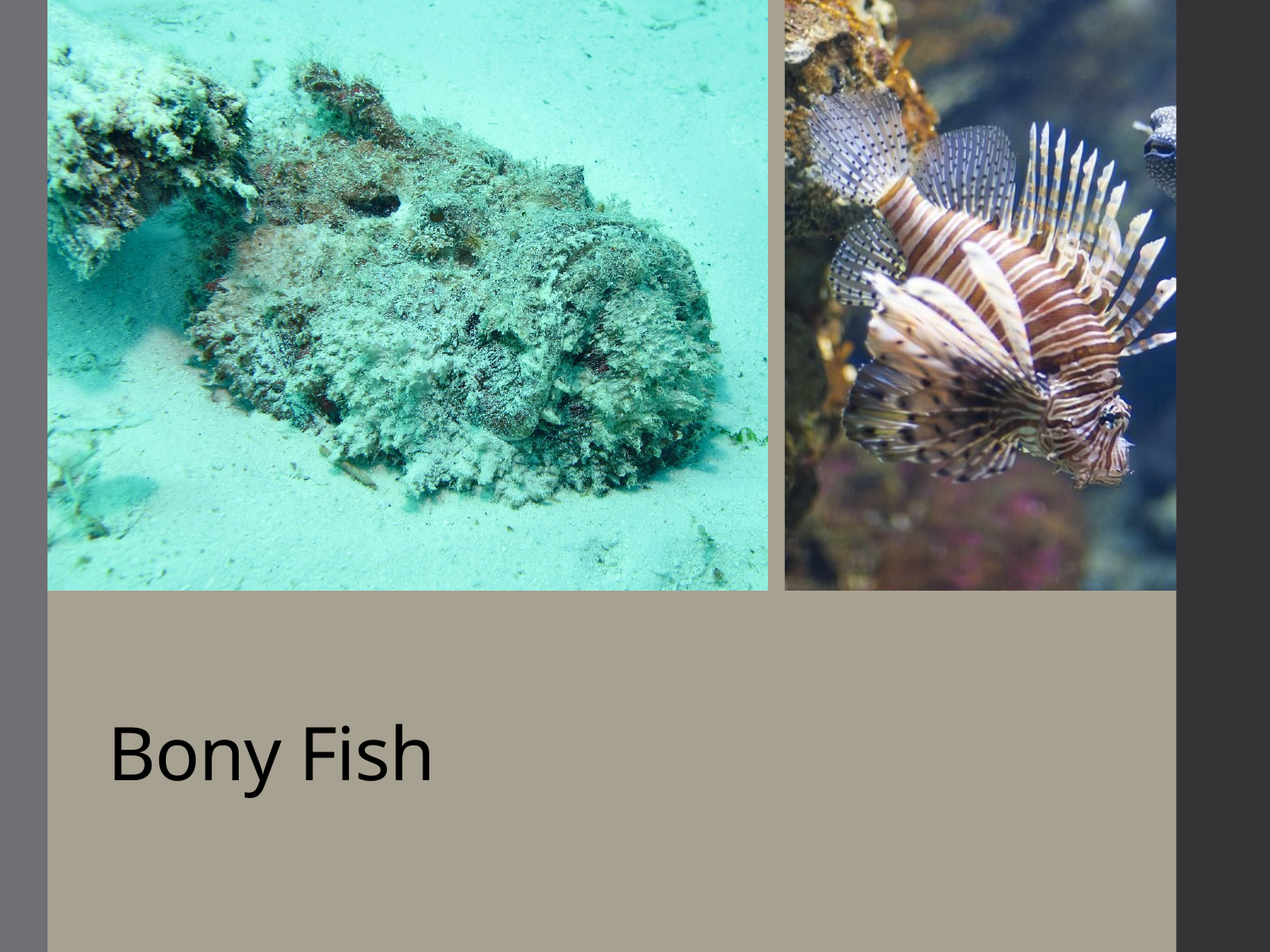

Bony Fish

## Slide 10
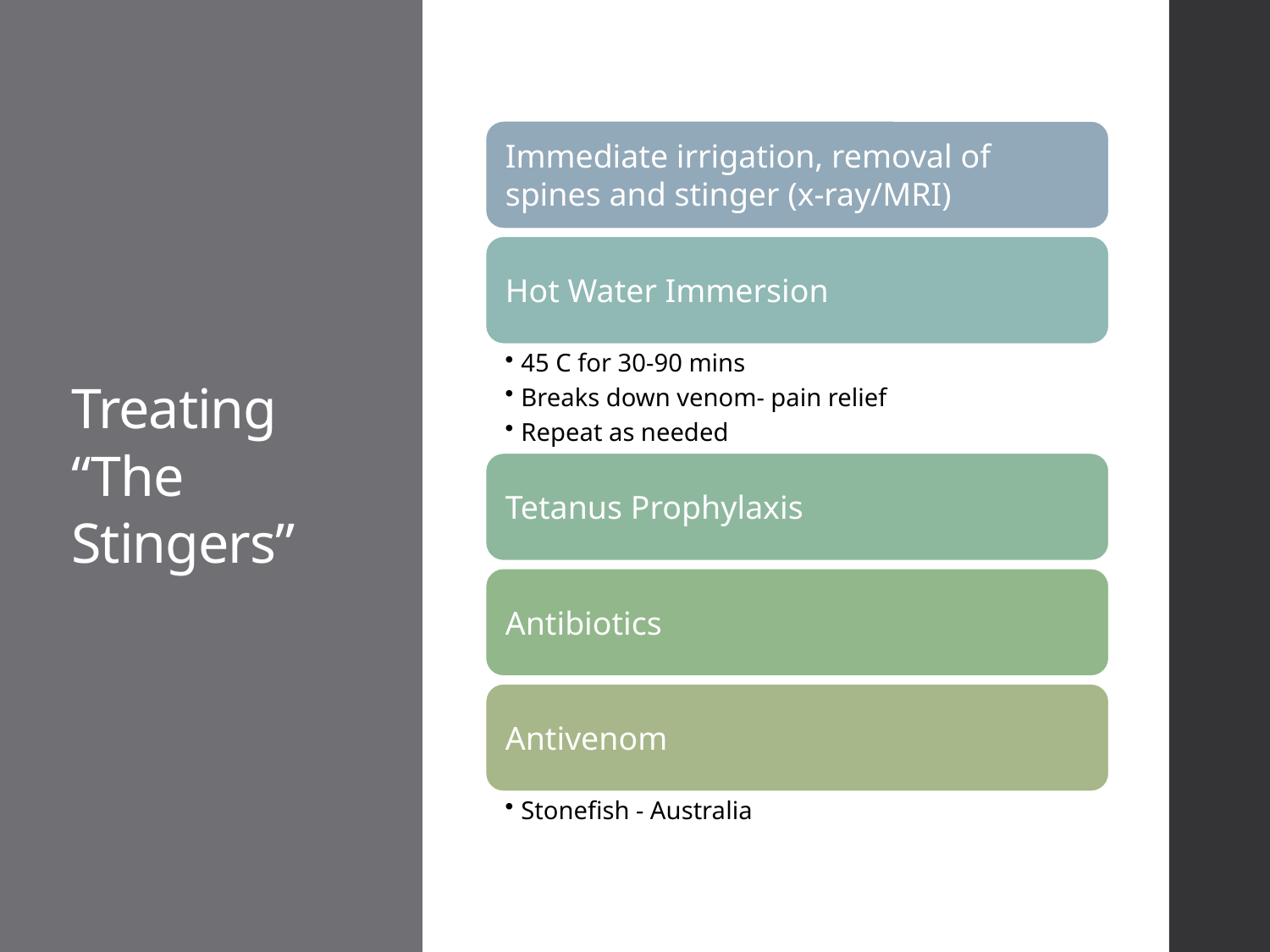

# Treating “The Stingers”

## Slide 11
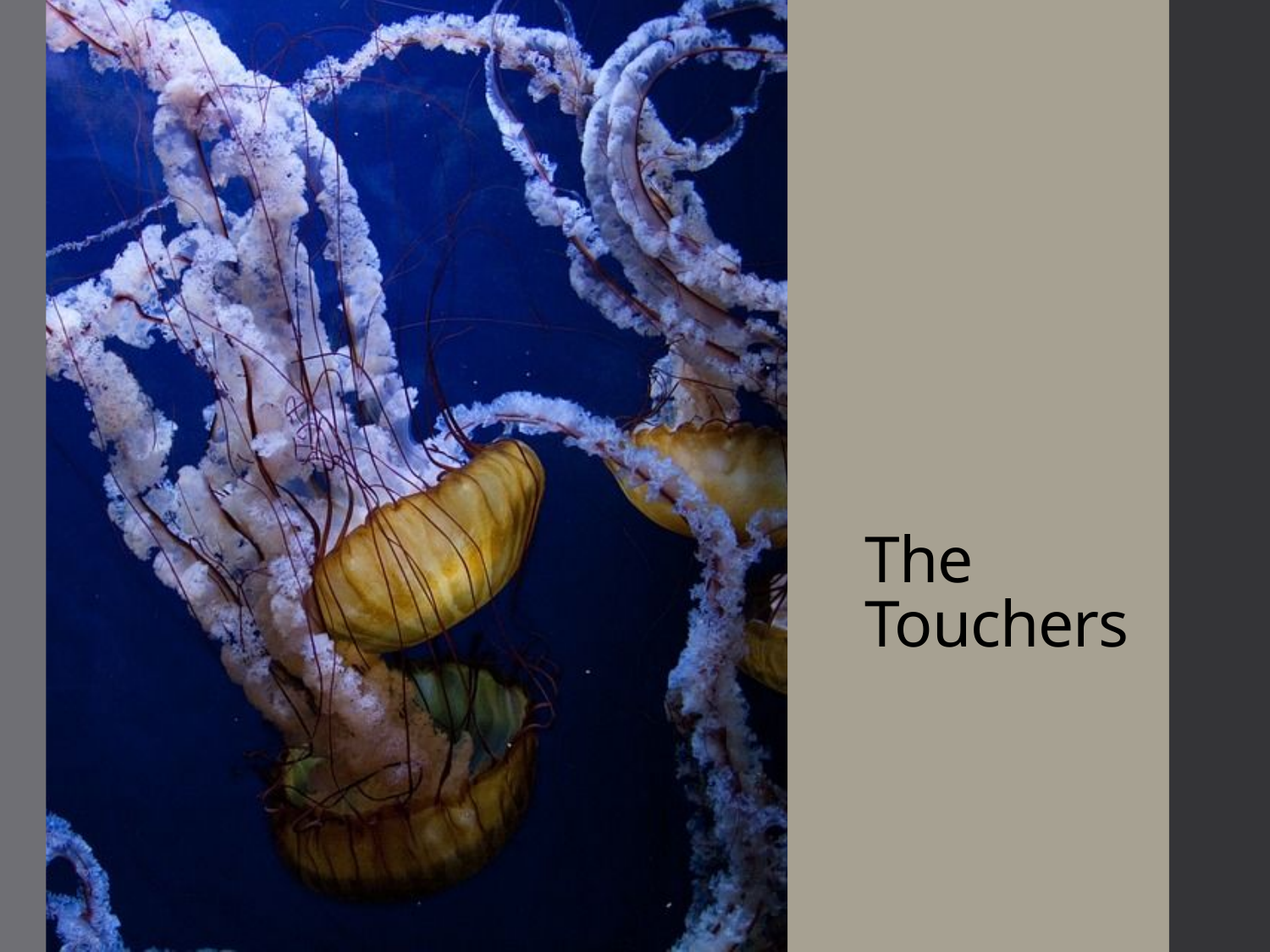

# The Touchers

## Slide 12
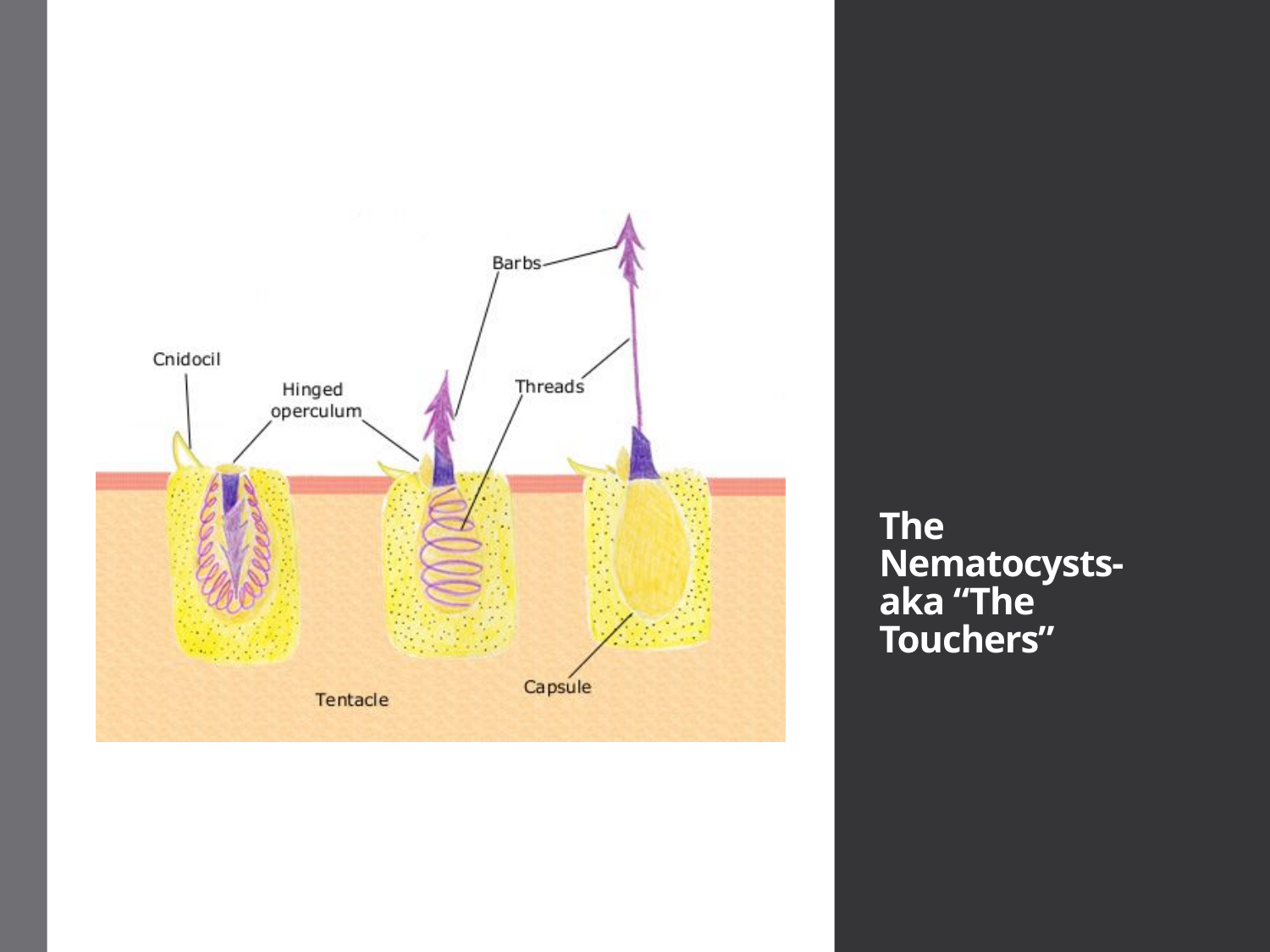

# The Nematocysts- aka “The Touchers”

## Slide 13
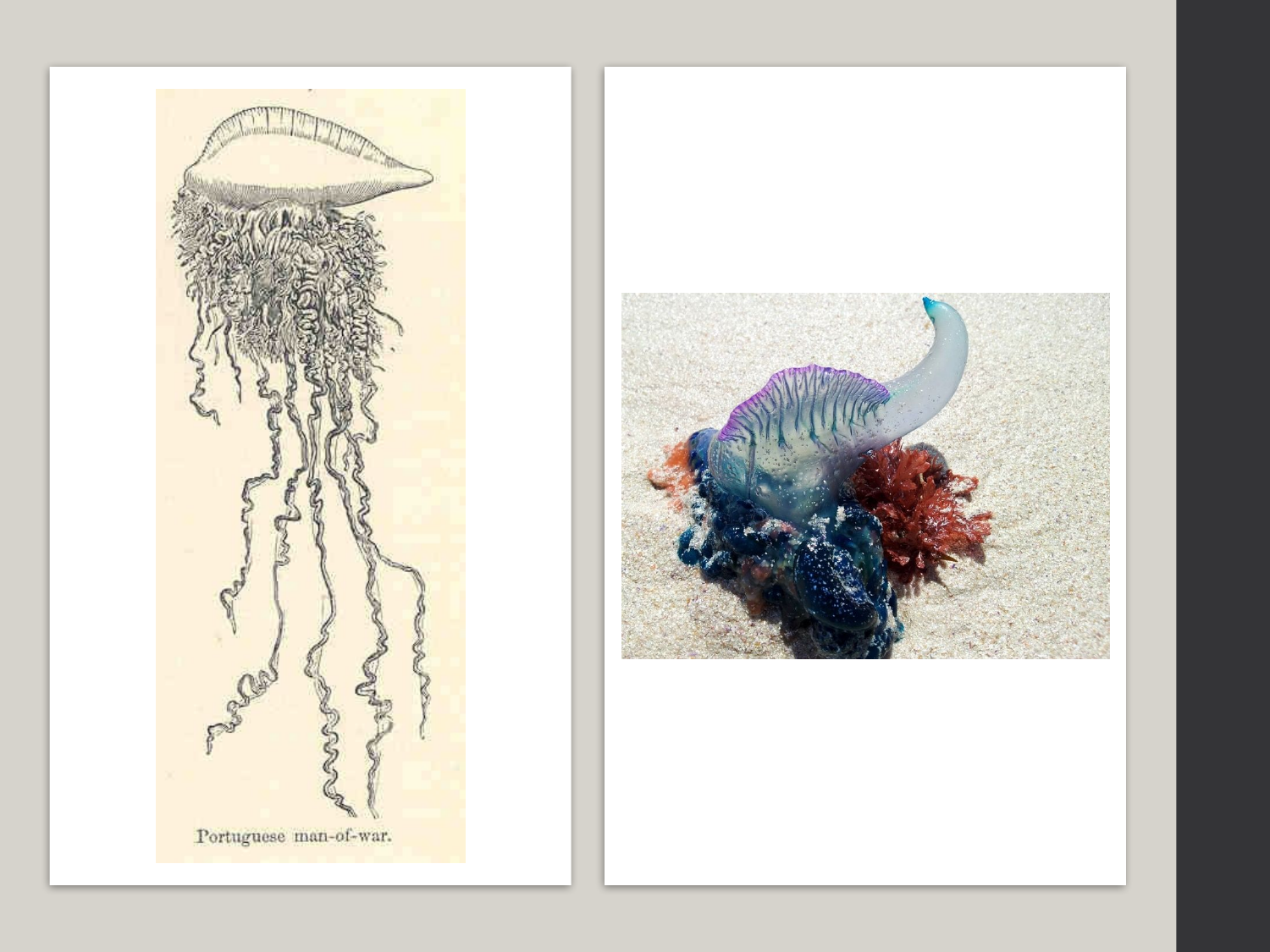

## Slide 14
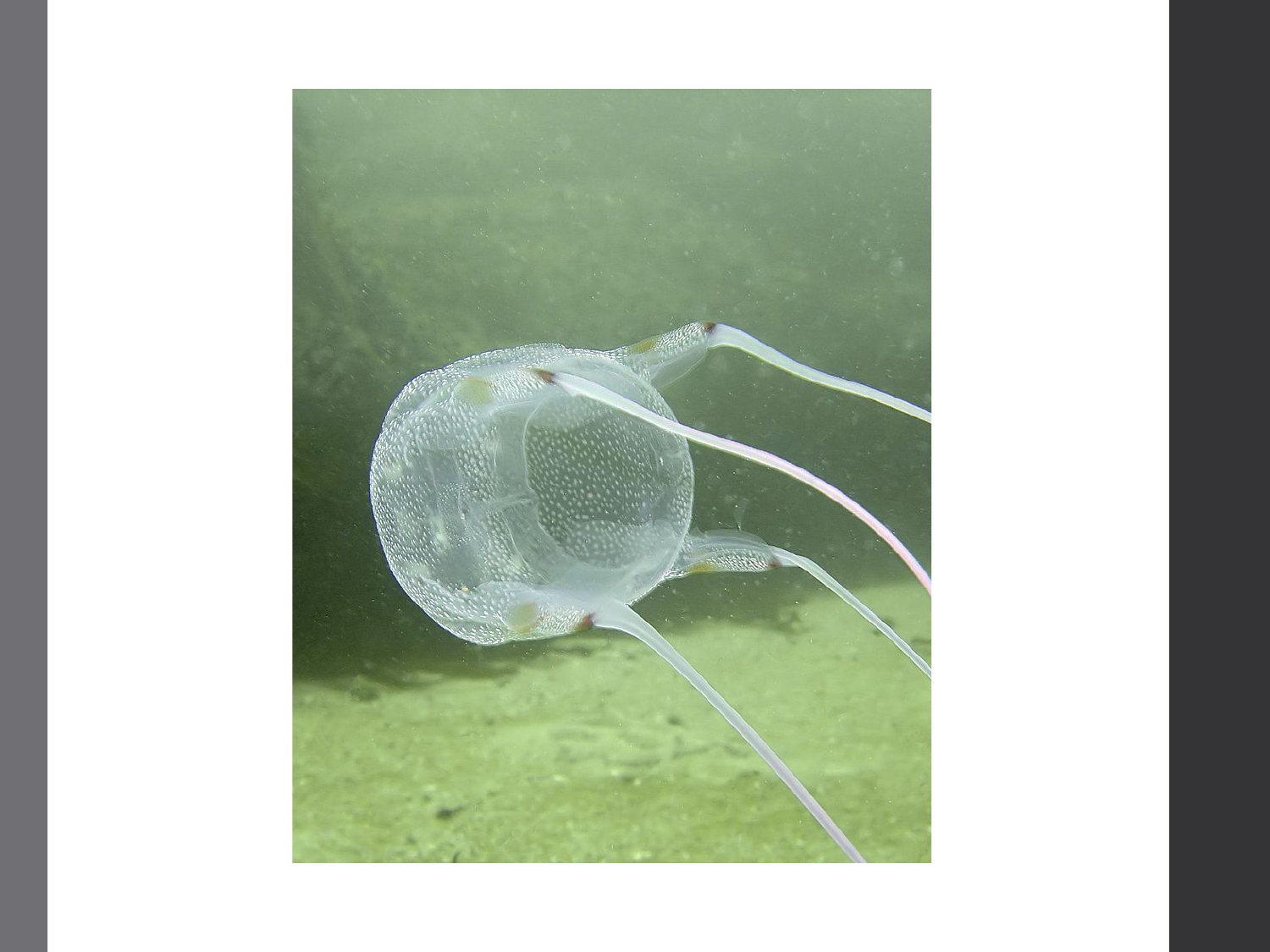

## Slide 15
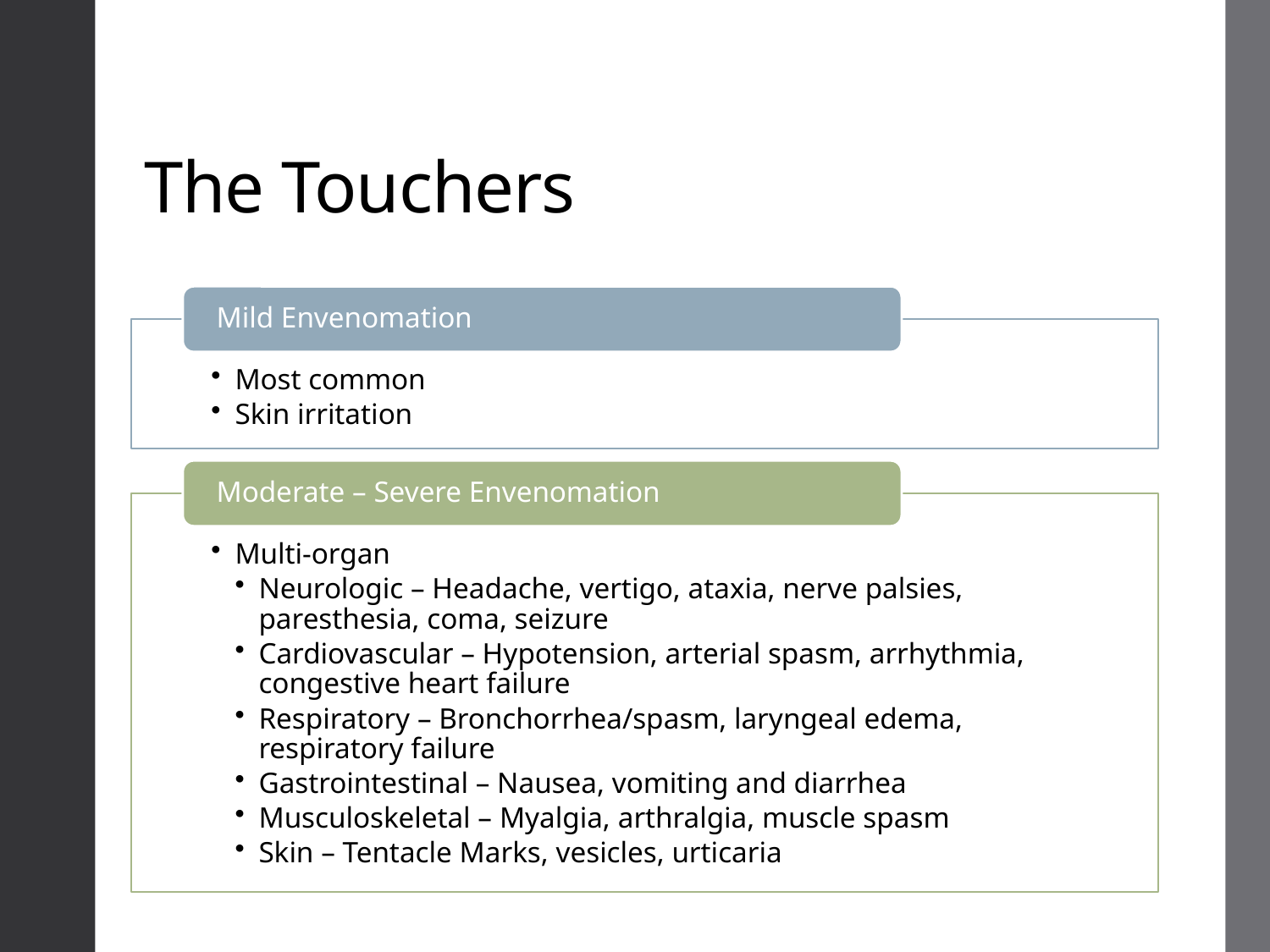

# The Touchers

## Slide 16
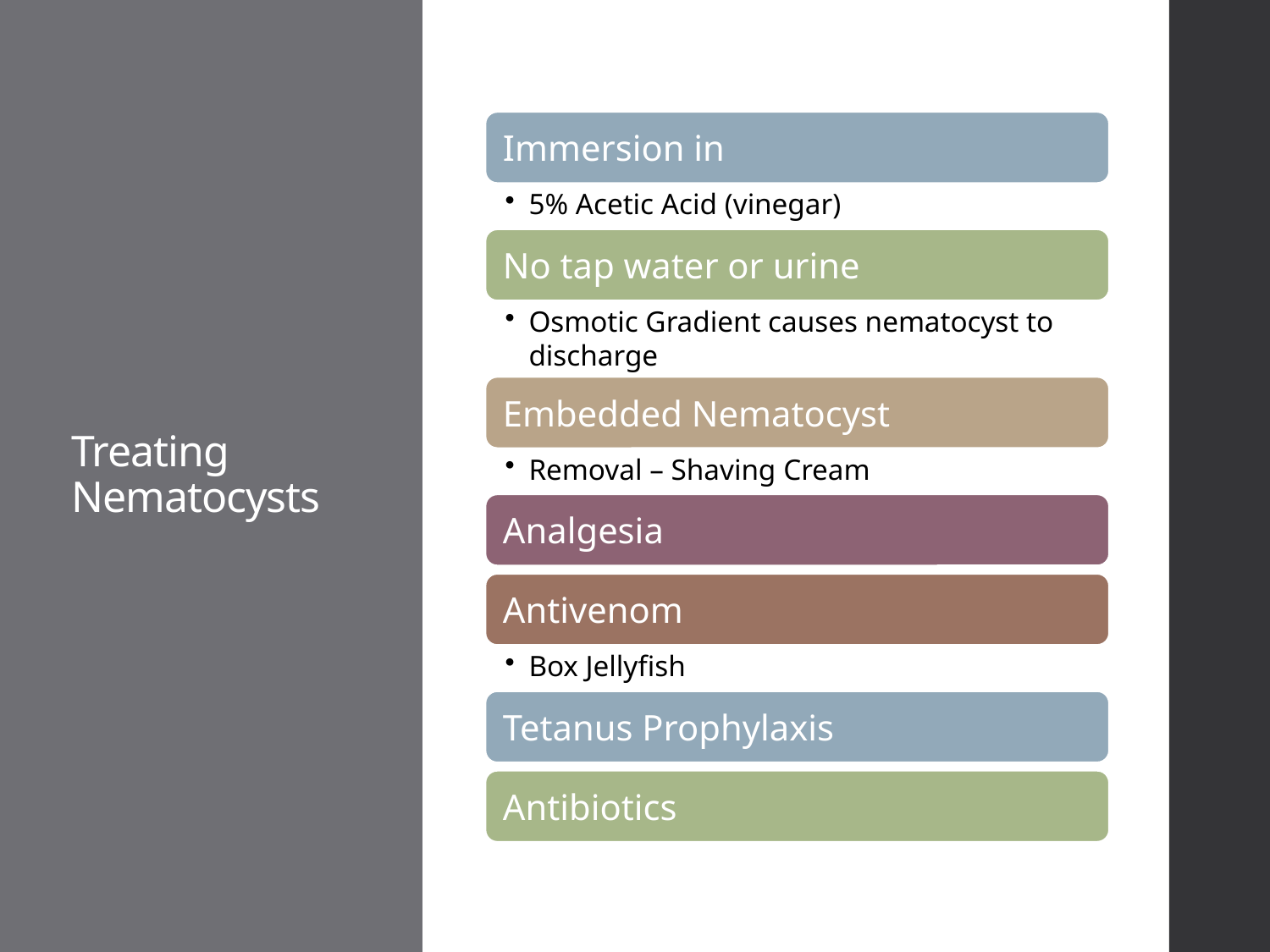

# Treating Nematocysts

## Slide 17
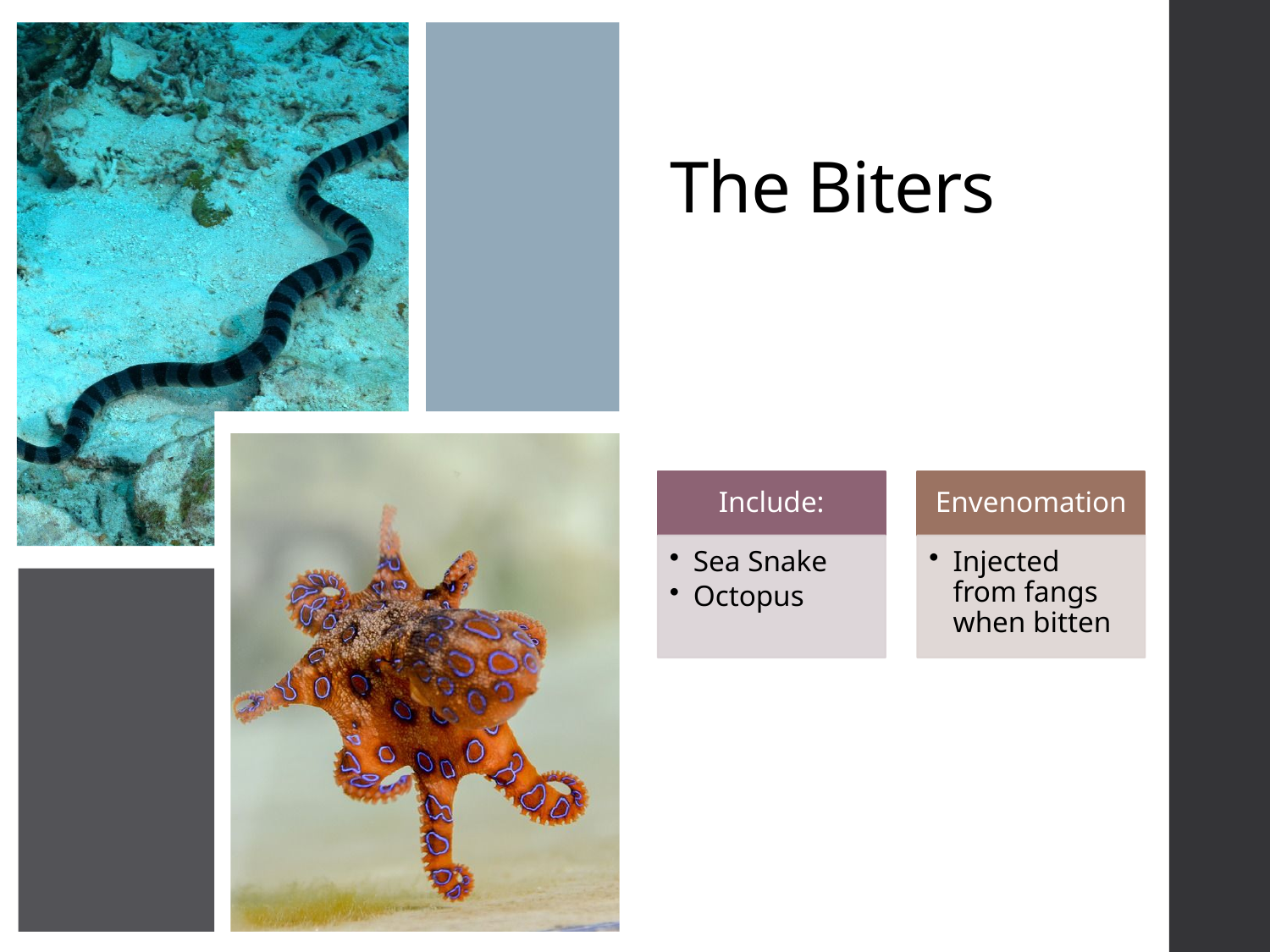

# The Biters

## Slide 18
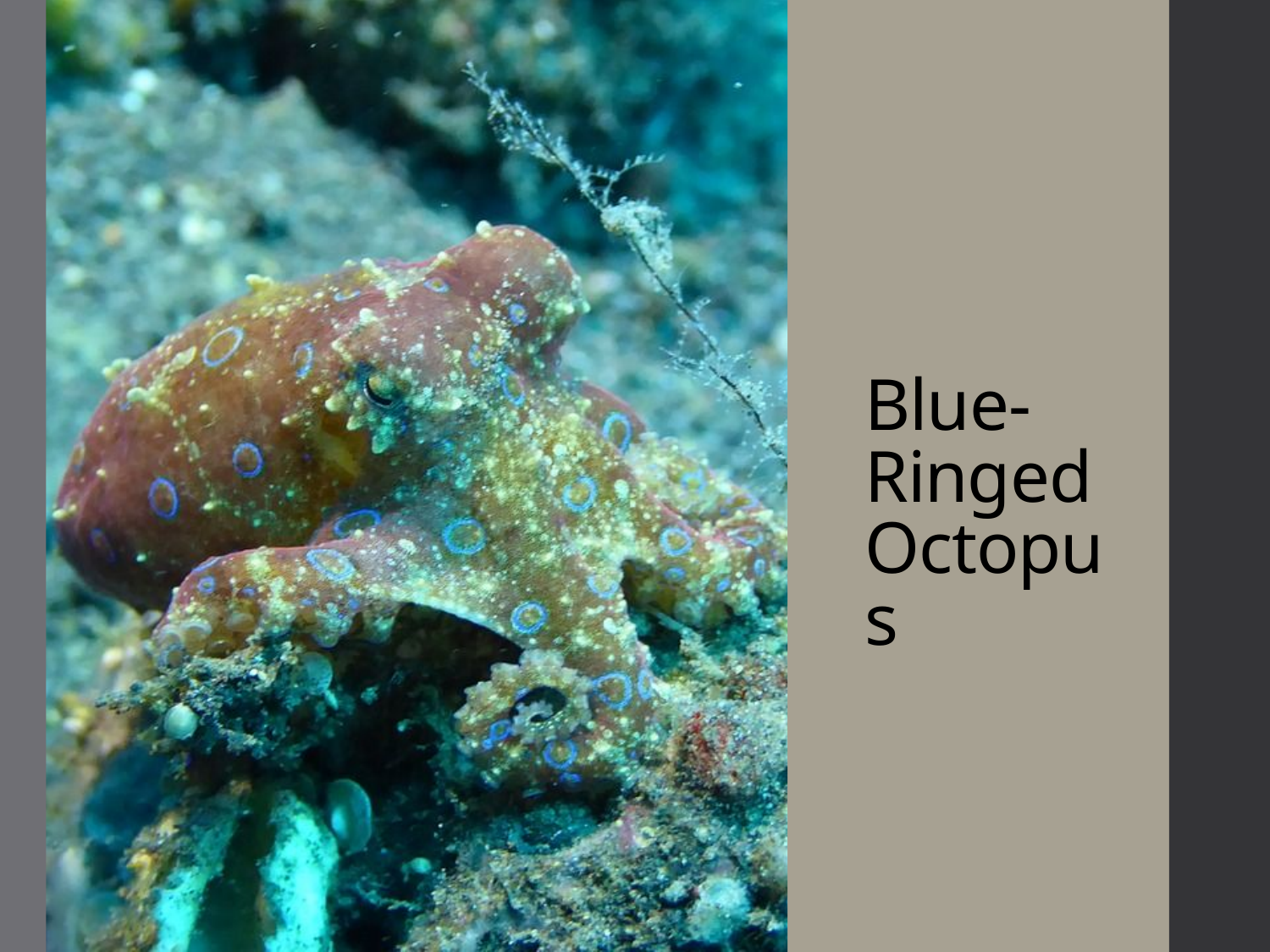

# Blue-Ringed Octopus

## Slide 19
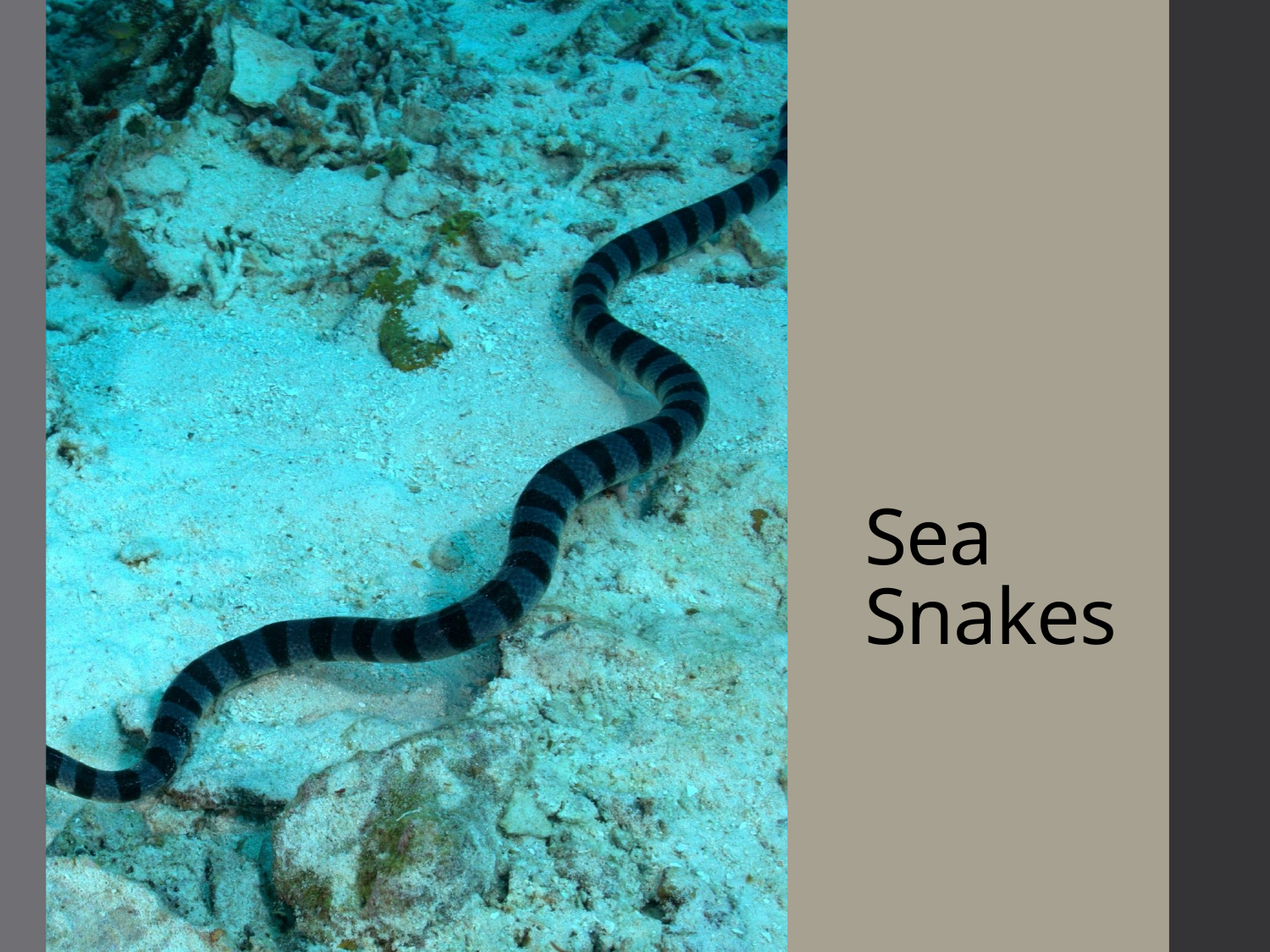

# Sea Snakes

## Slide 20
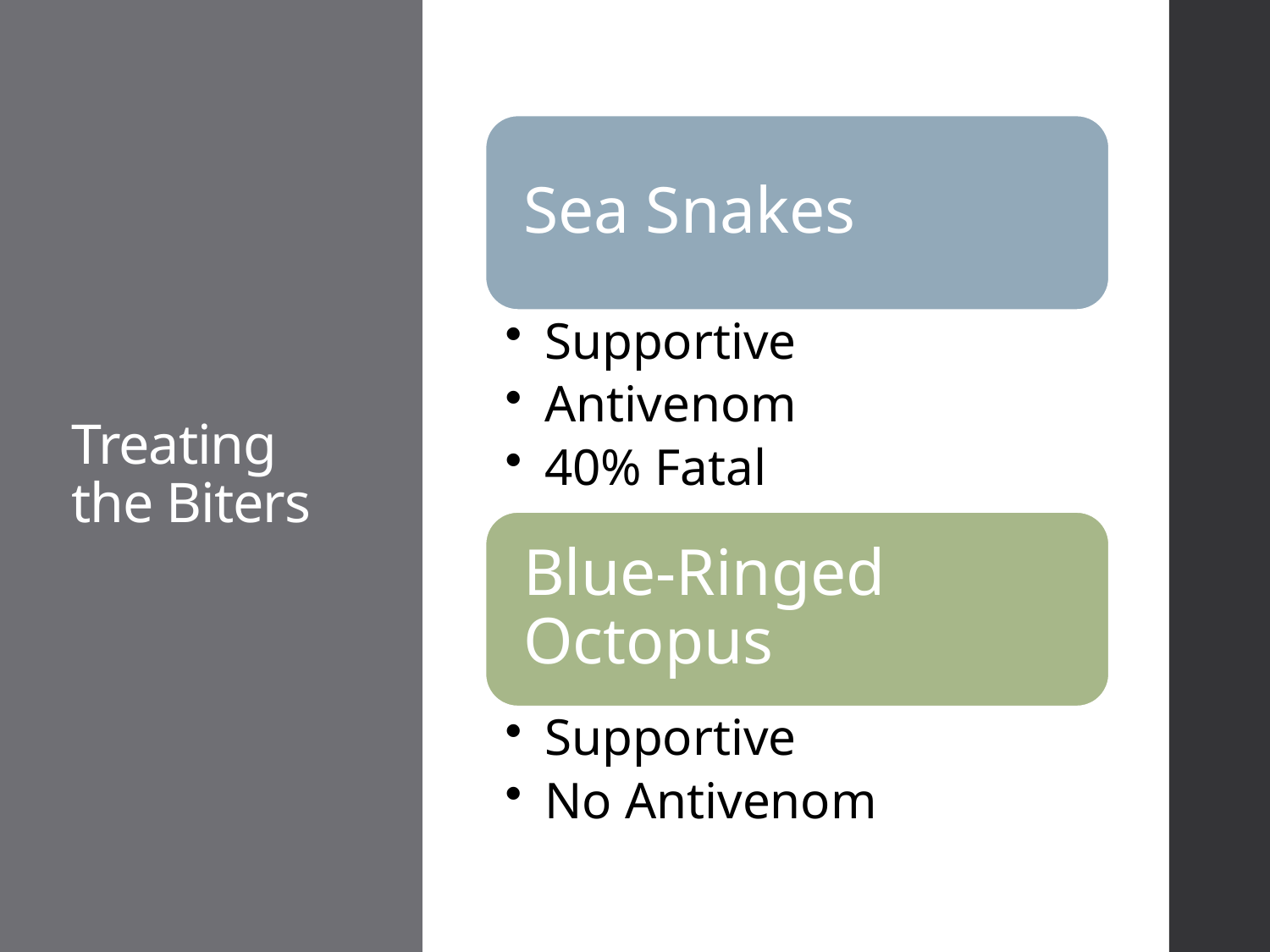

# Treating the Biters
