# Supplementary material for: Introduction to Wilderness Medicine—A Medical School Elective
Source: J Educ Teach Emerg Med. 2020 Jan 15;5(1):C1–C120. doi: 10.21980/J8B93X (PMC10332540; doi:10.21980/J8B93X)
Supplement: Supplementary file 2 — Please see associated lecture [file jetem-5-1-c1-appendixq.pptx]

## Slide 1
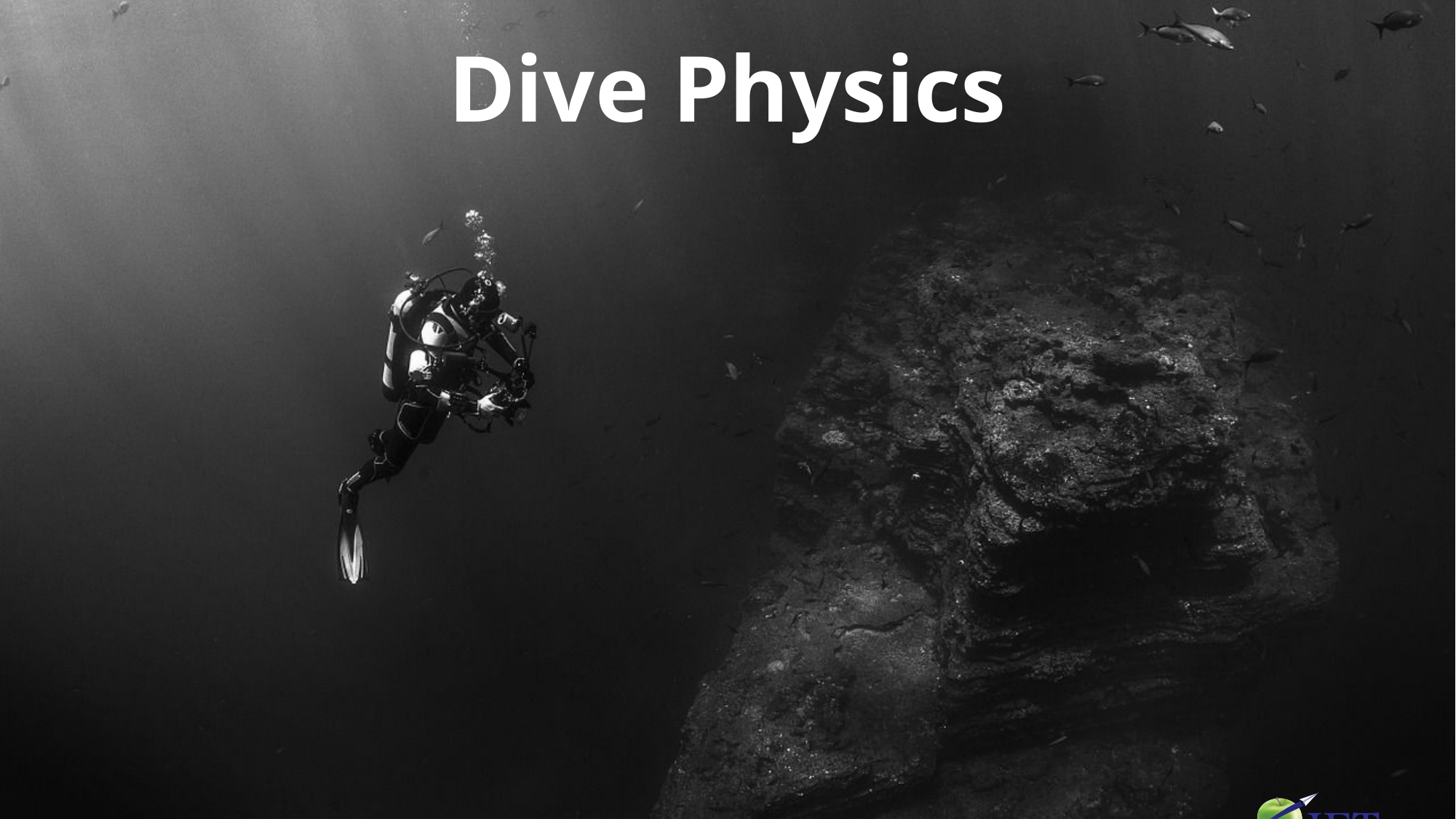

# Dive Physics

## Slide 2
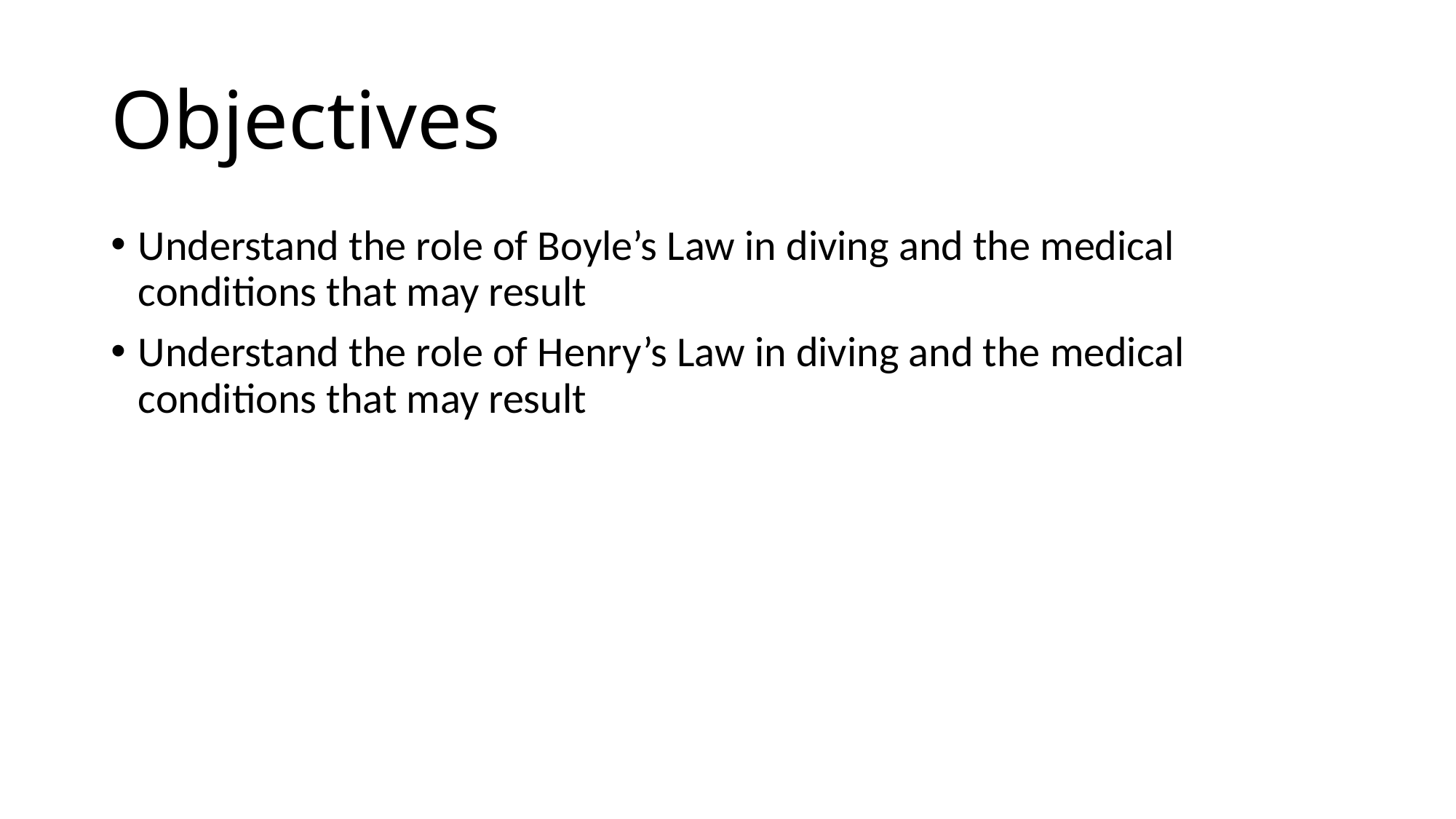

# Objectives
Understand the role of Boyle’s Law in diving and the medical conditions that may result
Understand the role of Henry’s Law in diving and the medical conditions that may result

## Slide 3
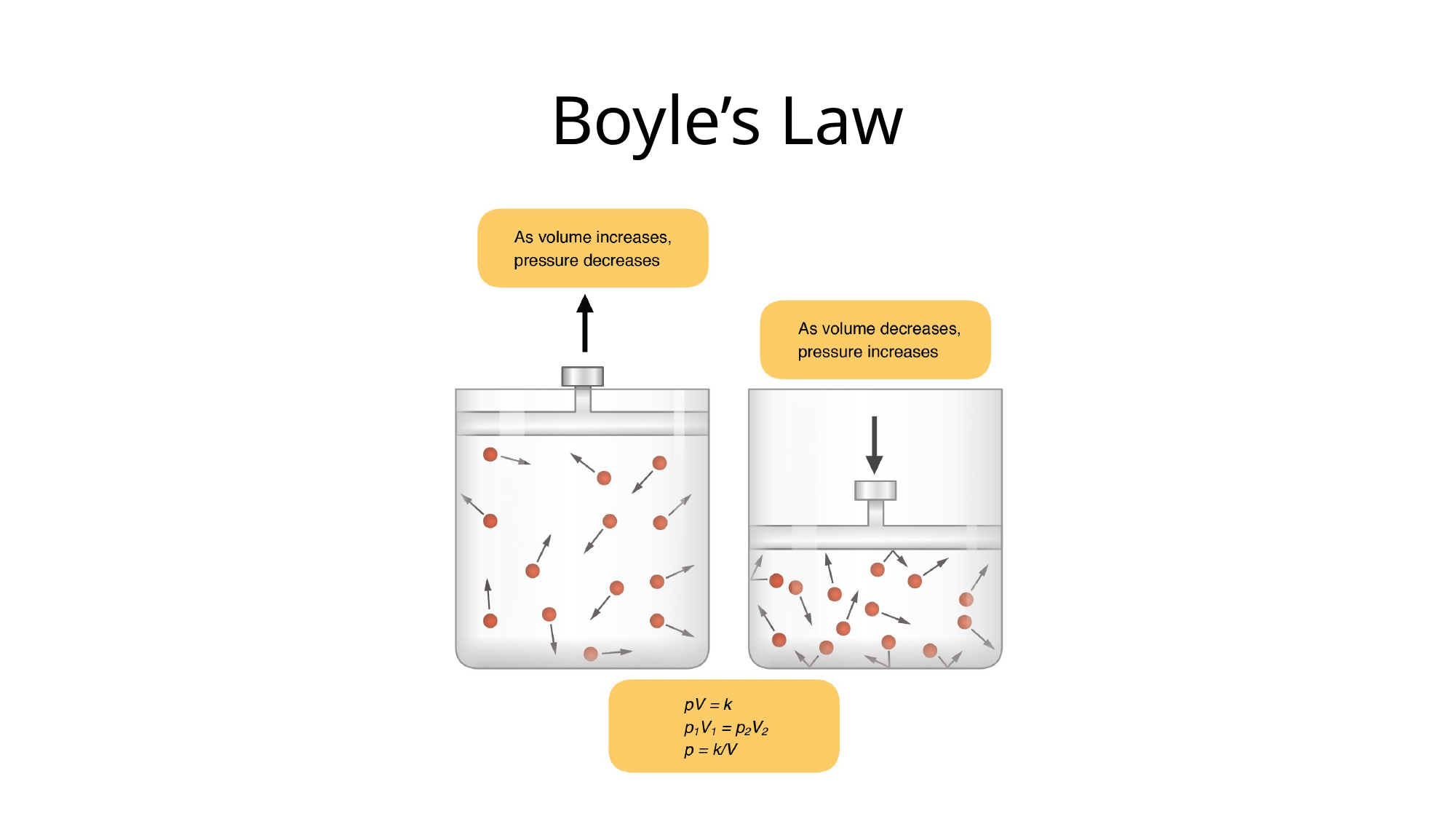

# Boyle’s Law

## Slide 4
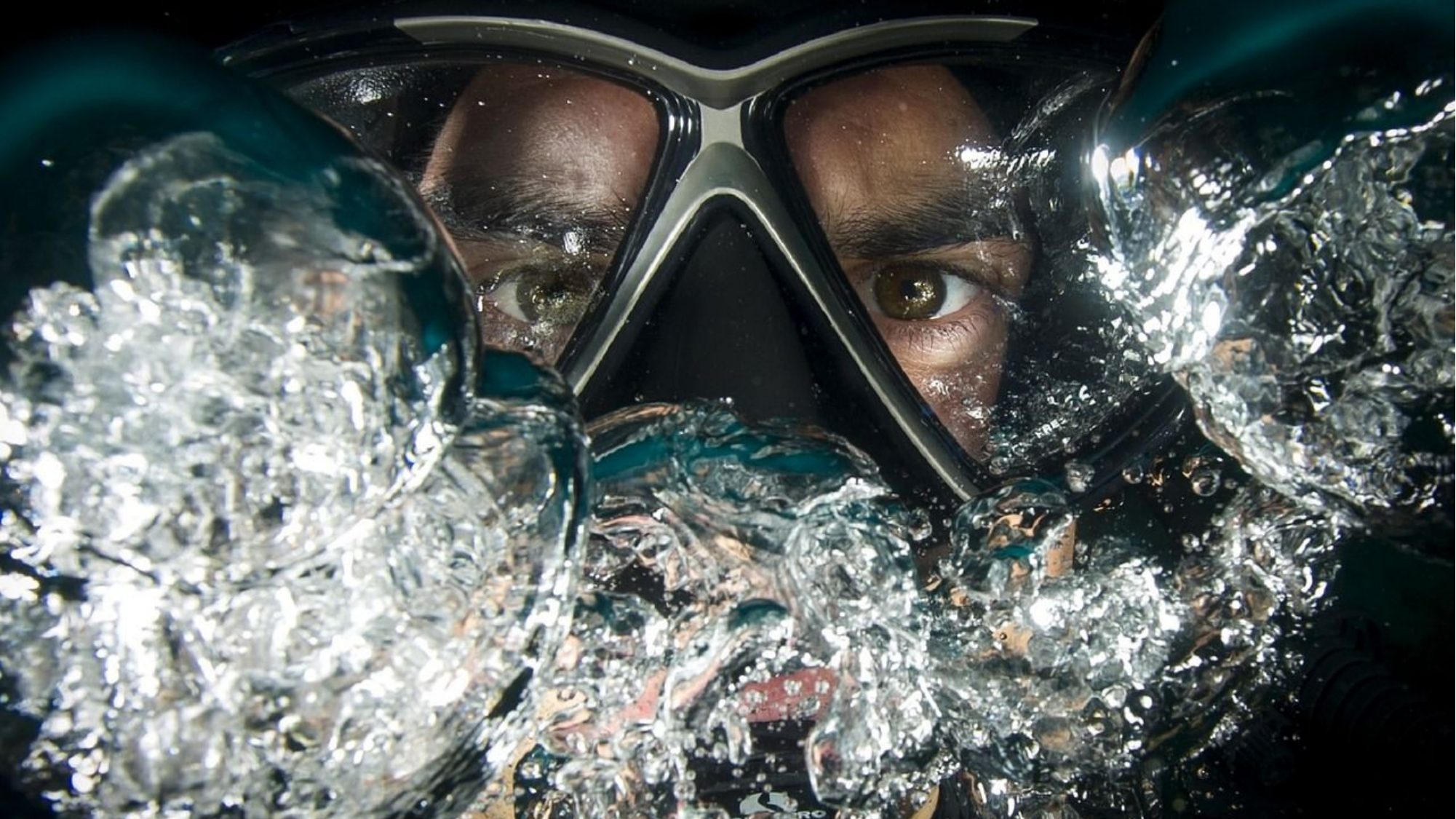

#

## Slide 5
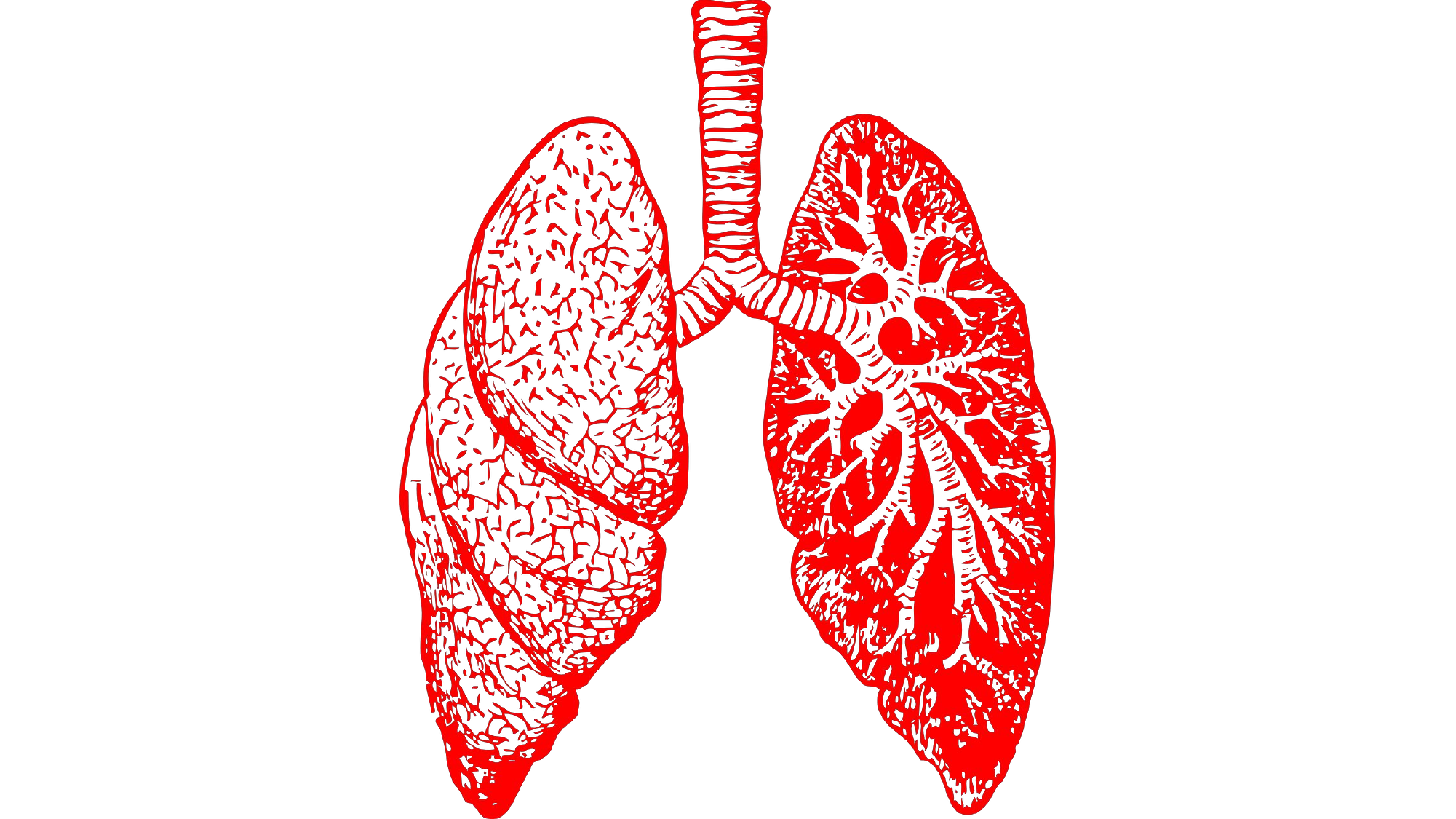

## Slide 6
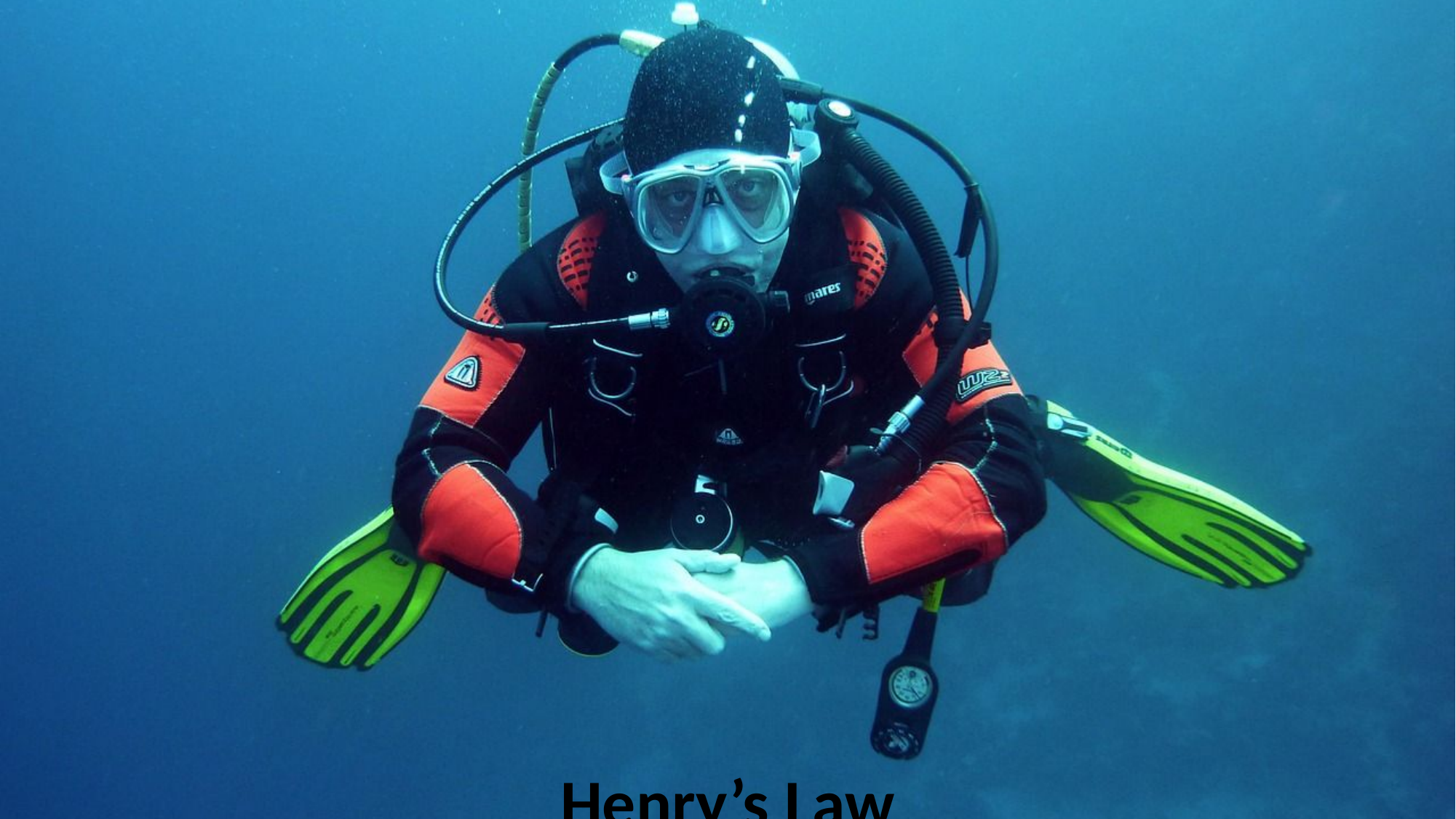

# Henry’s Law
Henry’s Law
