# Supplementary material for: Introduction to Wilderness Medicine—A Medical School Elective
Source: J Educ Teach Emerg Med. 2020 Jan 15;5(1):C1–C120. doi: 10.21980/J8B93X (PMC10332540; doi:10.21980/J8B93X)
Supplement: Supplementary file 3 — Please see associated lecture [file jetem-5-1-c1-appendixr.pptx]

## Slide 1
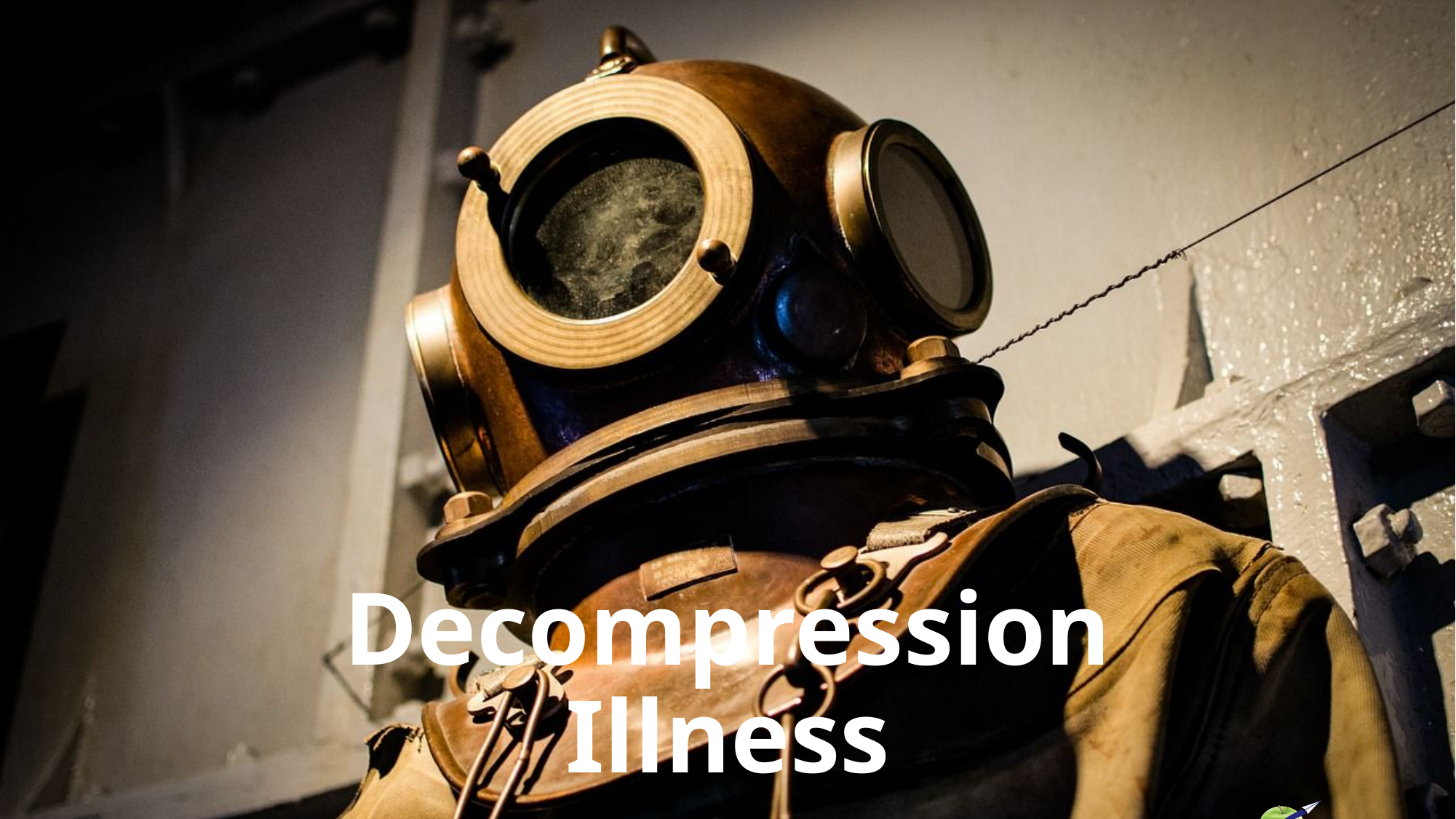

# Decompression Illness

## Slide 2
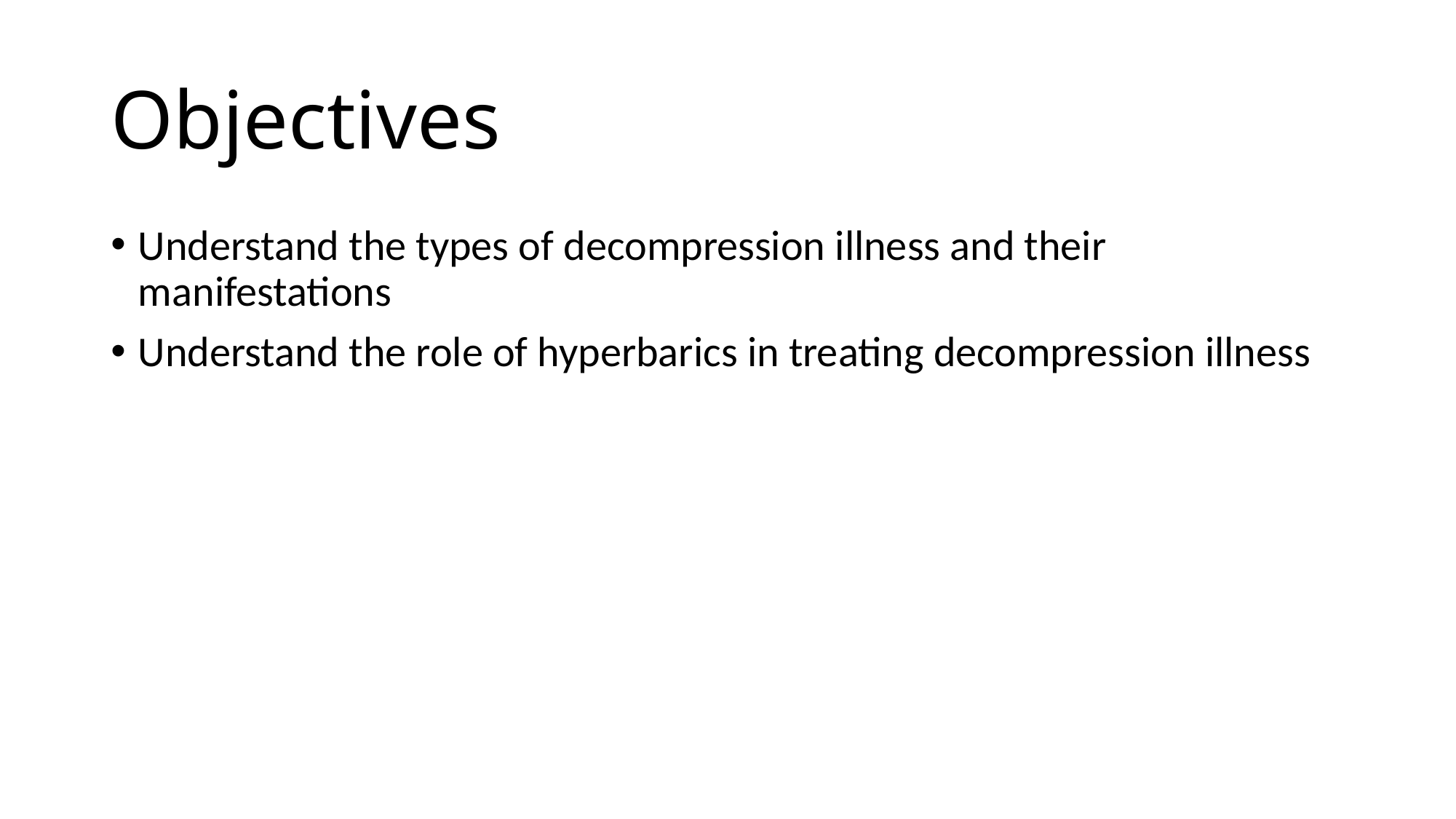

# Objectives
Understand the types of decompression illness and their manifestations
Understand the role of hyperbarics in treating decompression illness

## Slide 3
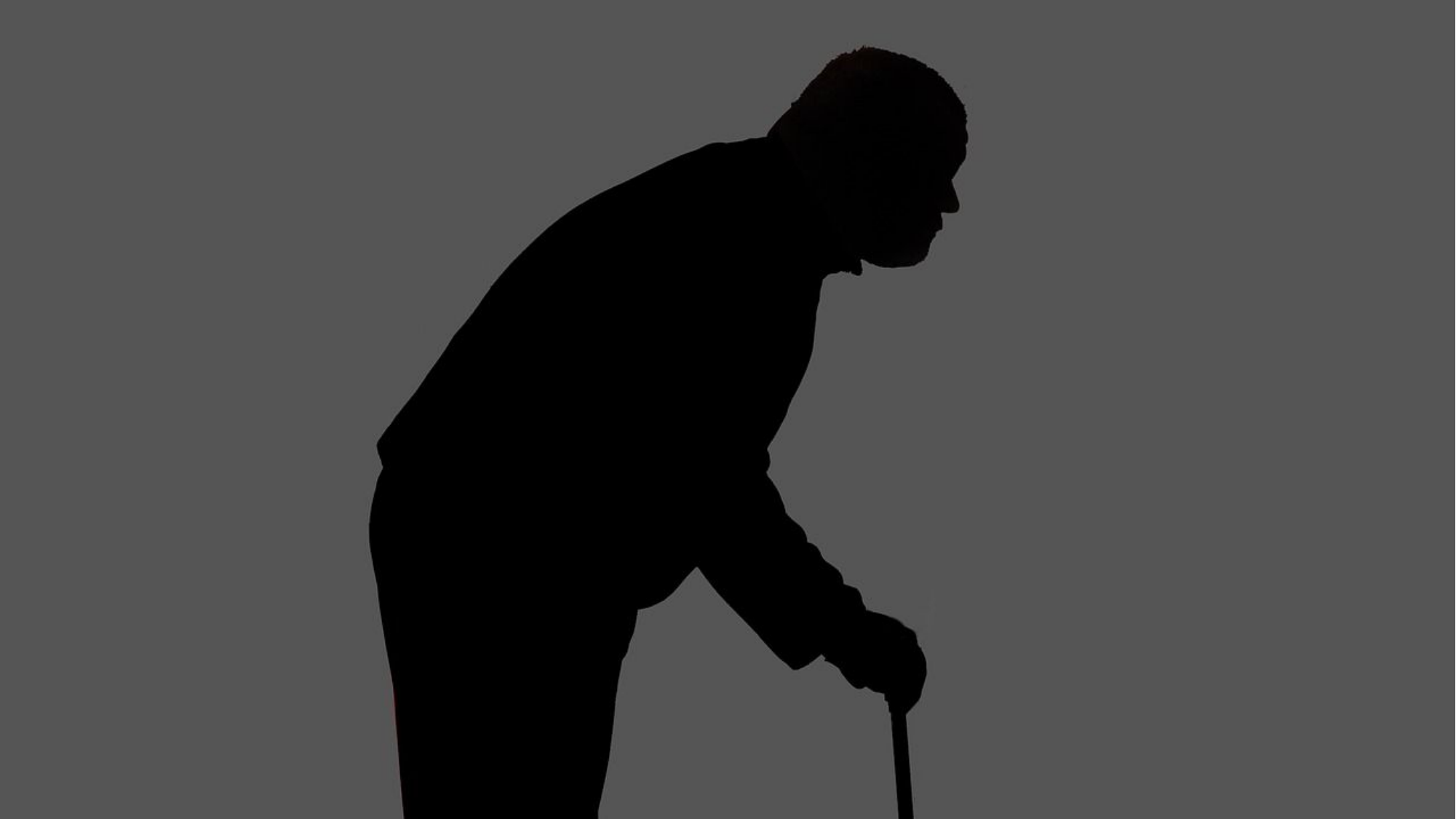

#

## Slide 4
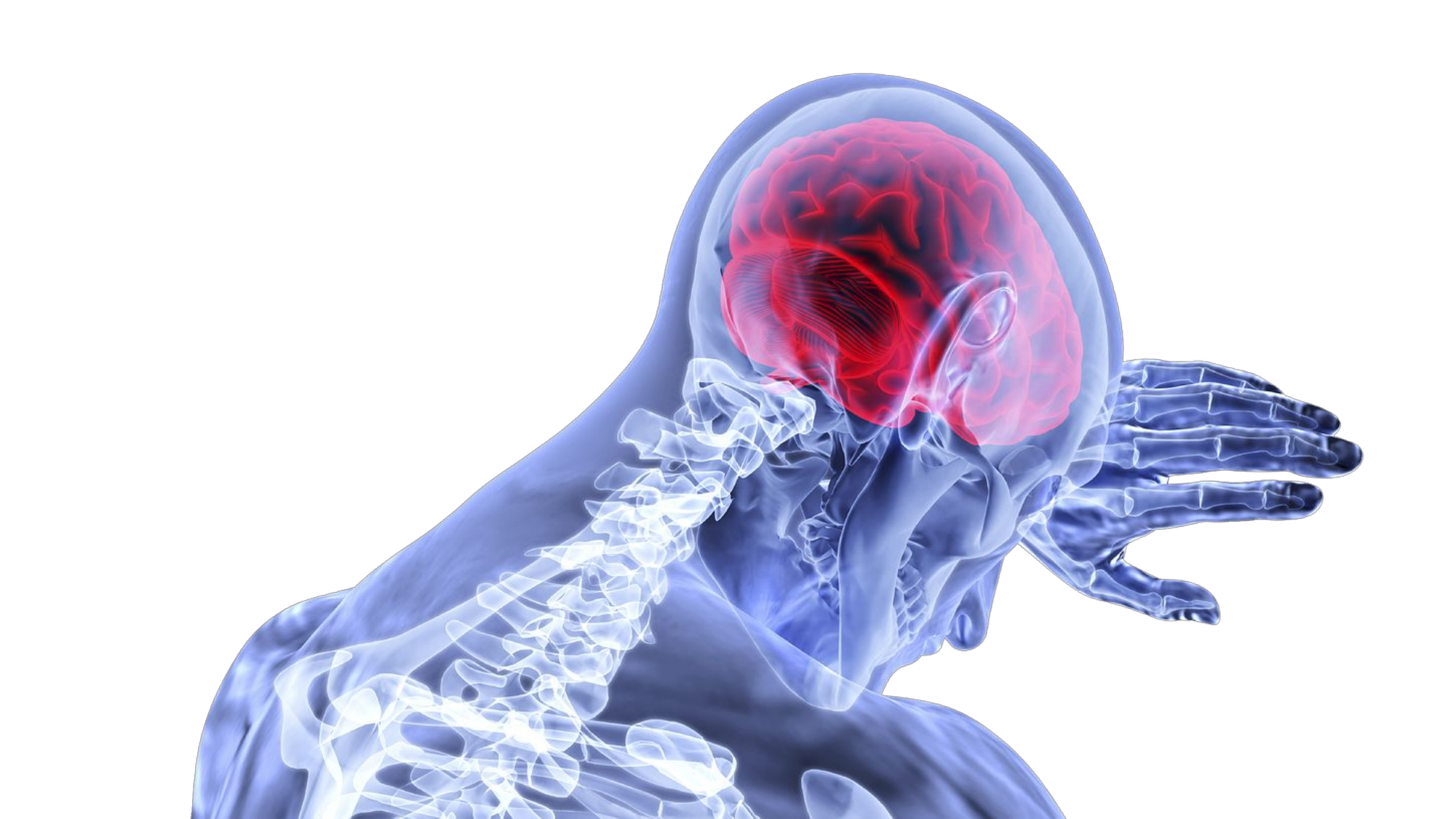

## Slide 5
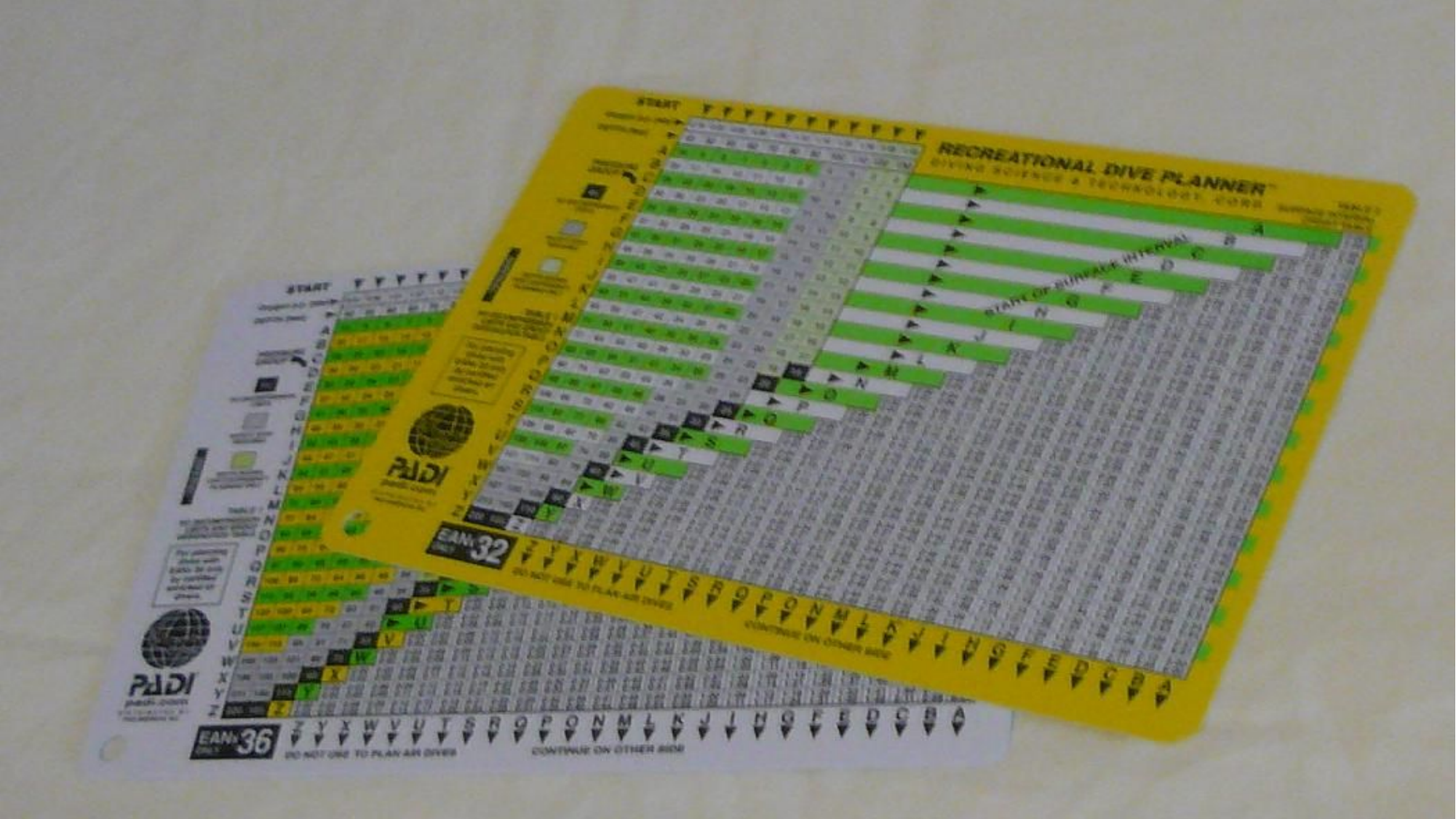

#

## Slide 6
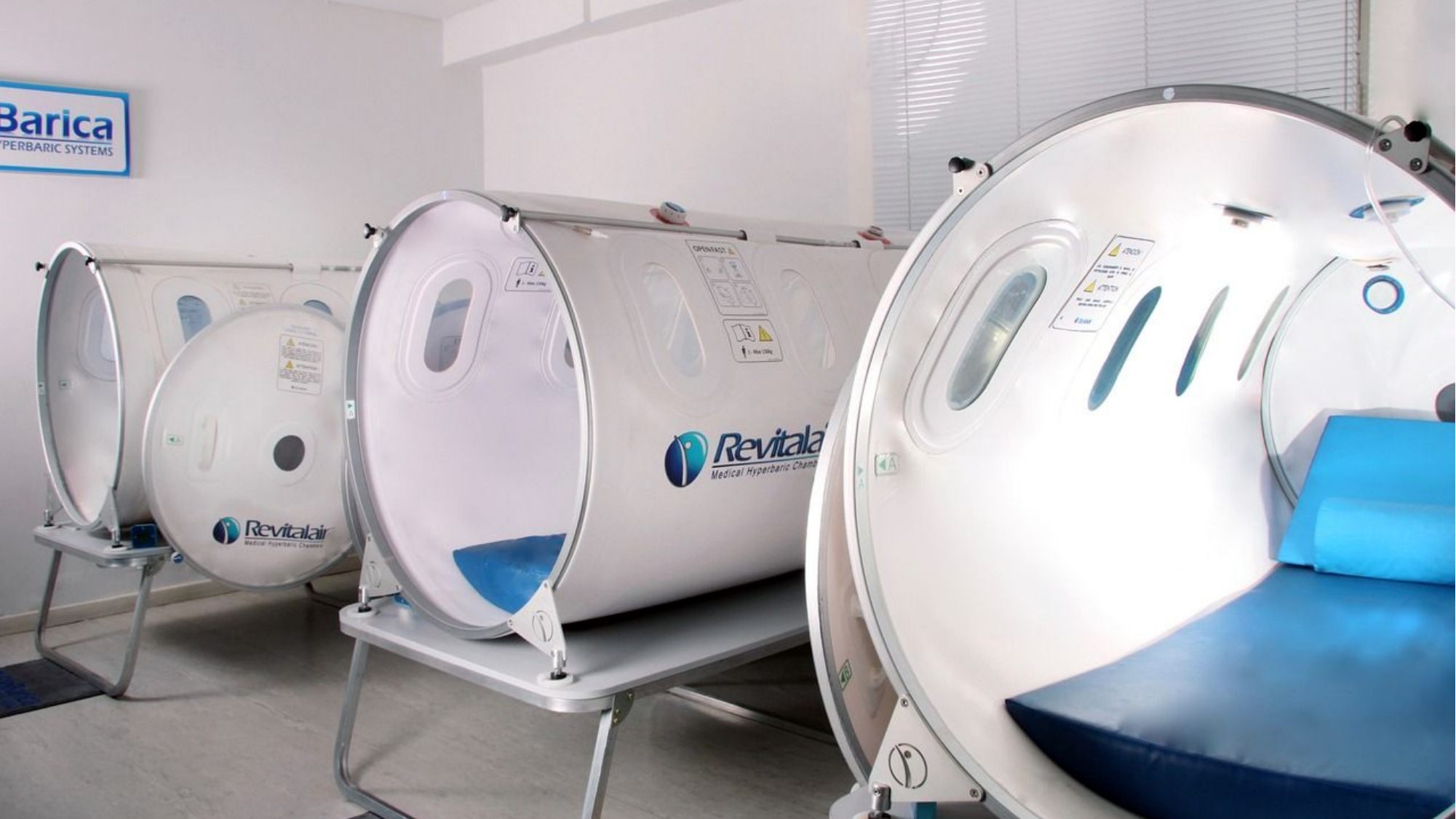

#
