# Supplementary material for: Introduction to Wilderness Medicine—A Medical School Elective
Source: J Educ Teach Emerg Med. 2020 Jan 15;5(1):C1–C120. doi: 10.21980/J8B93X (PMC10332540; doi:10.21980/J8B93X)
Supplement: Supplementary file 4 — Please see associated lecture [file jetem-5-1-c1-appendixs.pptx]

## Slide 1
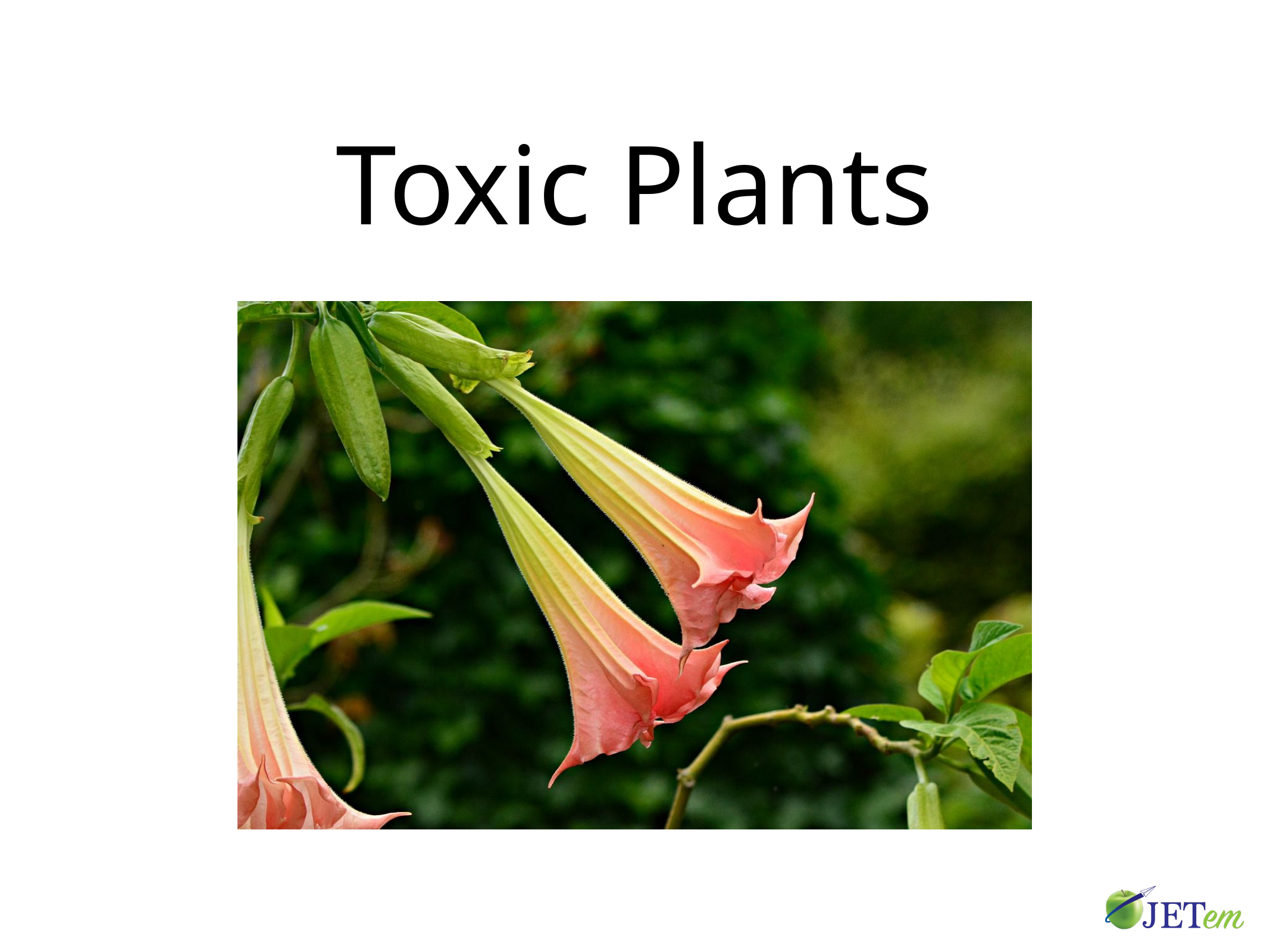

# Toxic Plants

## Slide 2
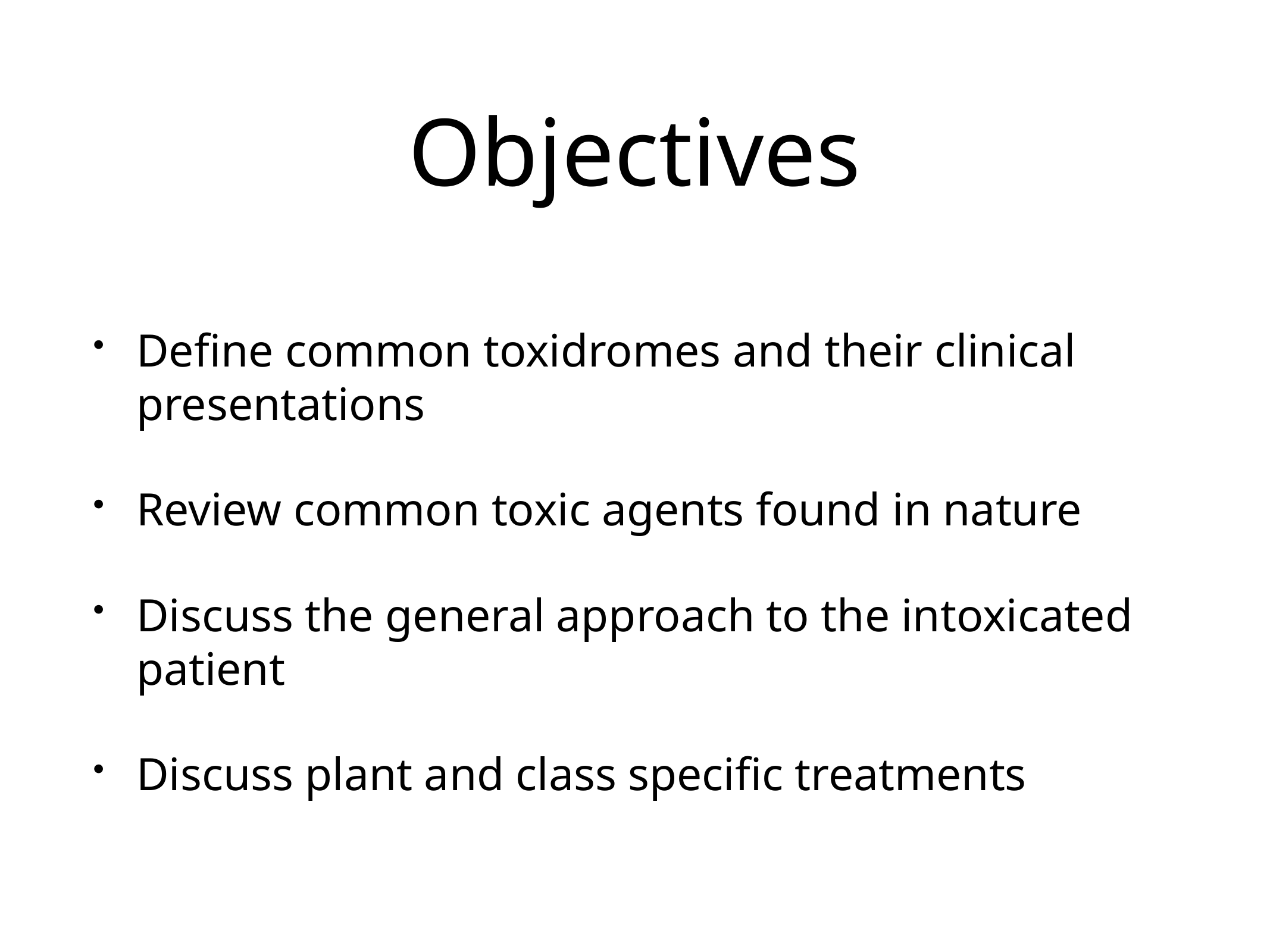

# Objectives
Define common toxidromes and their clinical presentations
Review common toxic agents found in nature
Discuss the general approach to the intoxicated patient
Discuss plant and class specific treatments

## Slide 3
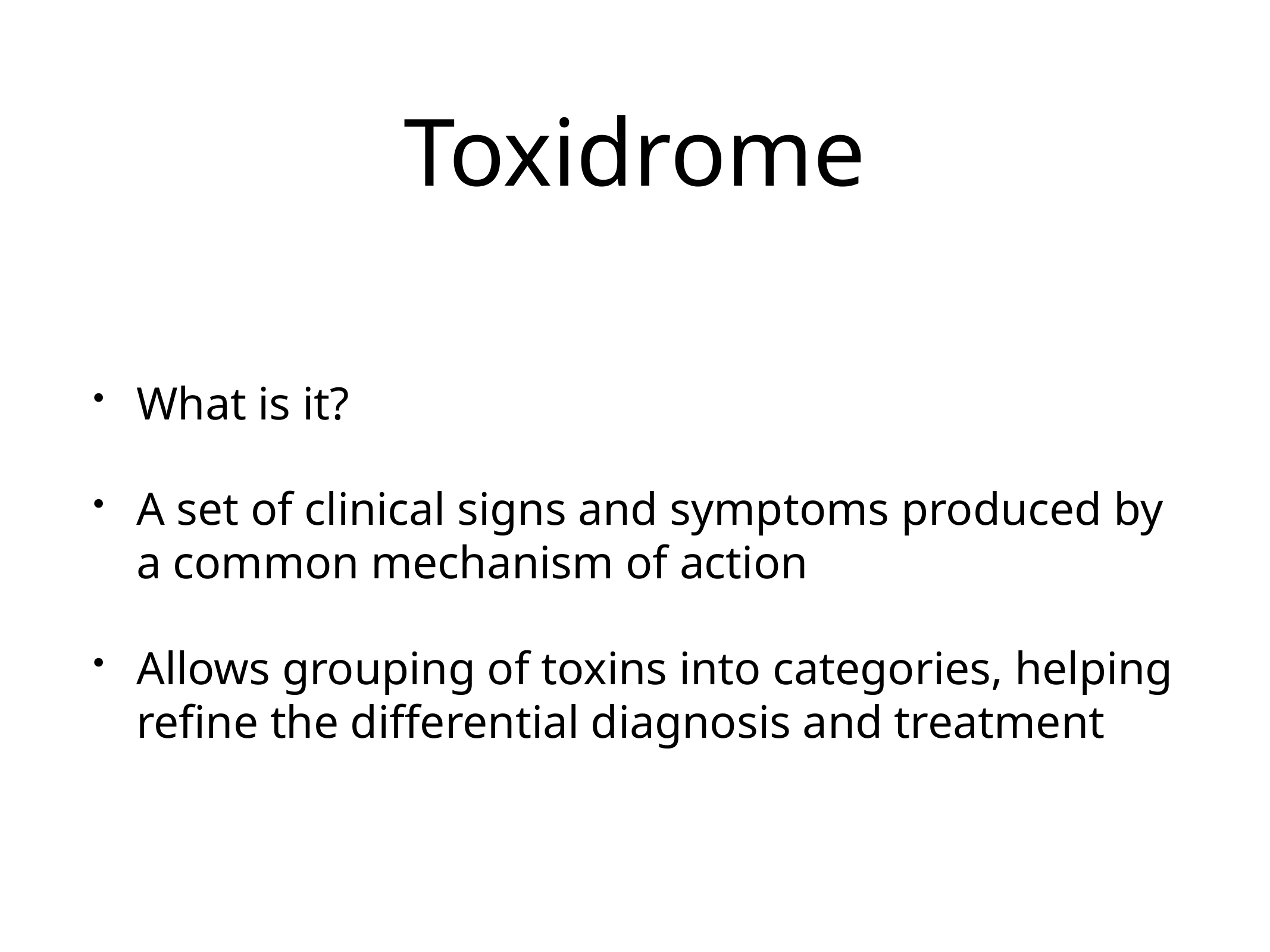

# Toxidrome
What is it?
A set of clinical signs and symptoms produced by a common mechanism of action
Allows grouping of toxins into categories, helping refine the differential diagnosis and treatment

## Slide 4
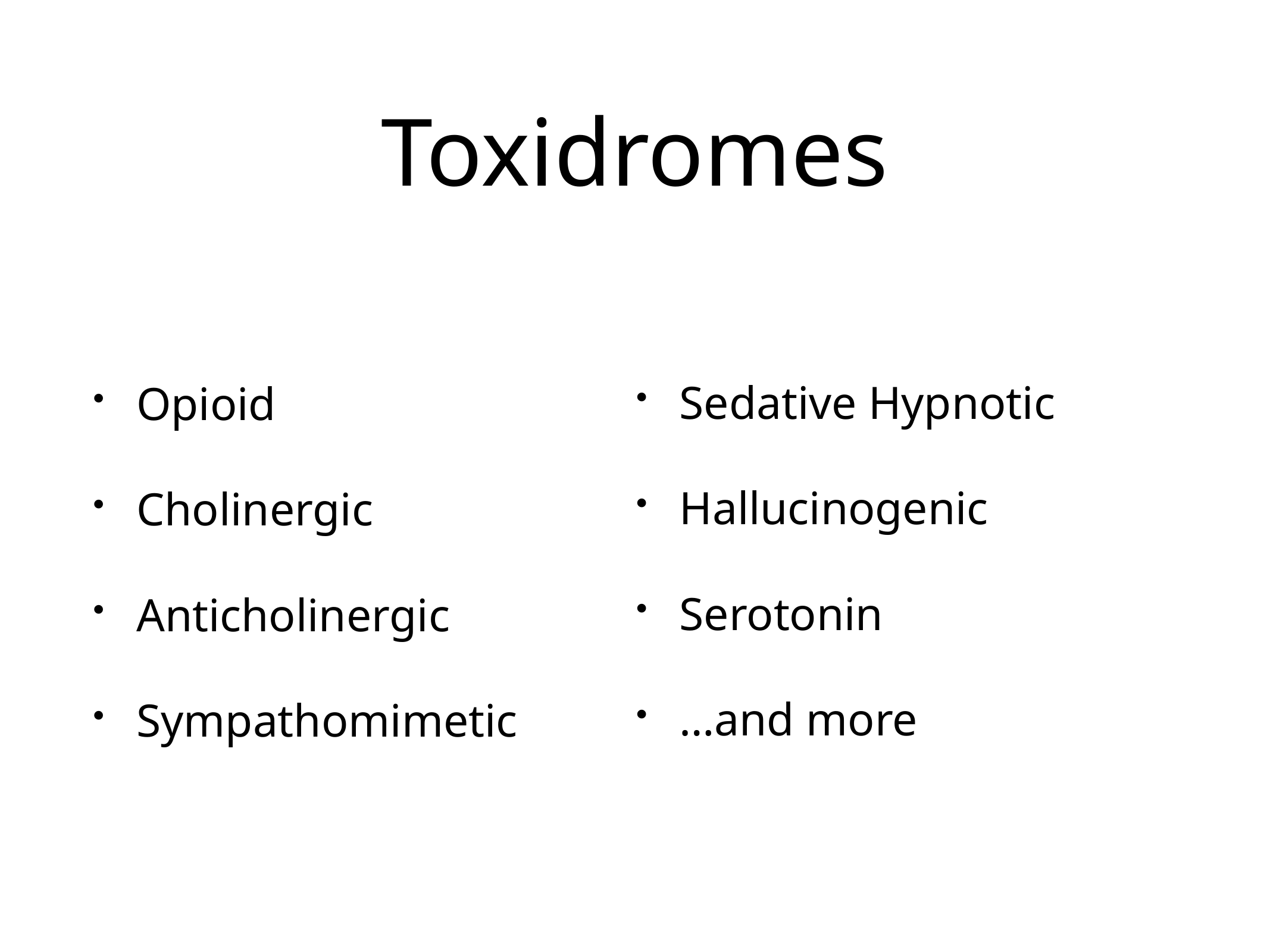

# Toxidromes
Sedative Hypnotic
Hallucinogenic
Serotonin
…and more
Opioid
Cholinergic
Anticholinergic
Sympathomimetic

## Slide 5
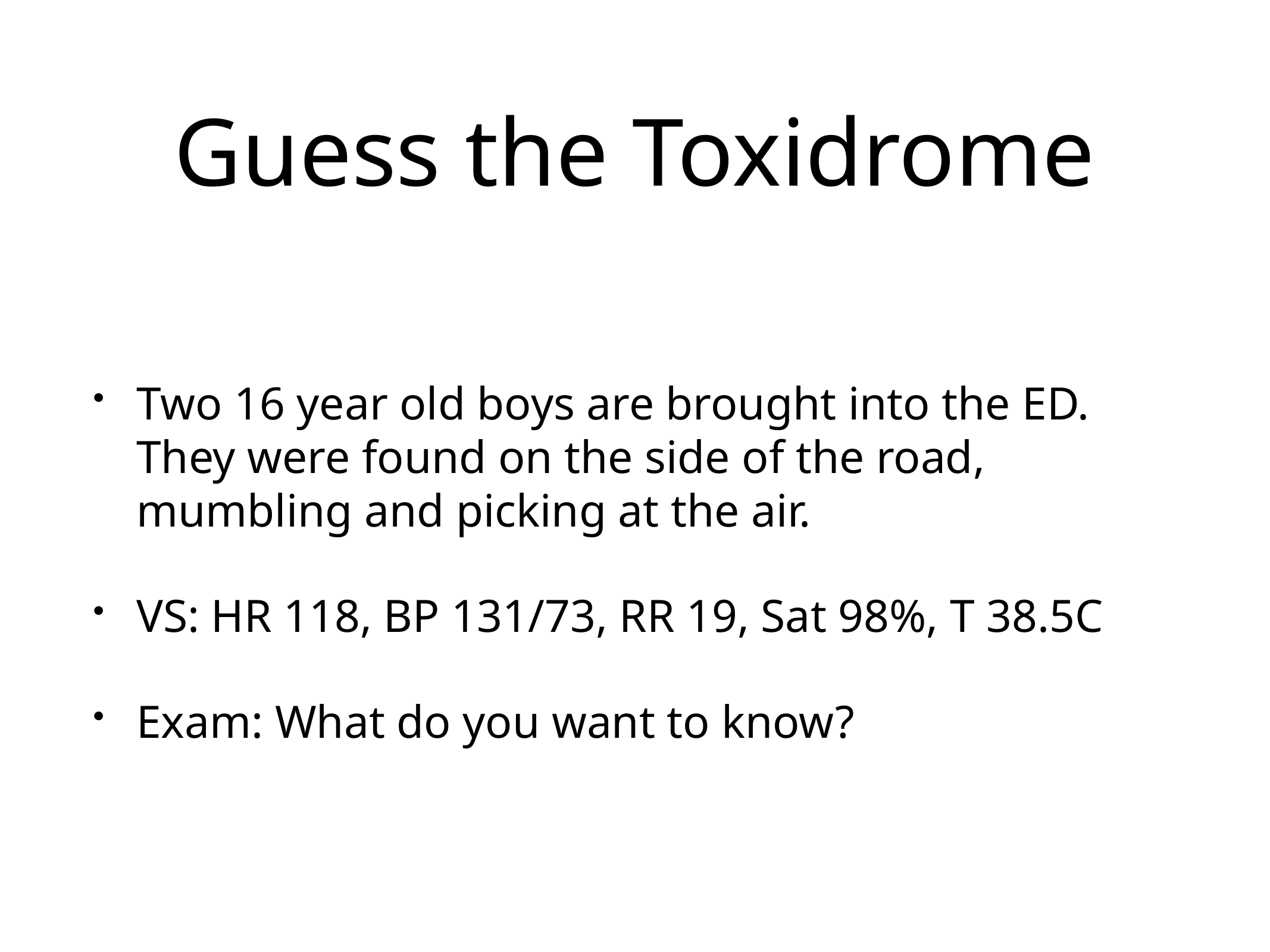

# Guess the Toxidrome
Two 16 year old boys are brought into the ED. They were found on the side of the road, mumbling and picking at the air.
VS: HR 118, BP 131/73, RR 19, Sat 98%, T 38.5C
Exam: What do you want to know?

## Slide 6
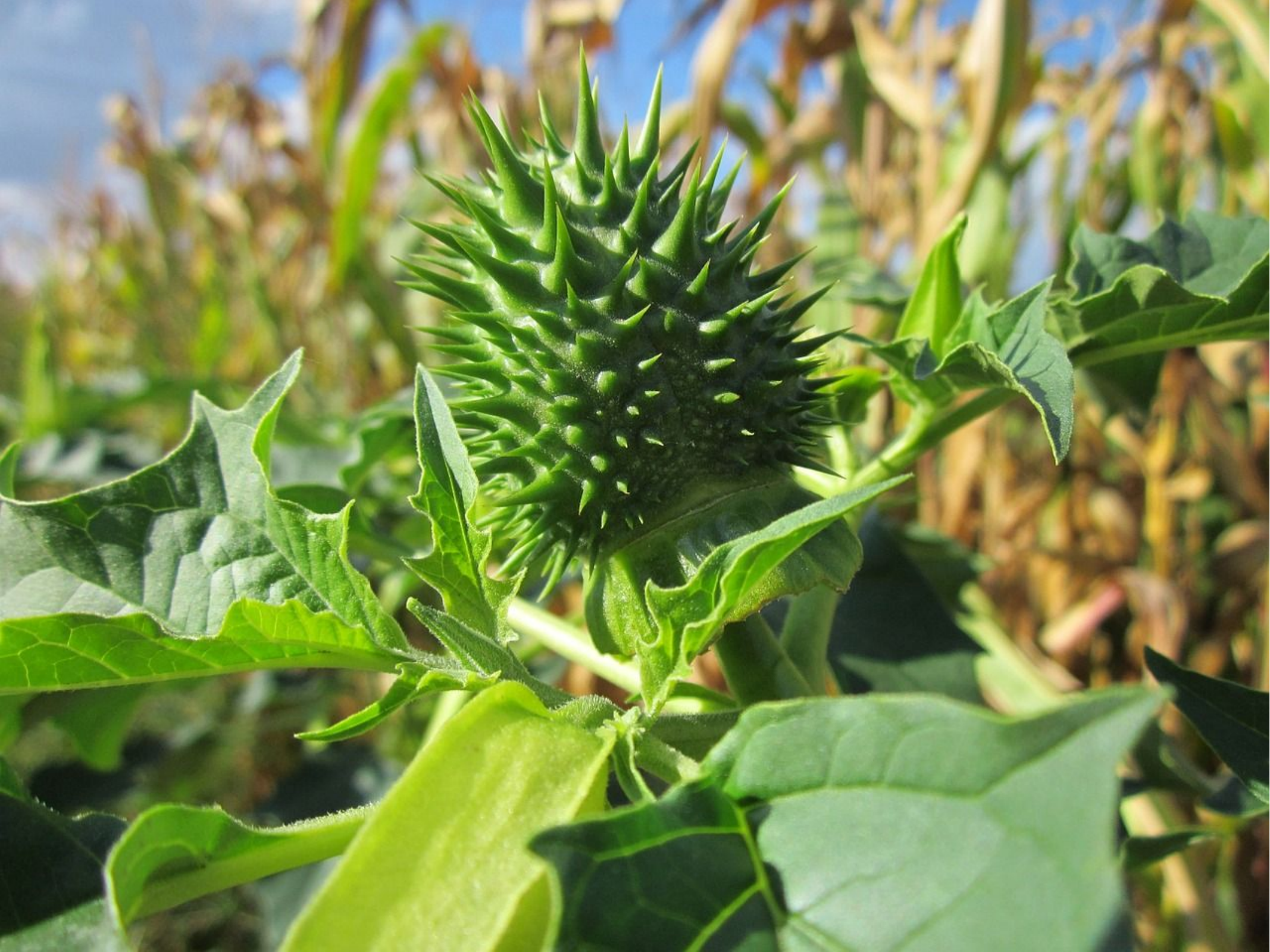

#

## Slide 7
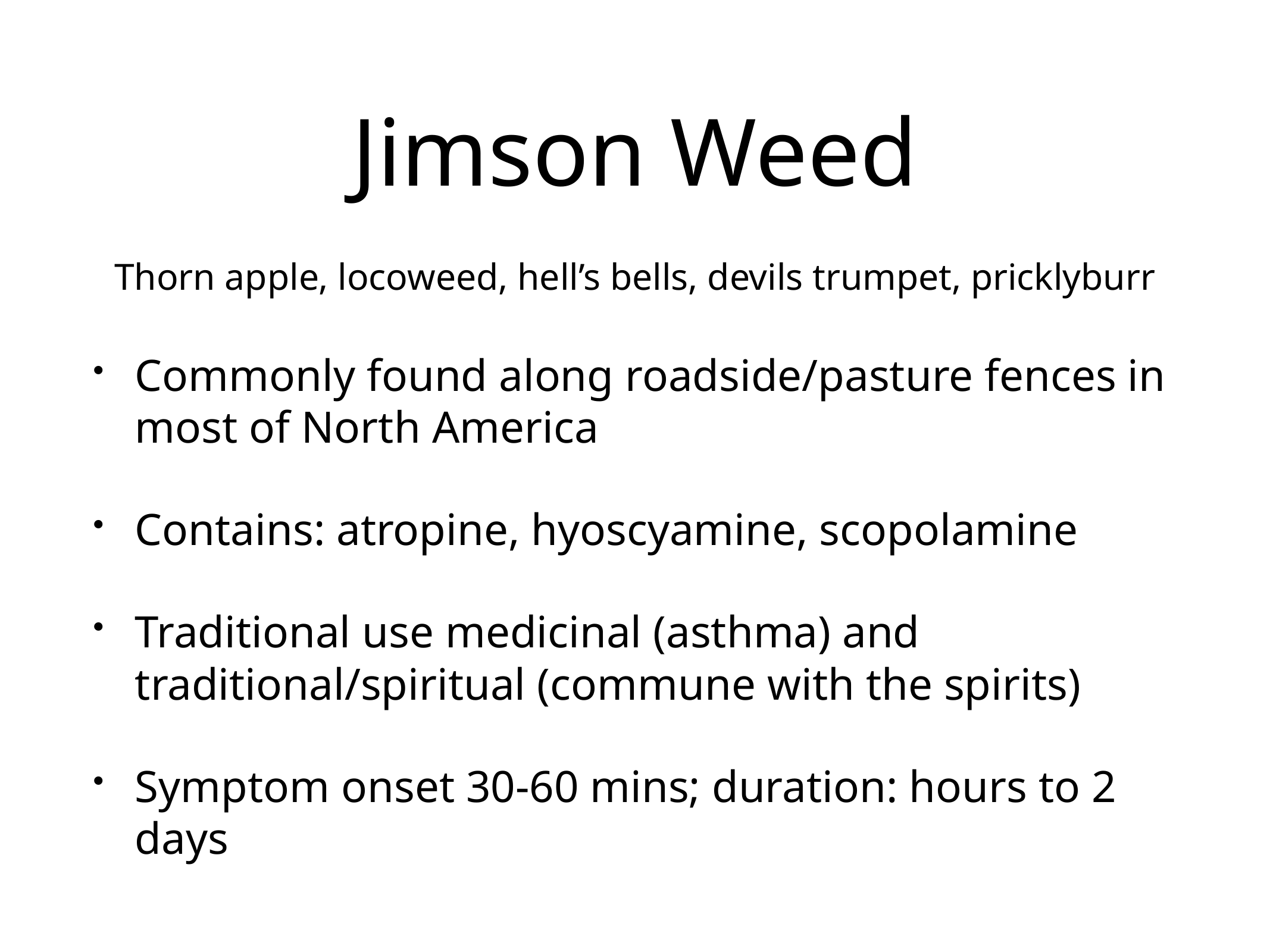

# Jimson Weed
Thorn apple, locoweed, hell’s bells, devils trumpet, pricklyburr
Commonly found along roadside/pasture fences in most of North America
Contains: atropine, hyoscyamine, scopolamine
Traditional use medicinal (asthma) and traditional/spiritual (commune with the spirits)
Symptom onset 30-60 mins; duration: hours to 2 days

## Slide 8
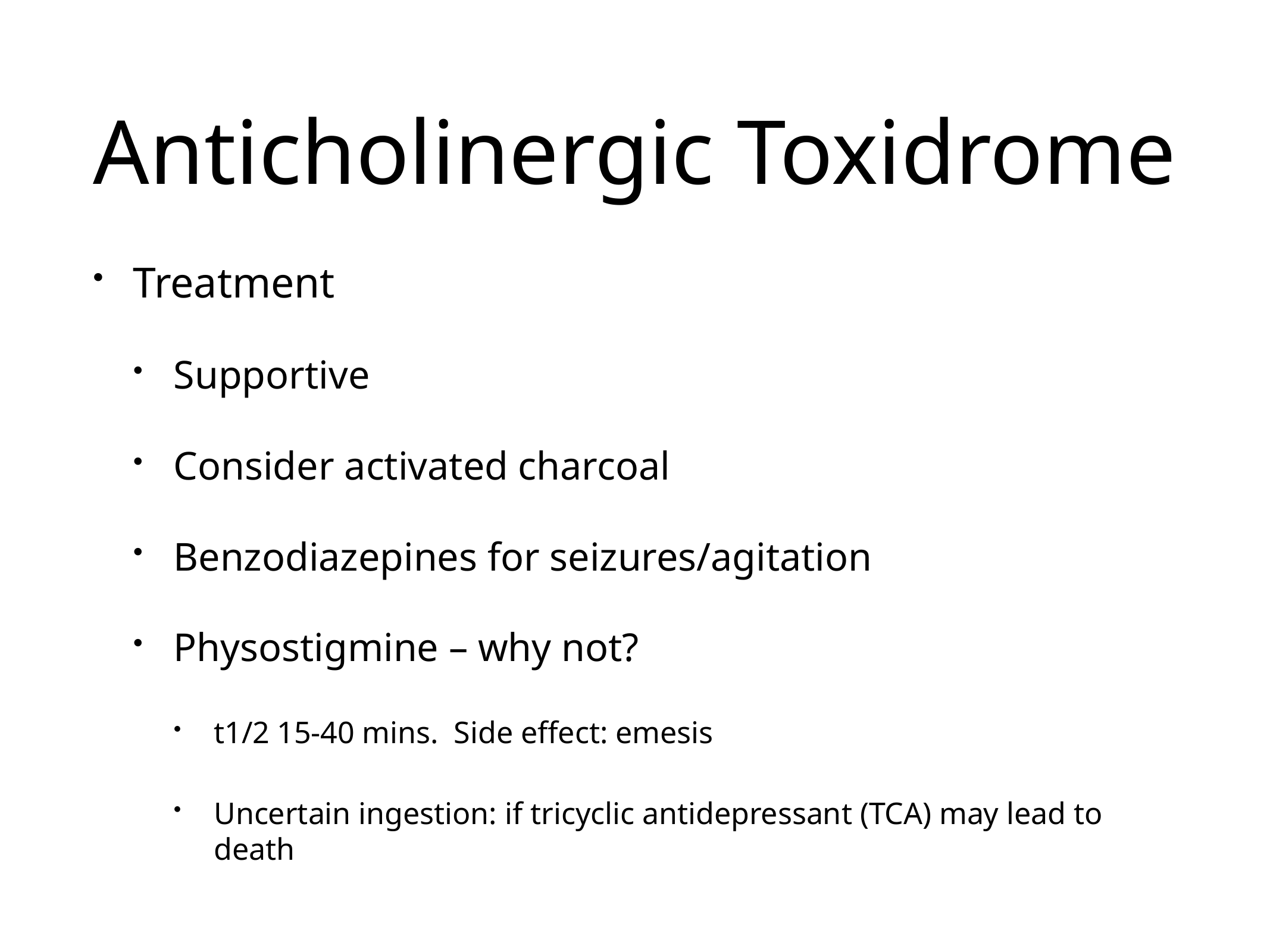

# Anticholinergic Toxidrome
Treatment
Supportive
Consider activated charcoal
Benzodiazepines for seizures/agitation
Physostigmine – why not?
t1/2 15-40 mins. Side effect: emesis
Uncertain ingestion: if tricyclic antidepressant (TCA) may lead to death

## Slide 9
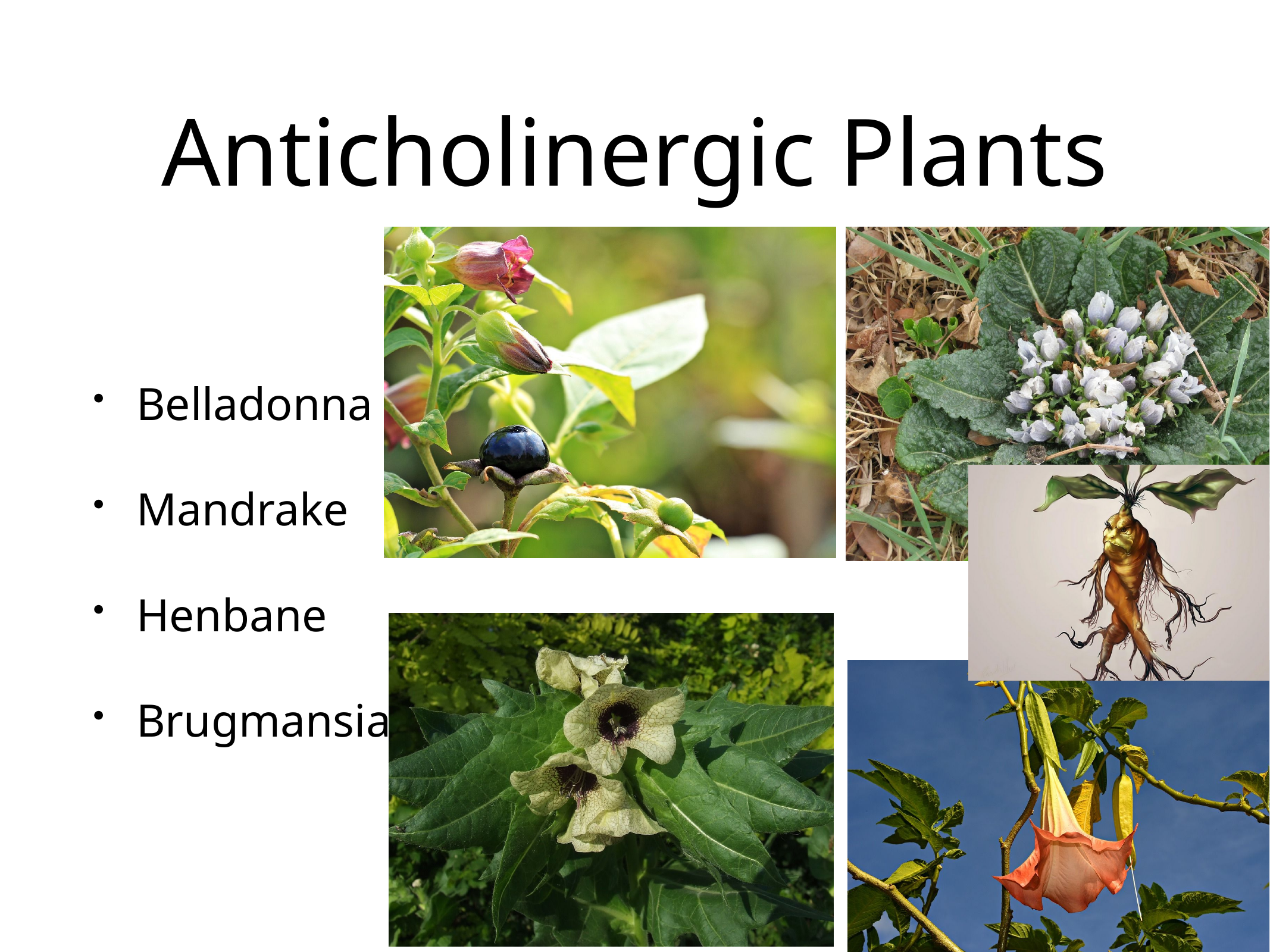

# Anticholinergic Plants
Belladonna
Mandrake
Henbane
Brugmansia

## Slide 10
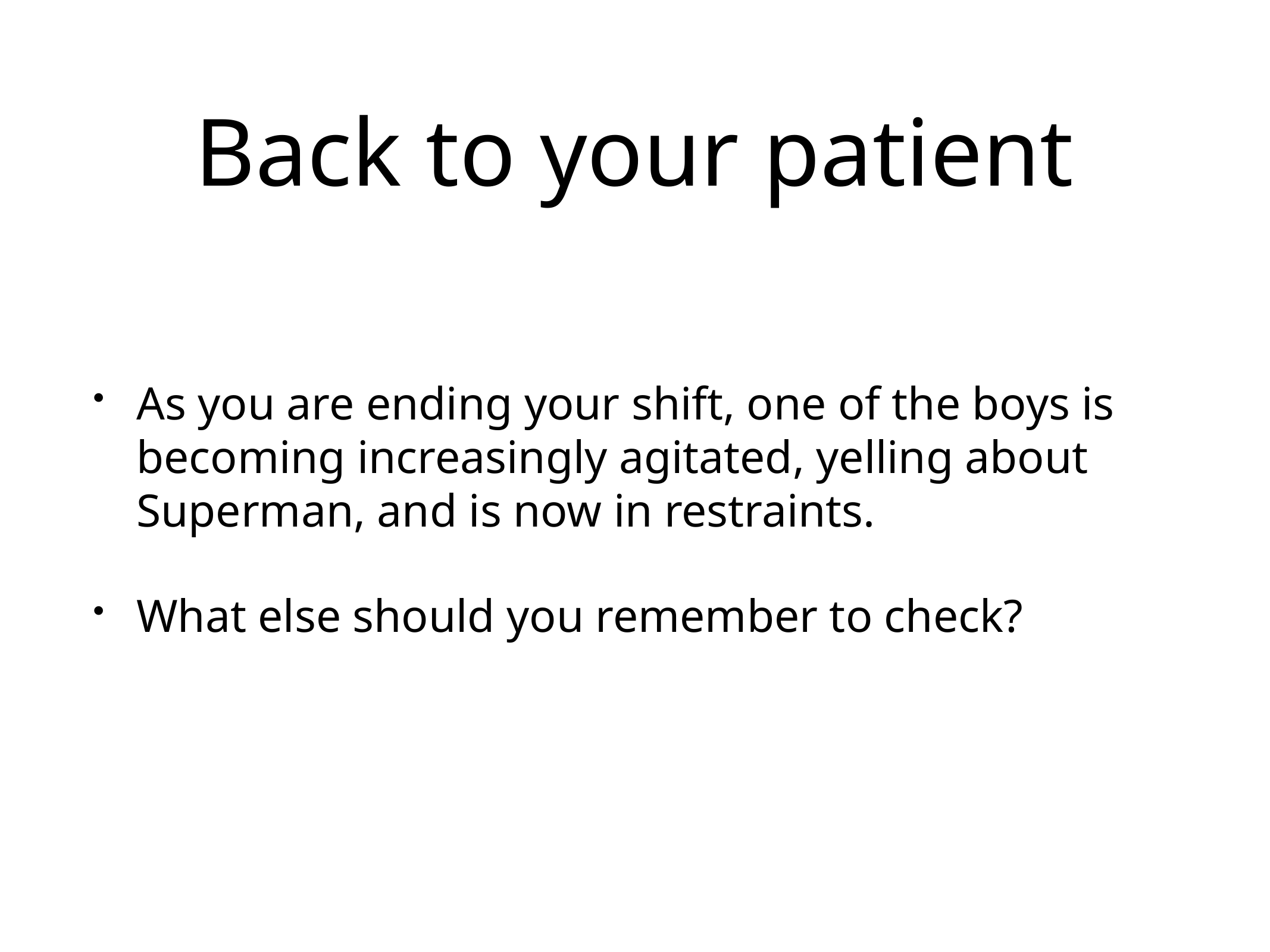

# Back to your patient
As you are ending your shift, one of the boys is becoming increasingly agitated, yelling about Superman, and is now in restraints.
What else should you remember to check?

## Slide 11
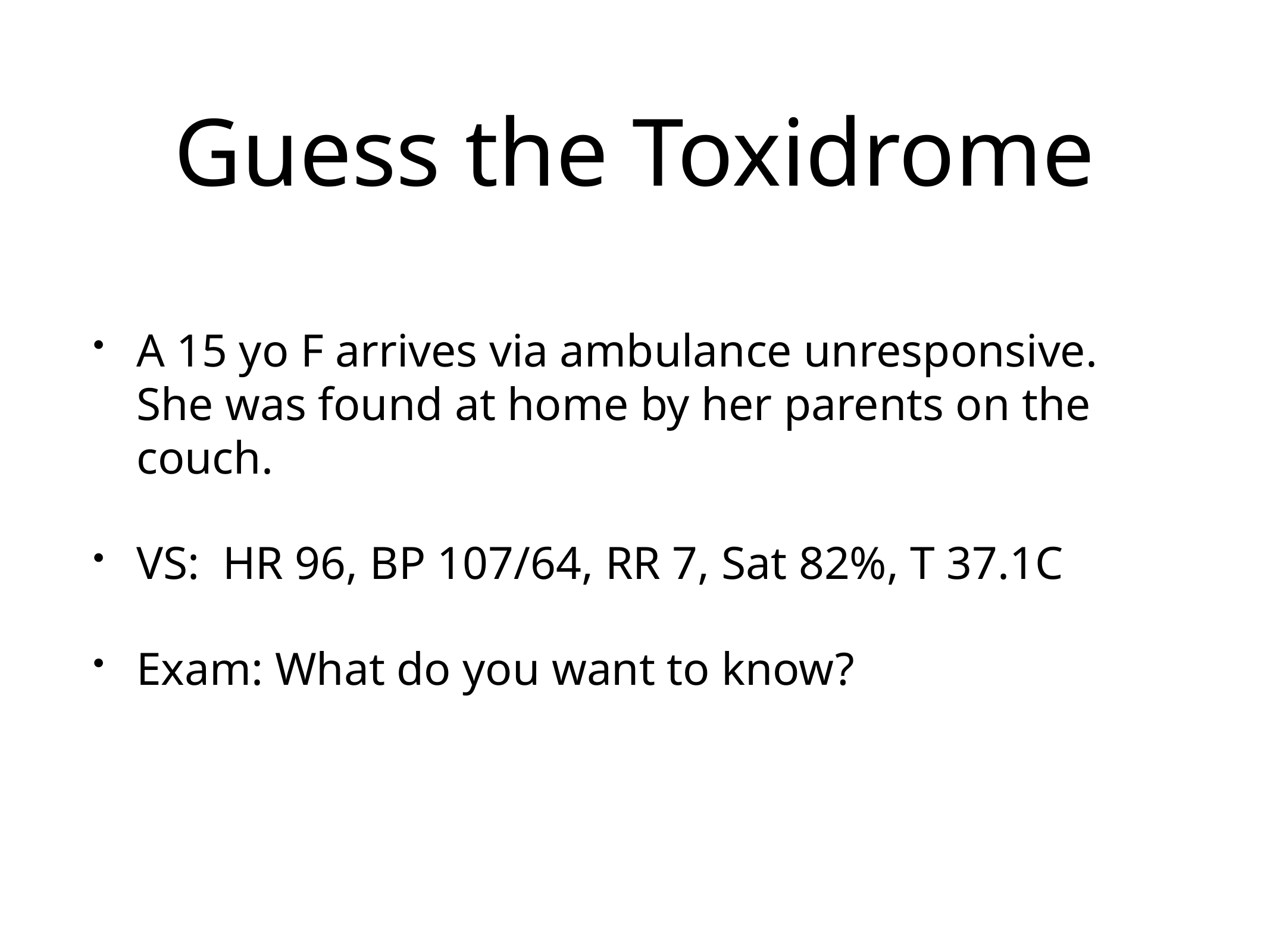

# Guess the Toxidrome
A 15 yo F arrives via ambulance unresponsive. She was found at home by her parents on the couch.
VS: HR 96, BP 107/64, RR 7, Sat 82%, T 37.1C
Exam: What do you want to know?

## Slide 12
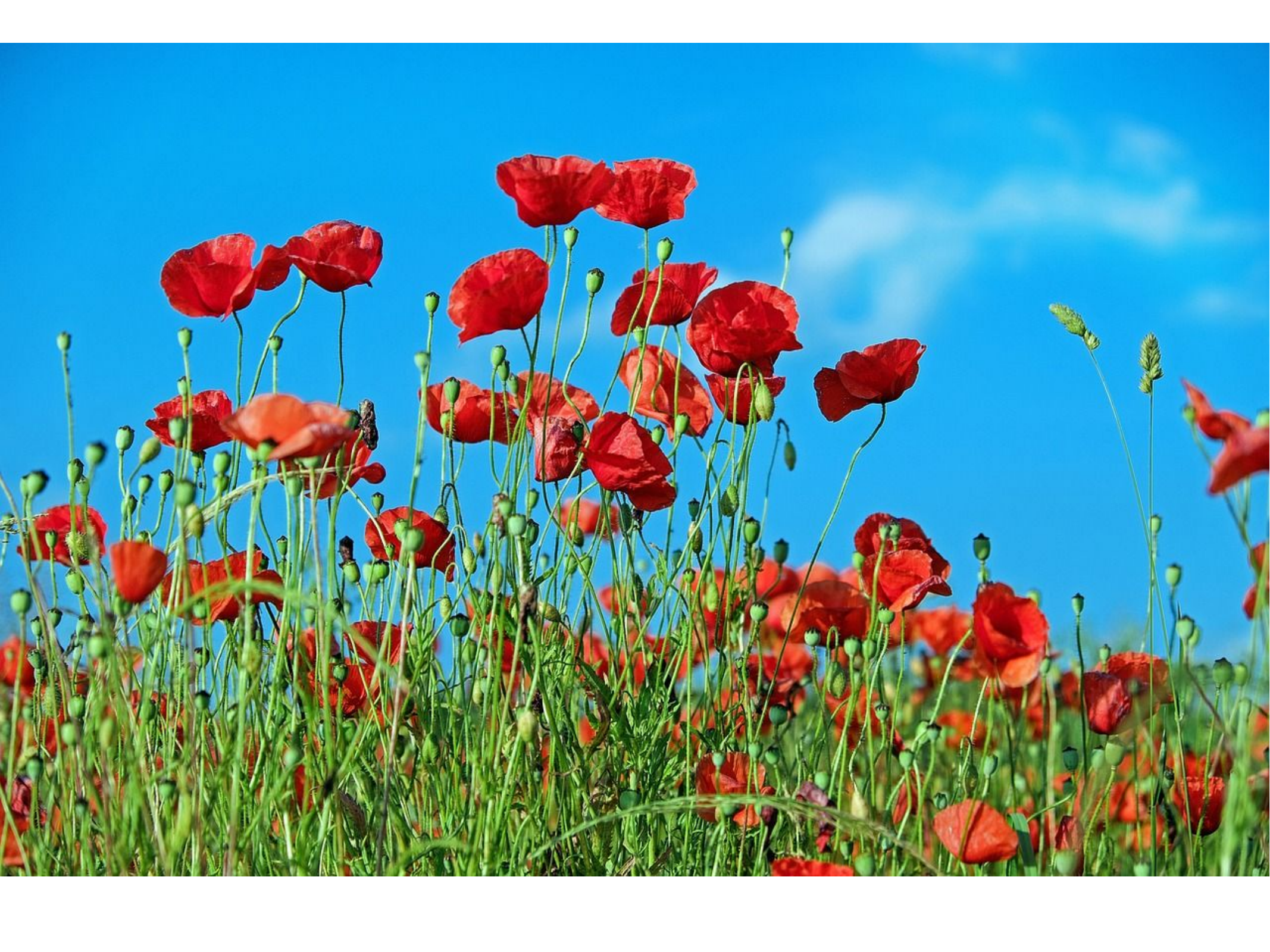

## Slide 13
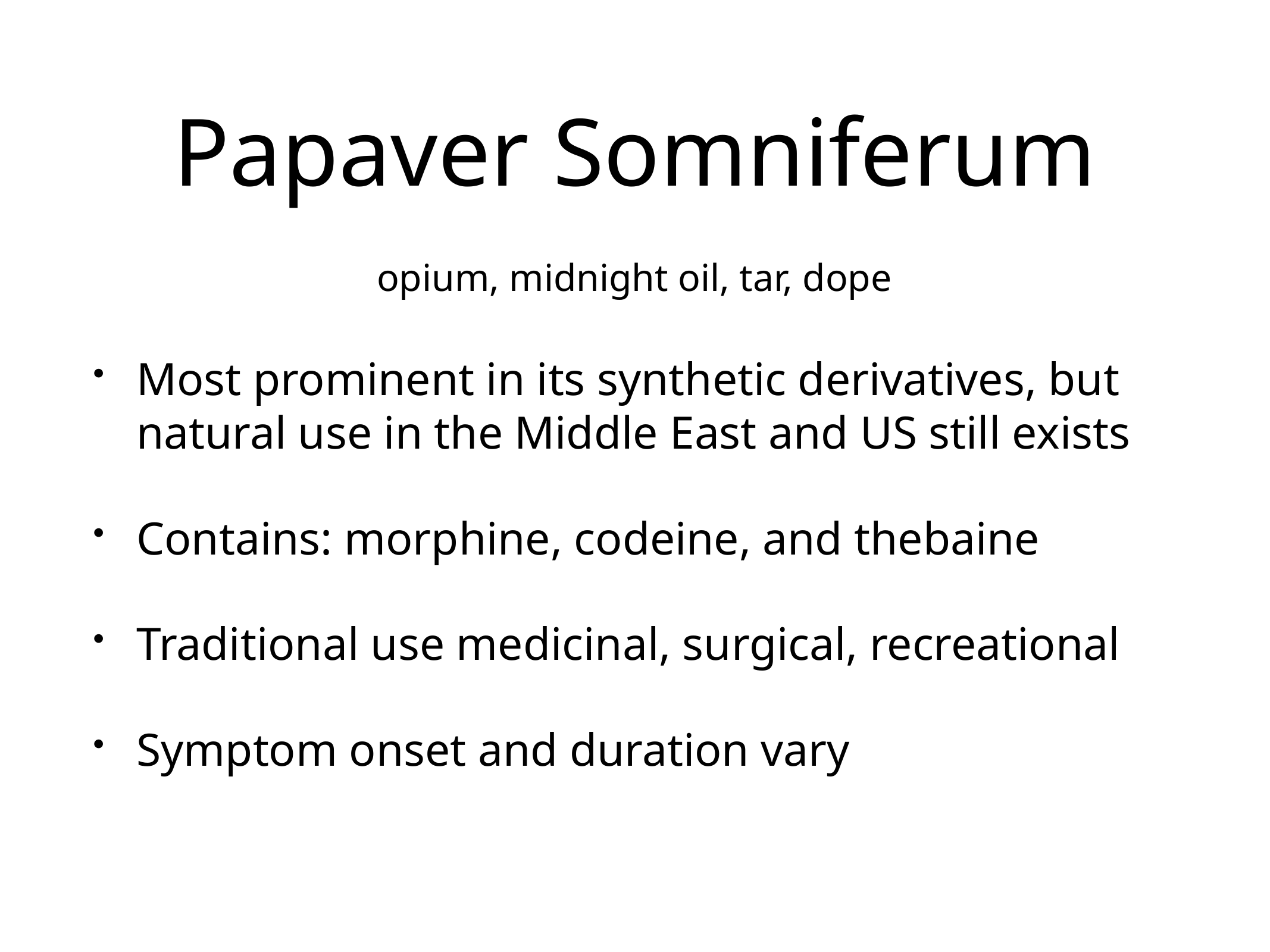

# Papaver Somniferum
opium, midnight oil, tar, dope
Most prominent in its synthetic derivatives, but natural use in the Middle East and US still exists
Contains: morphine, codeine, and thebaine
Traditional use medicinal, surgical, recreational
Symptom onset and duration vary

## Slide 14
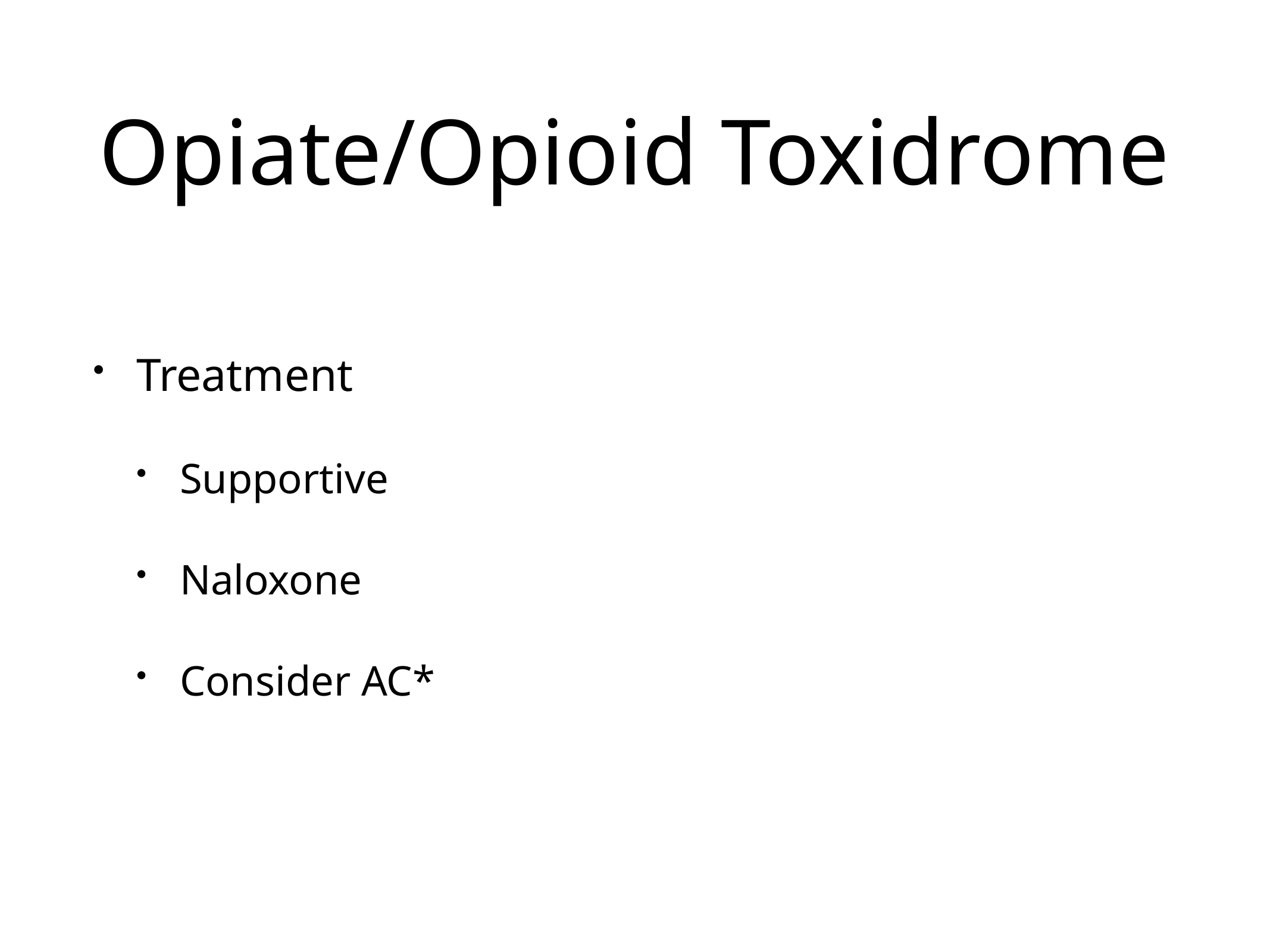

# Opiate/Opioid Toxidrome
Treatment
Supportive
Naloxone
Consider AC*

## Slide 15
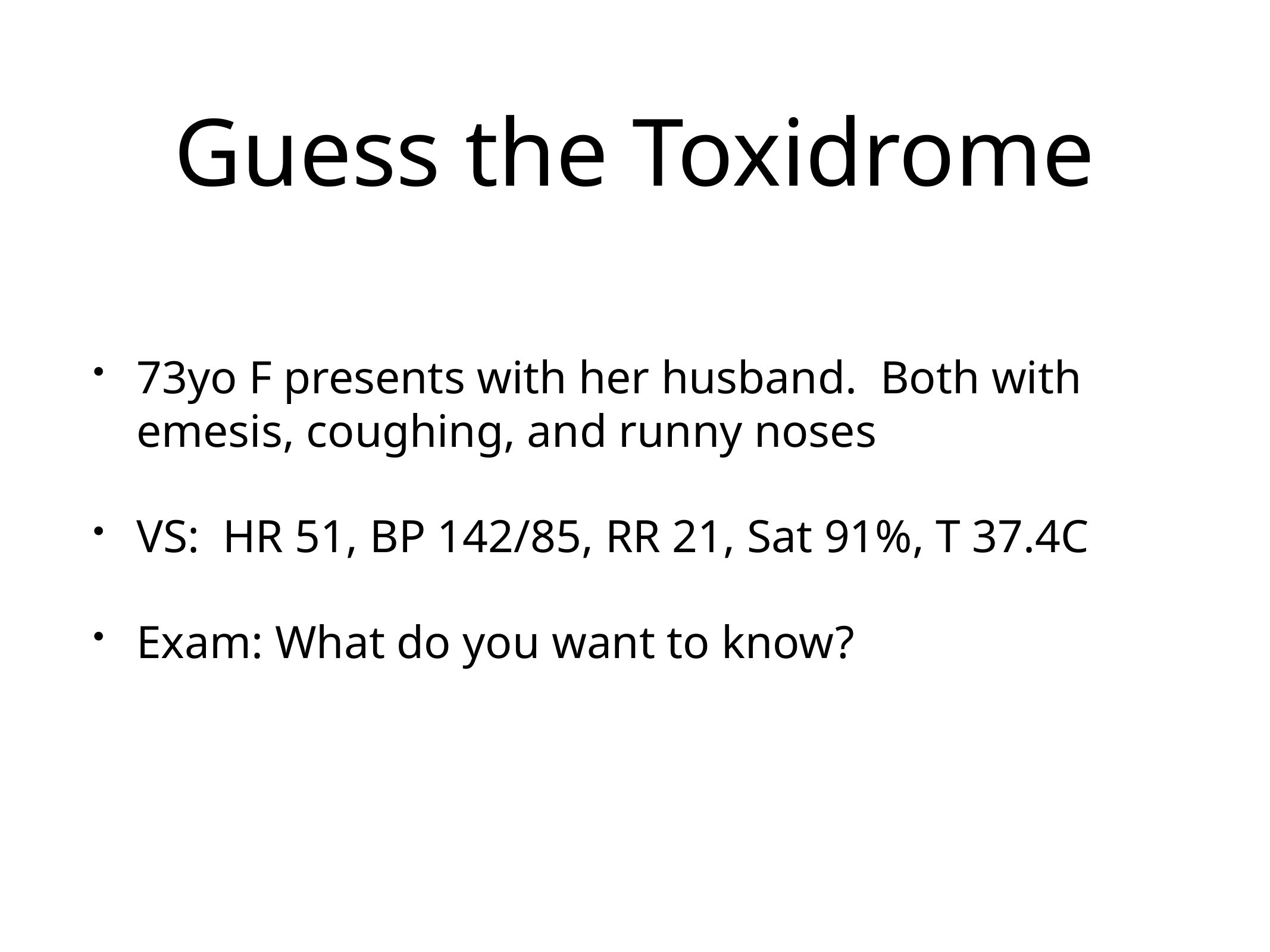

# Guess the Toxidrome
73yo F presents with her husband. Both with emesis, coughing, and runny noses
VS: HR 51, BP 142/85, RR 21, Sat 91%, T 37.4C
Exam: What do you want to know?

## Slide 16
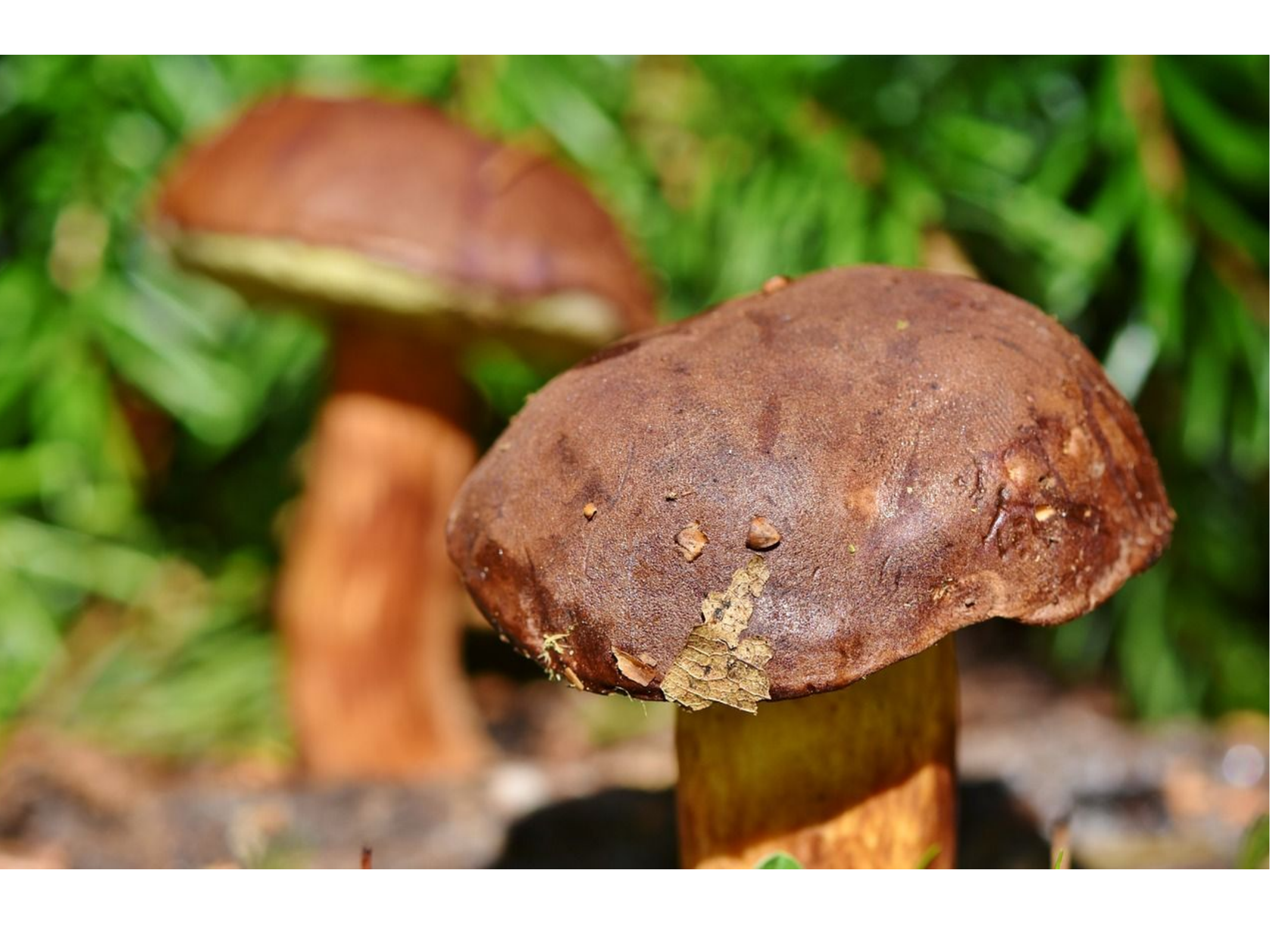

## Slide 17
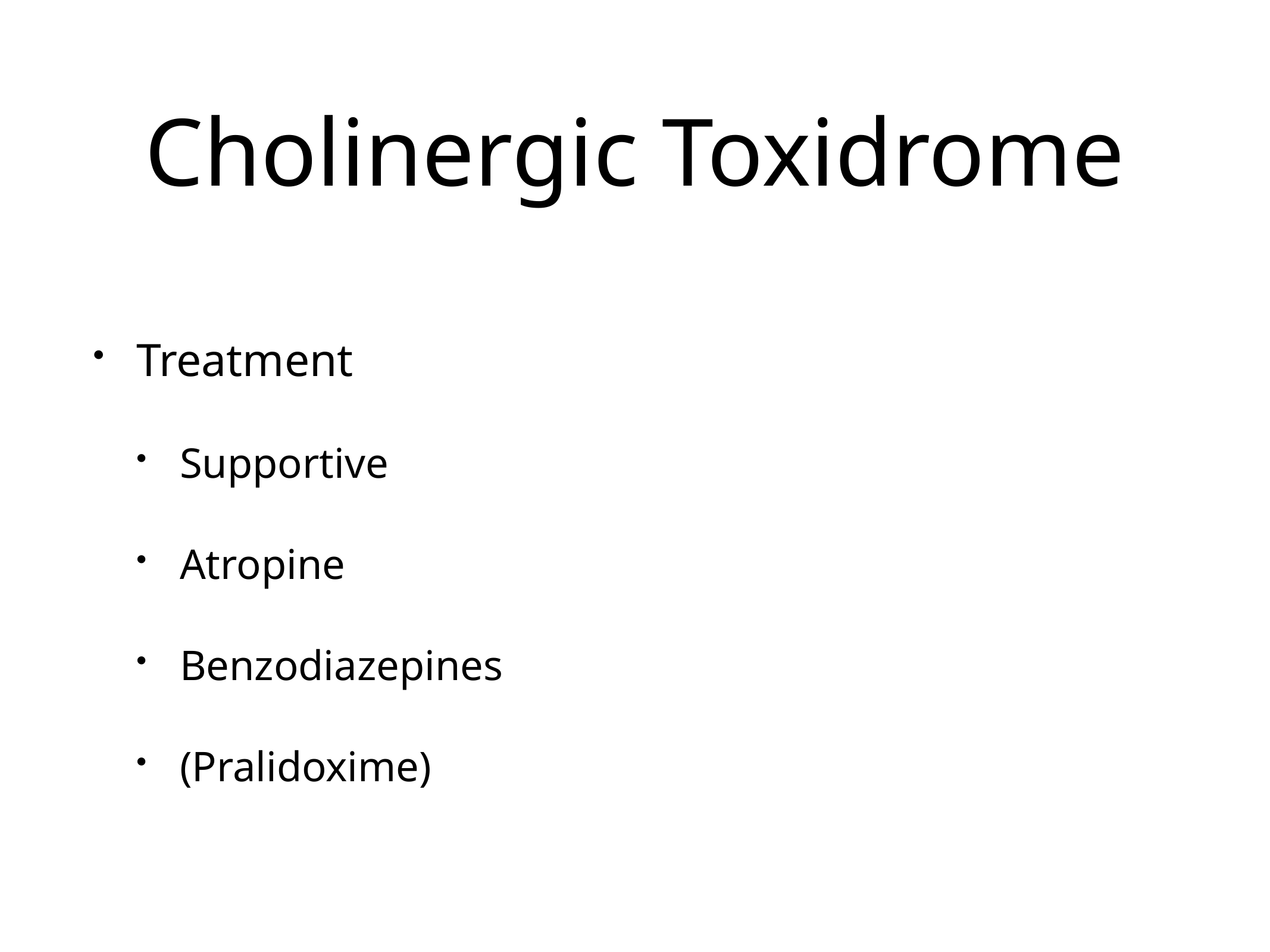

# Cholinergic Toxidrome
Treatment
Supportive
Atropine
Benzodiazepines
(Pralidoxime)

## Slide 18
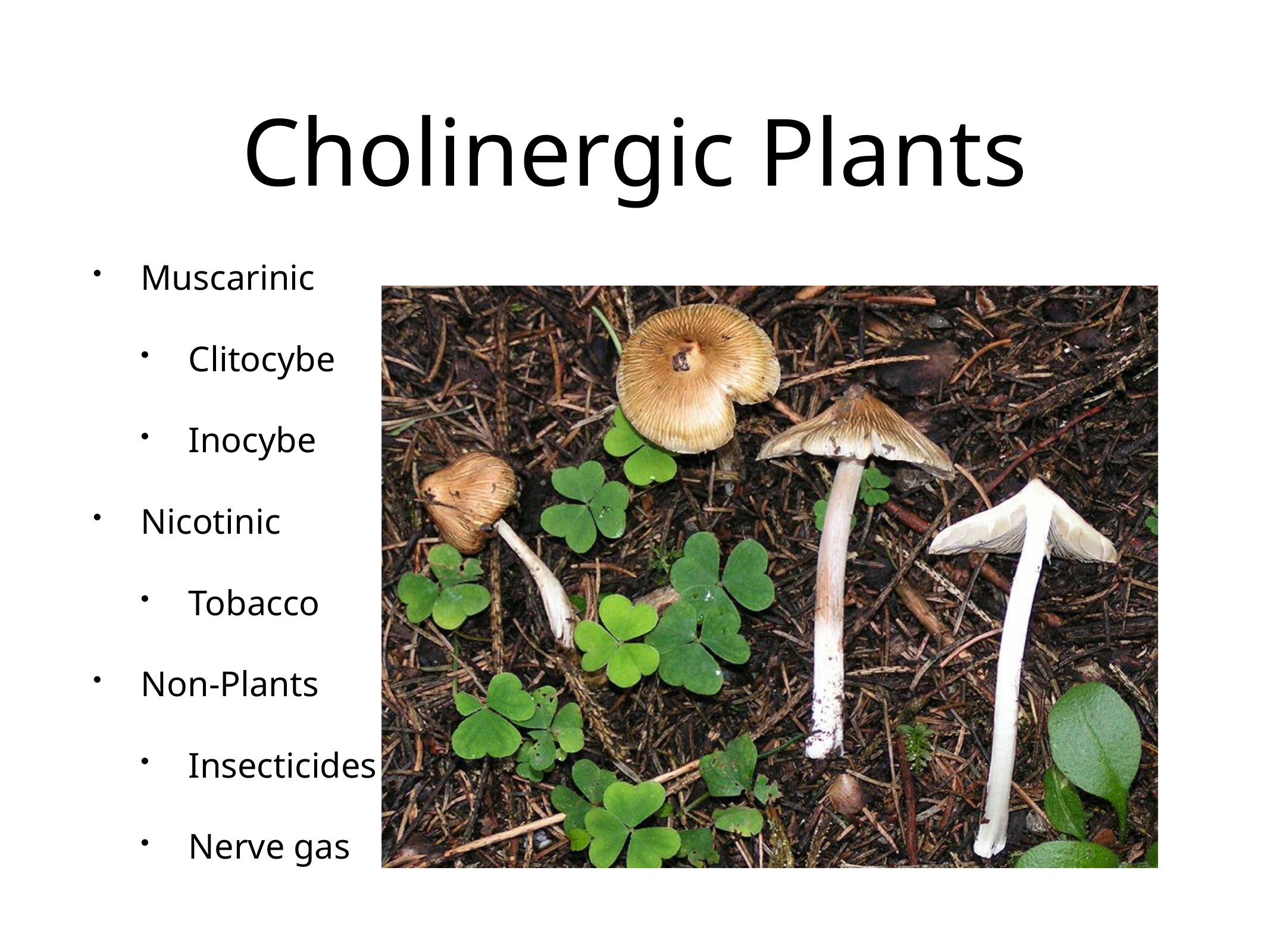

# Cholinergic Plants
Muscarinic
Clitocybe
Inocybe
Nicotinic
Tobacco
Non-Plants
Insecticides
Nerve gas

## Slide 19
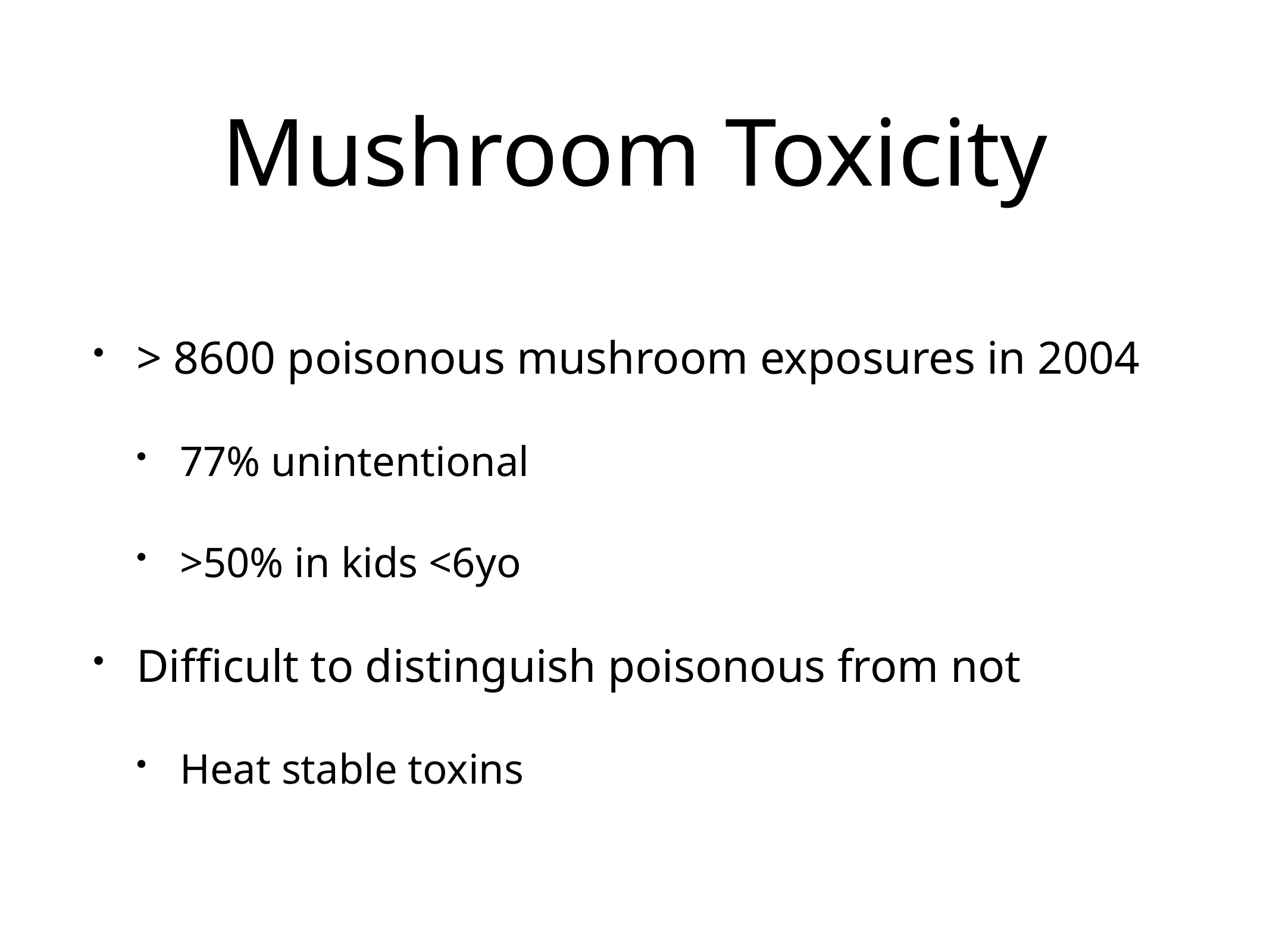

# Mushroom Toxicity
> 8600 poisonous mushroom exposures in 2004
77% unintentional
>50% in kids <6yo
Difficult to distinguish poisonous from not
Heat stable toxins

## Slide 20
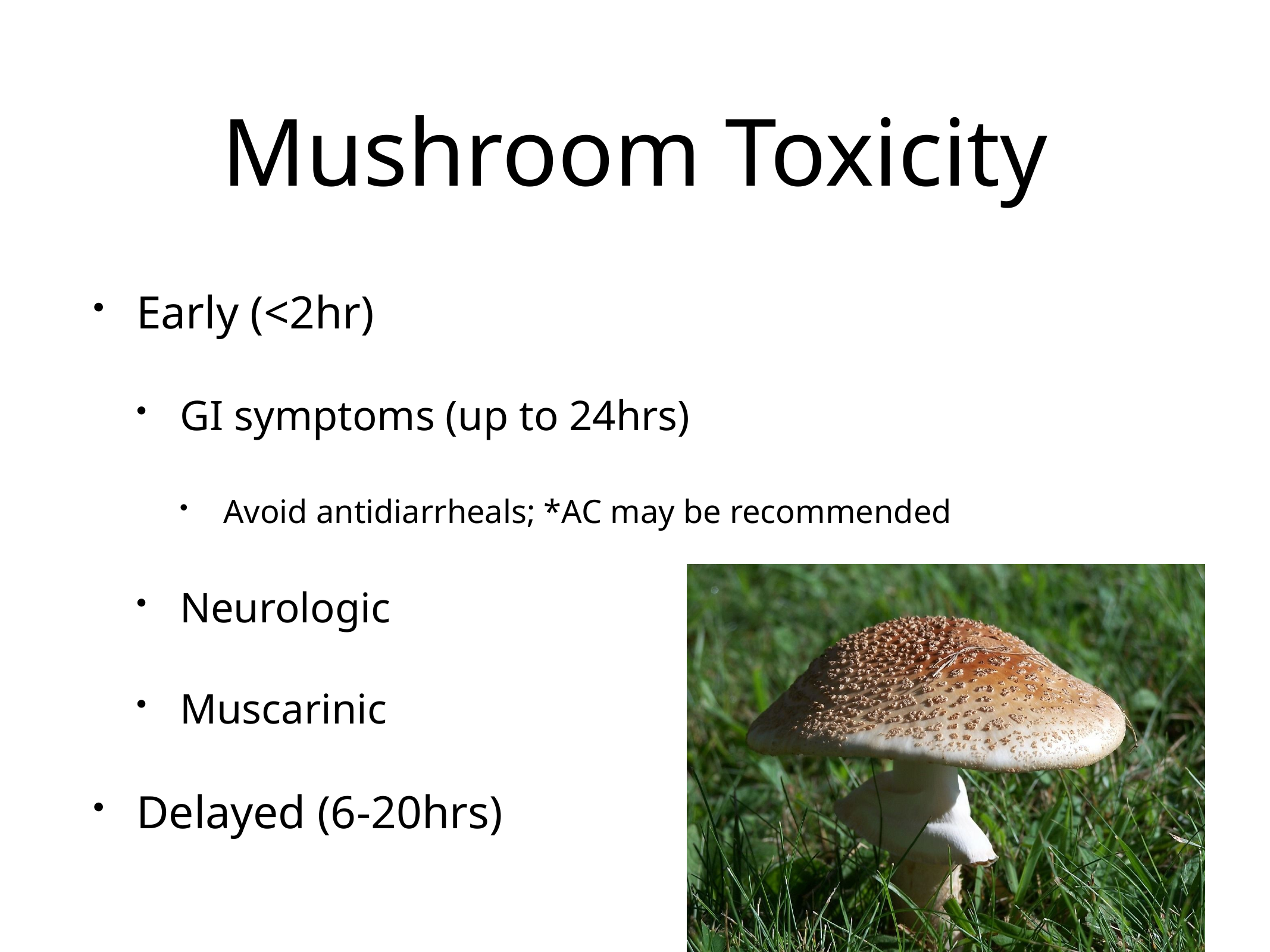

# Mushroom Toxicity
Early (<2hr)
GI symptoms (up to 24hrs)
Avoid antidiarrheals; *AC may be recommended
Neurologic
Muscarinic
Delayed (6-20hrs)

## Slide 21
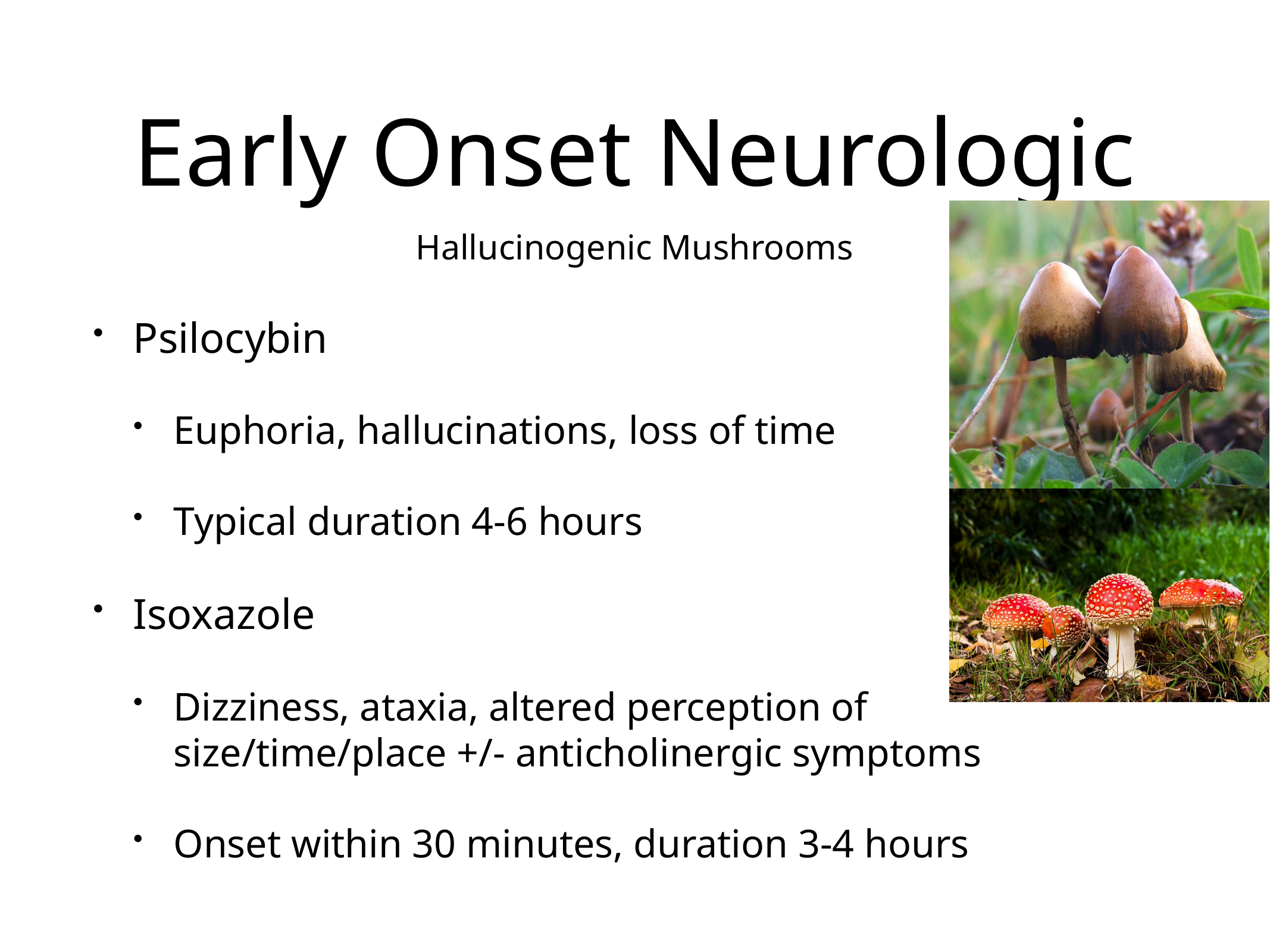

# Early Onset Neurologic
Hallucinogenic Mushrooms
Psilocybin
Euphoria, hallucinations, loss of time
Typical duration 4-6 hours
Isoxazole
Dizziness, ataxia, altered perception of size/time/place +/- anticholinergic symptoms
Onset within 30 minutes, duration 3-4 hours

## Slide 22
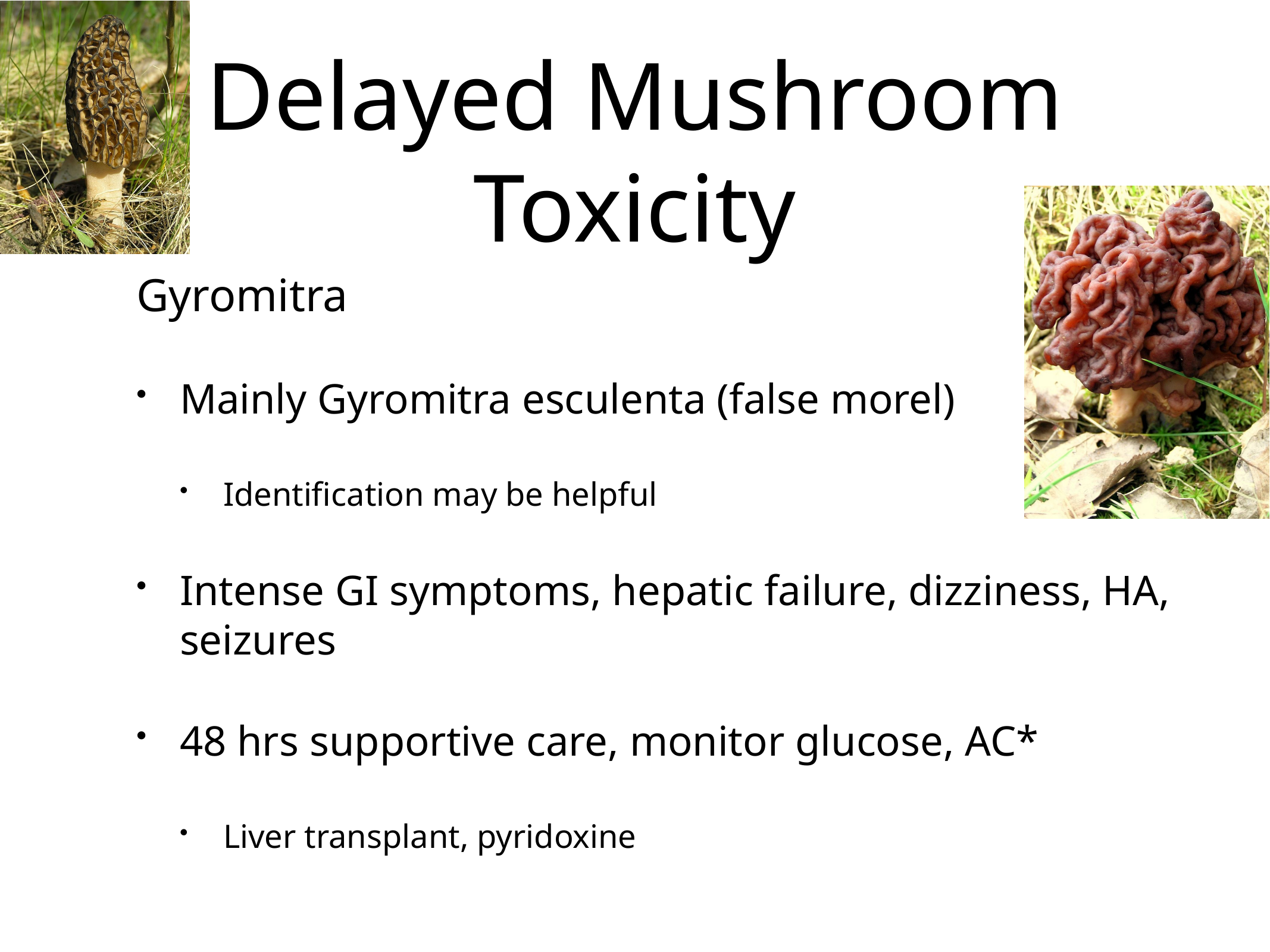

# Delayed Mushroom Toxicity
	Gyromitra
Mainly Gyromitra esculenta (false morel)
Identification may be helpful
Intense GI symptoms, hepatic failure, dizziness, HA, seizures
48 hrs supportive care, monitor glucose, AC*
Liver transplant, pyridoxine

## Slide 23
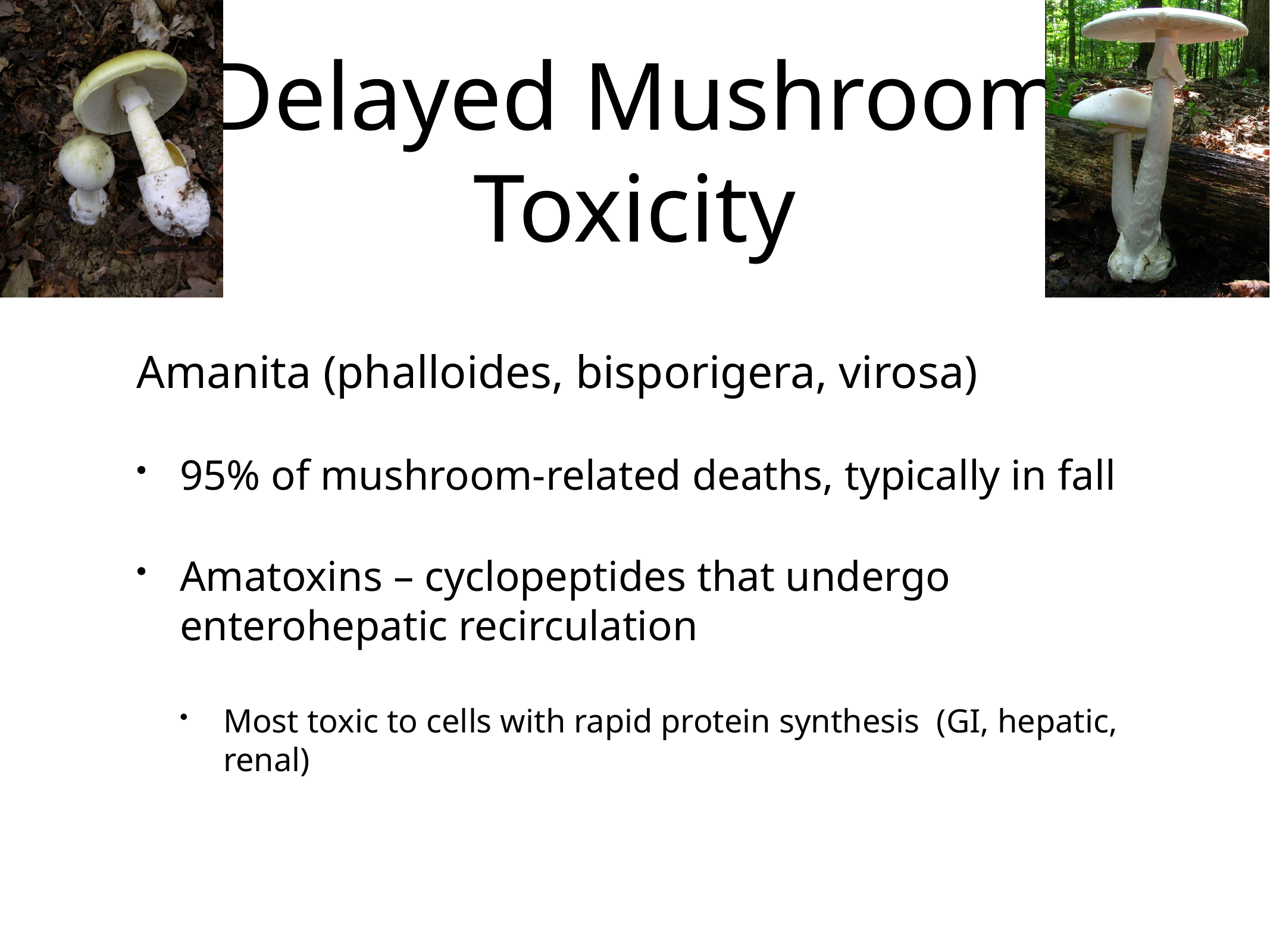

# Delayed Mushroom Toxicity
	Amanita (phalloides, bisporigera, virosa)
95% of mushroom-related deaths, typically in fall
Amatoxins – cyclopeptides that undergo enterohepatic recirculation
Most toxic to cells with rapid protein synthesis (GI, hepatic, renal)

## Slide 24
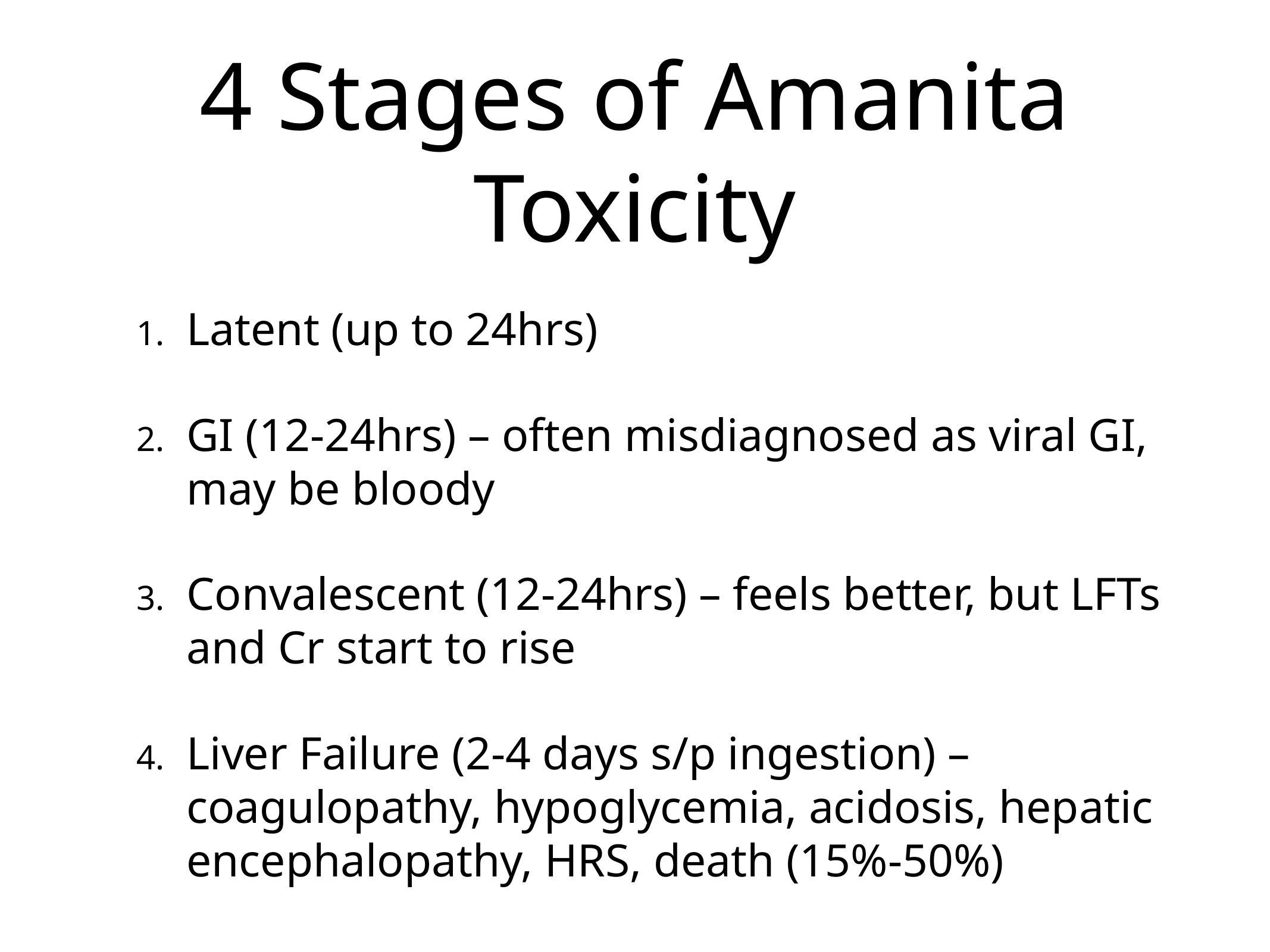

# 4 Stages of Amanita Toxicity
Latent (up to 24hrs)
GI (12-24hrs) – often misdiagnosed as viral GI, may be bloody
Convalescent (12-24hrs) – feels better, but LFTs and Cr start to rise
Liver Failure (2-4 days s/p ingestion) – coagulopathy, hypoglycemia, acidosis, hepatic encephalopathy, HRS, death (15%-50%)

## Slide 25
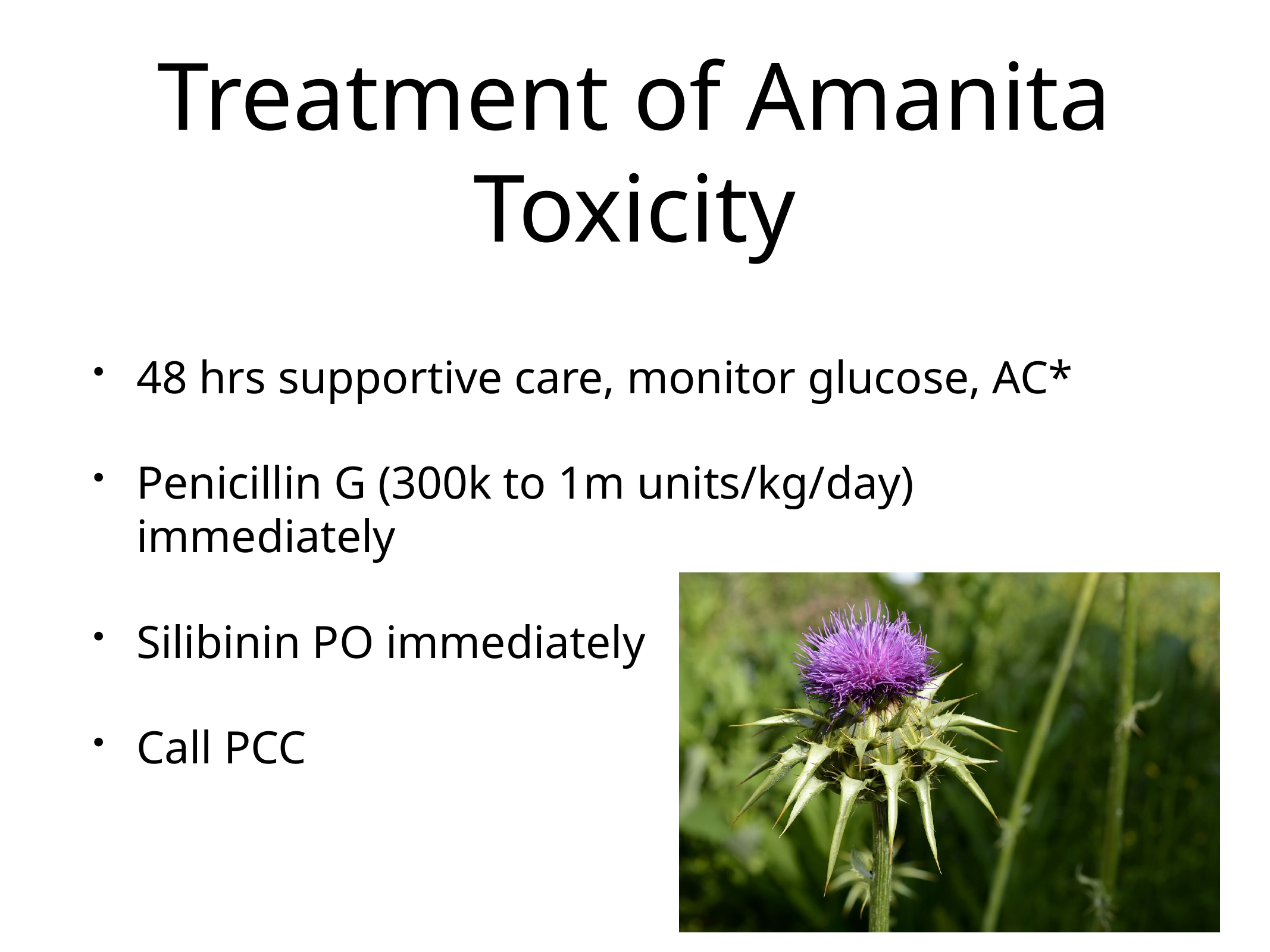

# Treatment of Amanita Toxicity
48 hrs supportive care, monitor glucose, AC*
Penicillin G (300k to 1m units/kg/day) immediately
Silibinin PO immediately
Call PCC

## Slide 26
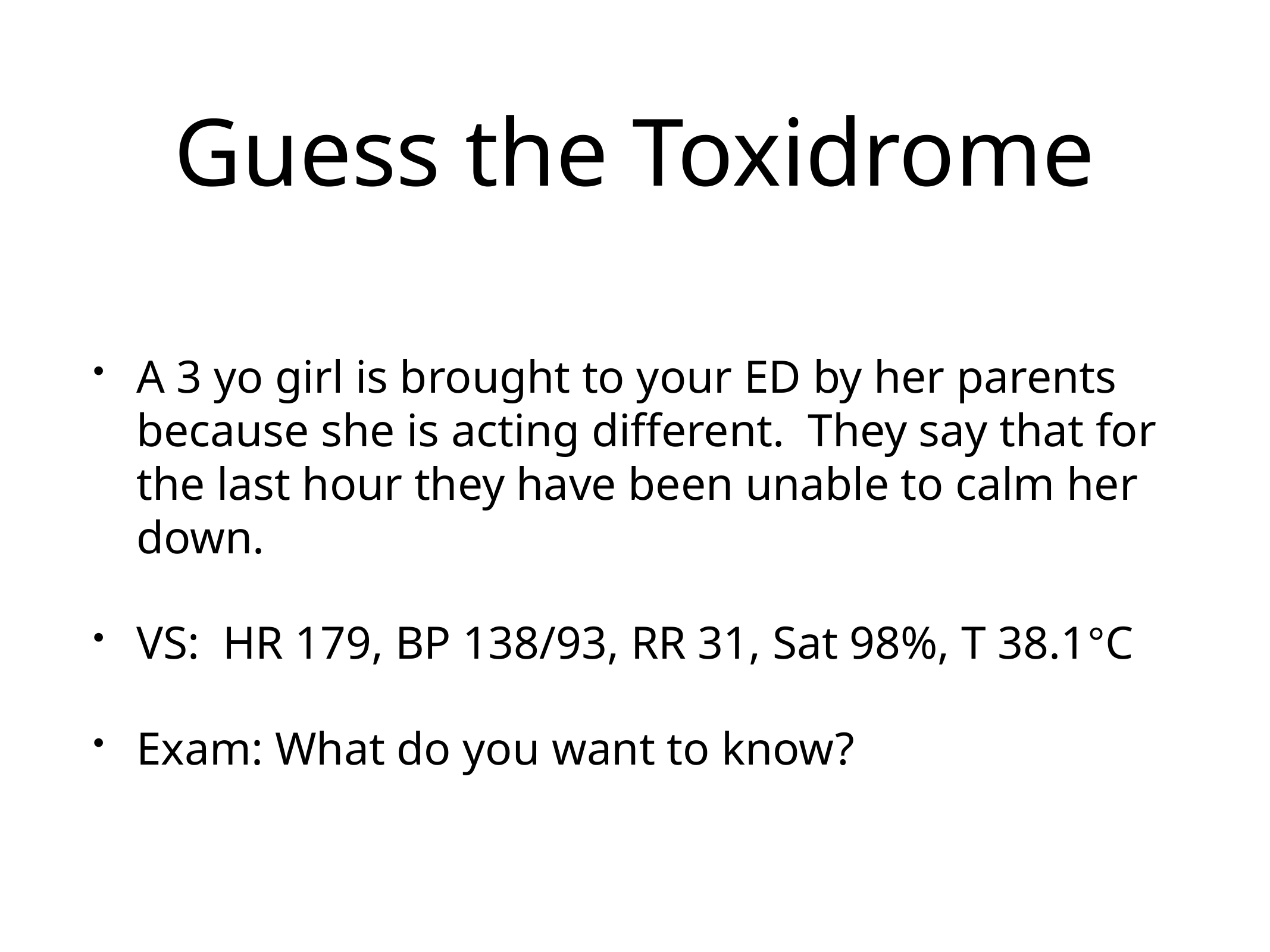

# Guess the Toxidrome
A 3 yo girl is brought to your ED by her parents because she is acting different. They say that for the last hour they have been unable to calm her down.
VS: HR 179, BP 138/93, RR 31, Sat 98%, T 38.1°C
Exam: What do you want to know?

## Slide 27
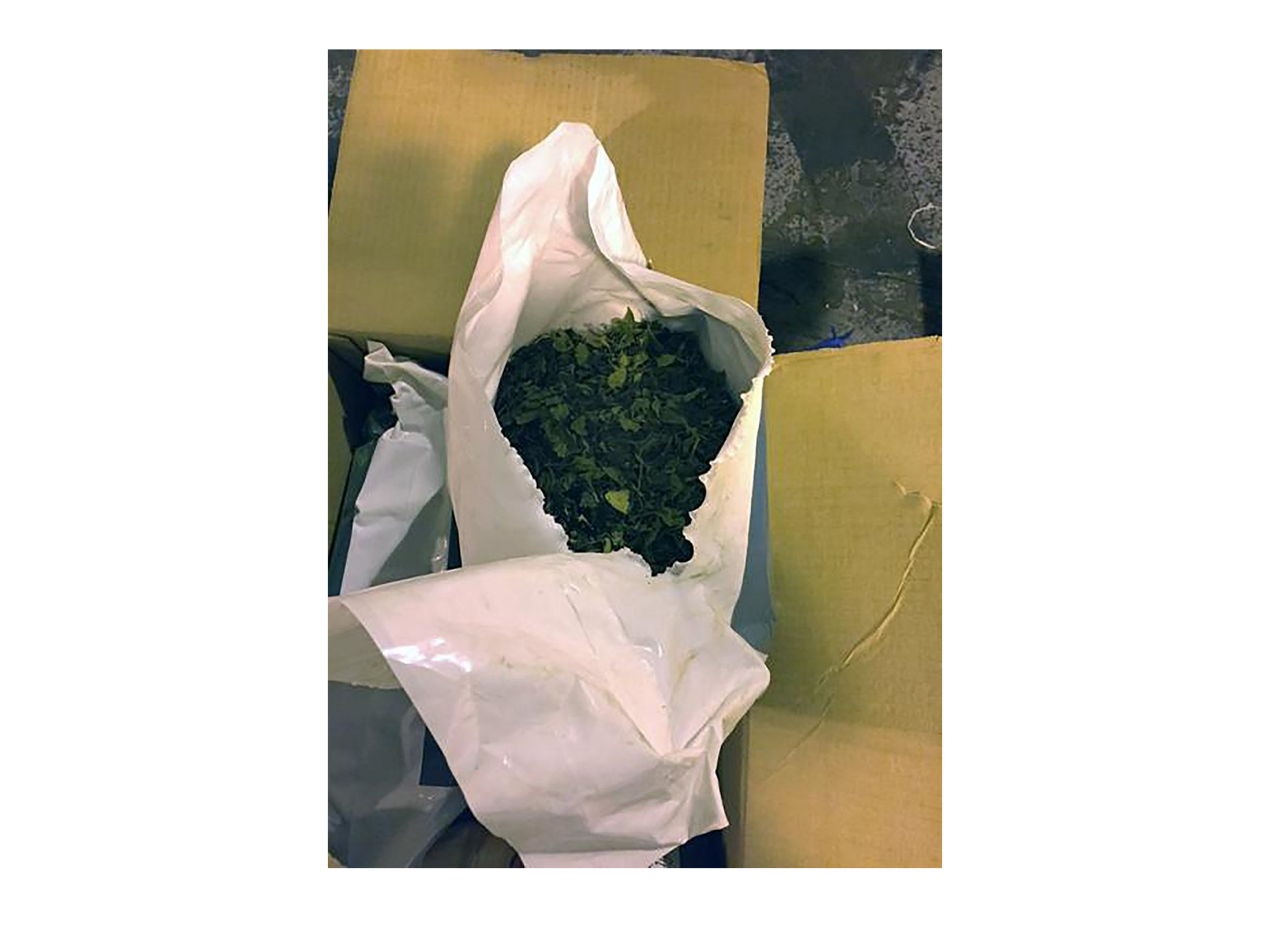

## Slide 28
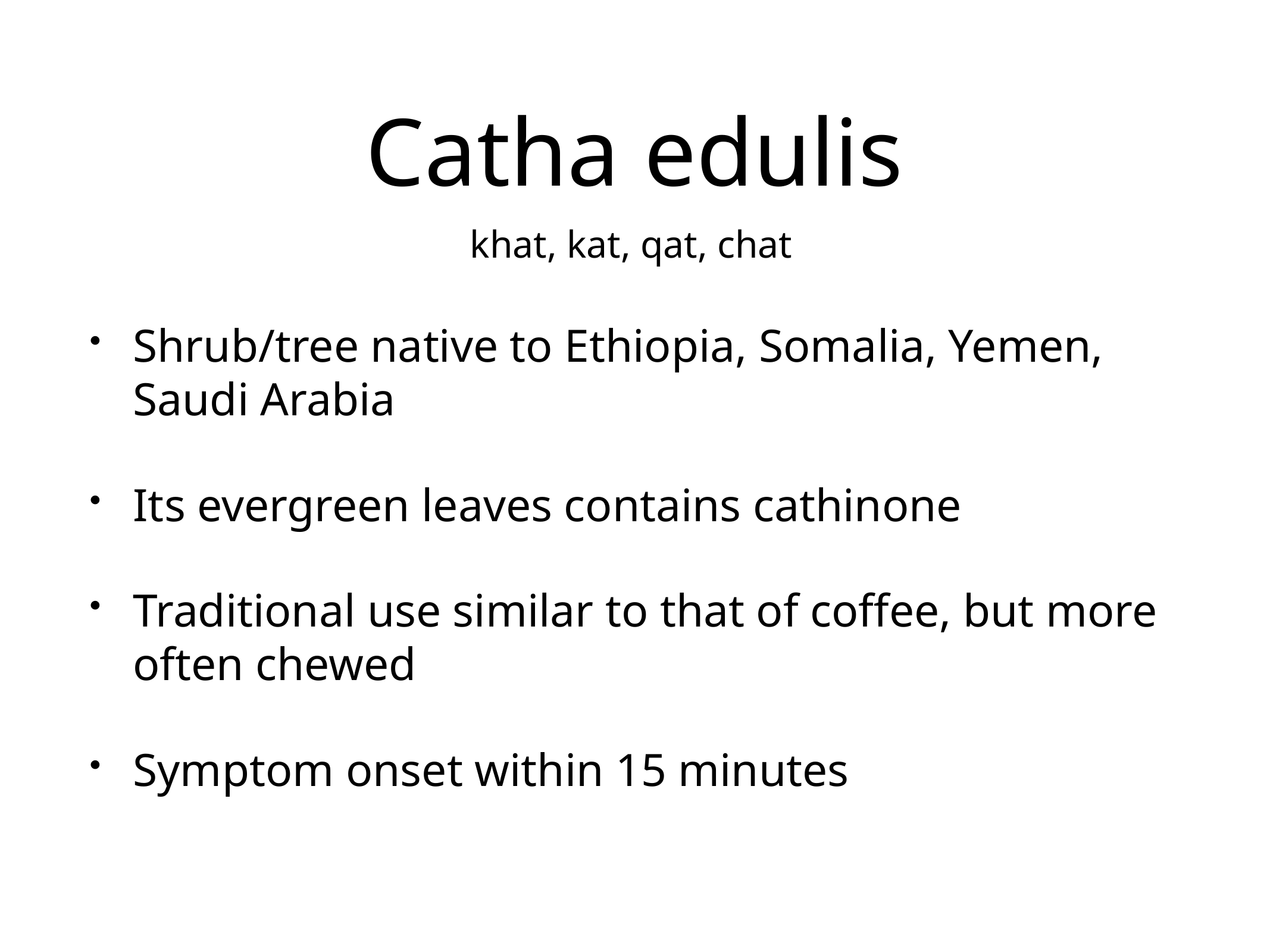

# Catha edulis
khat, kat, qat, chat
Shrub/tree native to Ethiopia, Somalia, Yemen, Saudi Arabia
Its evergreen leaves contains cathinone
Traditional use similar to that of coffee, but more often chewed
Symptom onset within 15 minutes

## Slide 29
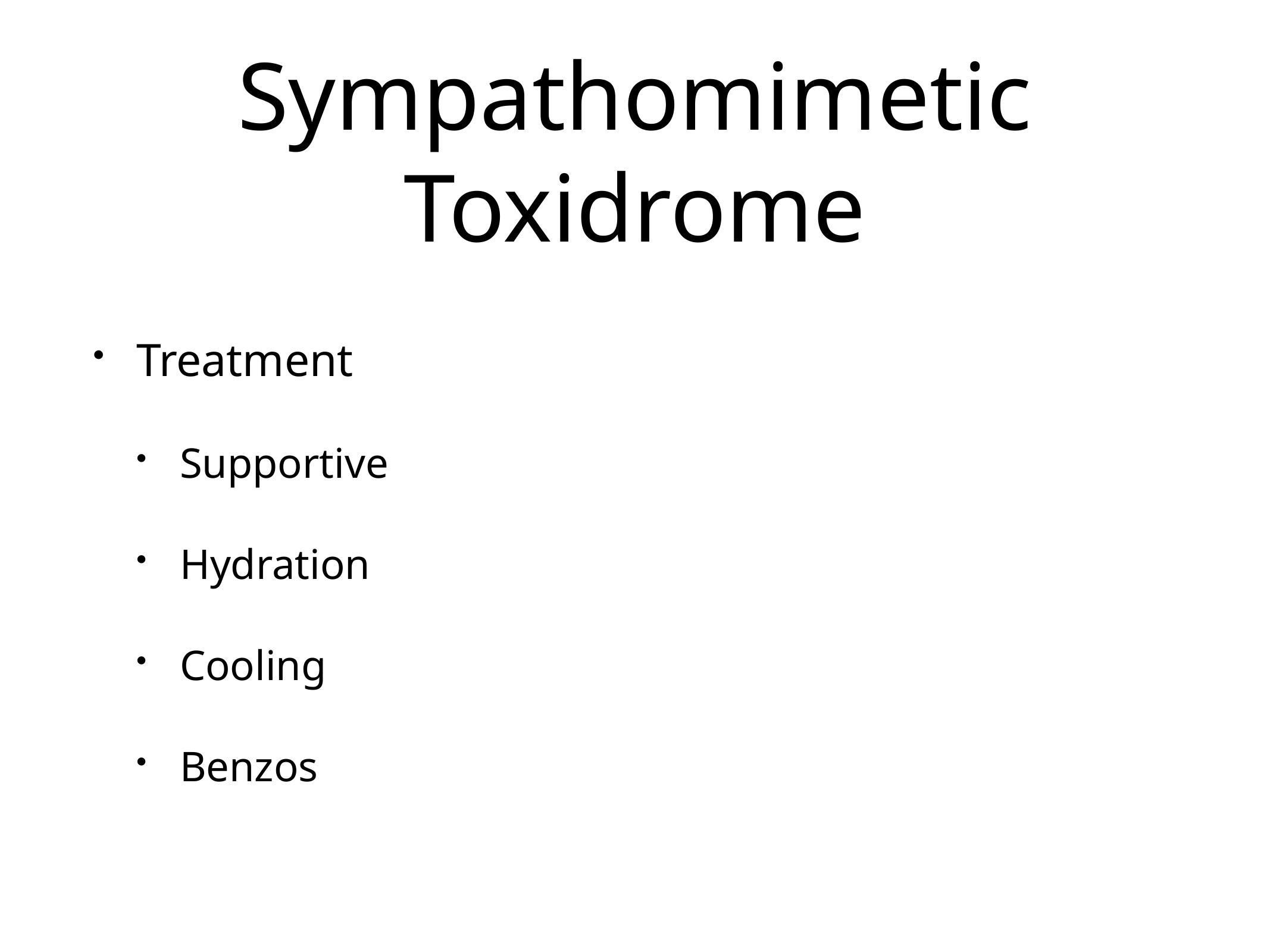

# Sympathomimetic Toxidrome
Treatment
Supportive
Hydration
Cooling
Benzos

## Slide 30
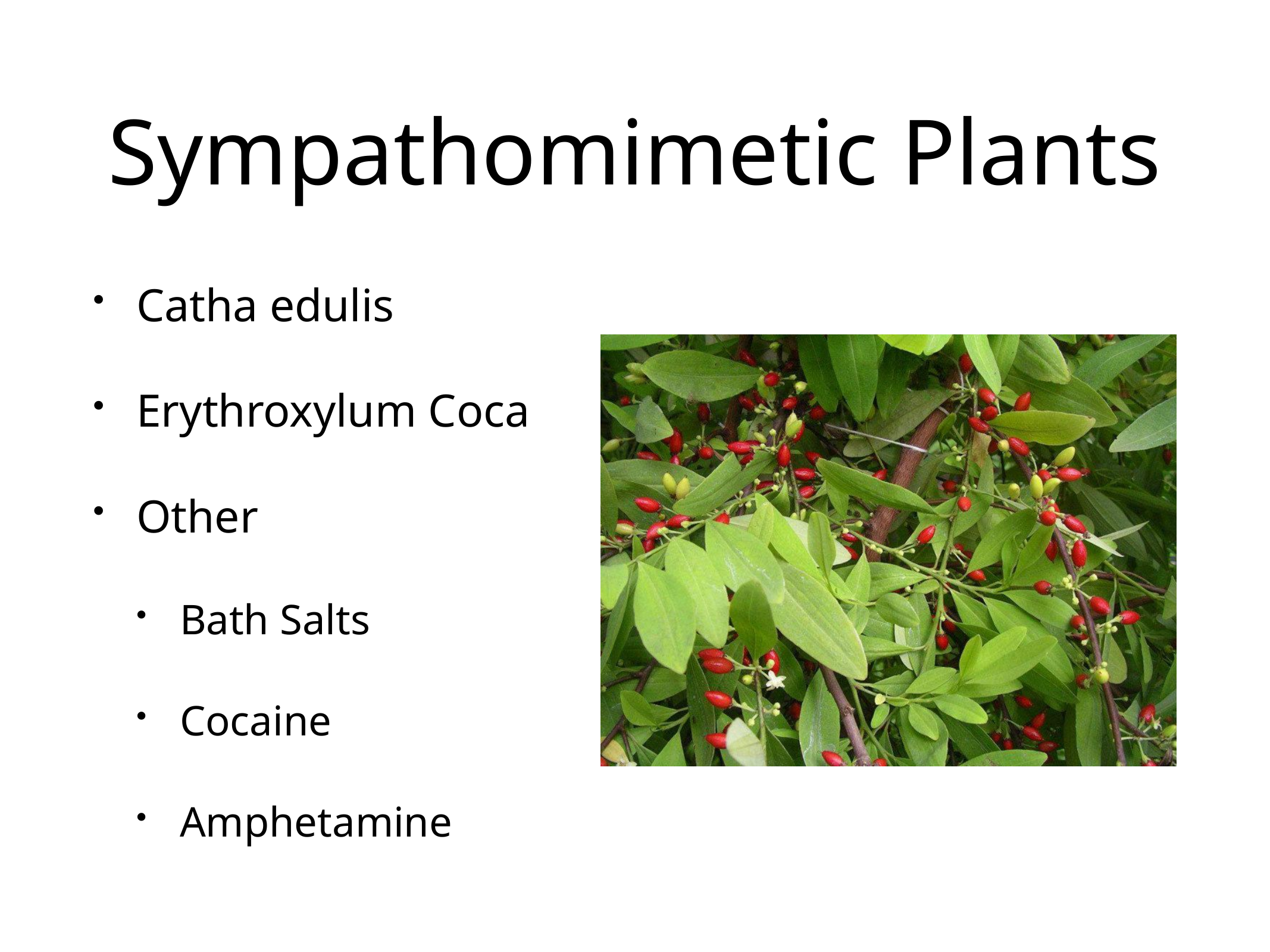

# Sympathomimetic Plants
Catha edulis
Erythroxylum Coca
Other
Bath Salts
Cocaine
Amphetamine

## Slide 31
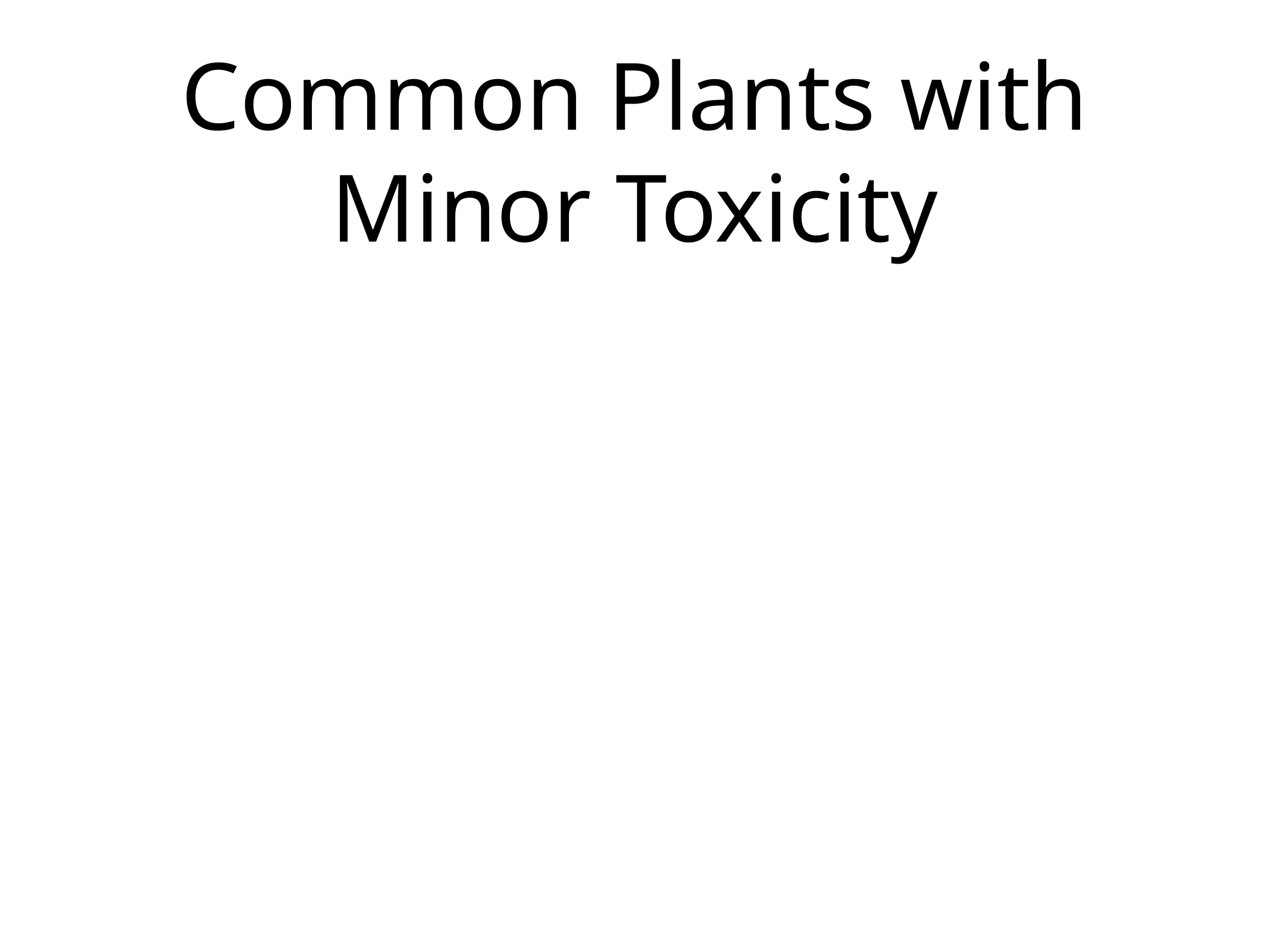

# Common Plants with Minor Toxicity

## Slide 32
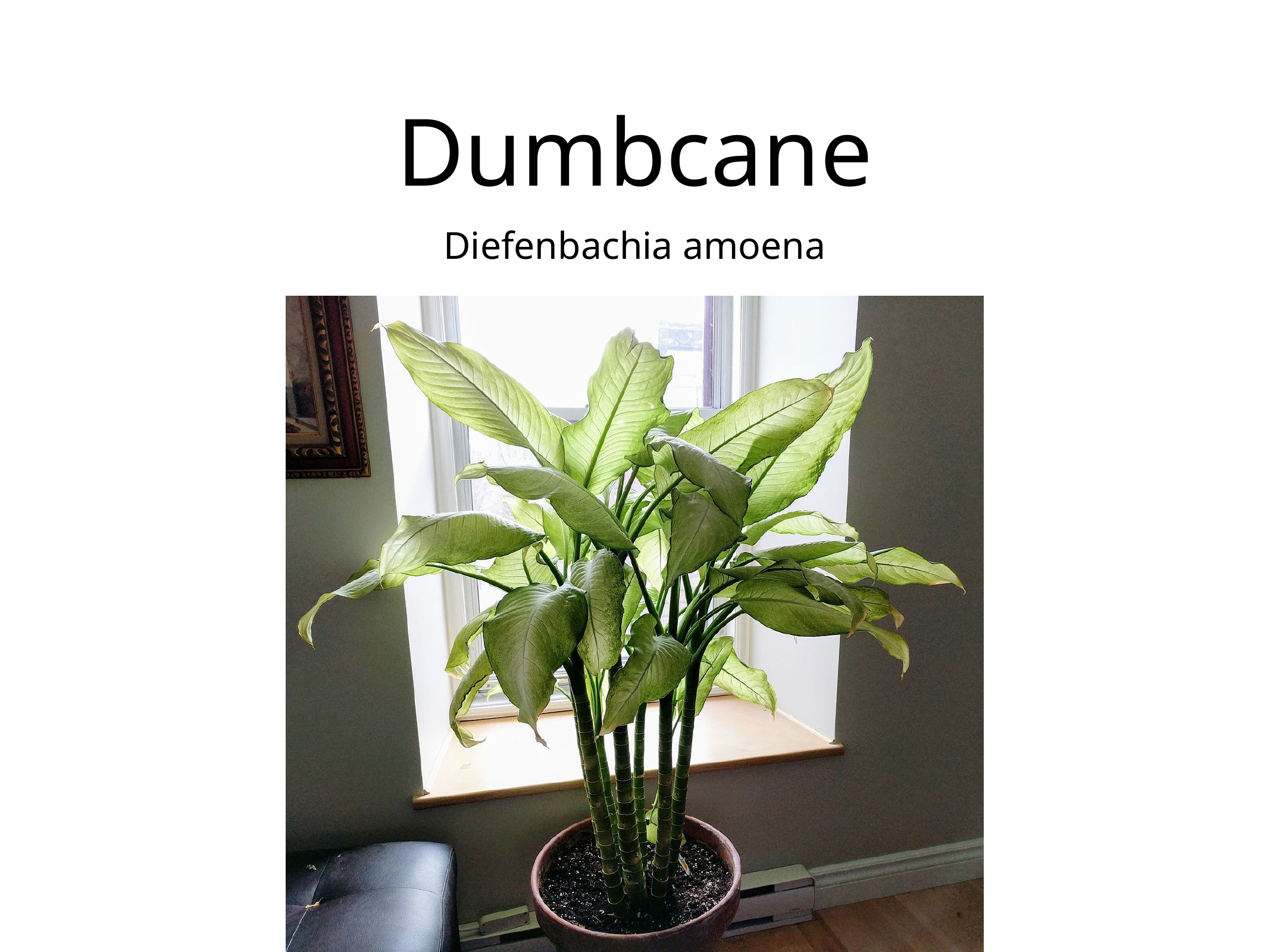

# Dumbcane
Diefenbachia amoena

## Slide 33
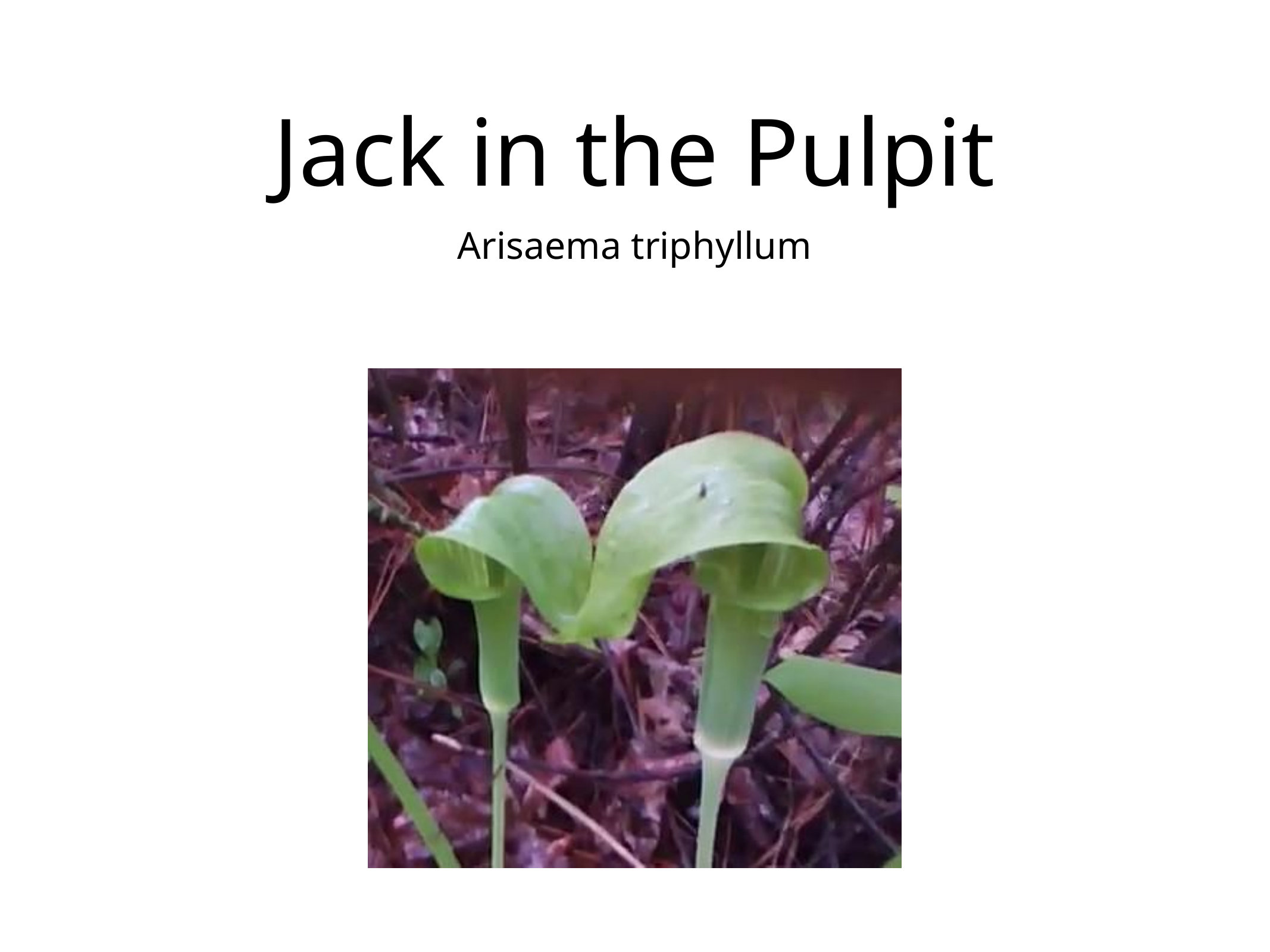

# Jack in the Pulpit
Arisaema triphyllum

## Slide 34
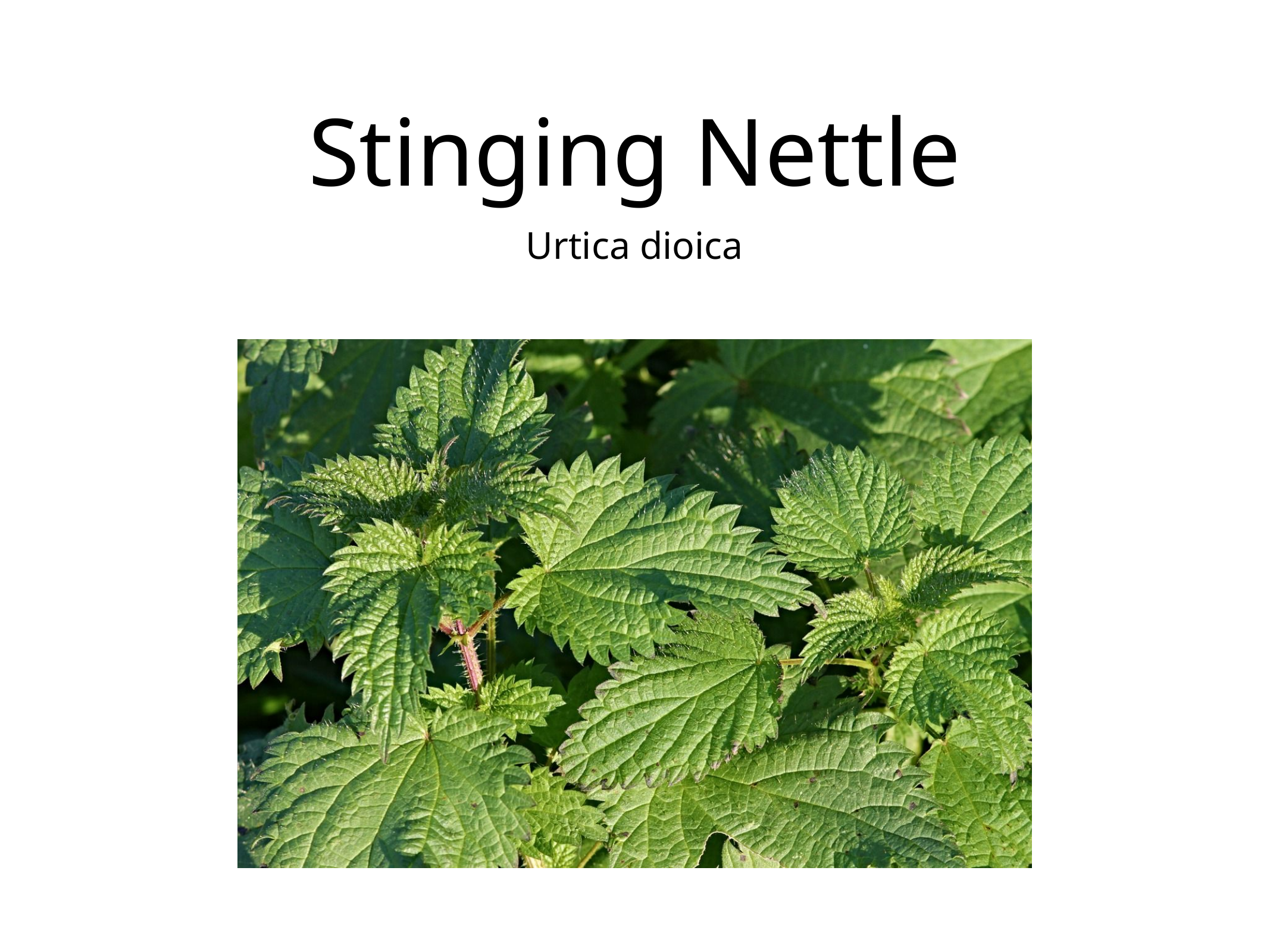

# Stinging Nettle
Urtica dioica

## Slide 35
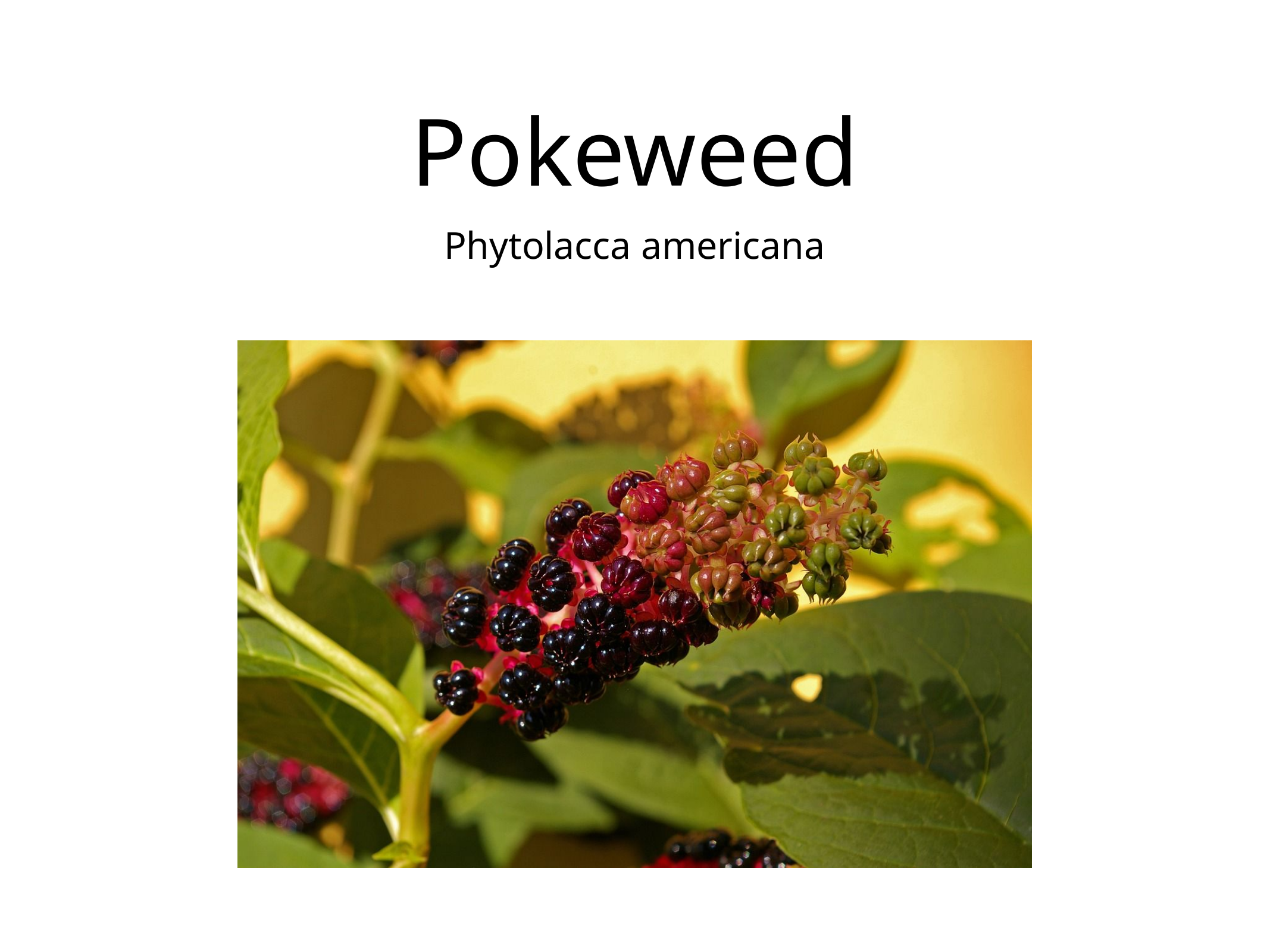

# Pokeweed
Phytolacca americana

## Slide 36
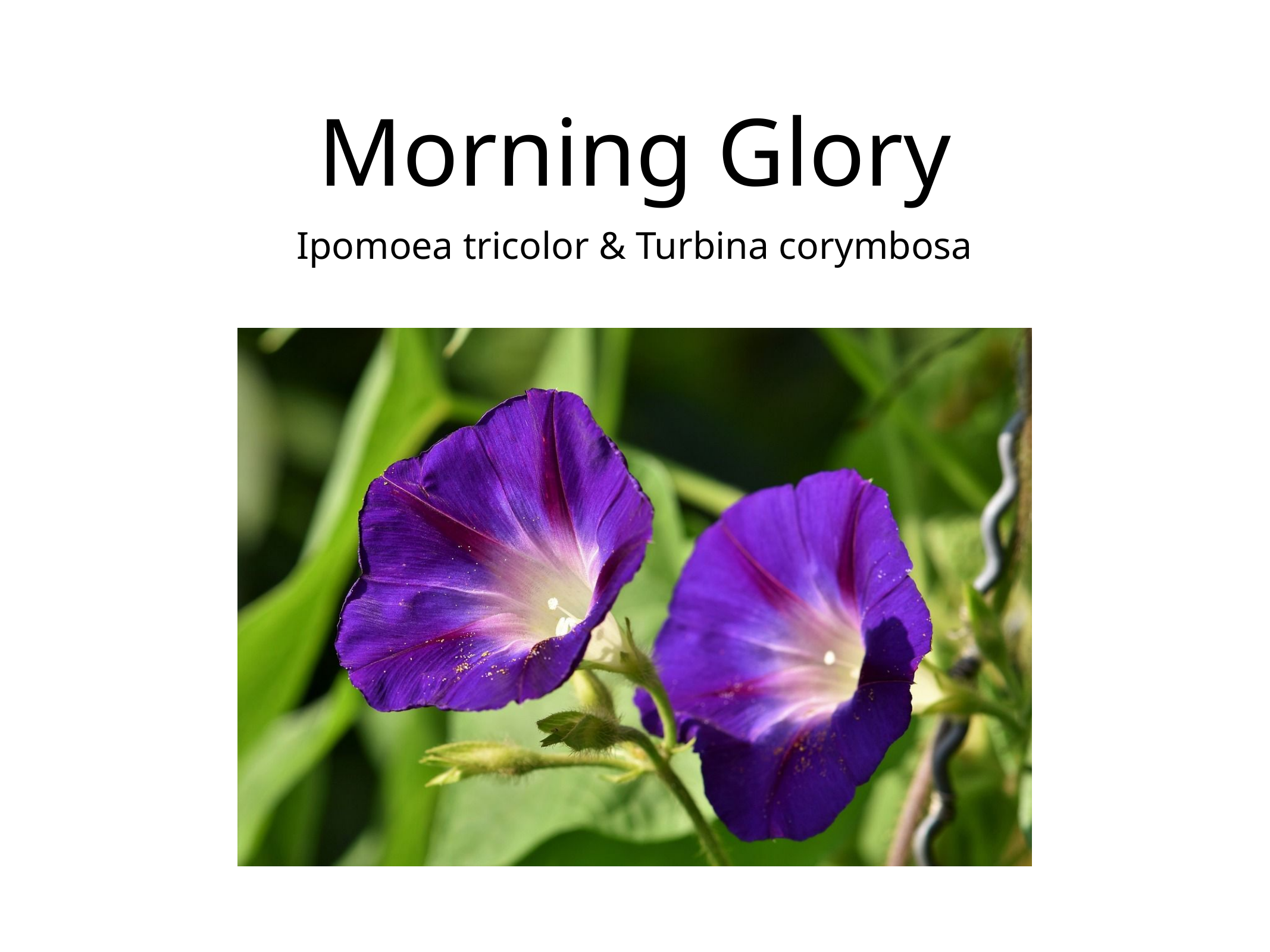

# Morning Glory
Ipomoea tricolor & Turbina corymbosa
